# Cobalt-catalyzed enantioselective intramolecular reductive cyclization via electrochemistry

**DOI:** 10.1038/s41467-023-36704-9

**Published:** 2023-03-09

**Authors:** Shiquan Gao, Chen Wang, Junfeng Yang, Junliang Zhang

**Affiliations:** 1 Department of Chemistry, Fudan University, 2005 Songhu Road, Shanghai, 200438 China; 2 Zhejiang Key Laboratory of Alternative Technologies for Fine Chemical Process, Shaoxing University, Shaoxing, 312000 China; 3 Fudan Zhangjiang Institute, Shanghai, 201203 China

**Keywords:** Synthetic chemistry methodology, Asymmetric catalysis, Electrocatalysis

## Abstract

Transition-metal catalyzed asymmetric cyclization of 1,6-enynes has emerged as a powerful method for the construction of carbocycles and heterocycles. However, very rare examples worked under electrochemical conditions. We report herein a Co-catalyzed enantioselective intramolecular reductive coupling of enynes via electrochemistry using H_2_O as hydride source. The products were obtained in good yields with high regio- and enantioselectivities. It represents the rare progress on the cobalt-catalyzed enantioselective transformation via electrochemistry with a general substrate scope. DFT studies explored the possible reaction pathways and revealed that the oxidative cyclization of enynes by LCo(I) is more favorable than oxidative addition of H_2_O or other pathways.

## Abstract (web summary)

1,6 enynes are a useful synthetic building block, but their use asymmetric intramolecular reductive couplings with cobalt catalysts have not been well-explored. Here, the authors describe an enantioselective intramolecular reductive coupling of enynes via the combination of electrochemistry and cobalt catalysis.

## Introduction

The stereoselective synthesis of optically active functionalized carbo- and heterocycles receives significant attention in organic synthesis due to the prevalence of these chiral scaffolds in the core structures of numerous bioactive natural products and pharmaceuticals. To this end, 1,6-enynes have emerged as versatile substrates in catalytic reactions to prepare cyclic skeletons with a broad range of functional moieties, which enable the direct transformation of a linear substrate to a cyclic product^1–9^. In these methods, noble metals have been most widely explored as the foremost studied catalyst^10–16^.

**Fig. 1:**
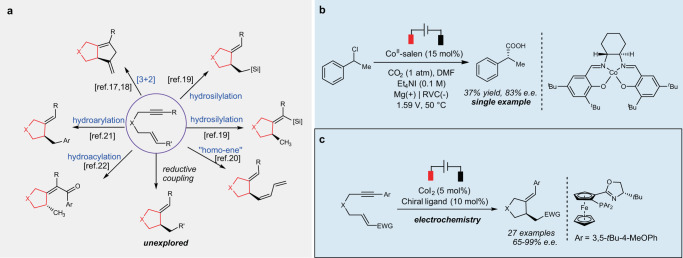
Background and project synopsis. **a** Asymmetric synthetic approaches involving 1,6-enynes by cobalt. **b** Precedent enantioselective carboxylation via electrochemistry by cobalt. **c** This work: enantioselective reductive cyclization of 1,6-enynes via electrochemistry by cobalt.

Recently, cobalt has emerged as a cost-effective alternative in the enantioselective cyclization-coupling reaction of 1,6-enynes and reveals unique reactivity patterns (Fig. 1a). Known methods include [3 + 2] cyclization^17,18^, hydrosilylation^19^, homo-ene cyclization^20^, hydroarylative^21^ and hydroacylative cyclization^22^, depending on the functional groups and the experimental conditions. Most of these reactions are proposed to be initiated by the formation of low-valent cobalt species, which promotes the alkyne/alkene oxidative cyclization to form a cobaltacyclopentane intermediate, followed by the subsequent transformation. Despite these advances, cobalt-catalyzed asymmetric intramolecular reductive coupling of 1,6-enyne has rarely been realized, which, if successful, would provide a protocol for stereoselective construction of ring systems decorated with exocyclic trisubstituted C = C bonds^23^.

Different from the known transformations shown in Fig. 1a, where the catalytic amount of reductant is used to initiate the catalytic cycle, reductive couplings are generally achieved via the use of stoichiometric amount of metallic reductants^24–26^, organo- metallic reagents^27^ or photocatalysis^28^. Recently, electrosynthesis has been revived as a reliable alternative to the conventional methods and could be utilized to replace hazardous reductants by electric current^29–50^. Because the reduction potential and current can be adjusted, electrochemistry offers precise, selective formation of reactive species, thus avoids designing a new reagent. Such tuning is impossible in the use of organic reductants or photocatalysis. These advantages render electrochemistry a sustainable, economical technique for chemical synthesis. However, rare examples have been realized in the field of asymmetric electrosynthesis^51–55^. To our best knowledge, there is only one enantioselective report involving cobalt via electrochemistry, in which moderate enantioselectivity was reported with one single example (Fig. 1b)^56^.

Herein, we report our progress on the cobalt-catalyzed asymmetric intramolecular reductive coupling of enynes, affording chiral five-membered cyclic compounds, which represents the rare progress on the cobalt-catalyzed enantioselective transformation via electrochemistry with a general substrate scope (Fig. 1c).

## Results and discussion

### Optimization of the reaction conditions

Prompted by the precedents on Co-catalyzed electroreduction of aryl halides^57–59^, we initiated the present study with the exploration of enyne **1a** as starting material in the presence of CoI_2_ and phosphine ligand with Zn as sacrificial anode and Ni foam as cathode in CH_3_CN at 80 ^o^C (Table 1). Et_4_NI is employed as the supporting electrolyte. Inspired by the good performance of bisphosphine ligands in cobalt catalysis, we first examined different chiral bisphosphine ligands. The use of (*S*)-BINAP ligand (**L1**) led to the reductive product **2a** in 19% yield with 53% ee. Quinoxp-type ligand (**L2**) improved the yield slightly, but resulted in poor enantioselectivity. Segphos (**L3**) delivered trace product. Sadphos ligands, such as PC-Phos (**L3**) and Ming-Phos (**L4**) were also tested, delivering lower yields and enantioselectivities. To our delight, the desired product could be obtained in 70% yield with 83% ee when Phosferrox ligand **L6** was employed. Evaluation of a series of Phosferrox ligands showed that the use of **L9** gave the best results, affording **2a** in 83% yield with 92% ee. The counterion on the cobalt has a significant effect. Replacement of CoI_2_ to CoCl_2_ or CoBr_2_ resulted in lower reaction efficiency, which may be attributed to reduction potential difference of cobalt complex (Table 1, entries 2-3). Not only the counterion on cobalt, the electrolyte also affected the reactivity and enantioselectivity significantly. Extensive screening indicated that the Et_4_NI gave better performance than other ammonium salts (Table 1, entry 4, for details, see Supplementary Table 4 and 5). It could be rationalized that the produced ZnI_2_ may work as Lewis acid to activate the acrylate and remove the iodide from the LCo(I)I complex, thereby generating a more active cationic cobalt complex^25^. Furthermore, the effect of the electrodes was also examined. Replacing the Zn anode with Al or Mg led to lower yields and enantioselectivities (Table 1, entries 5, 6). As for the cathode, C felt and Pt cathode also promoted the transformation with retention of the enantioselectivity, albeit with lower yield (Table 1, entries 7, 8). The choice of solvent is crucial to the reaction. CH_3_CN, DMA, and THF were not effective for this reaction (Table 1, entries 9-11). Furthermore, constant current showed better performance than constant voltage (Table 1, entry 12). Regarding to the hydride source, it is gratifying to find that water could be used as the environmentally benign choice^60–62^. Finally, control experiments confirmed that the cobalt catalyst, ligand, and electric current are all essential for this transformation. None or trace amount of cyclization product was formed in the absence of these reaction components (Table 1, entries 13–15).

**Table 1:** Reaction optimization


Entry	Deviation from the standard conditions	Yield [%]^*b*^	Ee [%]^*c*^
1	No change	83	92
2	CoCl_2_ was used instead of CoI_2_	12	45
3	CoBr_2_ was used instead of CoI_2_	30	55
4	Et_4_NBF_4_ as electrolyte	NR	–
5	Al as anode	NR	–
6	Mg as anode	NR	–
7	Graphite as cathode	13	75
8	Pt as cathode	56	87
9	CH_3_CN as solvent	NR	–
10	DMA as solvent	NR	–
11	THF as solvent	NR	–
12	Ucell = 2.0 V (CV)	23	92
13	No Co	NR	–
14	No electric current	NR	–
15	No ligand	10	–


^a^Reaction conditions: The reaction was performed using 0.4 mmol of 1a (0.1 M), CoI2 (5 mol%), L9 (10 mol%), H2O (1 equiv) Et4NI (0.2 mmol), CH3CN (3 mL) and THF (1 mL) in undivided cell with zinc anode and Ni foam cathode under constant current conditions (4 mA) for 36 h. ^b^Yield was determined by GC using 1,3-dimethoxy benzene as internal standard. ^c^Determined by HPLC using a chiral stationary phase. *NR* no reaction.

### Scope of the reaction

**Fig. 2:**
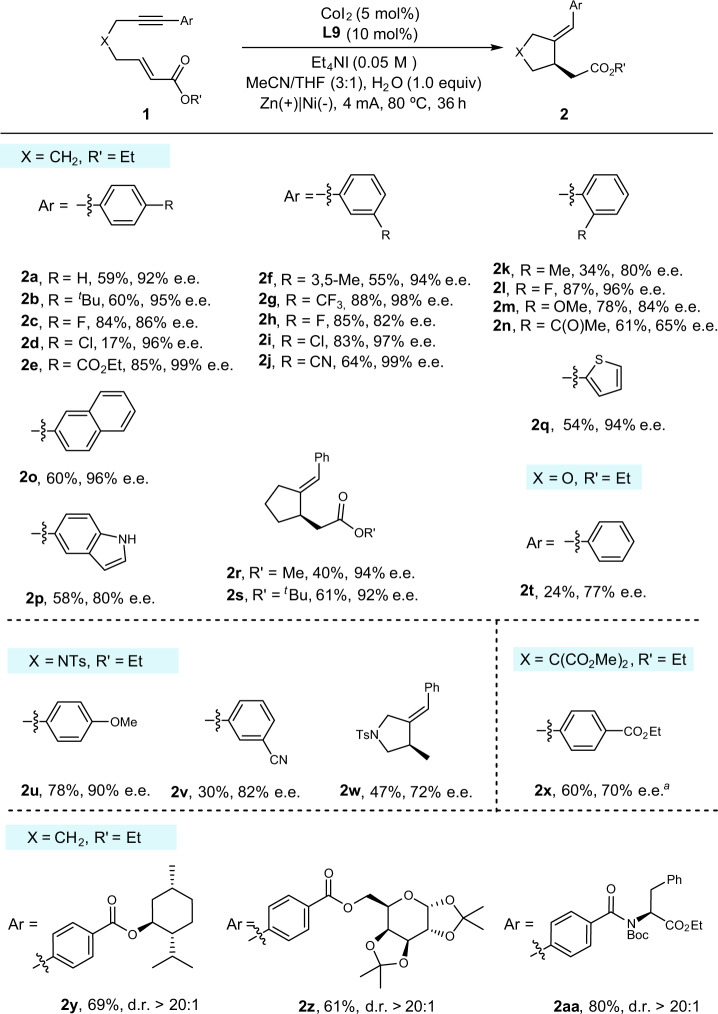
Exploration of substrate scope. The reaction was performed on a 0.4 mmol scale under the conditions in Table 1, entry 1. ^*a*^**L8** was used as ligand.

With the optimal conditions established, the substrate scope was next explored to probe the generality and to identify the limitation of this transformation. As depicted in Fig. 2, carbon tethered enynes with a wide range of aromatic alkyne bearing electron-rich and electron-deficient group at the *para*-, *meta*- and *ortho*-position were suitable to this Co-catalyzed electrosynthesis system (**2a**-**2n**). Fluoro (**2c**), ester (**2e**), cyano (**2j**), and trifluoromethyl group (**2g**) were well tolerated in the reaction, delivering the desired product in good yields with good to excellent enantioselectivities. However, the reaction was found to be sensitive to steric influence on the benzene ring because the *ortho*-substitutents have negative effects on the enantioselectivities. Moreover, the *para*-chloro group (**2d**) on alkyne were not well compatible, probably due to their sensitivity to Co(I) species^63,64^. Besides the chloride, the ketone group (**2n**) is also not well tolerated in the current electrosynthesis system, as it may tend to undergo cathode reduction^65^.

Gratifyingly, heterocycle-substituted alkyne, such as indole (**2p**) and thiophene (**2q**), proceeded in a generally efficient manner. Apart from the carbon-tethered enynes, oxygen-tethered enyne also participated in the reaction, though resulting in low yield and moderate enantioselectivity (**2t**). Pleasingly, nitrogen-tethered ones also underwent reductive coupling to afford substituted tetrahydropyrroles in good yield and enantioselectivity (**2u**-**2v**). Unactivated alkenes were also examined, however, the lower yield and enantioselectivity was obtained under standard conditions (**2w**). The absolute configuration of **2w** was determined by its single crystal X-ray analysis, and those of the others were assigned analogously^66^. Furthermore, to demonstrate the synthetic utility of this electrosynthesis protocol, it was subjected to the late-stage modification of natural products and derivatives. For example, bioactive alkynes containing menthol, glucose, and amino acid underwent the desired coupling to afford the desired products with good yields and excellent diastereoselectivities (**2y**-**2aa**).

### Mechanistic study

**Fig. 3:**
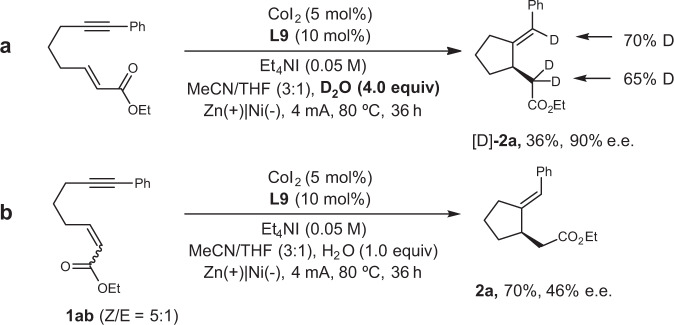
Deuterium labelling and control experiments. **a** Deuterium labelling experiment. **b** Control experiment on geometry of the alkene.

To understand the role of water and gain insight into the mechanism, an isotope labelling experiment using D_2_O (4.0 equiv) to replace normal water was conducted. The expected product was obtained with substantial deuterium incorporation into both the vinyl and α-carbonyl position (70% and 65%) (Fig. 3a). In addition, when using the enyne with modest alkene Z/E ratio as starting materials, the product with poor enantioselectivity (46% ee) was obtained. This result revealed that the stereochemistry of the alkene has significant effect on the enantio- control (Fig. 3b)^67,68^.

**Fig. 4:**
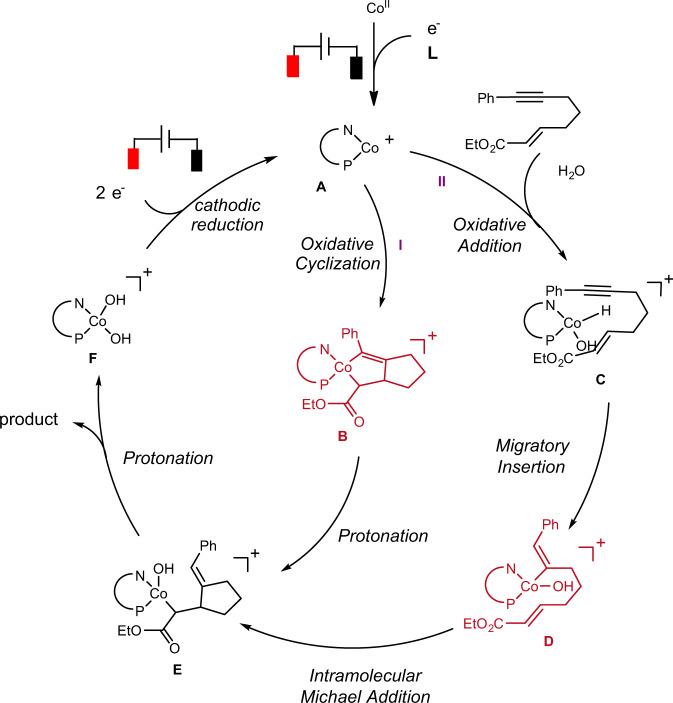
Proposed catalytic cycle. Two plausible different reaction pathways for reductive cyclization of 1,6-enynes.

On the basis of the above results and previous studies^24–26^, we proposed the putative pathways for the reaction (Fig. 4). Initially, the cathode reduction allows the LCo(II) to afford the LCo(I) species **A**, which then undergoes the oxidative cyclization to produce cobaltacyclopentene intermediate **B**. Then double protonation of **B** gives the product and LCo(III)(OH)_2_ species **F**. Finally, cathode reduction of LCo(III)(OH)_2_ regenerates the LCo(I) species **A**. Alternatively, it is also speculated that LCo(I) species A may react with H_2_O to afford LCo(III)H(OH) intermediate **C**, which undergoes alkyne migratory insertion to produce the vinyl Co(III) species **D**. Subsequent intramolecular 1,4-addition delivers species **E**, followed by the protonation to give the product and LCo(III)(OH)_2_ species **F**.

Both pathways well explained the isotope labelling experiment and the observed regioselectivity of alkynes. In order to better understand the reaction pathway, particularly which pathway is more favorable, DFT calculations were carried out to probe the proposed mechanism. To reduce the computational cost, the calculations were performed on a model reaction using carbon tethered enyne: methyl (*E*)-non-2-en-7-ynoate and (dppe)Co(I) (singlet or triplet) as the starting complex^17,18,20–22^.

**Fig. 5:**
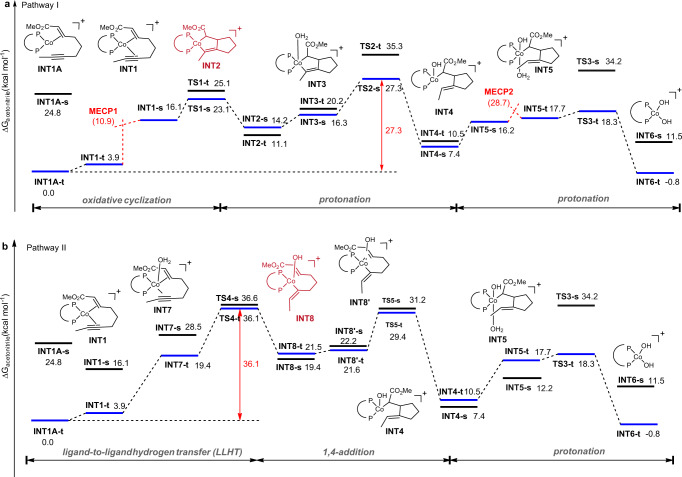
Gibbs energy profile for Pathway I and Pathway II. All calculations were performed at the SMD(acetonitrile)-M06L/6-311 + G(d,p)-SDD(Co)//M06L/6-31G(d)-SDD(Co) level. **a** Gibbs energy profile for Pathway I. **b** Gibbs energy profile for Pathway II.

Reaction pathways and energy diagrams for the pathway I and pathway II are summarized in Fig. 5a and b, respectively. Note that the suffixes s and t on the structure numberings refer to the singlet and triplet states, respectively. The cationic [(dppe)Co]^+^ prefers to form the mono-binding complex. The calculation shows that the alkene-binding complex (**INT1A**) is more stable than the alkyne-binding complex (**INT1B**) and enyne-binding complex (**INT1**) by 2.9 and 3.9 kcal mol^−1^ (for details, see Supplementary Fig. 4). The singlet species **INT1A-s** was calculated to be less stable than triplet counterparts **INT1A-t** (ΔG = 24.8 and 0 kcal mol^−1^, respectively). With the alkyne coordination to the cobalt, it forms enyne-binding complex **INT1**. Furthermore, the singlet species **INT1-s** was calculated to be less stable than triplet counterparts **INT1-t** (ΔG = 16.1 and 3.9 kcal mol^−1^, respectively). **INT1-s** may be connected to the corresponding triplet intermediate **INT1-t** by minimum energy crossing point (**MECP1**) with ΔG of 10.9 kcal mol^−1^. The subsequent oxidative cyclometalation to form cationic Co(III) metallacycle **INT2-s** was found to take place with an energy barrier of 23.1 kcal mol^-1^ (via **TS1-s**). Next, after the binding of H_2_O, protonation of the Co(III) metallacycle **INT3-s** afforded an C-enolate Co(III) species **INT4-s**. Here, the singlet TS (**TS2-s**, ΔG = 27.3 kcal mol^−1^) was found to be lower in energy than the triplet TS (**TS2-t**, ΔG = 35.3 kcal mol^−1^). Before the second protonation occurs, there is another **MECP2** with ΔG of 28.7 kcal mol^−1^ to connect the triplet state (**INT5-t**, ΔG = 17.7 kcal mol^−1^) and singlet state (**INT5-s**, ΔG = 16.2 kcal mol^−1^), as the following protonation preferentially occurs in triplet state (**TS3-t**, ΔG = 18.3 kcal mol^−1^) than the singlet state (**TS3-s**, ΔG = 34.2 kcal mol^−1^), forming the product complex **INT6-t** (ΔG = −0.8 kcal mol^-1^). Overall, the first protonation was found to be the step of the highest activation barrier (ΔG^#^ = 27.3 kcal mol^−1^). Alternatively, the first protonation might also afford an alkenyl Co(III) species (**INT10-t**). However, this pathway showed the higher activation barrier (ΔG^#^ = 29.8 kcal mol^−1^, see Fig. S5 for details).

Besides the oxidative cyclization pathway (Fig. 5a), we also probed the oxidative addition and migratory insertion of the enyne into the Co-H bond, which converges to **INT8-t** (Fig. 5b). However, our attempts to locate a H_2_O oxidative addition TS in both singlet and triplet state failed and the C-H bond cleavage was found to take place favorably through a ligand-to-ligand hydrogen transfer (LLHT) transition state^69–76^, where the Co-OH bond, Co-alkenyl bond, and the vinyl C-H bond form in a concerted approach with an overall energy barrier of 36.1 kcal mol-1. Next, the intramolecular 1,4-addition preferentially occurs in the triplet state (**TS5-t**, ΔG = 29.4 kcal mol^-1^). Overall, the LLHT step was found to require the highest activation energy (ΔG^#^ = 36.1 kcal mol^−1^). Alternatively, the LLHT to afford the C-enolate Co(III) species **INT9-t** was also explored and the result showed that it required even higher activation energy (ΔG^#^ = 48.2 kcal mol^−1^, see Fig. S5 for details). As a result, the energy gap of the two pathways suggests that the present reaction is likely to occur initially by oxidative cyclization rather than the stepwise oxidative addition−migratory insertion or LLHT mechanism.

In summary, we have successfully developed a Co-catalyzed enantioselective intramolecular reductive cyclization of enynes via electrochemistry. The products are generated in a highly stereocontrolled fashion in good yields and represent a powerful platform to access five-member ring-bearing exocyclic trisubstituted C=C bonds. Mechanistic studies by DFT shed light on the potential reaction pathway, indicating that the oxidative cyclization is more favorable than ligand-to-ligand hydrogen transfer pathway. Given the increasing interests in electrosynthesis and cobalt catalysis, we believe this report will stimulate further investigations in asymmetric organoelectrochemistry.

## Methods

### Representative procedure for cobalt-catalyzed enantioselective intramolecular reductive cyclization via electrochemistry

Under a nitrogen atmosphere, an oven-dried electrochemical cell with two stir bars was added enyne (0.4 mmol, 1 equiv), CoI_2_ (0.02 mmol, 5 mol%), ligand (0.04 mmol, 10 mol%), Et_4_NI (0.2 mmol, 0.5 equiv), H_2_O (1.0 equiv), 3 mL of CH_3_CN and 1 mL of THF. The tube was installed by a Ni foam (2.5 cm × 0.5 cm) as cathode and Zn flake (2.5 cm × 0.5 cm) as sacrificial anode. The distance of the electrodes is around 1 cm. The mixture was stirred at r.t. for 15 min. The reaction mixture was electrolyzed under a constant current of 4 mA at 80 °C until the complete consumption of the starting materials which was monitored by TLC (about 36 hours). The solvent was removed in vacuo, and the crude residue was purified via column chromatography to afford the desired product.

## Supplementary information

Supplementary Information

Description of Additional Supplementary Files

Supplementary Data 1

Supplementary Data 2

## Data Availability

The authors declare that all the data supporting this study, including the experimental details, data analysis, and spectra for all unknow compounds, see Supplementary Files. All data underlying the findings of this work are available from the corresponding author upon reasonable request. Crystal data and structure refinement for **2w** is available in Supplementary Data 1. Energy data (hartrees) for the calculated structures is available in Supplementary Data 2. The X-ray crystallographic coordinates for structures reported in this study have been deposited at the Cambridge Crystallographic Data Centre (CCDC), under deposition numbers 2184064 (**2w**). These data are provided free of charge by the joint Cambridge Crystallographic Data Centre and Fachinformationszentrum Karlsruhe Access Structures service www.ccdc.cam.ac.uk/structures.
