# Supplementary material for: Cobalt-catalyzed enantioselective intramolecular reductive cyclization via electrochemistry
Source: Nat Commun. 2023 Mar 9;14:1301. doi: 10.1038/s41467-023-36704-9 (PMC9998880; doi:10.1038/s41467-023-36704-9)
Supplement: Supplementary file 1 — Supplementary Information [file 41467_2023_36704_MOESM1_ESM.pdf]

Supplementary Information

Cobalt-Catalyzed Enantioselective Intramolecular Reductive Coupling of Enynes via Electrochemistry

Shiquan Gao,[1] Chen Wang,[2] Junfeng Yang,[1,3]* and Junliang Zhang[1]*

[1]Department of Chemistry, Fudan University, 2005 Songhu Road, Shanghai, 200438, China

[2]Zhejiang Key Laboratory of Alternative Technologies for Fine Chemical Process, Shaoxing University, Shaoxing 312000, China

[3]Fudan Zhangjiang Institute, Shanghai 201203, China

Table of Contents

1. Supplementary Methods…………………………………………….……………...S3

2. Supplementary Discussion………………………………………………...…..S4

2.1 Supplementary Table 1 Screening of Sadphos ligands…..…………..……..S4

2.2 Supplementary Table 2 Screening of commercially available chiral ligands..S5

2.3 Supplementary Table 3 Screening of solvents and electrolytes...…………….S6

2.4 Supplementary Table 4 Screening of [Co], electrolytes and voltage…………S7

2.5 Supplementary Table 5 Screening of the electrodes and electrolytes………..S8

2.6 Supplementary Table 6 Screening of [Co] and electrolytes…..………….…..S9

2.7 Supplementary Table 7 Screening of reaction time, current and voltage….S10

3. Supplementary Notes…………………………………..…..……………S11

3.1 Synthesis and characterization data for L28…………………..……..……S11

3.2 General procedure A for the synthesis of 1…………………………….………S12

3.3 General procedure B for the synthesis of 2……………………………………S25

3.4 Deuterium labelling and control experiments………………………………..S49

3.5 X-Ray crystal structures…………………………..…….…..……….……..S52

3.6 DFT calculation……..………………………..……………………….……..S53

3.7 NMR spectra………...…………………………………………...………...S190

4. Supplementary reference……………..……..…………………...…….……S250

1. Supplementary Methods

All reactions dealing with air- or moisture-sensitive compound were performed by standard Schlenk techniques in oven-dried reaction vessels under nitrogen atmosphere or in the argon-filled glove box. ^{1}H, ^{13}C, ^{31}P and ^{19}F NMR spectra were recorded on Bruker AV-400 (400 MHz) NMR spectrometers. ^{1}H and ^{13}C NMR spectra are reported in parts per million (ppm) downfield from an internal standard, tetramethylsilane (0 ppm) and CHCl3 (77.0 ppm), respectively. ^{19}F NMR spectra are reported in terms of chemical shift (ppm) relative to external peak (CF_3Ph: -62.74 ppm). Chiral HPLC analysis was performed on a Shimadzu LC-20AD instrument using Daicel Chiralpak columns IA, IG, IF, OJ-H, AD-H, AS-H, IB N-5, OD-H at room temperature (25–29 ℃). The instrumentation used for the crystal measurement was D8 VENTURE MetalJet. HRMS spectra were recorded on GCQTOF 7200. Reactions were monitored by thin layer chromatography (TLC) using silica gel plates. Flash column chromatography was performed over silica gel (300-400 mesh ASTM, purchased from Yantai, China). Melting point data were recorded on WRS-2 and were uncorrected.

Materials. Unless otherwise noted, commercial reagents were purchased from Energy chemical, Bidepharm, Aesar, and other commercial suppliers and were used as received. MeCN was freshly distilled from CaH_2. THF was freshly distilled from sodium metal prior to use. Nickel foam was purchased from TAOBAO (https://item.taobao.com/item.htm?spm=a1z09.2.0.0.3b142e8doL22fu&id=61346203 1143&_u=51bta621701); Carbon felt was purchased from Beijing Jinglong Special Carbon Technology Co., Ltd.

2. Supplementary Discussion

2.1 Supplementary Table 1. Screening of Sadphos ligands

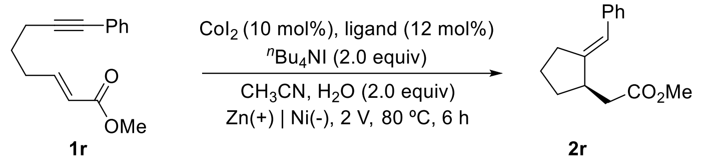

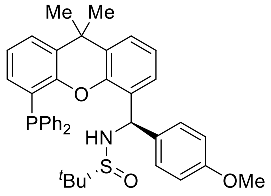	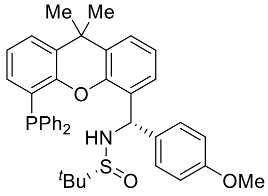	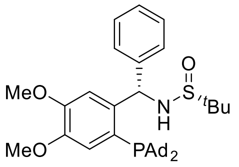	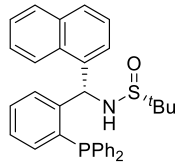
**L1**, 27% yield<br>*E/Z* = 1.5:1, 0% *ee*, 0% *ee*	**L2**, 26% yield<br>*E/Z* = 2.2:1, 0% *ee*, 0% *ee*	**L3**, 20% yield<br>*E/Z* = 2.6:1, 0% *ee*, 0% *ee*	**L4**, 34% yield<br>*E/Z* = 1.2:1, 0% *ee*, 0% *ee*
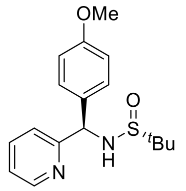	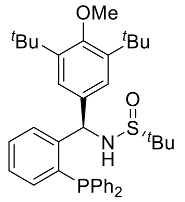	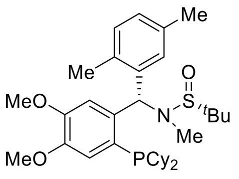	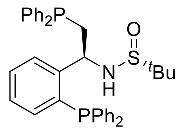
**L5**, 26% yield<br>*E/Z* = 10:1, 0% *ee*, 0% *ee*	**L6**, 55% yield<br>*E/Z* = 1.4:1 , 0% *ee*, 0% *ee*	**L7**, 29% yield<br>*E/Z* = 2:1, 0% *ee*, 0% *ee*	**L8**, 48% yield<br>*E/Z* = 4:1, -9% *ee*, -8% *ee*
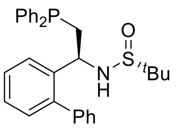	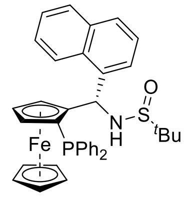	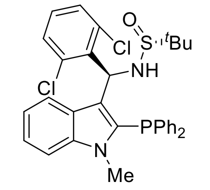	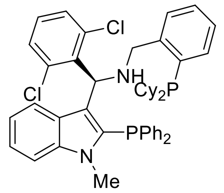
**L9**, 42% yield<br>*E/Z* = 4:1 , 0% *ee*, 0% *ee*	**L10**, 46% yield<br>*E/Z* = 15:1, 0% *ee*, -23% *ee*	**L11**, 29% yield<br>*E/Z* = 3:1, 0% *ee*, -28% *ee*	**L12**, 17% yield<br>*E/Z* = 13:1, 0% *ee*, -8% *ee*

Note: CoI_2 (10 mol%), ligand (12 mol%), H_2O (2.0 equiv), **1r** (0.2 mmol), $^nBu_4NI$ (2.0 equiv) and MeCN (4 mL) were added to the electrochemical reaction tube at room temperature in a glove box. Zinc flakes are used as the anode, and nickel foam is used as the cathode. The reaction mixture was electrolyzed at 80 °C for 6 h at a constant voltage of 2 V. The yield and *Z/E* selectivity were determined by GC, and the internal standard was *m*–dimethoxybenzene. *Ee* was determined by HPLC.

2.2 Supplementary Table 2. Screening of commercially available chiral ligands

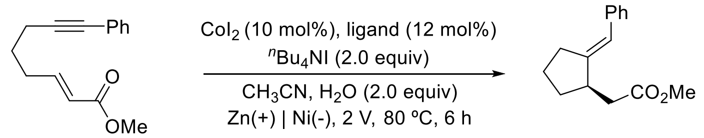

1r **2r**

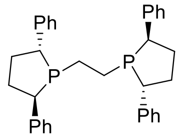

L13, 38% yield
E/Z = 38:1, 0% *ee*, 38% *ee*

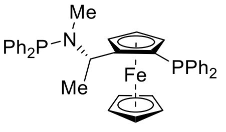

L14, 22% yield
E/Z = 1.5:1, 7% *ee*, 7% *ee*

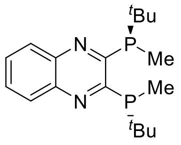

L15, 22% yield
E/Z = 2:1, 82% *ee*, 35% *ee*

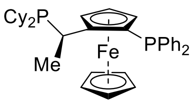

L16, 38% yield
E/Z = 1.2:1, 0% *ee*, -16% *ee*

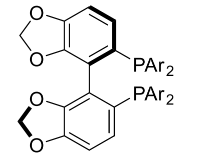

Ar = 3,5-$^tBu_2$-4-MeOC_6H_2
L17, 40% yield
E/Z = 1.5:1, 0% *ee*, 0% *ee*

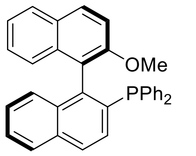

L18, 46% yield
E/Z = 25:1, 0% *ee*, 0% *ee*

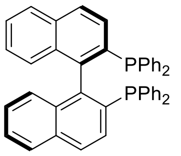

L19, 43% yield
E/Z = 1:1, 0% *ee*, 0% *ee*

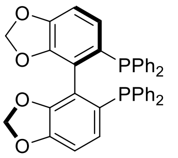

L20, 67% yield
E/Z = 25:1, 0% *ee*, -14% *ee*

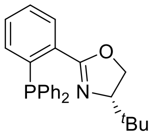

L21, 26% yield
E/Z = 1:0, 0% *ee*, -32% *ee*

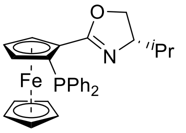

L22, 60% yield
E/Z = 6.5:1, -78% *ee*, -71% *ee*

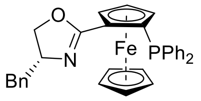

L23, 47% yield
E/Z = 3:1, 21% *ee*, 66% *ee*

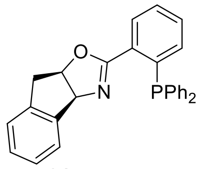

L24, 26% yield
E/Z = 1:1, -7% *ee*, -7% *ee*

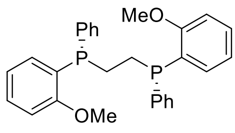

L25, 40% yield
E/Z = 34:1, 0% *ee*, 0% *ee*

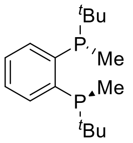

L26, 22% yield
E/Z = 1.6:1, -41% *ee*, 0% *ee*

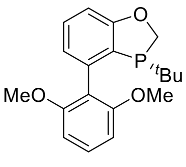

L27, 38% yield
E/Z = 2:1, 0% *ee*, 0% *ee*

Note: CoI_2 (10 mol%), ligand (12 mol%), H_2O (2.0 equiv), **1r** (0.2 mmol), $^nBu_4NI$ (2.0 equiv), and MeCN (4 mL) were added to the electrochemical reaction tube at room temperature in a glove box. Zinc flakes are used as the anode, and nickel foam is used as the cathode. The reaction mixture was electrolyzed at 80 °C for 6 h at a constant voltage of 2.0 V. The yield and *E/Z* selectivity were determined by GC, and the internal standard was *m*–dimethoxybenzene. *Ee* was determined by HPLC.

2.3 Supplementary Table 3. Screening of solvents and electrolytes

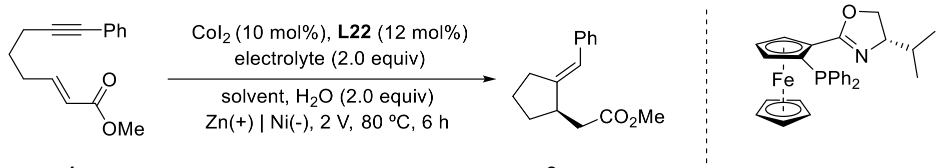

1r **2r**

entry	solvent	electrolyte	yield (%)	*E/Z*	*ee*%
1	DMF	^{n}Bu$_4$NI	13	5:1	89
2	DMA	^{n}Bu$_4$NI	21	1.5:1	81
3	^{i}PrCN	^{n}Bu$_4$NI	43	1:0	82
4	MeCN	^{n}Bu$_4$NI	13	1:0	64
5	MeCN	Et$_4$NI	18	7:1	90
6	MeCN:Dioxane (3:1)	Et$_4$NI	32	7:1	80
7	MeCN:DMF (3:1)	Et$_4$NI	51	10:1	87
8	MeCN: DMA (3:1)	Et$_4$NI	14	2:1	50
9	MeCN: ^{i}PrCN (3:1)	Et$_4$NI	37	15:1	81

Note: CoI_2 (10 mol%), **L22** (12 mol%), H_2O (2.0 equiv), **1r** (0.2 mmol), ^{n}Bu$_4$NI or Et$_4$NI (2.0 equiv) and the solvent were added to the electrochemical reaction tube at room temperature in a glove box. Zinc flakes are used as the anode, and nickel foam is used as the cathode. The reaction mixture was electrolyzed at 80 °C for 6 h at a constant voltage of 2.0 V. The yield and *E/Z* selectivity were determined by GC, and the internal standard was *m*–dimethoxybenzene. *Ee* was determined by HPLC.

2.4 Supplementary Table 4. Screening of [Co], electrolytes and voltage

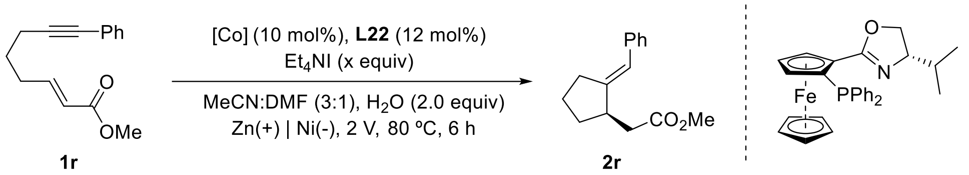

entry	[Co]	Et_4NI	V	yield (%)	*E/Z*	*ee*%
1	$Co(OAc)_2 \cdot H_2O$	2.0 equiv	2 V	22	10:1	83
2	$Co(acac)_2$	2.0 equiv	2 V	40	10:1	75
3	$CoCl_2$	2.0 equiv	2 V	18	5:1	55
4	$CoBr_2$	2.0 equiv	2 V	30	20:1	79
5	CoI_2	2.0 equiv	2 V	46	12:1	86
6	CoI_2	1.0 equiv	2 V	55	9:1	89
7	CoI_2	0.5 equiv	2 V	56	10:1	89
8	CoI_2	2.0 equiv	1.5 V	37	20:1	84
9	CoI_2	2.0 equiv	2.5 V	41	20:1	87

Note: [Co] (10 mol%), **L22** (12 mol%), H_2O (2.0 equiv), **1r** (0.2 mmol), Et_4NI, MeCN (3 mL) and DMF (1 mL) were added to the electrochemical reaction tube at room temperature in a glove box. Zinc flakes are used as the anode, and nickel foam is used as the cathode. The reaction mixture was electrolyzed at 80 °C for 6 h at a constant voltage of 1.5 – 2.5 V. The yield and *E/Z* selectivity were determined by GC, and the internal standard was *m*–dimethoxybenzene. *Ee* was determined by HPLC.

2.5 Supplementary Table 5. Screening of the electrodes and electrolytes

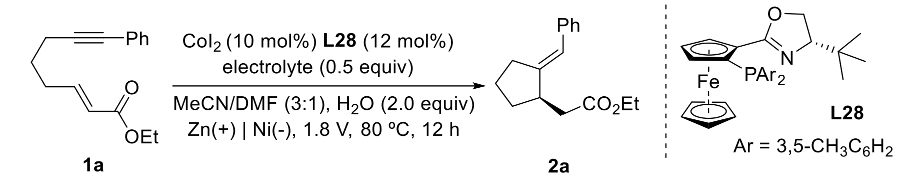

entry	electrode	electrolyte	yield	*ee* (%)
1	Al (+)	Et_4NI	19	40
2	Pt (–)	Et_4NI	16	90
3	Graphite	Et_4NI	10	89
4	C felt (–)	Et_4NI	31	90
5	Zn (+)	Et_4NBF_4	16	29
6	Zn (+)	Et_4NClO_4	36	80
7	Zn (+)	$^nBu_4NPF_6$	30	67
8	Zn (+)	Et_4NOTs	12	80
9	Zn (+)	Et_4NBr	26	67
10	Zn (+)	Bu_4NI	24	83

Note: CoI_2 (10 mol%), **L28** (12 mol%), H_2O (2.0 equiv), **1a** (0.2 mmol), electrolyte (0.5 equiv), MeCN (3 mL) and DMF (1 mL) were added to the electrochemical reaction tube at room temperature in a glove box. The corresponding cathode and anode electrodes were used. The reaction mixture was electrolyzed at 80 °C for 6 h at a constant voltage of 1.8 V. The yield and *E/Z* selectivity were determined by GC, and the internal standard was *m*–dimethoxybenzene. *Ee* was determined by HPLC.

2.6 Supplementary Table 6. Screening of [Co] and electrolytes

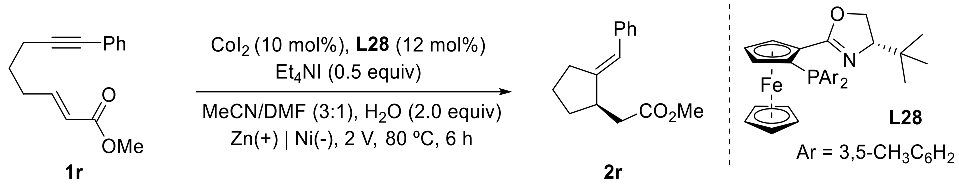

entry	Cat.	Et_4NI	yield	*ee* (%)
1	CoI_2 (10 mol%), **L28** (12 mol%)	0.5 equiv	17	84
2	CoI_2 (10 mol%), **L28** (12 mol%)	1.0 equiv	13	90
3	CoI_2 (10 mol%), **L28** (12 mol%)	2.0 equiv	18	60
4	CoI_2 (10 mol%), **L28** (20 mol%)	0.5 equiv	54	94
5	CoI_2 (8 mol%), **L28** (16 mol%)	0.5 equiv	21	94
6	CoI_2 (6 mol%), **L28** (12 mol%)	0.5 equiv	8	90
7	CoI_2(**L28**) (10 mol%)	0.5 equiv	10	27

Note: CoI_2, **L28**, H_2O (2.0 equiv), **1r** (0.2 mmol), Et_4NI, MeCN (3 mL) and DMF (1 mL) were added to the electrochemical reaction tube at room temperature in a glove box. Zinc flakes are used as the anode, and nickel foam is used as the cathode. The reaction mixture was electrolyzed at 80 °C for 6 h at a constant voltage of 2.0 V. The yield and *E/Z* selectivity were determined by GC, and the internal standard was *m*–dimethoxybenzene. *ee* was determined by HPLC.

2.7 Supplementary Table 7. Screening of reaction time, current and voltage

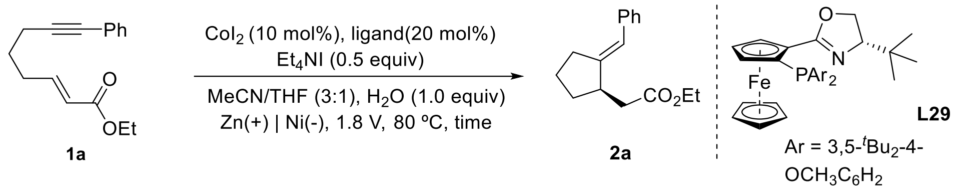

entry	time	CV/CC	L	yield (%)	*ee* (%)
1	3 h	1.8 V	**L28**	9	—
2	2 h	1.8 V	**L28**	11	—
3	1 h	1.8 V	**L28**	40	88
4	5.5 h	2 mA	**L28**	42	85
5	4.5 h	3 mA	**L28**	15	25
6	4.5 h	4 mA	**L28**	75	87
7	3.5 h	5 mA	**L28**	50	85
8	6 h	4 mA	**L29**	82	96
9	21 h<br>3–1b (0.4 mmol)<br>CoI_2 (5 mol%), **L29** (10 mol%)	4 mA	**L29**	64	94

Note: CoI_2, **L28** or **L29**, H_2O (2.0 equiv), **1A** (0.2 mmol), Et_4NI (0.5 equiv) and MeCN:THF = 3:1 (4 mL) were added to the electrochemical reaction tube at room temperature in a glove box. Zinc flakes are used as the anode, and nickel foam is used as the cathode. The reaction mixture was electrolyzed at 80 °C for 6 h at a constant current of 1.0 – 4.0 mA or at a constant voltage of 1.8 V. The yield and *E/Z* selectivity were determined by GC, and the internal standard was *m*–dimethoxybenzene. *Ee* was determined by HPLC.

3. Supplementary Notes

3.1 Synthesis and characterization data for L28

Ligands were prepared according to the literature procedure[1]. NMR spectra of these compounds showed good agreement with the literature data. Below are characterization data for new compounds synthesized by this method:

(*S*,*S*)–1–Bis[3,5–bimethyl–phenyl]phosphine–2–(4–tert–butyloxazolinyl)fer Rocene (L28)

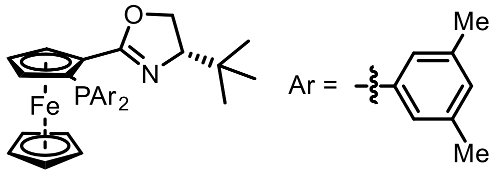

^{1}H NMR (400 MHz, $CDCl_3$) δ 7.15 (ddd, *J* = 7.6, 1.6, 0.8 Hz, 2H), 7.03 (td, *J* = 1.7, 0.9 Hz, 1H), 6.89 – 6.79 (m, 3H), 5.00 (dt, *J* = 2.5, 1.2 Hz, 1H), 4.37 (td, *J* = 2.5, 0.6 Hz, 1H), 4.24 (s, 6H), 3.89 – 3.74 (m, 2H), 3.67 (ddd, *J* = 2.5, 1.5, 0.9 Hz, 1H), 2.34 (s, 6H), 2.22 (s, 6H), 0.81 (s, 9H). **^{13}C NMR** (100 MHz, $CDCl_3$) δ 165.0, 139.2, 139.1, 138.3, 138.1, 137.4, 137.4, 137.1, 137.0, 132.7, 132.5, 130.6, 130.3, 130.1, 129.6, 79.3, 79.1, 75.9, 74.0, 73.9, 72.0, 72.0, 70.7, 70.7, 70.5, 68.5, 33.5, 25.7, 21.4, 21.3. **^{31}P NMR** (162 MHz, $CDCl_3$) δ –16.65. **HRMS (ESI)**: m/z: $[M+H]^+$ Calcd. for $C_{33}H_{39}FeNOP$: 552.2119, found: 552.2114.

3.2 General procedure A for the synthesis of 1

Enyne **1a** and **1w** were prepared according to the literature procedure[2-3]. NMR spectra of these compounds showed good agreement with the literature data.

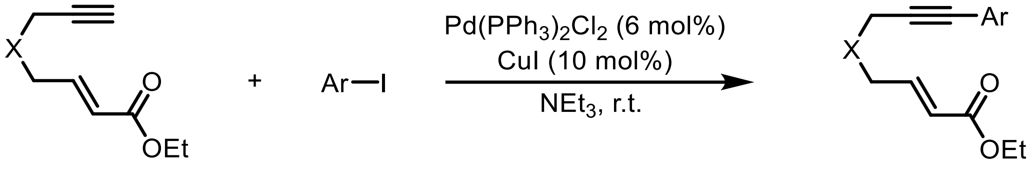

1

A mixture of enyne[4-7] (0.33 g, 2.0 mmol, 1.0 equiv), aromatic iodine (2.2 mmol, 1.1 equiv.), $PdCl_2(PPh_3)_2$ (84.0 mg, 0.12 mmol) and CuI (38.0 mg, 0.2 mmol) were added in an oven dried Schlenk bottle, which are stirred in NEt_3 (15 mL) under argon at room temperature overnight. When the enyne was completely consumed (monitored by TLC), it was quenched with NH_4Cl (aq.) and extracted with Et_2O (20 mL) three times. The organic layer was washed by water and dried over Na_2SO_4. After evaporation of the solvent under vacuum, the product was isolated by column chromatography on silica gel using petroleum ether/ethyl acetate as eluant.

Ethyl *(E)*-8-(4-(tert-butyl)phenyl)oct-2-en-7-ynoate (1b)

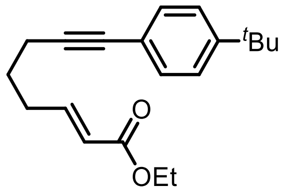

Prepared according to typical procedure **A**, after a flash column chromatography (petroleum ether/ethyl acetate = 20/1) afforded the product as yellow oil (350.5 mg, 59% yield).

^{1}H NMR (400 MHz, $CDCl_3$) δ 7.34 – 7.28 (m, 4H), 7.02 – 6.95 (m, 1H), 5.90 – 5.85 (m, 1H), 4.19 (q, *J* = 7.1 Hz, 2H), 2.46 – 2.35 (m, 4H), 1.80 – 1.73 (m, 2H), 1.30 (s, 9H). **^{13}C NMR** (100 MHz, $CDCl_3$) δ 166.7, 150.8, 148.2, 131.2, 130.1, 125.2, 122.0, 88.3, 81.4, 60.2, 34.7, 31.2, 31.1, 27.1, 18.9, 14.3. **HRMS (EI)**: m/z: $[M]^+$ Calcd. for $C_{20}H_{26}O_2$: 298.1933, found: 298.1923.

Ethyl *(E)*-8-(4-fluorophenyl)oct-2-en-7-ynoate (1c)

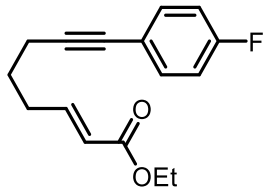

Prepared according to typical procedure **A**, after a flash column chromatography (petroleum ether/ethyl acetate = 20/1) afforded the product as yellow oil (454.1 mg, 87% yield).

^{1}H NMR (400 MHz, $CDCl_3$) δ 7.43 – 7.34 (m, 3H), 7.06 – 6.95 (m, 3H), 5.90 (dt, *J* = 15.6, 1.6 Hz, 1H), 4.21 (q, *J* = 7.1 Hz, 2H), 3.80 – 3.74 (m, 1H), 2.49 – 2.37 (m, 4H), 1.91 – 1.85 (m, 1H), 1.83 – 1.75 (m, 2H), 1.31 (t, *J* = 7.1 Hz, 3H). **^{13}C NMR** (100 MHz, $CDCl_3$) δ 166.5, 162.1 (d, $^{1}J_{C–F}$ = 247.0 Hz), 147.9, 133.3 (d, $^{3}J_{C–F}$ = 9.0 Hz), 122.0, 119.8 (d, $^{4}J_{C–F}$ = 3.0 Hz), 115.4 (d, $^{2}J_{C–F}$ = 22.0 Hz), 88.7, 88.7, 80.3, 60.2, 31.1, 26.9, 18.8, 14.2. **^{19}F NMR** (376 MHz, C_6D_6) δ –112.57. **HRMS (EI)**: m/z: $[M]^+$ Calcd. for $C_{16}H_{17}FO_2$: 260.1213, found: 260.1206.

Ethyl *(E)*–8–(4–chlorophenyl)oct–2–en–7–ynoate (1d)

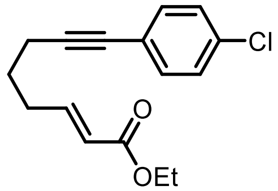

Prepared according to typical procedure **A**, after a flash column chromatography (petroleum ether/ethyl acetate = 20/1) afforded the product as yellow oil (350.6 mg, 63% yield).

^{1}H NMR (400 MHz, $CDCl_3$) δ 7.35 – 7.26 (m, 4H), 7.00 (dt, *J* = 15.7, 7.0 Hz, 1H), 5.90 (d, *J* = 8.0 Hz, 1H), 4.21 (q, *J* = 7.1 Hz, 2H), 2.46 (t, *J* = 7.0 Hz, 2H), 2.44 – 2.37 (m, 2H), 1.83–1.76 (m, 2H), 1.31 (t, *J* = 7.1 Hz, 3H). **^{13}C NMR** (100 MHz, $CDCl_3$) δ 166.6, 147.9, 133.6, 132.8, 128.5, 122.3, 122.1, 90.2, 80.3, 60.2, 31.2, 26.9, 18.9, 14.3. **HRMS (ESI)**: m/z: $[M+H]^+$ Calcd. for $C_{16}H_{18}ClO_2$: 277.0995, found: 277.0990.

Ethyl *(E)*–4–(8–ethoxy–8–oxooct–6–en–1–yn–1–yl)benzoate (1e)

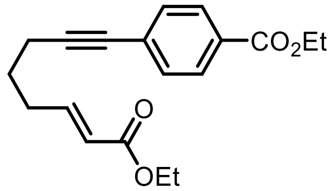

Prepared according to typical procedure **A**, after a flash column chromatography (petroleum ether/ethyl acetate = 10/1) afforded the product as yellow oil (482.1 mg, 75% yield).

^{1}H NMR (400 MHz, $CDCl_3$) δ 7.98 (d, J = 8.1 Hz, 2H), 7.45 (d, J = 8.1 Hz, 2H), 7.00 (dt, J = 15.6, 7.0 Hz, 1H), 5.90 (d, J = 15.6 Hz, 1H), 4.39 (q, J = 7.1 Hz, 2H), 4.21 (q, J = 7.1 Hz, 2H), 2.49 (t, J = 7.0 Hz, 2H), 2.41 (q, J = 7.3 Hz, 2H), 1.84–1.77 (m, 2H), 1.41 (t, J = 7.1 Hz, 3H), 1.30 (t, J = 7.1 Hz, 3H). **^{13}C NMR** (100 MHz, $CDCl_3$) δ 166.5, 166.1, 147.8, 131.4, 129.4, 128.4, 122.2, 92.5, 80.9, 61.0, 60.2, 31.2, 26.9, 19.0, 14.3, 14.3. **HRMS (EI)**: m/z: [M]$^+$ Calcd. for $C_{19}H_{22}O_4$: 314.1518, found: 314.1514.

Ethyl *(E)*–8–(3,5–dimethylphenyl)oct–2–en–7–ynoate (1f)

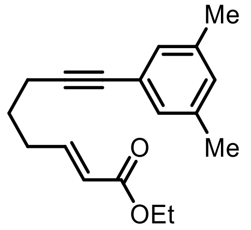

Prepared according to typical procedure **A**, after a flash column chromatography (petroleum ether/ethyl acetate = 20/1) afforded the product as yellow oil (469.0 mg, 79% yield).

^{1}H NMR (400 MHz, $CDCl_3$) δ 7.07 – 6.92 (m, 4H), 5.90 (dt, J = 15.6, 1.6 Hz, 1H), 4.21 (q, J = 7.1 Hz, 2H), 2.49 – 2.38 (m, 5H), 2.31 – 2.29 (m, 6H), 1.83 – 1.74 (m, 2H), 1.31 (t, J = 7.1 Hz, 3H). **^{13}C NMR** (100 MHz, $CDCl_3$) δ 166.6, 148.1, 137.8, 129.6, 129.3, 123.4, 122.0, 88.3, 81.6, 60.2, 31.2, 27.1, 21.1, 18.9, 14.3. **HRMS (EI)**: m/z: [M]$^+$ Calcd. for $C_{18}H_{22}O_2$: 270.1620, found: 270.1615.

Ethyl *(E)*–8–(3–(trifluoromethyl)phenyl)oct–2–en–7–ynoate (1g)

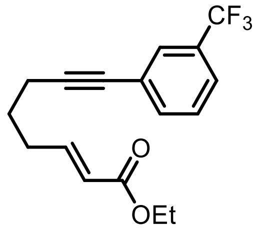

Prepared according to typical procedure **A**, after a flash column chromatography (petroleum ether/ethyl acetate = 20/1) afforded the product as yellow oil (496.2 mg, 75% yield).

^{1}H NMR (400 MHz, $CDCl_3$) δ 7.66 (d, J = 1.8 Hz, 1H), 7.59 – 7.52 (m, 2H), 7.46 –

7.40 (m, 1H), 7.01 (dt, J = 15.7, 7.0 Hz, 1H), 5.91 (dt, J = 15.6, 1.6 Hz, 1H), 4.21 (q, J = 7.1 Hz, 2H), 2.56 – 2.34 (m, 4H), 1.85–1.78 (m, 2H), 1.31 (t, J = 7.1 Hz, 3H). **^{13}C NMR** (100 MHz, $CDCl_3$) δ 166.5, 147.8, 134.7, 130.7, 128.7, 128.4(q, $^3J_{C–F}$ = 10.0 Hz), 124.7(q, $^2J_{C–F}$ = 50.0 Hz), 124.3, 124.2, 122.2, 91.0, 80.1, 60.2, 31.2, 26.9, 18.8, 14.3. **^{19}F NMR** (376 MHz, C_6D_6) δ –63.04. **HRMS (EI)**: m/z: [M] $^+$ Calcd. for $C_{17}H_{17}F_3O_2$: 310.1181, found: 310.1180.

Ethyl *(E)*–8–(3–fluorophenyl)oct–2–en–7–ynoate (1h)

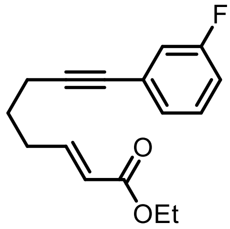

Prepared according to typical procedure **A**, after a flash column chromatography (petroleum ether/ethyl acetate = 20/1) afforded the product as yellow oil (318.5 mg, 63% yield).

^{1}H NMR (400 MHz, $CDCl_3$) δ 7.30 – 7.23 (m, 1H), 7.20 – 7.17 (m, 1H), 7.12–7.09 (m, 1H), 7.05 – 6.96 (m, 2H), 5.90 (d, J = 15.6 Hz, 1H), 4.21 (q, J = 7.1 Hz, 2H), 2.47 (t, J = 7.0 Hz, 2H), 2.43 – 2.38 (m, 2H), 1.84–1.76 (m, 2H), 1.31 (t, J = 7.1 Hz, 3H). **^{13}C NMR** (100 MHz, $CDCl_3$) δ 166.5, 163.4 (d, $^1J_{C–F}$ = 245.0 Hz), 147.8, 129.7 (d, $^3J_{C–F}$ = 9.0 Hz), 127.4 (d, $^4J_{C–F}$ = 3.0 Hz), 125.6 (d, $^3J_{C–F}$ = 10.0 Hz), 122.1, 118.3 (d, $^2J_{C–F}$ = 22.0 Hz), 115.0 (d, $^2J_{C–F}$ = 21.0 Hz), 90.3, 80.3(d, $^4J_{C–F}$ = 3.0 Hz), 60.2, 31.2, 26.9, 18.8, 14.3. **^{19}F NMR** (376 MHz, $CDCl_3$) δ –113.29. **HRMS (EI)**: m/z: [M] $^+$ Calcd. for $C_{16}H_{17}FO_2$: 260.1213, found: 260.1212.

Ethyl *(E)*–8–(3–chlorophenyl)oct–2–en–7–ynoate (1i)

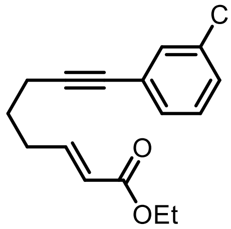

Prepared according to typical procedure **A**, after a flash column chromatography (petroleum ether/ethyl acetate = 20/1) afforded the product as yellow oil (401.6 mg, 73% yield).

^{1}H NMR (400 MHz, $CDCl_3$) δ 7.40 (t, *J* = 1.8 Hz, 1H), 7.30 – 7.21 (m, 3H), 7.00 (dt, *J* = 15.7, 7.0 Hz, 1H), 5.90 (dt, *J* = 15.6, 1.6 Hz, 1H), 4.21 (q, *J* = 7.1 Hz, 2H), 2.47 (t, *J* = 7.0 Hz, 2H), 2.43 – 2.37 (m, 2H), 1.83–1.76 (m, 2H), 1.31 (t, *J* = 7.1 Hz, 3H). **^{13}C NMR** (100 MHz, $CDCl_3$) δ 166.5, 147.8, 134.0, 131.5, 129.7, 129.4, 128.0, 125.5, 122.2, 90.6, 80.1, 60.2, 31.2, 26.9, 18.8, 14.3. **HRMS (ESI)**: m/z: $[M+H]^+$ Calcd. for $C_{16}H_{18}ClO_2$: 277.0995, found: 277.0990.

Ethyl *(E)*–8–(3–cyanophenyl)oct–2–en–7–ynoate (1j)

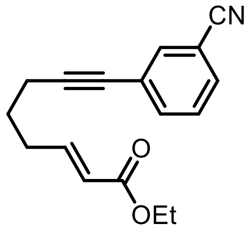

Prepared according to typical procedure **A**, after a flash column chromatography (petroleum ether/ethyl acetate = 10/1) afforded the product as yellow oil (438.8 mg, 80% yield).

^{1}H NMR (400 MHz, $CDCl_3$) δ 7.65 (d, *J* = 1.9 Hz, 1H), 7.60–7.53 (m, 2H), 7.39 (t, *J* = 7.8 Hz, 1H), 7.01–6.93 (m, 1H), 5.88 (d, *J* = 16 Hz, 1H), 4.19 (qd, *J* = 7.2, 1.5 Hz, 2H), 2.49 – 2.43 (m, 2H), 2.38 (q, *J* = 7.3 Hz, 2H), 1.82–1.75 (m, 2H), 1.28 (t, *J* = 7.2 Hz, 3H). **^{13}C NMR** (100 MHz, $CDCl_3$) δ 166.5, 147.7, 135.7, 134.9, 130.9, 129.1, 125.4, 122.2, 118.2, 112.7, 92.1, 79.3, 60.3, 31.2, 26.8, 18.8, 14.3. **HRMS (EI)**: m/z: $[M]^+$ Calcd. for $C_{17}H_{17}NO_2$: 267.1259, found: 267.1256.

Ethyl *(E)*–8–(o–tolyl)oct–2–en–7–ynoate (1k)

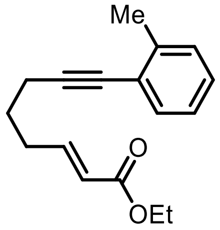

Prepared according to typical procedure **A**, after a flash column chromatography (petroleum ether/ethyl acetate = 20/1) afforded the product as yellow oil (450.9 mg, 88% yield).

^{1}H NMR (400 MHz, $CDCl_3$) δ 7.38 (d, *J* = 7.5 Hz, 1H), 7.20 (d, *J* = 4.3 Hz, 2H), 7.13 (dd, *J* = 8.0, 4.3 Hz, 1H), 7.01 (dt, *J* = 15.6, 6.9 Hz, 1H), 5.91 (dt, *J* = 15.7, 1.7 Hz, 1H),

4.21 (q, J = 7.1 Hz, 2H), 2.52 (t, J = 6.9 Hz, 2H), 2.47–2.41 (m, 5H), 1.85–1.78 (m, 2H), 1.31 (t, J = 7.1 Hz, 3H). **^{13}C NMR** (100 MHz, $CDCl_3$) δ 166.6, 148.1, 139.9, 131.8, 129.3, 127.7, 125.5, 123.6, 122.1, 93.1, 80.3, 60.2, 31.2, 27.2, 20.8, 19.0, 14.3. **HRMS (EI)**: m/z: $[M]^+$ Calcd. for $C_{17}H_{20}O_2$: 256.1463, found: 256.1459.

Ethyl *(E)*–8–(2–fluorophenyl)oct–2–en–7–ynoate (1l)

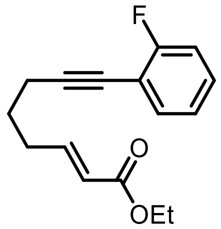

Prepared according to typical procedure **A**, after a flash column chromatography (petroleum ether/ethyl acetate = 20/1) afforded the product as yellow oil (368.4 mg, 72% yield).

^{1}H NMR (400 MHz, $CDCl_3$) δ 7.40–7.36 (m, 1H), 7.27–7.22 (m, 1H), 7.08–6.93 (m, 3H), 5.88 (dt, J = 15.6, 1.6 Hz, 1H), 4.18 (q, J = 7.1 Hz, 2H), 2.49 (t, J = 6.9 Hz, 2H), 2.43–2.37 (m, 1H), 1.83–1.75 (m, 2H), 1.28 (t, J = 7.1 Hz, 3H). **^{13}C NMR** (100 MHz, $CDCl_3$) δ 166.5, 162.8 (d, $^1J_{C–F}$ = 249 Hz), 147.9, 133.4 (d, $^4J_{C–F}$ = 2.0 Hz), 129.2 (d, $^3J_{C–F}$ = 8 Hz), 123.8 (d, $^4J_{C–F}$ = 4.0 Hz), 122.1, 115.3 (d, $^2J_{C–F}$ = 21.0 Hz), 112.2 (d, $^2J_{C–F}$ = 15.0 Hz), 94.6 (d, $^4J_{C–F}$ = 4.0 Hz), 74.7, 60.2, 31.0, 26.8, 19.0, 14.2. **^{19}F NMR** (376 MHz, $CDCl_3$) δ –110.97. **HRMS (EI)**: m/z: $[M]^+$ Calcd. for $C_{16}H_{17}FO_2$: 260.1213, found: 260.1208.

Ethyl *(E)*–8–(2–methoxyphenyl)oct–2–en–7–ynoate (1m)

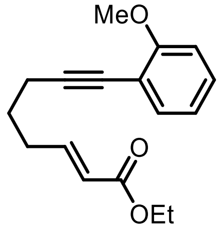

Prepared according to typical procedure **A**, after a flash column chromatography (petroleum ether/ethyl acetate = 20/1) afforded the product as yellow oil (393.7 mg, 72% yield).

^{1}H NMR (400 MHz, $CDCl_3$) δ 7.36 (dd, J = 7.5, 1.8 Hz, 1H), 7.27–7.23 (m, 1H), 6.99 (dt, J = 15.7, 7.0 Hz, 1H), 6.91 – 6.83 (m, 2H), 5.89 (dt, J = 15.6, 1.6 Hz, 1H), 4.19 (q,

J = 7.1 Hz, 2H), 3.88 (s, 3H), 2.51 (t, J = 6.9 Hz, 2H), 2.46 – 2.39 (m, 2H), 1.83–1.76 (m, 2H), 1.28 (t, J = 7.2 Hz, 3H). **^{13}C NMR** (100 MHz, $CDCl_3$) δ 166.6, 159.9, 148.2, 133.6, 129.1, 122.0, 120.4, 112.9, 110.5, 93.3, 77.6, 60.2, 55.8, 31.1, 27.1, 19.2, 14.3. **HRMS (EI)**: m/z: $[M]^+$ Calcd. for $C_{17}H_{20}O_3$: 272.1412, found: 272.1411.

Ethyl *(E)*–8–(2–acetylphenyl)oct–2–en–7–ynoate (1n)

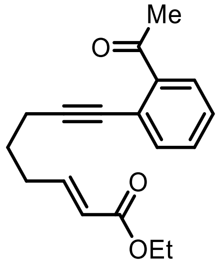

Prepared according to typical procedure **A**, after a flash column chromatography (petroleum ether/ethyl acetate = 20/1) afforded the product as yellow oil (336.7 mg, 59% yield).

^{1}H NMR (400 MHz, $CDCl_3$) δ 7.70–7.67 (m, 1H), 7.53 – 7.48 (m, 1H), 7.43 (td, J = 7.5, 1.5 Hz, 1H), 7.36 (td, J = 7.6, 1.5 Hz, 1H), 7.00 (dt, J = 15.7, 7.0 Hz, 1H), 5.90 (dt, J = 15.6, 1.6 Hz, 1H), 4.21 (q, J = 7.1 Hz, 2H), 2.72 (s, 3H), 2.53 (t, J = 7.0 Hz, 2H), 2.46–2.39 (m, 2H), 1.86–1.79 (m, 2H), 1.31 (t, J = 7.1 Hz, 3H). **^{13}C NMR** (100 MHz, $CDCl_3$) δ 200.7, 166.5, 147.8, 147.6, 141.0, 134.1, 131.1, 128.4, 127.7, 122.2, 95.3, 80.4, 60.2, 31.2, 29.9, 26.8, 19.2, 14.3. **HRMS (EI)**: m/z: $[M]^+$ Calcd. for $C_{18}H_{20}O_3$: 284.1412, found: 284.1408.

Ethyl *(E)*–8–(naphthalen–2–yl)oct–2–en–7–ynoate (1o)

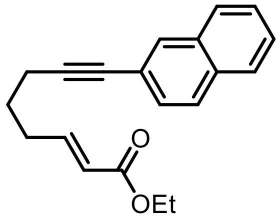

Prepared according to typical procedure **A**, after a flash column chromatography (petroleum ether/ethyl acetate = 20/1) afforded the product as yellow oil (419.0 mg, 72% yield).

^{1}H NMR (400 MHz, $CDCl_3$) δ 7.93 (s, 1H), 7.83 – 7.76 (m, 3H), 7.50–7.46 (m, 3H), 7.04 (dt, J = 15.7, 7.0 Hz, 1H), 5.93 (dt, J = 15.6, 1.6 Hz, 1H), 4.22 (q, J = 7.1 Hz, 2H), 2.53 (t, J = 7.0 Hz, 2H), 2.49 – 2.42 (m, 2H), 1.88–1.81 (m, 2H), 1.31 (t, J = 7.1 Hz,

3H). ^{13}C NMR (100 MHz, $CDCl_3$) δ 166.6, 148.1, 133.1, 132.5, 131.1, 128.7, 127.9, 127.7, 127.6, 126.4, 126.3, 122.1, 121.1, 89.5, 81.8, 60.2, 31.2, 27.1, 19.0, 14.3. **HRMS (EI)**: m/z: $[M]^+$ Calcd. for $C_{20}H_{20}O_2$: 292.1463, found: 292.1459.

Ethyl *(E)*–8–(1H–indol–5–yl)oct–2–en–7–ynoate (1p)

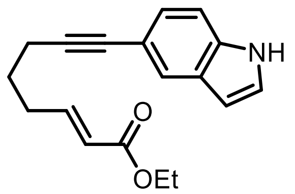

Prepared according to typical procedure **A**, after a flash column chromatography (petroleum ether/ethyl acetate = 5/1) afforded the product as yellow oil (292.1 mg, 50% yield).

^{1}H NMR (400 MHz, $CDCl_3$) δ 8.23 (s, 1H), 7.74 (s, 1H), 7.34–7.31 (m, 1H), 7.27 – 7.21 (m, 2H), 7.03 (dt, *J* = 15.6, 7.0 Hz, 1H), 6.55–6.53 (m, 1H), 5.92 (dt, *J* = 15.6, 1.6 Hz, 1H), 4.22 (q, *J* = 7.1 Hz, 2H), 2.52 – 2.41 (m, 4H), 1.85–1.78 (m, 2H), 1.31 (t, *J* = 7.1 Hz, 3H). **^{13}C NMR** (100 MHz, $CDCl_3$) δ 166.8, 148.5, 135.2, 127.8, 125.6, 125.0, 124.3, 122.0, 114.9, 111.0, 102.6, 86.3, 82.7, 60.3, 31.3, 27.3, 19.0, 14.3. **HRMS (EI)**: m/z: $[M]^+$ Calcd. for $C_{18}H_{19}NO_2$: 281.1416, found: 281.1412.

Ethyl *(E)*–8–(thiophen–2–yl)oct–2–en–7–ynoate (1q)

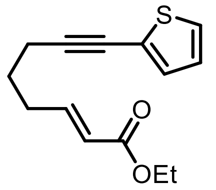

Prepared according to typical procedure **A**, after a flash column chromatography (petroleum ether/ethyl acetate = 20/1) afforded the product as yellow oil (417.8 mg, 86% yield).

^{1}H NMR (400 MHz, $CDCl_3$) δ 7.17 (dd, *J* = 5.2, 1.1 Hz, 1H), 7.12 (dd, *J* = 3.6, 1.1 Hz, 1H), 7.02 – 6.91 (m, 2H), 5.87 (dt, *J* = 15.6, 1.7 Hz, 1H), 4.19 (q, *J* = 7.1 Hz, 2H), 2.47 (t, *J* = 7.0 Hz, 2H), 2.37 (qd, *J* = 7.2, 1.6 Hz, 2H), 1.80–1.73 (m, 2H), 1.28 (t, *J* = 7.1 Hz, 3H). **^{13}C NMR** (100 MHz, $CDCl_3$) δ 166.6, 147.9, 131.1, 126.8, 126.1, 123.9, 122.1, 93.2, 74.5, 60.2, 31.2, 26.9, 19.1, 14.3. **HRMS (ESI)**: m/z: $[M+H]^+$ Calcd. for $C_{14}H_{17}O_2S$: 249.0949, found: 249.0944.

methyl *(E)*–8–phenyloct–2–en–7–ynoate (1r)

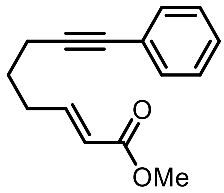

Prepared according to typical procedure **A**, after a flash column chromatography (petroleum ether/ethyl acetate = 20/1) afforded the product as yellow oil (1.14 g, 38% yield).

^{1}H NMR (400 MHz, $CDCl_3$) δ 7.41 (dd, J = 6.6, 3.0 Hz, 2H), 7.32–7.28 (m, 3H), 7.06–6.99 (m, 1H), 5.94–5.89 (m, 1H), 3.75 (s, 3H), 2.49 – 2.39 (m, 4H), 1.83–1.76 (m, 2H). **^{13}C NMR** (100 MHz, $CDCl_3$) δ 167.0, 148.4, 131.6, 128.2, 127.7, 123.8, 121.6, 89.1, 81.4, 51.4, 31.2, 27.0, 18.9. **HRMS (ESI)**: m/z: $[M+H]^+$ Calcd. for $C_{15}H_{17}O_2$: 229.1229, found: 229.1223.

***tert*–Butyl *(E)*–8–phenyloct–2–en–7–ynoate (1s)**

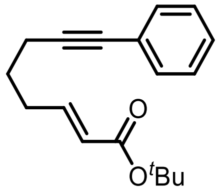

Prepared according to typical procedure **A**, after a flash column chromatography (petroleum ether/ethyl acetate = 20/1) afforded the product as yellow oil (1.57 g, 65% yield).

^{1}H NMR (400 MHz, $CDCl_3$) δ 7.41 (dd, J = 6.7, 3.0 Hz, 2H), 7.30 (dd, J = 5.1, 2.0 Hz, 3H), 6.90 (dt, J = 15.6, 6.9 Hz, 1H), 5.83 (d, J = 15.6 Hz, 1H), 2.47 (t, J = 7.0 Hz, 2H), 2.38 (qd, J = 7.1, 1.6 Hz, 2H), 1.79 (p, J = 7.2 Hz, 2H), 1.51 (s, 9H). ^{13}C NMR (100 MHz, $CDCl_3$) δ 166.0, 146.8, 131.6, 128.2, 127.6, 123.8, 123.8, 89.2, 81.3, 80.1, 31.1, 28.2, 27.1, 18.9. **HRMS (EI)**: m/z: $[M+Na]^+$ Calcd. for $C_{18}H_{22}O_2Na$: 293.1517, found: 293.1512.

Ethyl (*E*)-4-((3-phenylprop-2-yn-1-yl)oxy)but-2-enoate (1t)

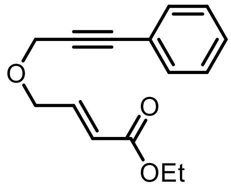

Prepared according to typical procedure **A**, after a flash column chromatography

(petroleum ether/ethyl acetate = 20/1) afforded the product as yellow oil (585.6 mg, 48% yield).

^{1}H NMR (400 MHz, $CDCl_3$) δ 7.52 – 7.41 (m, 2H), 7.41 – 7.30 (m, 3H), 7.01 (dt, *J* = 15.8, 4.5 Hz, 1H), 6.15 (dt, *J* = 15.7, 2.0 Hz, 1H), 4.45 (s, 2H), 4.33 (dd, *J* = 4.5, 2.0 Hz, 2H), 4.23 (q, *J* = 7.1 Hz, 2H), 1.31 (t, *J* = 7.2 Hz, 4H). **^{13}C NMR** (101 MHz, $CDCl_3$) δ 166.4, 143.7, 131.8, 128.6, 128.3 (two peaks overlap), 121.8, 86.9, 84.4, 68.2, 60.5, 58.7, 14.2.

Ethyl €–4–((N–(3–(4–methoxyphenyl)prop–2–yn–1–yl)–4–methylphenyl)sulfonamido)but–2–enoate (1u)

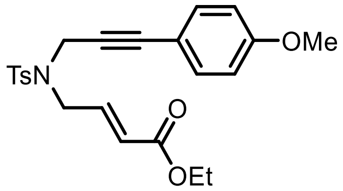

Prepared according to typical procedure **A**, after a flash column chromatography (petroleum ether/ethyl acetate = 5/1) afforded the product as yellow oil (289.3 mg, 34% yield).

^{1}H NMR (400 MHz, $CDCl_3$) δ 7.82 – 7.76 (m, 2H), 7.31 – 7.26 (m, 2H), 7.07 – 7.02 (m, 2H), 6.88 (dt, *J* = 15.6, 5.8 Hz, 1H), 6.81 – 6.76 (m, 2H), 6.09 (d, *J* = 15.6 Hz, 1H), 4.31 (s, 2H), 4.21 (q, *J* = 7.1 Hz, 2H), 4.06 (dd, *J* = 5.9, 1.7 Hz, 2H), 3.81 (s, 3H), 2.38 (s, 3H), 1.30 (t, *J* = 7.1 Hz, 3H). **^{13}C NMR** (100 MHz, $CDCl_3$) δ 165.7, 159.8, 143.8, 141.6, 135.8, 133.0, 129.7, 127.8, 124.5, 114.0, 113.8, 86.1, 79.8, 60.6, 55.3, 47.4, 37.7, 21.5, 14.2. **HRMS (ESI)**: m/z: $[M+Na]^+$ Calcd. For $C_{23}H_{25}NnaO_5S$: 450.1351, found: 450.1346.

Ethyl €–4–((N–(3–(3–cyanophenyl)prop–2–yn–1–yl)–4–methylphenyl)sulfonamido)but–2–enoate (1v)

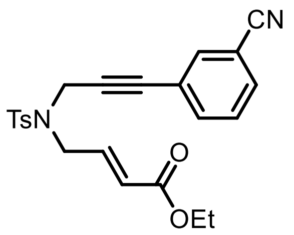

Prepared according to typical procedure **A**, after a flash column chromatography (petroleum ether/ethyl acetate = 5/1) afforded the product as yellow oil (745.4 mg, 88%

yield).

^{1}H NMR (400 MHz, $CDCl_3$) δ 7.79 (dd, *J* = 8.3, 2.2 Hz, 2H), 7.61 – 7.56 (m, 1H), 7.43 – 7.28 (m, 5H), 6.94 – 6.81 (m, 1H), 6.08 (dt, *J* = 15.7, 1.6 Hz, 1H), 4.35 – 4.31 (m, 2H), 4.27 – 4.18 (m, 2H), 4.06 (d, *J* = 5.8 Hz, 2H), 2.42 (s, 3H), 1.33 – 1.28 (m, 3H). **^{13}C NMR** (100 MHz, $CDCl_3$) δ 165.5, 144.1, 141.2, 135.8, 135.5, 134.9, 131.8, 129.8, 129.2, 127.9, 124.8, 123.5, 117.8, 112.8, 84.2, 83.7, 60.7, 47.7, 37.5, 21.5, 14.2. **HRMS (EI)**: m/z: $[M]^+$ Calcd. For $C_{23}H_{22}N_2O_4S$: 422.1300, found: 422.1295.

1– Ethyl 4,4–dimethyl €–7–(4–(ethoxycarbonyl)phenyl)hept–1–en–6–yne–1,4,4–tricarboxylate (1x)

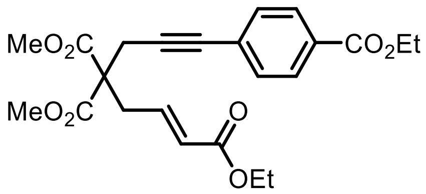

Prepared according to typical procedure **A**, after a flash column chromatography (petroleum ether/ethyl acetate = 10/1) afforded the product as yellow oil (668.5 mg, 80% yield).

^{1}H NMR (400 MHz, $CDCl_3$) δ 7.95 (d, *J* = 8.4 Hz, 2H), 7.41 (d, *J* = 8.4 Hz, 2H), 6.80 (dt, *J* = 15.5, 7.8 Hz, 1H), 5.95 (d, *J* = 15.5 Hz, 1H), 4.36 (q, *J* = 7.1 Hz, 2H), 4.17 (q, *J* = 7.1 Hz, 2H), 3.78 (s, 6H), 3.05 (s, 2H), 3.00 (dd, *J* = 7.8, 1.4 Hz, 2H), 1.38 (t, *J* = 7.1 Hz, 3H), 1.27 (t, *J* = 8.0 Hz, 3H). **^{13}C NMR** (100 MHz, $CDCl_3$) δ 169.7, 166.0, 165.8, 141.5, 131.6, 129.9, 129.4, 129.3, 127.5, 125.9, 86.8, 83.6, 61.1, 60.5, 56.9, 53.1, 35.3, 24.2, 14.3, 14.2. **HRMS (EI)**: m/z: $[M]^+$ Calcd. For $C_{23}H_{26}O_8$: 430.1628, found: 430.1623.

Ethyl (*Z*)-8-phenyloct-2-en-7-ynoate (1ab)

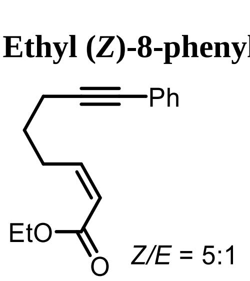

Prepared according to typical procedure **A** from the corresponding enyne[7], after a flash column chromatography (petroleum ether/ethyl acetate = 20/1) afforded the product as yellow oil (96.8 mg, 20% yield, *Z/E* = 5:1).

^{1}H NMR (400 MHz, $CDCl_3$) Z isomer: δ 7.46 – 7.38 (m, 2H), 7.29 (m, 3H), 6.28 (dt,

J = 11.5, 7.5 Hz, 1H), 5.83 (dt, J = 11.5, 1.7 Hz, 1H), 4.19 (q, J = 7.1 Hz, 2H), 2.84 (m, 2H), 2.48 (t, J = 7.2 Hz, 2H), 1.79 (m, 2H), 1.30 (t, J = 7.1 Hz, 3H). ^{13}**C NMR** (101 MHz, $CDCl_3$) Z isomer: δ 166.4, 149.0, 131.6, 128.2, 127.6, 123.9, 120.5, 89.6, 81.1, 59.9, 28.3, 28.2, 19.2, 14.3.

***(1S,2R,5R)*–2–*iso*–Propyl–5–methylcyclohexyl 4–(*€*–8–ethoxy–8–oxooct–6–en–1–yn–1–yl)benzoate (1y)**

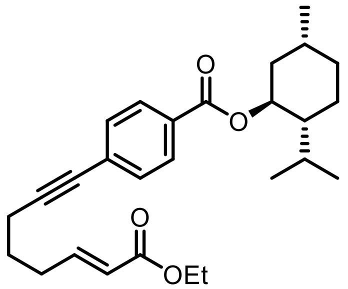

Prepared according to typical procedure **A**, after a flash column chromatography (petroleum ether/ethyl acetate = 20/1) afforded the product as yellow oil (1.35 g, 80% yield).

^{1}**H NMR** (400 MHz, $CDCl_3$) δ 8.01 – 7.94 (m, 2H), 7.45 (d, J = 8.0 Hz, 2H), 7.06 – 6.94 (m, 1H), 5.90 (dt, J = 15.6, 1.5 Hz, 1H), 4.94 (td, J = 10.8, 4.3 Hz, 1H), 4.21 (qt, J = 7.1, 1.2 Hz, 2H), 3.76 (d, J = 6.0 Hz, 2H), 2.50 (t, J = 7.0 Hz, 2H), 2.41 (q, J = 7.4 Hz, 2H), 2.14 (d, J = 12.3 Hz, 1H), 1.99 – 1.92 (m, 1H), 1.88 – 1.71 (m, 6H), 1.57 (s, 1H), 1.33 – 1.28 (m, 3H), 0.94 (t, J = 6.4 Hz, 7H), 0.81 (dt, J = 7.0, 1.2 Hz, 3H). ^{13}**C NMR** (100 MHz, $CDCl_3$) δ 166.5, 165.6, 147.8, 131.4, 129.8, 129.4, 128.3, 122.2, 92.4, 81.0, 75.0, 60.2, 47.3, 41.0, 34.3, 31.5, 31.2, 26.9, 26.5, 23.7, 22.0, 20.8, 19.0, 16.5, 14.3. **HRMS (ESI)**: m/z: $[M+Na]^+$ Calcd. For $C_{27}H_{36}NaO_4$: 447.2511, found: 447.2506.

(*(3aR,5R,5aS,8aS,8bR)*–2,2,7,7–Tetramethyltetrahydro–5H–bis([1,3]dioxolo)[4,5–b:4',5'–d]pyran–5–yl)methyl 4–(*€*–8–ethoxy–8–oxooct–6–en–1–yn–1–yl)benzoate (1z)

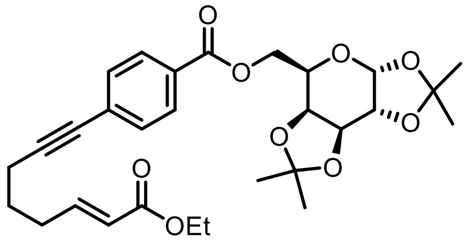

Prepared according to typical procedure **A**, after a flash column chromatography (petroleum ether/ethyl acetate = 10/1) afforded the product as yellow oil (1.20 g, 88%

yield).

^{1}H NMR (400 MHz, $CDCl_3$) δ 8.01 – 7.94 (m, 2H), 7.48 – 7.41 (m, 2H), 6.99 (dt, *J* = 15.7, 6.9 Hz, 1H), 5.89 (dt, *J* = 15.6, 1.6 Hz, 1H), 5.57 (d, *J* = 5.0 Hz, 1H), 4.66 (dd, *J* = 7.9, 2.5 Hz, 1H), 4.53 (dd, *J* = 11.5, 4.8 Hz, 1H), 4.42 (dd, *J* = 11.5, 7.5 Hz, 1H), 4.34 (ddd, *J* = 9.8, 6.4, 2.2 Hz, 2H), 4.23 – 4.15 (m, 3H), 2.48 (t, *J* = 7.0 Hz, 2H), 2.43 – 2.36 (m, 2H), 1.83–1.76 (m, 2H), 1.51 (s, 3H), 1.48 (s, 3H), 1.36 (s, 3H), 1.34 (s, 3H), 1.29 (t, *J* = 7.1 Hz, 3H). **^{13}C NMR** (100 MHz, $CDCl_3$) δ 166.5, 166.0, 147.8, 131.5, 129.6, 128.9, 128.6, 122.2, 109.7, 108.8, 96.3, 92.6, 80.9, 71.2, 70.8, 70.6, 66.2, 64.0, 60.2, 31.2, 26.9, 26.0, 26.0, 25.0, 24.5, 19.0, 14.3. **HRMS (ESI)**: m/z: $[M+Na]^+$ Calcd. For $C_{29}H_{36}NaO_9$: 551.2257, found: 551.2252.

Ethyl *(S,E)*–8–(4–((1–ethoxy–1–oxo–3–24henylpropane–2–yl)(tosyl)carbamoyl)phenyl)oct–2–en–7–ynoate (1aa)

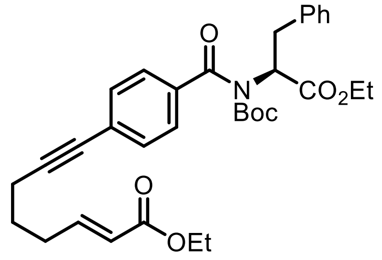

Prepared according to typical procedure **A**, after a flash column chromatography (petroleum ether/ethyl acetate = 20/1) afforded the product as yellow oil (1.43 g, 85% yield).

^{1}H NMR (400 MHz, $CDCl_3$) δ 7.31 – 7.22 (m, 7H), 7.07 (d, *J* = 8.1 Hz, 2H), 6.26 (dt, *J* = 11.5, 7.5 Hz, 1H), 5.87 – 5.81 (m, 1H), 5.43 (dd, *J* = 11.0, 5.7 Hz, 1H), 4.27 (qd, *J* = 7.1, 4.9 Hz, 2H), 4.21 – 4.14 (m, 2H), 3.67 – 3.37 (m, 2H), 2.83 (qd, *J* = 7.6, 1.7 Hz, 2H), 2.48 (t, *J* = 7.2 Hz, 2H), 2.33 (q, *J* = 6.4 Hz, 1H), 1.83 – 1.74 (m, 2H), 1.31 – 1.28 (m, 6H), 1.09 (s, 9H). **^{13}C NMR** (100 MHz, $CDCl_3$) δ 172.4, 170.1, 166.5, 152.6, 147.8, 137.4, 136.4, 130.9, 129.4, 128.5, 127.5, 126.7, 126.6, 122.2, 91.6, 83.5, 80.9, 61.6, 60.3, 58.7, 35.4, 31.2, 27.3, 26.9, 18.9, 14.3, 14.2. **HRMS (ESI)**: m/z: $[M+Na]^+$ Calcd. For $C_{33}H_{37}NnaO_7$: 584.2624, found: 584.2619.

3.3 General procedure B for the synthesis of 2

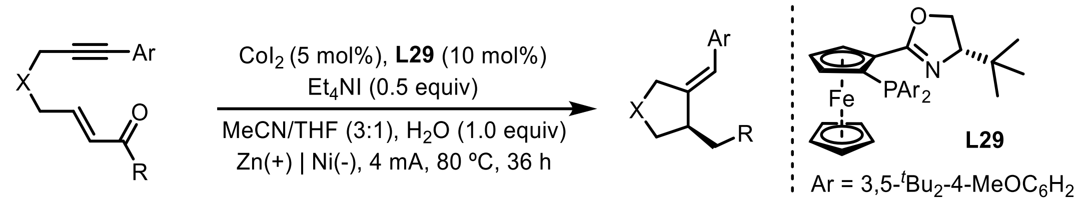

In Glove box, an oven–dried electrochemical cell with two stir bars was added enyne (0.4 mmol, 1 equiv), CoI_2 (0.02 mmol, 5 mol%), ligand (0.04 mmol, 10 mol%), Et_4NI (0.2 mmol, 0.5 equiv), H_2O (1.0 equiv), 3 mL of MeCN and 1 mL of THF. The tube was installed by a Ni foam (2.5 cm × 0.5 cm) as cathode and Zn flake (2.5 cm × 0.5 cm) as sacrificial anode. The distance of the electrodes is around 1 cm. The mixture was stirred at r.t. for 15 min. The reaction mixture was electrolyzed under a constant current of 4 mA at 80 °C until the complete consumption of the starting materials which was monitored by TLC (about 36 hours). The solvent was removed in vacuo, and the crude residue was purified via column chromatography to afford the desired product. The potential range over the reaction course is 0.3 V to 1.6 V.

Photographic guide for electrochemical reaction

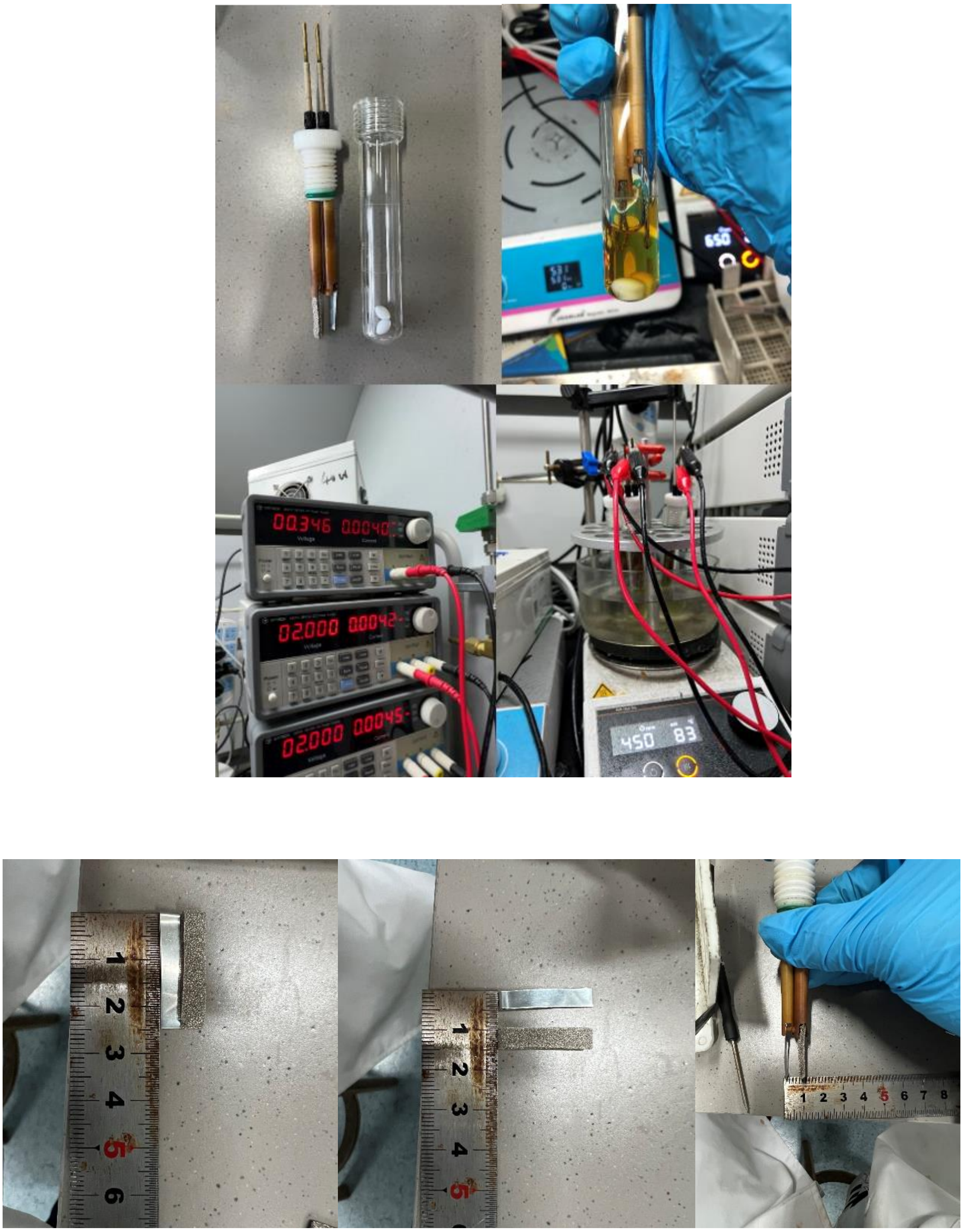

Supplementary Figure 1. Electrochemical apparatus

Ethyl *(R,E)*–2–(2–benzylidenecyclopentyl)acetate (2a)

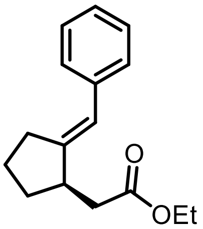

Prepared according to typical procedure **B**, after a flash column chromatography (petroleum ether/ethyl acetate = 20/1) afforded the product as colorless oil (60.8 mg, 59% yield) with 92% *ee*.

^{1}H NMR (400 MHz, $CDCl_3$) δ 7.37 – 7.32 (m, 4H), 7.21 (m, 1H), 6.31 (q, J = 2.4 Hz, 1H), 4.20 (q, J = 7.1 Hz, 2H), 3.12 – 3.01 (m, 1H), 2.75 – 2.57 (m, 3H), 2.40 (dd, J = 15.1, 9.2 Hz, 1H), 2.06–1.99 (m, 1H), 1.96 – 1.83 (m, 1H), 1.78–1.67 (m, 1H), 1.50 – 1.41 (m, 1H), 1.31 (t, J = 7.1 Hz, 3H). **^{13}C NMR** (100 MHz, $CDCl_3$) δ 172.9, 148.6, 138.5, 128.2 (two peaks overlap), 126.0, 121.3, 60.3, 42.7, 39.8, 32.1, 31.3, 24.7, 14.3. **HRMS (EI)**: m/z: [M] $^+$ Calcd. For $C_{16}H_{20}O_2$: 244.1463, found: 244.1460. **HPLC** analysis of the product: Daicel Chiralpak OD–H column; hexane/2–propanol = 98/02, 0.5 mL/min, 245 nm; Retention times: 12.90 min (minor), 13.33 min (major). $[\alpha]^{20}_D$ = 19.2 (c = 0.5, $CHCl_3$).

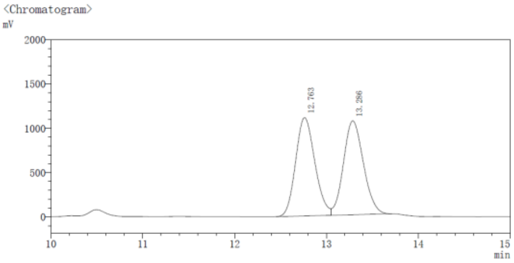

<Peak Table>
PDA Ch2 254nm

No.	Ret. Time(min)	Height	Height%	Area	Area%
1	12.763	1109215	51.129	16197180	50.274
2	13.286	1060222	48.871	16020846	49.726
Total		2169436	100.000	32218026	100.000

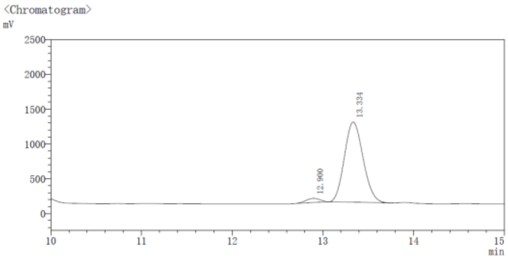

<Peak Table>
PDA Ch2 210nm

No.	Ret. Time(min)	Height	Height%	Area	Area%
1	12.900	59222	4.889	641183	3.740
2	13.334	1152113	95.111	16500807	96.260
Total		1211336	100.000	17141990	100.000

Ethyl *(R,E)*–2–(2–(4–(tert–butyl)benzylidene)cyclopentyl)acetate (2b)

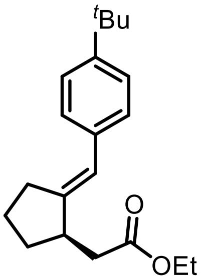

Prepared according to typical procedure **B**, after a flash column chromatography (petroleum ether/ethyl acetate = 20/1) afforded the product as colorless oil (72.1 mg,

60% yield) with 95% *ee*.

^{1}H NMR (400 MHz, $CDCl_3$) δ 7.40 – 7.36 (m, 2H), 7.30 – 7.26 (m, 2H), 6.28 (d, *J* = 2.8 Hz, 1H), 4.22–4.17 (m, 2H), 3.09–3.01 (m, 1H), 2.73 – 2.59 (m, 3H), 2.41–2.35 (m, 1H), 2.03–1.97 (m, 1H), 1.92–1.84 (m, 1H), 1.75–1.64 (m, 1H), 1.47 – 1.40 (m, 1H), 1.35 (s, 9H), 1.33 – 1.29 (m, 3H). **^{13}C NMR** (100 MHz, $CDCl_3$) δ 173.0, 148.9, 147.7, 135.6, 127.9, 125.1, 121.0, 60.3, 42.7, 39.8, 34.5, 32.1, 31.3 (two peaks overlap), 24.7, 14.3. **HRMS (EI)**: m/z: $[M]^+$ Calcd. For $C_{20}H_{28}O_2$: 300.2089, found:300.2085. **HPLC** analysis of the product: Daicel Chiralpak AD–H column; hexane/2–propanol = 98/02, 0.5 mL/min, 245 nm; Retention times: 12.81 min (major), 13.67 min (minor). $[\alpha]^{20}_D$ = 13.7 (*c* = 0.5, $CHCl_3$).

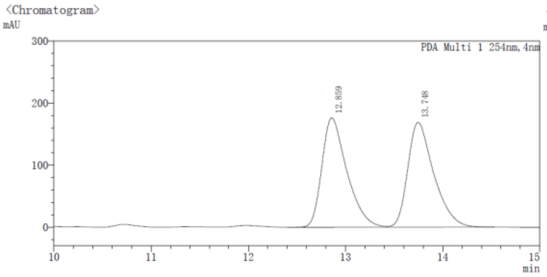

<Peak Table>
PDA Ch1 254nm

No.	Ret.Time(min)	Height	Height%	Area	Area%
1	12.859	176070	51.056	3147874	50.119
2	13.748	168787	48.944	3132973	49.881
Total		344857	100.000	6280847	100.000

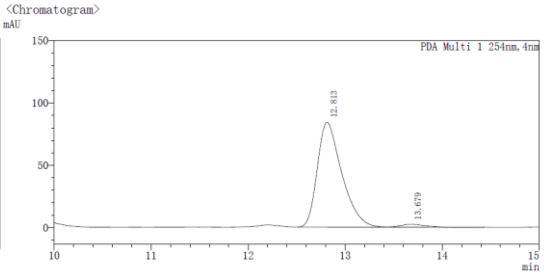

<Peak Table>
PDA Ch1 254nm

No.	Ret.Time(min)	Height	Height%	Area	Area%
1	12.813	84128	97.338	1471673	97.422
2	13.679	2301	2.662	38945	2.578
Total		86428	100.000	1510618	100.000

Ethyl *(R,E)*–2–(2–(4–fluorobenzylidene)cyclopentyl)acetate (2c)

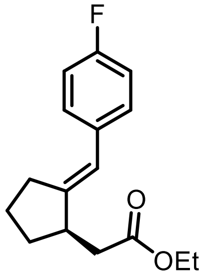

Prepared according to typical procedure **B**, after a flash column chromatography (petroleum ether/ethyl acetate = 20/1) afforded the product as colorless oil (87.8 mg, 84% yield) with 86% *ee*.

^{1}H NMR (400 MHz, $CDCl_3$) δ 7.30 – 7.25 (m, 2H), 7.06 – 6.99 (m, 2H), 6.26–6.24 (m, 1H), 4.19 (q, *J* = 7.2 Hz, 2H), 3.07–2.99 (m, 1H), 2.71 – 2.51 (m, 3H), 2.38 (dd, *J* = 15.1, 9.1 Hz, 1H), 2.04–1.98 (m, 1H), 1.95 – 1.82 (m, 1H), 1.77 – 1.66 (m, 1H), 1.47 – 1.39 (m, 1H), 1.30 (t, *J* = 7.1 Hz, 3H). **^{13}C NMR** (100 MHz, $CDCl_3$) δ 172.8, 161.1 (d, $^1J_{C\text{–}F}$ = 244.1 Hz), 148.1 (d, $^5J_{C\text{–}F}$ = 2.0 Hz), 134.5 (d, $^3J_{C\text{–}F}$ = 3.2 Hz), 129.6 (d, $^3J_{C\text{–}F}$ =

7.7 Hz), 120.1, 115.0 (d, $^2J_{C-F}$ = 21.1 Hz), 60.3, 42.6, 39.7, 32.1, 31.1, 24.6, 14.3. **^{19}F NMR** (376 MHz, $CDCl_3$) –116.37. **HRMS (EI)**: m/z: [M] $^+$ Calcd. For $C_{16}H_{19}FO_2$: 262.1369, found: 262.1365. **HPLC** analysis of the product: Daicel Chiralpak AD–H column; hexane/2–propanol = 98/02, 0.5 mL/min, 245 nm; Retention times: 19.25 min (minor), 20.20 min (major). $[\alpha]^{20}_D$ = 16.8 (*c* = 0.5, $CHCl_3$).

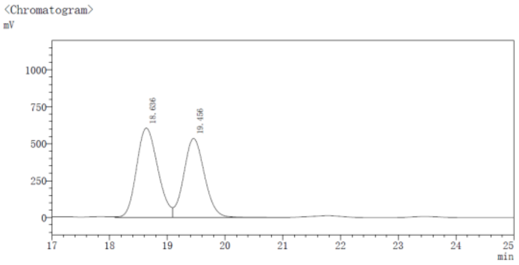

<Peak Table>
PDA Ch1 254nm

No.	Ret. Time(min)	Height	Height%	Area	Area%
1	18.636	605674	53.094	15486495	53.601
2	19.456	535073	46.906	13405870	46.399
Total		1140748	100.000	28892364	100.000

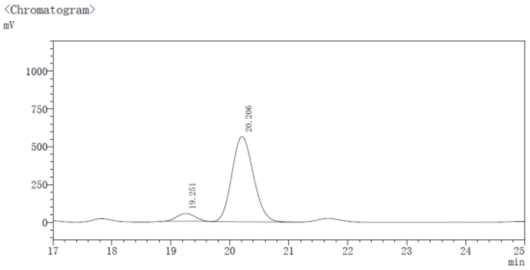

<Peak Table>
PDA Ch1 254nm

No.	Ret. Time(min)	Height	Height%	Area	Area%
1	19.251	50542	8.235	1094819	7.127
2	20.206	563223	91.765	14267069	92.873
Total		613765	100.000	15361887	100.000

Ethyl *(R,E)*–2–(2–(4–chlorobenzylidene)cyclopentyl)acetate (2d)

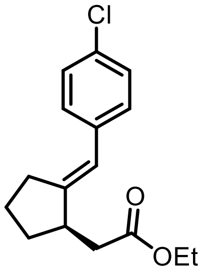

Prepared according to typical procedure **B**, after a flash column chromatography (petroleum ether/ethyl acetate = 20/1) afforded the product as colorless oil (19.5 mg, 17% yield) with 96% *ee*.

^{1}H NMR (400 MHz, $CDCl_3$) δ 7.30 (d, *J* = 8.8 Hz, 2H), 7.24 (d, *J* = 8.6 Hz, 2H), 6.24 (d, *J* = 2.5 Hz, 1H), 4.19 (q, *J* = 7.1 Hz, 2H), 3.07–2.99 (m, 1H), 2.71 – 2.54 (m, 3H), 2.38 (dd, *J* = 15.1, 9.1 Hz, 1H), 2.05–1.97 (m, 1H), 1.92–1.86 (m, 1H), 1.75 – 1.68 (m, 1H), 1.48–1.41 (m, 1H), 1.29 (t, *J* = 6.5 Hz, 3H). **^{13}C NMR** (100 MHz, $CDCl_3$) δ 172.8, 149.4, 136.9, 129.4, 128.3, 120.2, 60.4, 42.7, 39.6, 32.1, 31.3, 24.7, 14.3. **HRMS (EI)**: m/z: [M] $^+$ Calcd. For $C_{16}H_{19}ClO_2$: 278.1074, found: 278.1069. **HPLC** analysis of the product: Daicel Chiralpak OD–H column; hexane/2–propanol = 98/02, 0.5 mL/min, 245 nm; Retention times: 10.35 min (major), 11.37 min (minor). $[\alpha]^{20}_D$ = 10.3 (*c* = 0.5, $CHCl_3$).

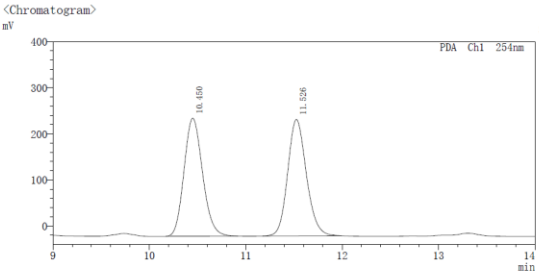

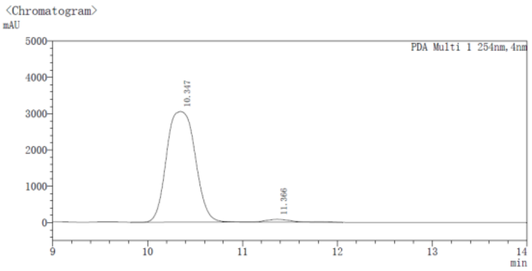

<Peak Table>
PDA Ch1 254nm

No.	Ret.Time(min)	Height	Height%	Area	Area%
1	10.450	256351	50.343	3432435	50.172
2	11.526	252862	49.657	3408874	49.828
Total		509213	100.000	6841309	100.000

<Peak Table>
PDA Ch1 254nm

No.	Ret.Time(min)	Height	Height%	Area	Area%
1	10.347	3053110	97.504	64212900	97.616
2	11.366	78148	2.496	1568475	2.384
Total		3131258	100.000	65781375	100.000

Ethyl *(R,E)*–4–((2–(2–ethoxy–2–oxoethyl)cyclopentylidene)methyl)benzoate (2e)

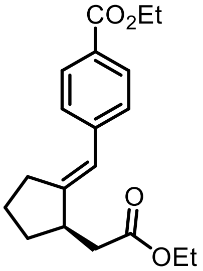

Prepared according to typical procedure **B**, after a flash column chromatography (petroleum ether/ethyl acetate = 10/1) afforded the product as colorless oil (107.1 mg, 85% yield) with 99% *ee*.

^{1}H NMR (400 MHz, $CDCl_3$) δ 7.98 (dd, *J* = 8.3, 1.6 Hz, 2H), 7.37 – 7.32 (m, 2H), 6.31 (s, 1H), 4.40 – 4.34 (m, 2H), 4.20–4.14 (m, 2H), 3.08–3.00 (m, 1H), 2.73 – 2.54 (m, 3H), 2.41–2.35 (m, 1H), 2.00 (dq, *J* = 12.4, 6.4 Hz, 1H), 1.93–1.84 (m, 1H), 1.76 – 1.69 (m, 1H), 1.48–1.37 (m, 4H), 1.27 (t, *J* = 8.0 Hz 3H). **^{13}C NMR** (100 MHz, $CDCl_3$) δ 172.7, 166.6, 151.7, 142.9, 129.5, 128.0 (two peaks overlap), 120.8, 60.8, 60.4, 43.0, 39.5, 32.0, 31.6, 24.7, 14.4, 14.3. **HRMS (EI)**: m/z: $[M]^+$ Calcd. For $C_{19}H_{24}O_4$: 316.1675, found: 316.1670. **HPLC** analysis of the product: Daicel Chiralpak AD–H column; hexane/2–propanol = 98/02, 0.5 mL/min, 245 nm; Retention times: 26.95 min (minor), 27.75 min (major). $[\alpha]^{20}_D$ = 13.2 (*c* = 0.5, $CHCl_3$).

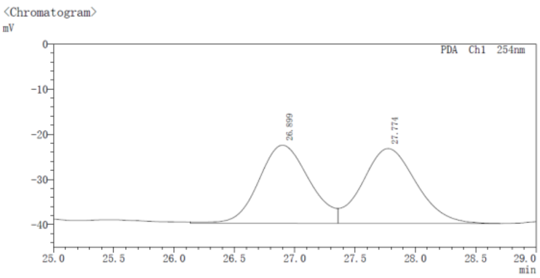

<Peak Table>
PDA Ch1 254nm

No.	Ret.Time(min)	Height	Height%	Area	Area%
1	26.899	17302	51.101	505194	49.733
2	27.774	16557	48.899	510623	50.267
Total		33859	100.000	1015818	100.000

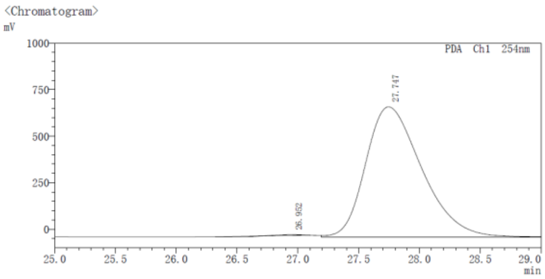

<Peak Table>
PDA Ch1 254nm

No.	Ret.Time(min)	Height	Height%	Area	Area%
1	26.952	4664	0.663	41710	0.186
2	27.747	699244	99.337	22382788	99.814
Total		703908	100.000	22424498	100.000

Ethyl *(R,E)*–2–(2–(3,5–dimethylbenzylidene)cyclopentyl)acetate (2f)

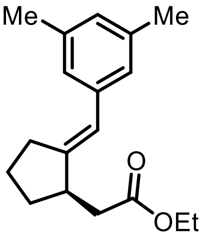

Prepared according to typical procedure **B**, after a flash column chromatography (petroleum ether/ethyl acetate = 20/1) afforded the product as colorless oil (60.4 mg, 56% yield) with 94% *ee*.

^{1}H NMR (400 MHz, $CDCl_3$) δ 6.97 (s, 2H), 6.87 (s, 1H), 6.25 (s, 1H), 4.24–4.18 (m, 2H), 3.08–3.01 (m, 1H), 2.74 – 2.58 (m, 3H), 2.42–2.38 (m, 1H), 2.35 (s, 6H), 2.06–1.98 (m, 1H), 1.94–1.84 (m, 1H), 1.75–1.68 (m, 1H), 1.51 – 1.40 (m, 1H), 1.34 – 1.29 (m, 3H). **^{13}C NMR** (100 MHz, $CDCl_3$) δ 173.0, 148.1, 138.4, 137.6, 127.8, 126.1, 121.4, 60.3, 42.7, 39.8, 32.1, 31.4, 24.7, 21.4, 14.3. **HRMS (EI)**: m/z: $[M]^+$ Calcd. For $C_{18}H_{24}O_2$: 272.1776, found: 272.1772. **HPLC** analysis of the product: Daicel Chiralpak OD–H column; hexane/2–propanol = 98/02, 0.5 mL/min, 245 nm; Retention times: 9.76 min (minor), 10.50 min (major). $[\alpha]^{20}_D$ = 22.6 (*c* = 0.5, $CHCl_3$).

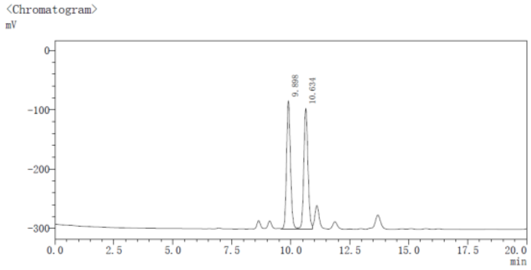

<Peak Table>
PDA Ch1 254nm

No.	Ret.Time(min)	Height	Height%	Area	Area%
1	9.898	215501	51.490	2485840	50.093
2	10.634	203031	48.510	2476640	49.907
Total		418533	100.000	4962480	100.000

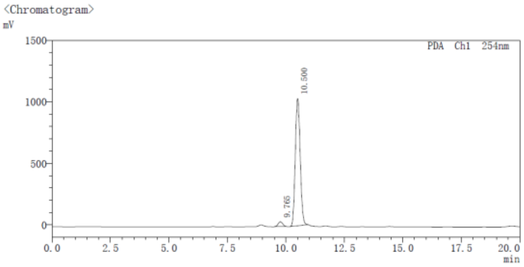

<Peak Table>
PDA Ch1 254nm

No.	Ret.Time(min)	Height	Height%	Area	Area%
1	9.765	38441	3.583	514593	3.288
2	10.500	1034396	96.417	15136327	96.712
Total		1072836	100.000	15650921	100.000

Ethyl *(R,E)*–2–(2–(3–(trifluoromethyl)benzylidene)cyclopentyl)acetate (2g)

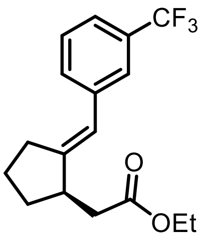

Prepared according to typical procedure **B**, after a flash column chromatography (petroleum ether/ethyl acetate = 20/1) afforded the product as colorless oil (110.4 mg, 88% yield) with 98% *ee*.

^{1}H NMR (400 MHz, $CDCl_3$) δ 7.53 (s, 1H), 7.46 – 7.40 (m, 3H), 6.30 (d, J = 2.5 Hz, 1H), 4.17 (q, J = 7.1 Hz, 2H), 3.08 – 3 .00 (m, 1H), 2.71 – 2.52 (m, 3H), 2.38 (dd, J = 15.2, 9.0 Hz, 1H), 2.06 – 1.95 (m, 1H), 1.93–1.84 (m, 1H), 1.75 – 1.66 (m, 1H), 1.48 – 1.39 (m, 1H), 1.28 (t, J = 7.1 Hz, 3H). **^{13}C NMR** (100 MHz, $CDCl_3$) δ 172.7, 150.8, 139.1, 131.2, 129.9 (q, $^1J_{C–F}$ = 267.9 Hz), 124.7 (d, $^3J_{C–F}$ = 4.0 Hz), 122.5 (q, $^3J_{C–F}$ = 3.0 Hz), 120.1, 60.3, 42.7, 39.5, 32.0, 31.3, 24.6, 14.2. **^{19}F NMR** (376 MHz, $CDCl_3$) δ –62.73. **HRMS (EI)**: m/z: [M] $^+$ Calcd. For $C_{17}H_{19}F_3O_2$ $[M]^+$: 312.1337, found: 312.1333. **HPLC** analysis of the product: Daicel Chiralpak AS–H column; hexane/2–propanol = 98/02, 0.5 mL/min, 245 nm; Retention times: 9.74 min (major), 10.86 min (minor). $[\alpha]^{20}_D$ = –5.0 (c = 0.5, $CHCl_3$).

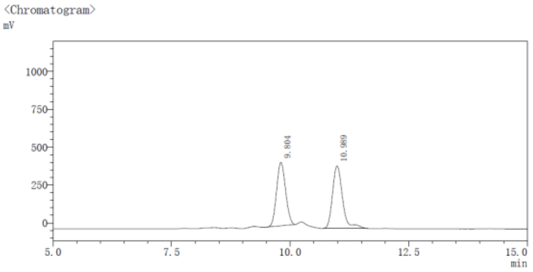

<Peak Table>
PDA Ch1 254nm

No.	Ret. Time(min)	Height	Height%	Area	Area%
1	9.804	419038	50.366	5532786	47.819
2	10.989	412949	49.634	6037483	52.181
Total		831987	100.000	11570269	100.000

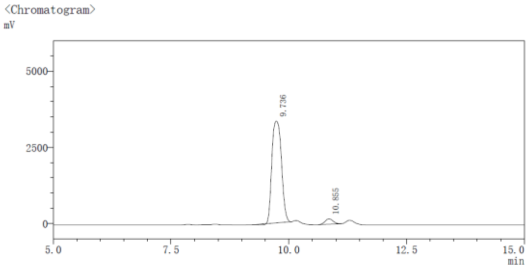

<Peak Table>
PDA Ch1 254nm

No.	Ret. Time(min)	Height	Height%	Area	Area%
1	9.736	3338816	94.956	46988084	95.800
2	10.855	177370	5.044	2060244	4.200
Total		3516186	100.000	49048328	100.000

Ethyl *(R,E)*–2–(2–(3–fluorobenzylidene)cyclopentyl)acetate (2h)

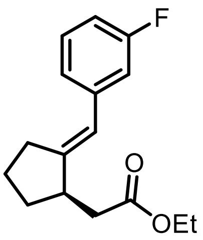

Prepared according to typical procedure **B**, after a flash column chromatography

(petroleum ether/ethyl acetate = 20/1) afforded the product as colorless oil (88.7 mg, 85% yield) with 82% *ee*.

^{1}H NMR (400 MHz, $CDCl_3$) δ 7.28 – 7.23 (m, 1H), 7.06 – 6.97 (m, 2H), 6.89–6.84 (m, 1H), 6.24 (q, J = 2.5 Hz, 1H), 4.17 (q, J = 7.1 Hz, 2H), 3.08 – 2.96 (m, 1H), 2.69 – 2.52 (m, 3H), 2.36 (dd, J = 15.2, 9.1 Hz, 1H), 2.03–1.95 (m, 1H), 1.92–1.82 (m, 1H), 1.75–1.65 (m, 1H), 1.46 – 1.39 (m, 1H), 1.27 (t, J = 7.1 Hz, 3H). **^{13}C NMR** (100 MHz, $CDCl_3$) δ 172.7, 162.8(d, $^1J_{C–F}$ = 243 Hz), 150.2, 140.6(d, $^3J_{C–F}$ = 7 Hz), 129.5(d, $^3J_{C–F}$ = 8 Hz), 124.0(d, $^4J_{C–F}$ = 3 Hz), 120.4(d, $^4J_{C–F}$ = 3 Hz), 114.6(d, $^2J_{C–F}$ = 22 Hz), 112.7(d, $^2J_{C–F}$ = 21 Hz), 60.3, 42.7, 39.5, 32.0, 31.4, 24.6, 14.3. **^{19}F NMR** (376 MHz, $CDCl_3$) δ –113.80. **HRMS (ESI)**: m/z: $[M+H]^+$ Calcd. For $C_{16}H_{19}FO_2$: 263.1442, found: 263.1441. **HPLC** analysis of the product: Daicel Chiralpak AS–H column; hexane/2–propanol = 99/01, 0.3 mL/min, 245 nm; Retention times: 20.18 min (major), 23.26 min (minor). $[\alpha]^{20}_D$ = 2.6 (c = 0.25, $CHCl_3$).

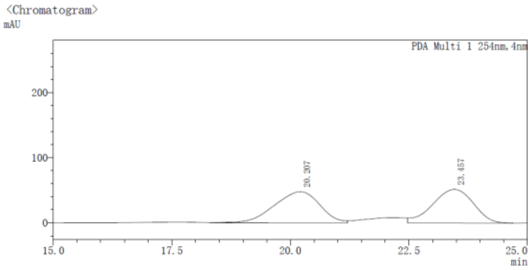

<Peak Table>
PDA Ch1 254nm

No.	Ret. Time (min)	Height	Height%	Area	Area%
1	20.207	47839	48.149	3199835	51.003
2	23.457	51518	51.851	3073925	48.997
Total		99357	100.000	6273759	100.000

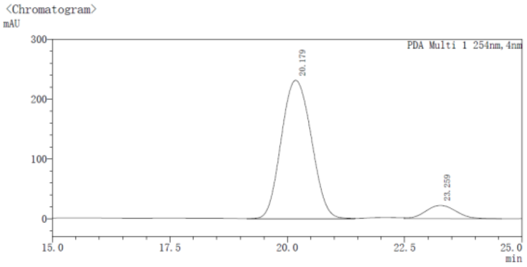

<Peak Table>
PDA Ch1 254nm

No.	Ret. Time (min)	Height	Height%	Area	Area%
1	20.179	231224	91.268	10284167	91.221
2	23.259	22123	8.732	989773	8.779
Total		253347	100.000	11273939	100.000

Ethyl *(R,E)*–2–(2–(3–chlorobenzylidene)cyclopentyl)acetate (2i)

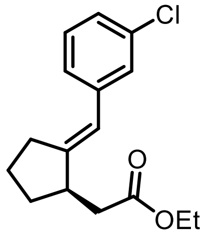

Prepared according to typical procedure **B**, after a flash column chromatography (petroleum ether/ethyl acetate = 20/1) afforded the product as colorless oil (92.0 mg, 83% yield) with 97% *ee*.

^{1}H NMR (400 MHz, $CDCl_3$) δ 7.30 – 7.27 (m, 1H), 7.26 – 7.21 (m, 1H), 7.16–7.13 (m, 2H), 6.20 (d, J = 2.5 Hz, 1H), 4.17 (q, J = 7.1 Hz, 2H), 3.02 (t, J = 7.2 Hz, 1H), 2.69 – 2.53 (m, 3H), 2.36 (dd, J = 15.2, 9.1 Hz, 1H), 2.02–1.96 (m, 1H), 1.91 – 1.82 (m, 1H),

1.75–1.65 (m, 1H), 1.45 – 1.39 (m, 1H), 1.27 (t, J = 7.1 Hz, 3H). **^{13}C NMR** (100 MHz, $CDCl_3$) δ 172.8, 150.4, 140.2, 134.1, 129.4, 128.0, 126.4, 126.0, 120.2, 60.4, 42.7, 39.6, 32.0, 31.4, 24.6, 14.3. **HRMS (EI)**: m/z: $[M]^+$ Calcd. For $C_{16}H_{19}ClO_2$: 278.1074, found: 278.1067. **HPLC** analysis of the product: Daicel Chiralpak OD–H column; hexane/2–propanol = 98/02, 0.5 mL/min, 245 nm; Retention times: 22.90 min (minor), 23.96 min (major). $[\alpha]^{20}_D$ = 17.8 (c = 0.5, $CHCl_3$).

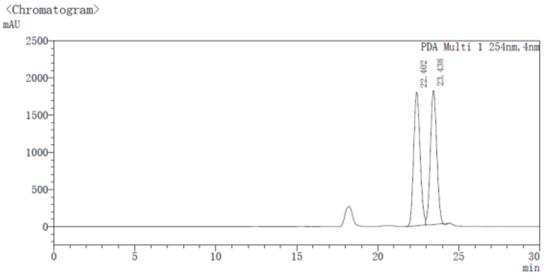

<Peak Table>
PDA Ch1 254nm

No.	Ret. Time (min)	Height	Height%	Area	Area%
1	22.402	1796478	49.933	49816147	50.404
2	23.438	1801266	50.067	49017213	49.596
Total		3597744	100.000	98833360	100.000

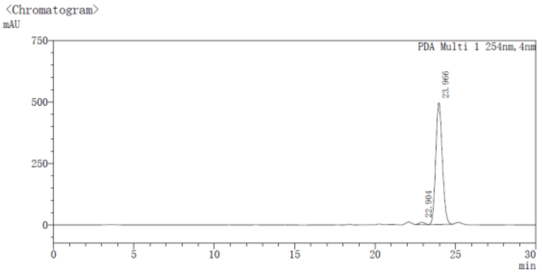

<Peak Table>
PDA Ch1 254nm

No.	Ret. Time (min)	Height	Height%	Area	Area%
1	22.904	9248	1.835	213458	1.490
2	23.966	494820	98.165	14113990	98.510
Total		504068	100.000	14327447	100.000

Ethyl *(R,E)*–2–(2–(3–cyanobenzylidene)cyclopentyl)acetate (2j)

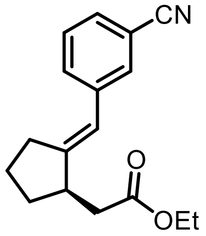

Prepared according to typical procedure **B**, after a flash column chromatography (petroleum ether/ethyl acetate = 20/1) afforded the product as colorless oil (68.7 mg, 64% yield) with 99% *ee*.

^{1}H NMR (400 MHz, $CDCl_3$) δ 7.56 (s, 1H), 7.51 – 7.37 (m, 3H), 6.24 (d, J = 2.5 Hz, 1H), 4.17 (q, J = 7.2 Hz, 2H), 3.07–3.00 (m, 1H), 2.69 – 2.53 (m, 3H), 2.38 (dd, J = 15.2, 8.9 Hz, 1H), 2.05 – 1.97 (m, 1H), 1.93–1.84 (m, 1H), 1.77 – 1.67 (m, 1H), 1.48–1.39 (m, 1H), 1.28 (t, J = 7.1 Hz, 3H). **^{13}C NMR** (100 MHz, $CDCl_3$) δ 172.6, 152.0, 139.6, 132.5, 131.4, 129.4, 129.0, 119.4, 119.1, 112.4, 60.4, 42.8, 39.5, 32.0, 31.5, 24.6, 14.3. **HRMS (EI)**: m/z: $[M]^+$ Calcd. For $C_{17}H_{19}NO_2$: 269.1416, found: 269.1405. **HPLC** analysis of the product: Daicel Chiralpak AD–H column; hexane/2–propanol = 90/10, 0.5 mL/min, 245 nm; Retention times: 14.53 min (minor), 15.10 min (major). $[\alpha]^{20}_D$ = 13.5 (c = 0.5, $CHCl_3$).

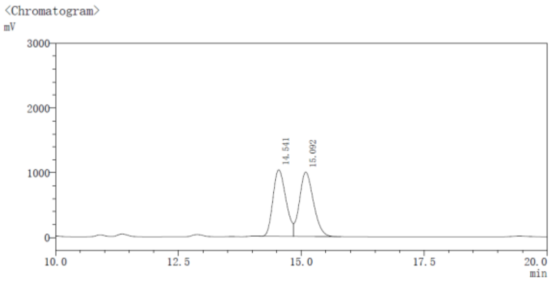

<Peak Table>

PDA Ch1 254nm

No.	Ret. Time (min)	Height	Height%	Area	Area%
1	14.541	1025478	50.801	18812744	48.889
2	15.092	993140	49.199	19667832	51.111
Total		2018618	100.000	38480575	100.000

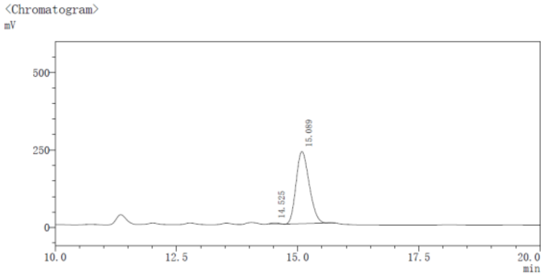

<Peak Table>

PDA Ch1 254nm

No.	Ret. Time (min)	Height	Height%	Area	Area%
1	14.525	2397	1.019	22975	0.518
2	15.089	232822	98.981	4413113	99.482
Total		235219	100.000	4436088	100.000

Ethyl *(R,E)*–2–(2–(2–methylbenzylidene)cyclopentyl)acetate (2k)

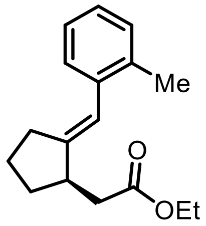

Prepared according to typical procedure **B**, after a flash column chromatography (petroleum ether/ethyl acetate = 20/1) afforded the product as colorless oil (35.0 mg, 34% yield) with 80% *ee*.

^{1}H NMR (400 MHz, $CDCl_3$) δ 7.26 – 7.23 (m, 1H), 7.16 – 7.08 (m, 4H), 6.31 (d, J = 2.5 Hz, 1H), 4.17 (q, J = 7.2 Hz, 2H), 3.07–2.99 (m, 1H), 2.67 (dd, J = 15.1, 5.6 Hz, 1H), 2.47 – 2.35 (m, 3H), 2.26 (s, 3H), 2.04–1.97 (m, 1H), 1.81 – 1.73 (m, 1H), 1.67 – 1.54 (m, 2H), 1.45 – 1.39 (m, 1H), 1.27 (t, J = 6.0 Hz, 3H). **^{13}C NMR** (100 MHz, $CDCl_3$) δ 173.0, 148.6, 137.5, 135.9, 129.8, 128.2, 126.3, 125.4, 119.4, 60.3, 41.8, 40.0, 32.3, 30.7, 24.5, 20.0, 14.3. **HRMS (EI)**: m/z: $[M]^+$ Calcd. For $C_{17}H_{22}O_2$: 258.1620, found: 258.1618. **HPLC** analysis of the product: Daicel Chiralpak OD–H column; hexane/2–propanol = 98/02, 0.5 mL/min, 245 nm; Retention times: 9.73 min (minor), 10.88 min (major). $[\alpha]^{20}_D$ = 1.6 (c = 0.5, $CHCl_3$).

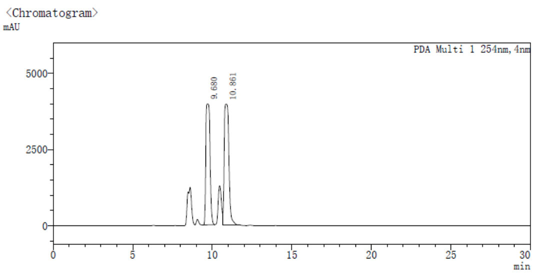

<Peak Table>

PDA Ch1 254nm

No.	Ret. Time (min)	Height	Height%	Area	Area%
1	9.680	3971910	50.059	72476440	47.376
2	10.861	3962550	49.941	80503475	52.624
Total		7934461	100.000	152979915	100.000

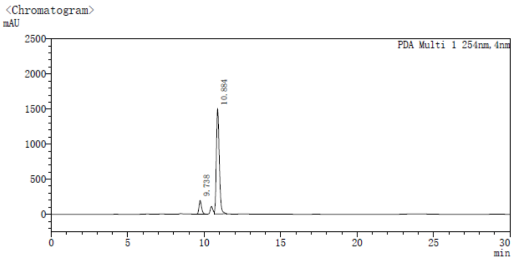

<Peak Table>

PDA Ch1 254nm

No.	Ret. Time (min)	Height	Height%	Area	Area%
1	9.738	194103	11.492	2308199	10.323
2	10.884	1494868	88.508	20051981	89.677
Total		1688971	100.000	22360181	100.000

Ethyl *(R,E)*–2–(2–(2–fluorobenzylidene)cyclopentyl)acetate (2l)

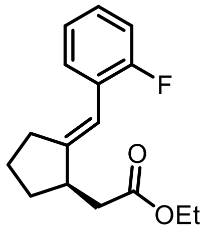

Prepared according to typical procedure **B**, after a flash column chromatography (petroleum ether/ethyl acetate = 20/1) afforded the product as colorless oil (90.8 mg, 87% yield) with 96% *ee*.

^{1}H NMR (400 MHz, $CDCl_3$) δ 7.36 (t, J = 7.8 Hz, 1H), 7.18–7.13 (m, 1H), 7.12 – 6.98 (m, 2H), 6.39 (s, 1H), 4.20–4.14 (m, 2H), 3.08–3.00 (m, 1H), 2.71–2.66 (m, 1H), 2.58–2.49 (m, 2H), 2.41–2.35 (m, 1H), 2.05–1.98 (m, 1H), 1.91 – 1.77 (m, 1H), 1.70–1.65 (m, 1H), 1.45–1.40 (m, 1H), 1.30 – 1.26 (m, 3H). **^{13}C NMR** (100 MHz, $CDCl_3$) δ 172.8, 159.9 (d, $^1J_{C–F}$ = 247.4 Hz), 150.7, 129.2 (d, $^4J_{C–F}$ = 3.4 Hz), 127.6 (d, $^3J_{C–F}$ = 8.3 Hz), 126.1 (d, $^2J_{C–F}$ = 13.0 Hz), 123.5 (d, $^4J_{C–F}$ = 3.7 Hz), 115.1 (d, $^2J_{C–F}$ = 22.5 Hz), 113.0 (d, $^3J_{C–F}$ = 5.1 Hz), 60.3, 42.4, 39.7, 32.1, 31.2, 24.5, 14.2. **^{19}F NMR** (376 MHz, $CDCl_3$) δ –116.72. **HRMS (EI)**: m/z: $[M]^+$ Calcd. For $C_{16}H_{19}FO_2$: 262.1369, found: 262.1362. **HPLC** analysis of the product: Daicel Chiralpak OD–H column; hexane/2–propanol = 98/02, 0.5 mL/min, 245 nm; Retention times: 10.13 min (minor), 11.25 min (major). $[\alpha]^{20}_D$ = –1.5 (c = 0.5, $CHCl_3$).

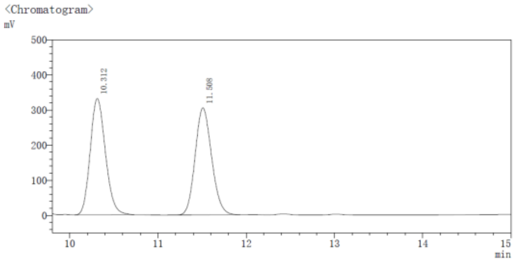

<Peak Table>
PDA Ch2 254nm

No.	Ret.Time(min)	Height	Height%	Area	Area%
1	10.312	330919	52.050	3961361	49.995
2	11.508	304855	47.950	3962194	50.005
Total		635774	100.000	7923555	100.000

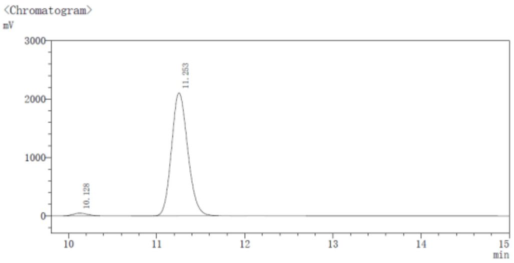

<Peak Table>
PDA Ch2 254nm

No.	Ret.Time(min)	Height	Height%	Area	Area%
1	10.128	45025	2.095	498714	1.818
2	11.253	2104633	97.905	26934668	98.182
Total		2149658	100.000	27433382	100.000

Ethyl *(R,E)*–2–(2–(2–methoxybenzylidene)cyclopentyl)acetate (2m)

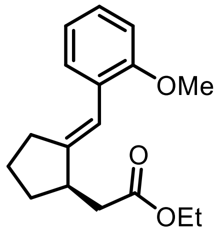

Prepared according to typical procedure **B**, after a flash column chromatography (petroleum ether/ethyl acetate = 20/1) afforded the product as colorless oil (85.4 mg, 78% yield) with 84% *ee*.

^{1}H NMR (400 MHz, $CDCl_3$) δ 7.32 (dd, J = 7.6, 1.7 Hz, 1H), 7.19–7.15 (m, 1H), 6.94–6.84 (m, 1H), 6.85 (dd, J = 8.2, 1.1 Hz, 1H), 6.51 (d, J = 2.5 Hz, 1H), 4.16 (q, J = 7.1 Hz, 2H), 3.82 (s, 3H), 3.04 (t, J = 7.6 Hz, 1H), 2.71 (dd, J = 15.1, 5.2 Hz, 1H), 2.59–2.53 (m, 2H), 2.37 (dd, J = 15.1, 9.5 Hz, 1H), 2.04 – 1.97 (m, 1H), 1.86 – 1.80 (m, 1H), 1.68 – 1.61 (m, 1H), 1.44 – 1.38 (m, 1H), 1.28 (t, J = 7.1 Hz, 3H). **^{13}C NMR** (100 MHz, $CDCl_3$) δ 173.1, 156.6, 148.3, 128.9, 127.4, 127.4, 120.1, 115.6, 110.3, 60.3, 55.4, 42.3, 40.0, 32.2, 31.2, 24.7, 14.3. **HRMS (EI)**: m/z: [M]$^+$ Calcd. For $C_{17}H_{22}O_3$: 274.1569, found: 274.1563. **HPLC** analysis of the product: Daicel Chiralpak IG column; hexane/2–propanol = 98/02, 0.5 mL/min, 245 nm; Retention times: 20.00 min (minor), 21.25 min (major). $[\alpha]^{20}_D$ = 11.1 (c = 0.5, $CHCl_3$).

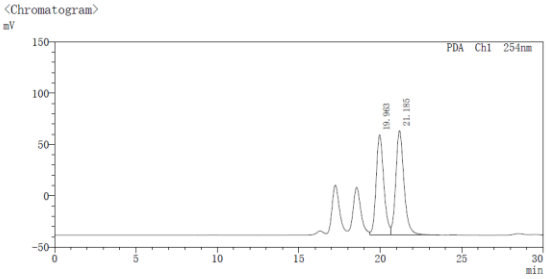

<Peak Table>
PDA Ch1 254nm

No.	Ret. Time(min)	Height	Height%	Area	Area%
1	19.963	97501	48.970	3186080	47.220
2	21.185	101605	51.030	3561176	52.780
Total		199106	100.000	6747256	100.000

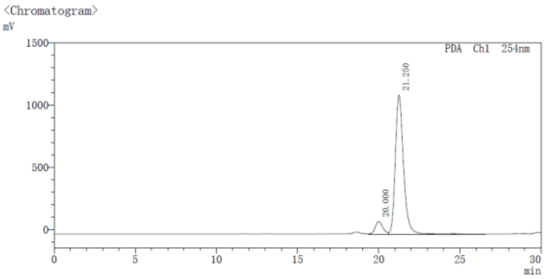

<Peak Table>
PDA Ch1 254nm

No.	Ret. Time(min)	Height	Height%	Area	Area%
1	20.000	101305	8.307	3273573	7.726
2	21.250	1118159	91.693	39095117	92.274
Total		1219464	100.000	42368689	100.000

Ethyl *(R,E)*-2-(2-(2-acetylbenzylidene)cyclopentyl)acetate (2n)

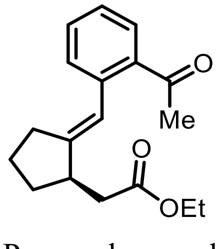

Prepared according to typical procedure **B**, after a flash column chromatography (petroleum ether/ethyl acetate = 20/1) afforded the product as colorless oil (92.4 mg, 61% yield) with 65% *ee*.

^{1}H NMR (400 MHz, $CDCl_3$) δ 7.61 (dd, J = 7.7, 1.4 Hz, 1H), 7.41 (dd, J = 7.4, 1.5 Hz, 1H), 7.37 – 7.32 (m, 1H), 7.30 – 7.23 (m, 2H), 6.58 (d, J = 2.5 Hz, 1H), 4.16 (q, J = 7.1 Hz, 2H), 3.06-2.98 (m, 1H), 2.70 (dd, J = 15.3, 5.3 Hz, 1H), 2.53 (s, 3H), 2.43-2.35 (m,

3H), 2.05-1.97 (m, 1H), 1.80-1.73 (m, 1H), 1.71 – 1.61 (m, 1H), 1.49 – 1.38 (m, 1H), 1.27 (t, J = 7.1 Hz, 3H). **^{13}C NMR** (101 MHz, $CDCl_3$) δ 202.6, 172.9, 149.7, 138.2, 137.8, 130.9, 129.9, 128.5, 126.2, 120.3, 60.3, 41.8, 39.6, 32.2, 30.8, 30.1, 24.5, 14.3. **HRMS (EI)**: m/z: $[M]^+$ Calcd. For $C_{18}H_{22}O_3$: 286.1569, found: 286.1563. **HPLC** analysis of the product: Daicel Chiralpak OD-H column; hexane/2-propanol = 98/02, 0.5 mL/min, 245 nm; Retention times: 19.55 min (minor), 24.11 min (major). $[\alpha]^{20}_D$ = 6.3 (c = 0.5, $CHCl_3$).

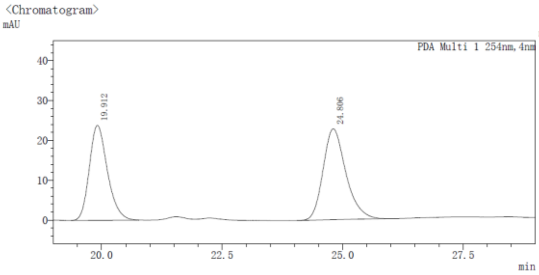

<Peak Table>
PDA Ch1 254nm

No.	Ret. Time(min)	Height	Height%	Area	Area%
1	19.912	23850	51.094	623236	45.318
2	24.806	22829	48.906	752000	54.682
Total		46679	100.000	1375236	100.000

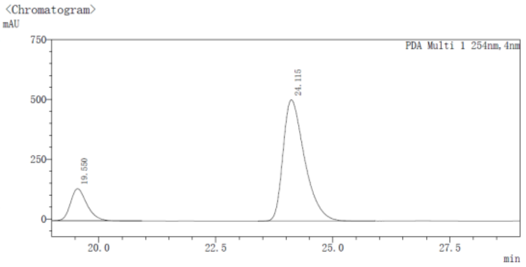

<Peak Table>
PDA Ch1 254nm

No.	Ret. Time(min)	Height	Height%	Area	Area%
1	19.550	134950	21.021	3395718	17.289
2	24.115	507039	78.979	16245138	82.711
Total		641989	100.000	19640856	100.000

Ethyl *(R,E)*–2–(2–(naphthalen–2–ylmethylene)cyclopentyl)acetate (2o)

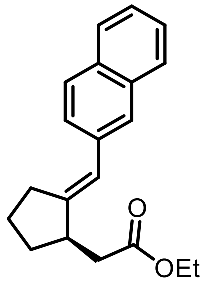

Prepared according to typical procedure **B**, after a flash column chromatography (petroleum ether/ethyl acetate = 20/1) afforded the product as colorless oil (71.3 mg, 60% yield) with 96% *ee*.

^{1}H NMR (400 MHz, $CDCl_3$) δ 7.79 (dd, J = 8.0, 6.5 Hz, 3H), 7.75 – 7.71 (m, 1H), 7.48 – 7.41 (m, 3H), 6.43 (d, J = 2.4 Hz, 1H), 4.19 (q, J = 7.1 Hz, 2H), 3.08 (t, J = 7.4 Hz, 1H), 2.82 – 2.66 (m, 3H), 2.41 (dd, J = 15.1, 9.2 Hz, 1H), 2.06–1.99 (m, 1H), 1.95–1.85 (m, 1H), 1.76–1.69 (m, 1H), 1.51 – 1.41 (m, 1H), 1.29 (t, J = 7.1 Hz, 3H). ^{13}C NMR (100 MHz, $CDCl_3$) δ 173.0, 149.2, 136.0, 133.5, 131.9, 127.9, 127.6, 127.5, 126.9, 126.7, 126.0, 125.5, 121.4, 60.4, 42.8, 39.8, 32.1, 31.5, 24.7, 14.3. **HRMS (EI)**: m/z: $[M]^+$ Calcd. For $C_{22}H_{22}O_2$: 294.1620, found: 294.1617. **HPLC** analysis of the product: Daicel Chiralpak OD–H column; hexane/2–propanol = 98/02, 0.2 mL/min, 210 nm;

Retention times: 41.76 min (major), 46.95 min (minor). $[\alpha]^{20}_{D} = -16.7$ ($c = 0.5$, $CHCl_3$).

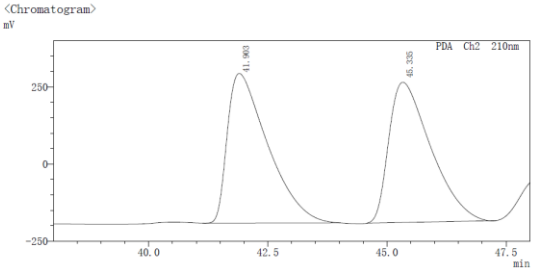

<Peak Table>
PDA Ch2 210nm

No.	Ret.Time(min)	Height	Height%	Area	Area%
1	41.903	485297	51.643	28604372	50.667
2	45.335	454425	48.357	27850872	49.333
Total		939722	100.000	56455244	100.000

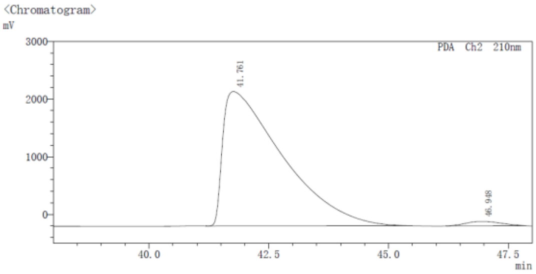

<Peak Table>
PDA Ch2 210nm

No.	Ret.Time(min)	Height	Height%	Area	Area%
1	41.761	2329553	96.799	201684442	98.139
2	46.948	77038	3.201	3825486	1.861
Total		2406591	100.000	205509928	100.000

Ethyl (R,E)–2–(2–((1H–indol–5–yl)methylene)cyclopentyl)acetate (2p)

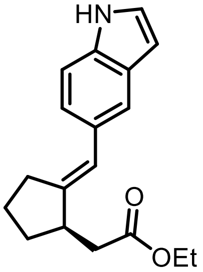

Prepared according to typical procedure **B**, after a flash column chromatography (petroleum ether/ethyl acetate = 5/1) afforded the product as a yellow solid (66.2 mg, 58% yield) with 80% *ee*. m.p.: 60–61 ℃.

^{1}H NMR (400 MHz, $CDCl_3$) δ 8.14 (s, 1H), 7.62 – 7.57 (m, 1H), 7.33 (d, J = 8.5 Hz, 1H), 7.21 – 7.15 (m, 2H), 6.54–6.52 (m, 1H), 6.39 (d, J = 2.4 Hz, 1H), 4.19 (q, J = 7.1 Hz, 2H), 3.09–3.00 (m, 1H), 2.79 – 2.60 (m, 3H), 2.38 (dd, J = 15.0, 9.3 Hz, 1H), 2.04–1.96 (m, 1H), 1.92–1.82 (m, 1H), 1.76 – 1.67 (m, 1H), 1.47 – 1.39 (m, 1H), 1.29 (t, J = 7.1 Hz, 3H). **^{13}C NMR** (100 MHz, $CDCl_3$) δ 173.2, 145.5, 134.4, 130.6, 128.0, 124.5, 123.3, 122.1, 120.1, 110.7, 102.8, 60.3, 42.6, 40.0, 32.2, 31.3, 24.8, 14.3. **HRMS (EI)**: m/z: $[M]^+$ Calcd. For $C_{18}H_{21}NO_2$: 283.1572, found: 283.1565. **HPLC** analysis of the product: Daicel Chiralpak AD–H column; hexane/2–propanol = 90/10, 0.5 mL/min, 245 nm; Retention times: 28.13 min (major), 29.13 min (minor). $[\alpha]^{20}_{D} = -12.1$ ($c = 0.5$, $CHCl_3$).

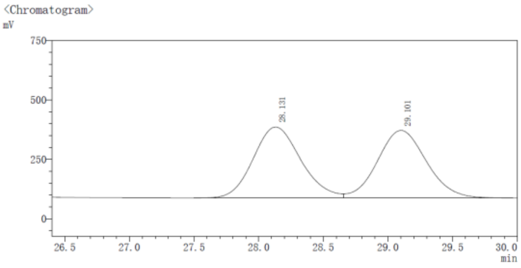

<Peak Table>
PDA Ch2 254nm

No.	Ret. Time (min)	Height	Height%	Area	Area%
1	28.131	299037	51.225	7666203	50.589
2	29.101	284734	48.775	7487775	49.411
Total		583771	100.000	15153978	100.000

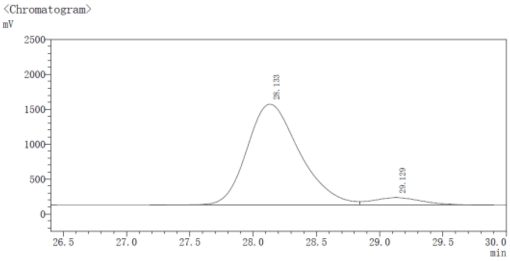

<Peak Table>
PDA Ch2 254nm

No.	Ret. Time (min)	Height	Height%	Area	Area%
1	28.133	1442965	93.320	41673168	93.821
2	29.129	103295	6.680	2744527	6.179
Total		1546260	100.000	44417695	100.000

Ethyl *(R,E)*–2–(2–(thiophen–2–ylmethylene)cyclopentyl)acetate (2q)

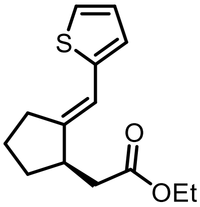

Prepared according to typical procedure **B**, after a flash column chromatography (petroleum ether/ethyl acetate = 20/1) afforded the product as colorless oil (53.0 mg, 54% yield) with 94% *ee*.

^{1}H NMR (400 MHz, $CDCl_3$) δ 7.22 (d, *J* = 5.2 Hz, 1H), 7.01 (dd, *J* = 5.1, 3.5 Hz, 1H), 6.92 (d, *J* = 3.7 Hz, 1H), 6.50 (d, *J* = 2.5 Hz, 1H), 4.17 (q, *J* = 7.1 Hz, 2H), 3.05–2.98 (m, 1H), 2.67 – 2.53 (m, 3H), 2.34 (dd, *J* = 15.1, 9.1 Hz, 1H), 2.05–1.96 (m, 1H), 1.95–1.88 (m, 1H), 1.78–1.71 (m, 1H), 1.47 – 1.40 (m, 1H), 1.28 (t, *J* = 6.9 Hz, 3H). **^{13}C NMR** (100 MHz, $CDCl_3$) δ 172.8, 147.0, 142.4, 126.9, 125.4, 124.3, 114.8, 60.4, 42.5, 39.3, 32.7, 31.8, 24.5, 14.3. **HRMS (EI)**: m/z: $[M]^+$ Calcd. For $C_{14}H_{18}O_2S$: 250.1028, found: 250.1025. **HPLC** analysis of the product: Daicel Chiralpak OJ–H column; hexane/2–propanol = 98/02, 0.5 mL/min, 245 nm; Retention times: 21.67 min (major), 22.90 min (minor). $[\alpha]^{20}_{D}$ = 6.0 (*c* = 0.5, $CHCl_3$).

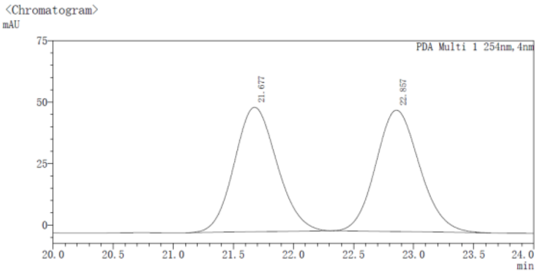

<Peak Table>
PDA Ch1 254nm

No.	Ret. Time (min)	Height	Height%	Area	Area%
1	21.677	50721	50.624	1254402	49.977
2	22.857	49472	49.376	1255575	50.023
Total		100193	100.000	2509977	100.000

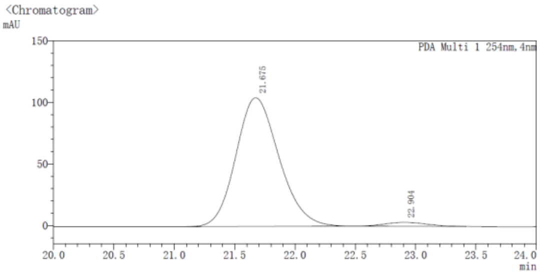

<Peak Table>
PDA Ch1 254nm

No.	Ret. Time (min)	Height	Height%	Area	Area%
1	21.675	104605	97.003	2602226	97.142
2	22.904	3232	2.997	76562	2.858
Total		107837	100.000	2678788	100.000

Methyl *(R,E)*–2–(2–benzylidenecyclopentyl)acetate (2r)

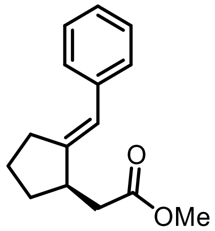

Prepared according to typical procedure **B**, after a flash column chromatography (petroleum ether/ethyl acetate = 20/1) afforded the product as colorless oil (36.5 mg, 40% yield) with 94% *ee*.

^{1}H NMR (400 MHz, $CDCl_3$) δ 7.35–7.33 (m, 4H), 7.22–7.19 (m, 1H), 6.29 (d, *J* = 2.4 Hz, 1H), 3.73 (s, 3H), 3.05–3.04 (m, 1H), 2.75 – 2.57 (m, 3H), 2.41 (dd, *J* = 15.2, 9.3 Hz, 1H), 2.04 – 1.98 (m, 1H), 1.92 – 1.85 (m, 1H), 1.76 – 1.70 (m, 1H), 1.46–1.41 (m, 1H). **^{13}C NMR** (100 MHz, $CDCl_3$) δ 208.4, 149.1, 138.4, 128.2, 128.2, 126.0, 121.0, 49.1, 41.6, 32.3, 31.4, 30.5, 24.8. **HRMS (EI)**: m/z: $[M]^+$ Calcd. For $C_{15}H_{18}O_2$: 230.1307, found: 230.1303. **HPLC** analysis of the product: Daicel Chiralpak OD–H column; hexane/2–propanol = 98/02, 0.5 mL/min, 245 nm; Retention times: 14.68 min (major), 17.57 min (minor). $[\alpha]^{20}_D$ = –12.8 (*c* = 0.5, $CHCl_3$).

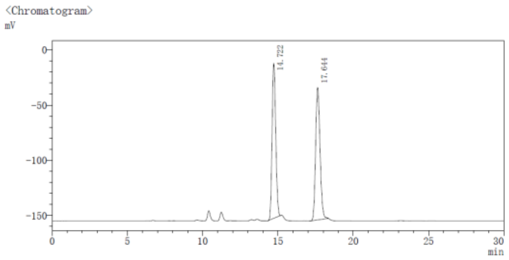

<Peak Table>
PDA Ch1 254nm

No.	Ret.Time(min)	Height	Height%	Area	Area%
1	14.722	140456	53.909	2369112	49.290
2	17.644	120085	46.091	2437343	50.710
Total		260542	100.000	4806455	100.000

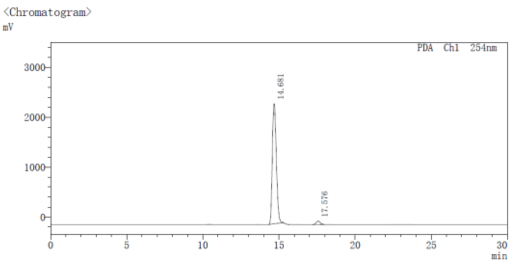

<Peak Table>
PDA Ch1 254nm

No.	Ret.Time(min)	Height	Height%	Area	Area%
1	14.681	2407844	97.276	44111197	97.171
2	17.576	67415	2.724	1284328	2.829
Total		2475260	100.000	45395525	100.000

***tert*–Butyl *(R,E)*–2–(2–benzylidenecyclopentyl)acetate (2s)**

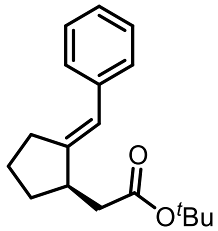

Prepared according to typical procedure **B**, after a flash column chromatography (petroleum ether/ethyl acetate = 20/1) afforded the product as colorless oil (66.8 mg, 61% yield) with 92% *ee*.

^{1}H NMR (400 MHz, $CDCl_3$) δ 7.34–7.29 (m, 4H), 7.19–7.15 (m, 1H), 6.27 (d, *J* = 2.4

Hz, 1H), 3.02–2.95 (m, 1H), 2.69 – 2.54 (m, 3H), 2.28 (dd, J = 14.9, 9.1 Hz, 1H), 2.02 – 1.94 (m, 1H), 1.89–1.82 (m, 1H), 1.72 – 1.64 (m, 1H), 1.49–1.45 (m, 10H). **^{13}C NMR** (100 MHz, $CDCl_3$) δ 172.3, 148.8, 138.5, 128.2, 128.1, 125.9, 121.1, 80.3, 42.9, 41.0, 32.0, 31.4, 28.2, 24.7. **HRMS (ESI)**: m/z: $[M+Na]^+$ Calcd. For $C_{18}H_{24}NaO_2$: 295.1674, found: 295.1669. **HPLC** analysis of the product: Daicel Chiralpak OJ–H*2 column; hexane/2–propanol = 98/02, 0.5 mL/min, 245 nm; Retention times: 23.23 min (minor), 24.07 min (major). $[\alpha]^{20}_D$ = 13.9 (c = 0.5, $CHCl_3$).

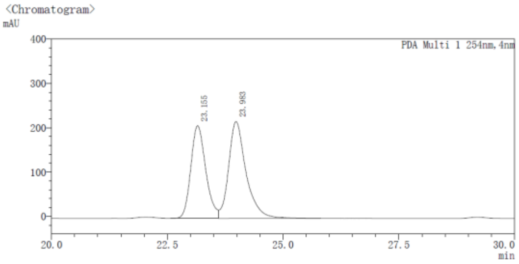

<Peak Table>
PDA Ch1 254nm

No.	Ret. Time (min)	Height	Height%	Area	Area%
1	23.155	208895	48.905	4678780	45.093
2	23.983	218253	51.095	5697011	54.907
Total		427149	100.000	10375791	100.000

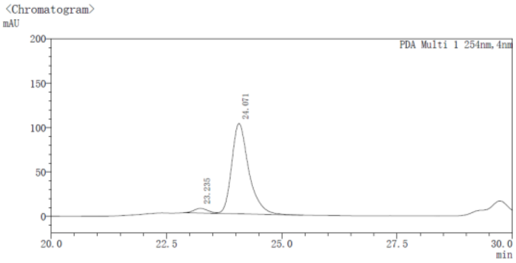

<Peak Table>
PDA Ch1 254nm

No.	Ret. Time (min)	Height	Height%	Area	Area%
1	23.235	5209	4.865	109155	3.990
2	24.071	101867	95.135	2626868	96.010
Total		107076	100.000	2736023	100.000

Ethyl (*R*,*Z*)-2-(4-benzylidenetetrahydrofuran-3-yl)acetate (2t)

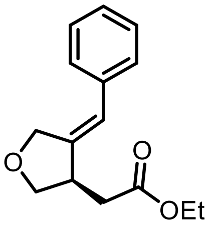

Prepared according to typical procedure **B**, after a flash column chromatography (petroleum ether/ethyl acetate = 20/1) afforded the product as colorless oil (27.5 mg, 24% yield) with 77% *ee*.

^{1}H NMR (400 MHz, $CDCl_3$) δ 7.37 (t, J = 7.7 Hz, 2H), 7.32 – 7.21 (m, 1H), 7.19 – 7.12 (m, 2H), 6.40 (d, J = 2.3 Hz, 1H), 4.74 – 4.60 (m, 2H), 4.20 (q, J = 7.1 Hz, 2H), 4.12 (dd, J = 8.7, 6.5 Hz, 1H), 3.68 (dd, J = 8.7, 5.5 Hz, 1H), 3.39 – 3.24 (m, 1H), 2.70 (dd, J = 16.0, 5.4 Hz, 1H), 2.55 (dd, J = 16.0, 9.3 Hz, 1H), 1.30 (t, J = 7.1 Hz, 3H). **^{13}C NMR** (101 MHz, $CDCl_3$) δ 172.1, 143.7, 137.0, 128.6, 128.0, 126.9, 121.5, 72.6, 70.0, 60.6, 41.9, 38.0, 14.3. **HPLC** analysis of the product: Daicel Chiralpak OD–H column; hexane/2–propanol = 98/02, 0.5 mL/min, 245 nm; Retention times: 27.53 min (major), 29.49 min (minor).

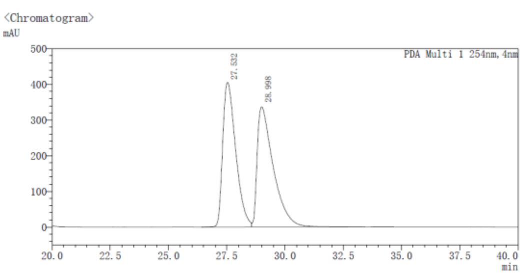

<Peak Table>
PDA Ch1 254nm

No.	Ret. Time (min)	Height	Height%	Area	Area%
1	27.532	405522	54.627	15738310	49.295
2	28.998	336827	45.373	16188493	50.705
Total		742348	100.000	31926803	100.000

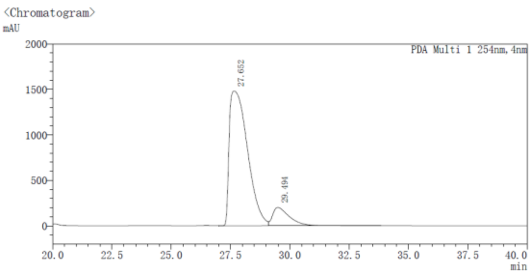

<Peak Table>
PDA Ch1 254nm

No.	Ret. Time (min)	Height	Height%	Area	Area%
1	27.652	1482490	87.983	79585629	88.751
2	29.494	202478	12.017	10087110	11.249
Total		1684968	100.000	89672739	100.000

Ethyl *(R,Z)*–2–(4–(4–methoxybenzylidene)–1–tosylpyrrolidin–3–yl)acetate (2u)

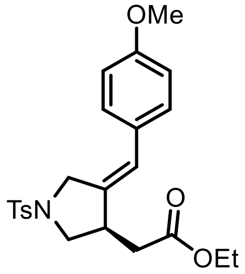

Prepared according to typical procedure **B**, after a flash column chromatography (petroleum ether/ethyl acetate = 5/1) afforded the product as a white solid (133.4 mg, 78% yield) with 90% *ee*. m.p.: 63–64 ℃.

^{1}H NMR (400 MHz, $CDCl_3$) δ 7.75–7.72 (m, 2H), 7.34 (d, *J* = 7.9 Hz, 2H), 7.10 – 7.05 (m, 2H), 6.92 – 6.87 (m, 2H), 6.24 (s, 1H), 4.22 – 4.01 (m, 5H), 3.84 (d, *J* = 1.0 Hz, 3H), 3.49 – 3.43 (m, 1H), 3.21 (d, *J* = 7.7 Hz, 1H), 3.12 (dd, *J* = 9.4, 5.0 Hz, 1H), 2.62 (dd, *J* = 16.1, 5.1 Hz, 1H), 2.44 (s, 3H), 1.30–1.26 (m, 3H). **^{13}C NMR** (100 MHz, $CDCl_3$) δ 171.6, 158.8, 143.8, 136.6, 132.8, 129.8, 129.4, 128.9, 127.8, 123.1, 114.1, 60.7, 55.3, 52.2, 50.4, 41.0, 38.1, 21.5, 14.2. **HRMS (EI)**: m/z: $[M]^+$ Calcd. for $C_{23}H_{27}NO_5S$: 429.1610, found: 429.1605. **HPLC** analysis of the product: Daicel Chiralpak IA column; hexane/2–propanol = 80/20, 0.5 mL/min, 245 nm; Retention times: 16.43 min (major), 19.60 min (minor). $[\alpha]^{20}_{D}$ = 19.9 (*c* = 0.5, $CHCl_3$).

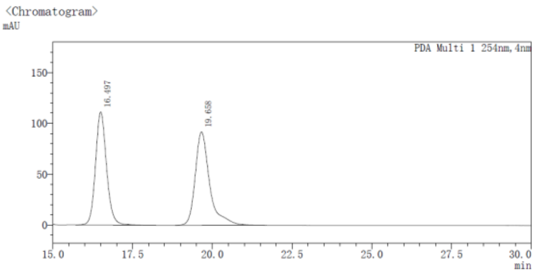

<Peak Table>

PDA Ch1 254nm

No.	Ret. Time(min)	Height	Height%	Area	Area%
1	16.497	111281	54.828	2670166	48.308
2	19.658	91681	45.172	2857157	51.692
Total		202963	100.000	5527323	100.000

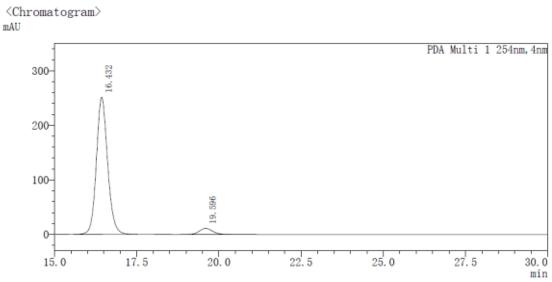

<Peak Table>

PDA Ch1 254nm

No.	Ret. Time(min)	Height	Height%	Area	Area%
1	16.432	251291	95.718	5998543	94.809
2	19.596	11243	4.282	328434	5.191
Total		262534	100.000	6326977	100.000

Ethyl *(R,Z)*–2–(4–(3–cyanobenzylidene)–1–tosylpyrrolidin–3–yl)acetate (2v)

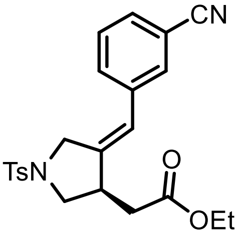

Prepared according to typical procedure **B**, after a flash column chromatography (petroleum ether/ethyl acetate = 5/1) afforded the product as colorless oil (49.8 mg, 30% yield) with 82% *ee*.

^{1}H NMR (400 MHz, $CDCl_3$) δ 7.71 (d, J = 7.9 Hz, 2H), 7.36 – 7.30 (m, 3H), 7.24 – 7.21 (m, 1H), 7.17 – 6.99 (m, 2H), 6.32 – 6.19 (m, 1H), 4.14 (q, J = 7.1 Hz, 3H), 4.08 – 3.99 (m, 1H), 3.49–3.44 (m, 1H), 3.21 (s, 1H), 3.09 (dd, J = 9.6, 5.5 Hz, 1H), 2.63 – 2.55 (m, 1H), 2.48 – 2.40 (m, 4H), 1.25 (t, J = 7.1 Hz, 3H). **^{13}C NMR** (100 MHz, $CDCl_3$) δ 171.3, 144.1, 142.4, 137.4, 132.5, 132.1, 131.5, 130.7, 129.9, 129.5, 127.8, 127.2, 121.7, 118.5, 112.9, 60.9, 52.0, 50.3, 41.0, 37.8, 21.6, 14.2. **HRMS (ESI)**: m/z: [M+H]$^+$ Calcd. for $C_{23}H_{25}N_2O_4S$: 425.1535, found: 425.1530. **HPLC** analysis of the product: Daicel Chiralpak IB N-5 column; hexane/2–propanol = 10/90, 0.5 mL/min, 245 nm; Retention times: 16.48 min (major), 17.75 min (minor). $[\alpha]^{20}_D$ = 35.2 (c = 0.5, $CHCl_3$).

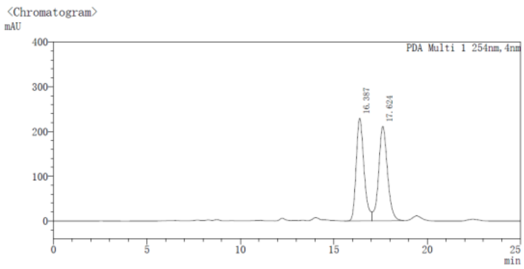

<Peak Table>

PDA Ch1 254nm

No.	Ret. Time(min)	Height	Height%	Area	Area%
1	16.387	229349	52.087	6700684	49.070
2	17.624	210970	47.913	6954793	50.930
Total		440320	100.000	13655477	100.000

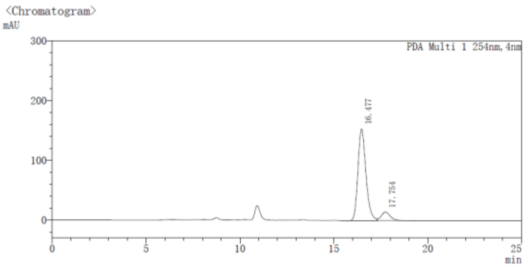

<Peak Table>

PDA Ch1 254nm

No.	Ret. Time(min)	Height	Height%	Area	Area%
1	16.477	153676	91.494	4528911	90.695
2	17.754	14287	8.506	464645	9.305
Total		167963	100.000	4993556	100.000

(R,Z)–3–Benzylidene–4–methyl–1–tosylpyrrolidine (2w)

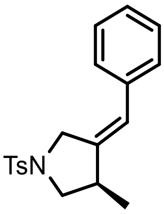

Prepared according to typical procedure **B**, after a flash column chromatography (petroleum ether/ethyl acetate = 5/1) afforded the product as a yellow solid (62.2 mg, 47% yield) with 72% *ee*. m.p.: 67–68 ℃.

^{1}H NMR (400 MHz, $CDCl_3$) δ 7.72 (d, *J* = 8.2 Hz, 2H), 7.37 – 7.29 (m, 4H), 7.23 (t, *J* = 7.4 Hz, 1H), 7.16 – 7.10 (m, 2H), 6.22 (d, *J* = 2.5 Hz, 1H), 4.29 – 4.20 (m, 1H), 4.08 – 4.02 (m, 1H), 3.55 (dd, *J* = 9.0, 7.2 Hz, 1H), 2.90–2.84 (m, 1H), 2.73 (t, *J* = 8.5 Hz, 1H), 2.41 (s, 3H), 1.17 (d, *J* = 6.7 Hz, 3H). **^{13}C NMR** (100 MHz, $CDCl_3$) δ 143.7, 141.8, 136.6, 133.0, 129.8, 128.6, 128.1, 127.8, 127.0, 122.1, 53.9, 50.8, 39.1, 21.5, 17.0. **HRMS (EI)**: m/z: $[M]^+$ Calcd. for $C_{19}H_{21}NO_2S$: 327.1293, found: 327.1290. **HPLC** analysis of the product: Daicel Chiralpak IF column; hexane/2–propanol = 90/10, 0.5 mL/min, 245 nm; Retention times: 32.89 min (major), 34.82 min (minor). $[\alpha]^{20}_D$ = 2.9 (*c* = 0.5, $CHCl_3$).

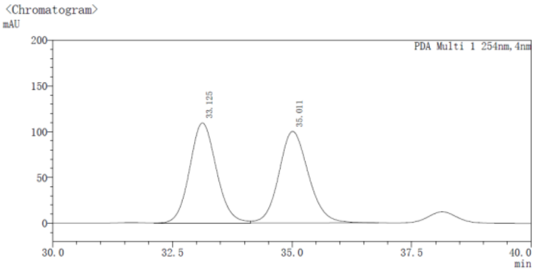

<Peak Table>
PDA Ch1 254nm

No.	Ret.Time(min)	Height	Height%	Area	Area%
1	33.125	109446	52.230	4260088	49.677
2	35.011	100101	47.770	4315426	50.323
Total		209548	100.000	8575514	100.000

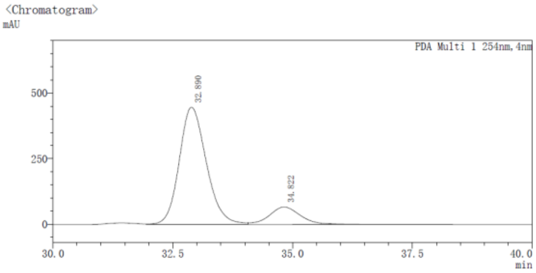

<Peak Table>
PDA Ch1 254nm

No.	Ret.Time(min)	Height	Height%	Area	Area%
1	32.890	446374	87.116	17545932	85.590
2	34.822	66015	12.884	2953948	14.410
Total		512389	100.000	20499880	100.000

Dimethyl *(R,E)*–3–(2–ethoxy–2–oxoethyl)–4–(4–(ethoxycarbonyl)benzylidene)cyclopentane–1,1–dicarboxylate (2x)

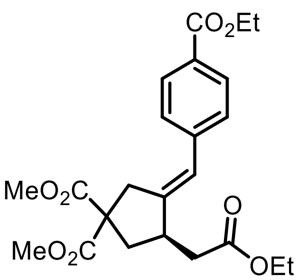

Prepared according to typical procedure **B**, after a flash column chromatography (petroleum ether/ethyl acetate = 10/1) afforded the product as colorless oil (104.8 mg, 60% yield) with 67% *ee*.

^{1}H NMR (400 MHz, $CDCl_3$) δ 7.99 (d, *J* = 8.3 Hz, 2H), 7.31 (d, *J* = 8.3 Hz, 2H), 6.29 (q, *J* = 2.5 Hz, 1H), 4.37 (q, *J* = 7.1 Hz, 2H), 4.19–4.13 (m, 2H), 3.74 (s, 3H), 3.71 (s, 3H), 3.40 – 3.32 (m, 1H), 3.26 – 3.16 (m, 2H), 2.76 – 2.68 (m, 2H), 2.45 (dd, *J* = 15.6, 8.5 Hz, 1H), 2.00 (dd, *J* = 13.3, 10.5 Hz, 1H), 1.39 (t, *J* = 7.1 Hz, 3H), 1.27 (t, *J* = 7.1 Hz, 3H). **^{13}C NMR** (100 MHz, $CDCl_3$) δ 172.0, 171.8, 171.7, 166.4, 146.3, 141.9, 129.6, 128.4, 128.2, 122.1, 60.9, 60.6, 59.1, 53.0, 52.9, 40.8, 39.1, 38.9, 38.9, 14.3, 14.3. **HRMS (EI)**: m/z: $[M]^{+}$ Calcd. for $C_{23}H_{28}O_8$: 432.1784, found: 432.1781. **HPLC** analysis of the product: Daicel Chiralpak AD–H column; hexane/2–propanol = 50/50, 0.15 mL/min, 210 nm; Retention times: 42.90 min (major), 45.15 min (minor). $[\alpha]^{20}_{D}$ = 6.6 (*c* 0.5, $CHCl_3$).

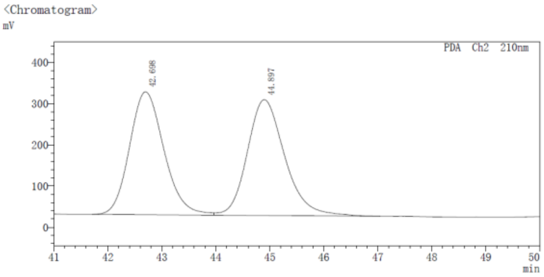

<Peak Table>
PDA Ch2 210nm

No.	Ret.Time(min)	Height	Height%	Area	Area%
1	42.698	298133	51.492	13227742	49.382
2	44.897	280854	48.508	13558953	50.618
Total		578987	100.000	26786695	100.000

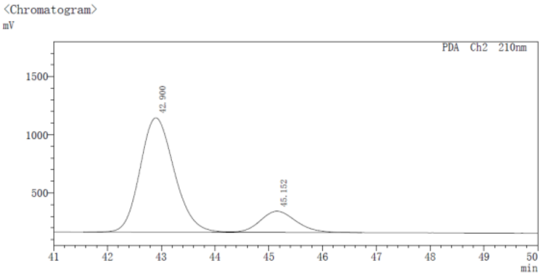

<Peak Table>
PDA Ch2 210nm

No.	Ret.Time(min)	Height	Height%	Area	Area%
1	42.900	982454	84.364	43524904	83.678
2	45.152	182085	15.636	8489944	16.322
Total		1164539	100.000	52014848	100.000

***(1S,2R,5R)*–2–*iso*–Propyl–5–methylcyclohexyl 4–(*(E)*–(*(R)*–2–(2–ethoxy–2–oxoethyl)cyclopentylidene)methyl)benzoate (2y)**

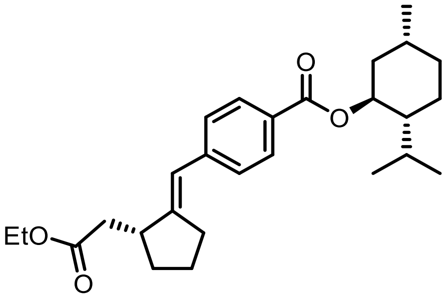

Prepared according to typical procedure **B**, after a flash column chromatography (petroleum ether/ethyl acetate = 20/1) afforded the product as colorless oil (117.2 mg, 69% yield) with >20:1 *dr*.

^{1}H NMR (400 MHz, $CDCl_3$) δ 8.00 (d, *J* = 8.0 Hz, 2H), 7.36 (d, *J* = 8.0 Hz, 2H), 6.33 (d, *J* = 2.8 Hz, 1H), 4.93 (td, *J* = 11.0, 4.3 Hz, 1H), 4.18 (q, *J* = 7.1 Hz, 2H), 3.05 (t, *J* = 7.6 Hz, 1H), 2.66 (td, *J* = 15.5, 7.3 Hz, 3H), 2.39 (dd, *J* = 15.2, 9.0 Hz, 1H), 2.20 – 2.10 (m, 1H), 2.07 – 1.83 (m, 4H), 1.72 (tq, *J* = 12.8, 5.4 Hz, 3H), 1.56 (t, *J* = 10.6 Hz, 2H), 1.44 (dq, *J* = 15.6, 8.0 Hz, 1H), 1.28 (t, *J* = 7.1 Hz, 3H), 1.23 – 1.03 (m, 3H), 0.93 (dd, *J* = 6.9, 3.9 Hz, 6H), 0.81 (d, *J* = 6.9 Hz, 3H). **^{13}C NMR** (100 MHz, $CDCl_3$) δ 172.8, 166.0, 151.6, 142.8, 129.5, 128.1, 128.0, 120.8, 74.7, 60.4, 47.3, 42.9, 41.0, 39.6, 34.4, 32.0, 31.6, 31.5, 26.5, 24.7, 23.7, 22.1, 20.8, 16.6, 14.3. **HRMS (ESI)**: m/z: $[M+Na]^+$ Calcd. for $C_{27}H_{38}NaO_4$: 449.2668, found: 449.2662.

(*(3aR,5R,5aS,8aS,8bR)*–2,2,7,7–Tetramethyltetrahydro–5H–bis([1,3]dioxolo)[4,5–b:4',5'–d]pyran–5–yl)methyl 4–(*(E)*–(*(R)*–2–(2–ethoxy–2–oxoethyl)cyclopentylidene)methyl)benzoate (2z)

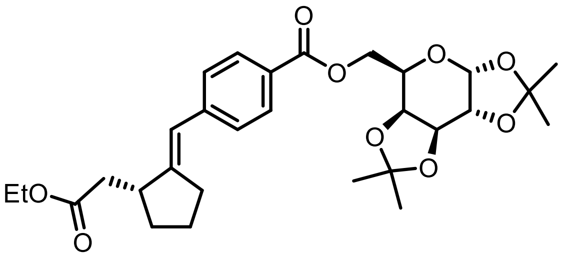

Prepared according to typical procedure **B**, after a flash column chromatography (petroleum ether/ethyl acetate = 10/1) afforded the product as colorless oil (129.5 mg, 61% yield) with >20:1 *dr*.

^{1}H NMR (400 MHz, $CDCl_3$) δ 7.99 (d, *J* = 8.3 Hz, 2H), 7.34 (d, *J* = 8.2 Hz, 2H), 6.30

(d, J = 2.5 Hz, 1H), 5.57 (d, J = 4.9 Hz, 1H), 4.65 (dd, J = 7.9, 2.4 Hz, 1H), 4.52 (dd, J = 11.5, 4.9 Hz, 1H), 4.41 (dd, J = 11.5, 7.4 Hz, 1H), 4.36 – 4.32 (m, 2H), 4.19 – 4.14 (m, 3H), 3.05–3.00 (m, 1H), 2.69–2.60 (m, 3H), 2.38 (dd, J = 15.2, 9.0 Hz, 1H), 2.04–1.96 (m, 1H), 1.91–1.85 (m, 1H), 1.74 – 1.66 (m, 1H), 1.52 (s, 3H), 1.48 (s, 3H), 1.36 (s, 3H), 1.33 (s, 3H), 1.27 (t, J = 7.1 Hz, 4H). **^{13}C NMR** (100 MHz, $CDCl_3$) δ 172.7, 166.3, 151.8, 143.1, 129.7, 128.0, 127.3, 120.8, 109.7, 108.8, 96.3, 71.2, 70.8, 70.6, 66.2, 63.8, 60.4, 43.0, 39.5, 32.0, 31.7, 26.0, 26.0, 25.0, 24.7, 24.5, 14.3. **HRMS (EI)**: m/z: $[M]^+$ Calcd. for $C_{29}H_{38}O_9$: 530.2516, found: 530.2513.

Ethyl N–(tert–butoxycarbonyl)–N–(4–(*(E)*–(*(R)*–2–(2–ethoxy–2–oxoethyl)cyclopentylidene)methyl)benzoyl)–L–phenylalaninate (2aa)

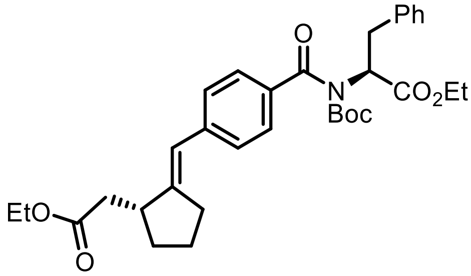

Prepared according to typical procedure **B**, after a flash column chromatography (petroleum ether/ethyl acetate = 10/1) afforded the product as colorless oil (183.2 mg, 80% yield) with >20:1 *dr*.

^{1}H NMR (400 MHz, $CDCl_3$) δ 7.28 – 7.23 (m, 5H), 7.13 (dd, J = 11.8, 9.4 Hz, 4H), 6.28 – 6.21 (m, 1H), 5.40 (dd, J = 10.9, 5.7 Hz, 1H), 4.29 – 4.22 (m, 2H), 4.16 (q, J = 7.1 Hz, 2H), 3.56 (dd, J = 14.3, 5.7 Hz, 1H), 3.43 (dd, J = 14.3, 10.9 Hz, 1H), 3.03–2.99 (m, 1H), 2.69 – 2.52 (m, 3H), 2.36 (dd, J = 15.1, 9.0 Hz, 1H), 2.03 – 1.95 (m, 1H), 1.92 – 1.83 (m, 1H), 1.73 – 1.65 (m, 1H), 1.27 (m, 6H), 1.07 (s, 9H), 0.96 (dd, J = 7.7, 6.4 Hz, 1H). **^{13}C NMR** (100 MHz, $CDCl_3$) δ 172.8, 170.3, 150.9, 141.4, 137.6, 134.6, 129.5, 128.5, 127.7, 127.4, 126.6, 120.7, 83.2, 61.6, 60.4, 58.8, 42.9, 39.5, 35.5, 32.0, 31.5, 27.3, 24.7, 14.3, 14.2. **HRMS (ESI)**: m/z: $[M+H]^+$ Calcd. for $C_{33}H_{41}NNaO_7$: 586.2781, found: 586.2775.

3.4 Deuterium labelling and control experiments

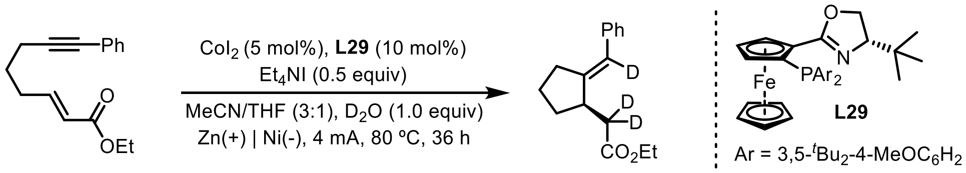

In Glove box, an oven–dried electrochemical cell with two stir bars was added enyne (0.4 mmol, 1 equiv), CoI_2 (0.02 mmol, 5 mol%), ligand (0.04 mmol, 10 mol%), Et_4NI (0.2 mmol, 0.5 equiv), D_2O (1.0 equiv), 3 mL of MeCN and 1 mL of THF. The tube was installed by a Ni foam as cathode and Zn flake as sacrificial anode. The mixture was stirred at r.t. for 15 min. The reaction mixture was electrolyzed under a constant current of 4 mA at 80 °C until the complete consumption of the starting materials which was monitored by TLC (about 36 hours). The solvent was removed in vacuo, and the crude residue was purified via column chromatography to afford the desired product.

Ethyl (*R,E*)-2-(2-(phenylmethylene-*d*)cyclopentyl)acetate-*d*$_2$ ([D]-2a)

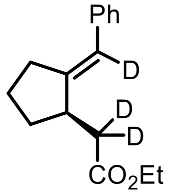

Prepared according to typical procedure **B**, after a flash column chromatography (petroleum ether/ethyl acetate = 20/1) afforded the product as colorless oil (37.9 mg, 36% yield) with 90% *ee*.

^{1}H NMR (400 MHz, $CDCl_3$) δ 7.34 (d, *J* = 6.2 Hz, 4H), 7.20 (tt, *J* = 5.8, 2.5 Hz, 1H), 6.30 (s, 0.30 H), 4.20 (q, *J* = 7.1 Hz, 2H), 3.04 (q, *J* = 7.3 Hz, 1H), 2.75 – 2.55 (m, 3H), 2.43 – 2.35 (m, 1H), 2.07 – 1.96 (m, 1H), 1.95 – 1.83 (m, 1H), 1.78 – 1.65 (m, 1H), 1.44 (m, 1H), 1.31 (t, *J* = 7.1 Hz, 3H). **HPLC** analysis of the product: Daicel Chiralpak OD–H column; hexane/2–propanol = 98/02, 0.5 mL/min, 245nm; Retention times: 12.74 min (major), 13.25 min (minor). $[\alpha]^{20}_D$ = 19.2 (*c* 0.5, $CHCl_3$).

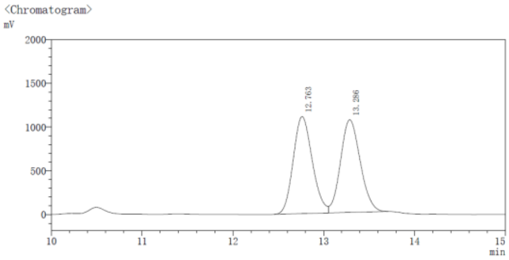

<Peak Table>
PDA Ch2 254nm

No.	Ret. Time(min)	Height	Height%	Area	Area%
1	12.763	1109215	51.129	16197180	50.274
2	13.286	1060222	48.871	16020846	49.726
Total		2169436	100.000	32218026	100.000

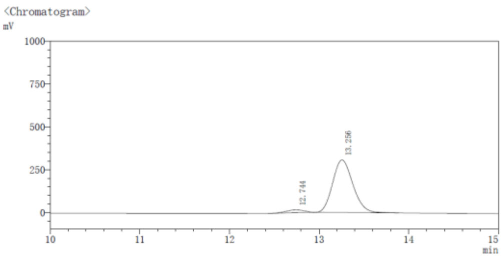

<Peak Table>
PDA Ch2 254nm

No.	Ret. Time(min)	Height	Height%	Area	Area%
1	12.744	16428	5.092	231182	4.589
2	13.256	306199	94.908	4806169	95.411
Total		322627	100.000	5037351	100.000

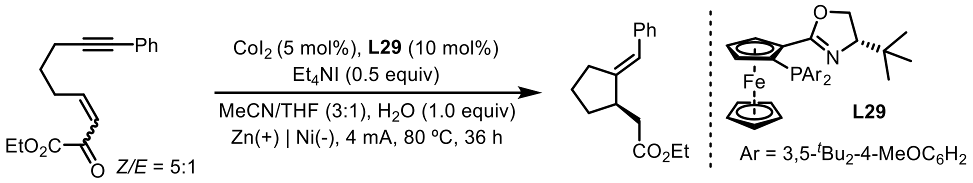

In Glove box, an oven–dried electrochemical cell with two stir bars was added enyne (0.4 mmol, 1 equiv), CoI_2 (0.02 mmol, 5 mol%), ligand (0.04 mmol, 10 mol%), Et_4NI (0.2 mmol, 0.5 equiv), H_2O (1.0 equiv), 3 mL of MeCN and 1 mL of THF. The tube was installed by a Ni foam as cathode and Zn flake as sacrificial anode. The mixture was stirred at r.t. for 15 min. The reaction mixture was electrolyzed under a constant current of 4 mA at 80 °C until the complete consumption of the starting materials which was monitored by TLC (about 36 hours). The solvent was removed in vacuo, and the crude residue was purified via column chromatography to afford the desired product.

Ethyl *(R,E)*–2–(2–benzylidenecyclopentyl)acetate (2a)

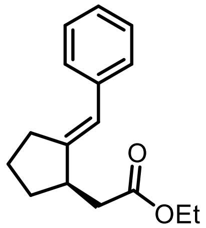

Prepared according to typical procedure **B**, after a flash column chromatography (petroleum ether/ethyl acetate = 20/1) afforded the product as colorless oil (68.4 mg, 70% yield) with 46% *ee*.

^{1}H NMR (400 MHz, $CDCl_3$) δ 7.39 – 7.31 (m, 4H), 7.24 – 7.17 (m, 1H), 6.30 (q, J = 2.5 Hz, 1H), 4.20 (q, J = 7.1 Hz, 2H), 3.12 – 3.00 (m, 1H), 2.75 – 2.57 (m, 3H), 2.40 (dd, J = 15.1, 9.2 Hz, 1H), 2.07 – 1.97 (m, 1H), 1.95 – 1.84 (m, 1H), 1.79 – 1.69 (m, 1H), 1.51 – 1.39 (m, 2H), 1.31 (t, J = 7.1 Hz, 3H). **^{13}C NMR** (100 MHz, $CDCl_3$) δ 173.0, 148.6, 138.4, 128.2 (two peaks overlap), 126.0, 121.3, 60.3, 42.7, 39.8, 32.1,

31.3, 24.7, 14.3. **HPLC** analysis of the product: Daicel Chiralpak AD–H column; hexane/2–propanol = 98/02, 0.5 mL/min, 245nm; Retention times: 48.55 min (major), 51.88 min (minor). $[\alpha]^{20}_{D}$ = 19.2 (*c* 0.5, $CHCl_3$).

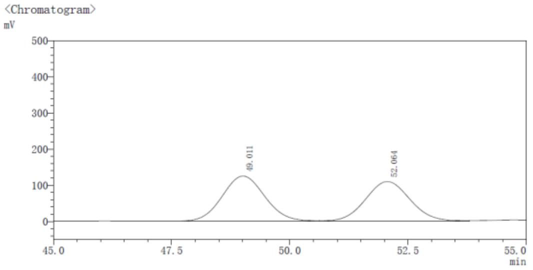

<Peak Table>

PDA Ch2 254nm

No.	Ret.Time(min)	Height	Height%	Area	Area%
1	49.011	124740	53.251	7814898	52.057
2	52.064	109509	46.749	7197190	47.943
Total		234249	100.000	15012088	100.000

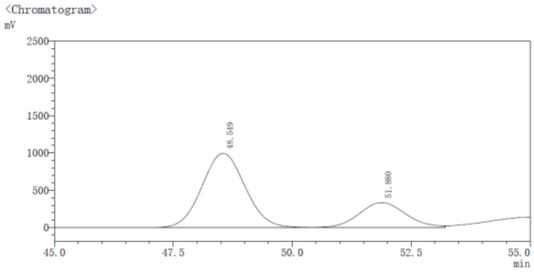

<Peak Table>

PDA Ch2 254nm

No.	Ret.Time(min)	Height	Height%	Area	Area%
1	48.549	996435	74.800	62948124	73.251
2	51.880	335689	25.200	22986561	26.749
Total		1332124	100.000	85934685	100.000

3.5 X-Ray crystal structures

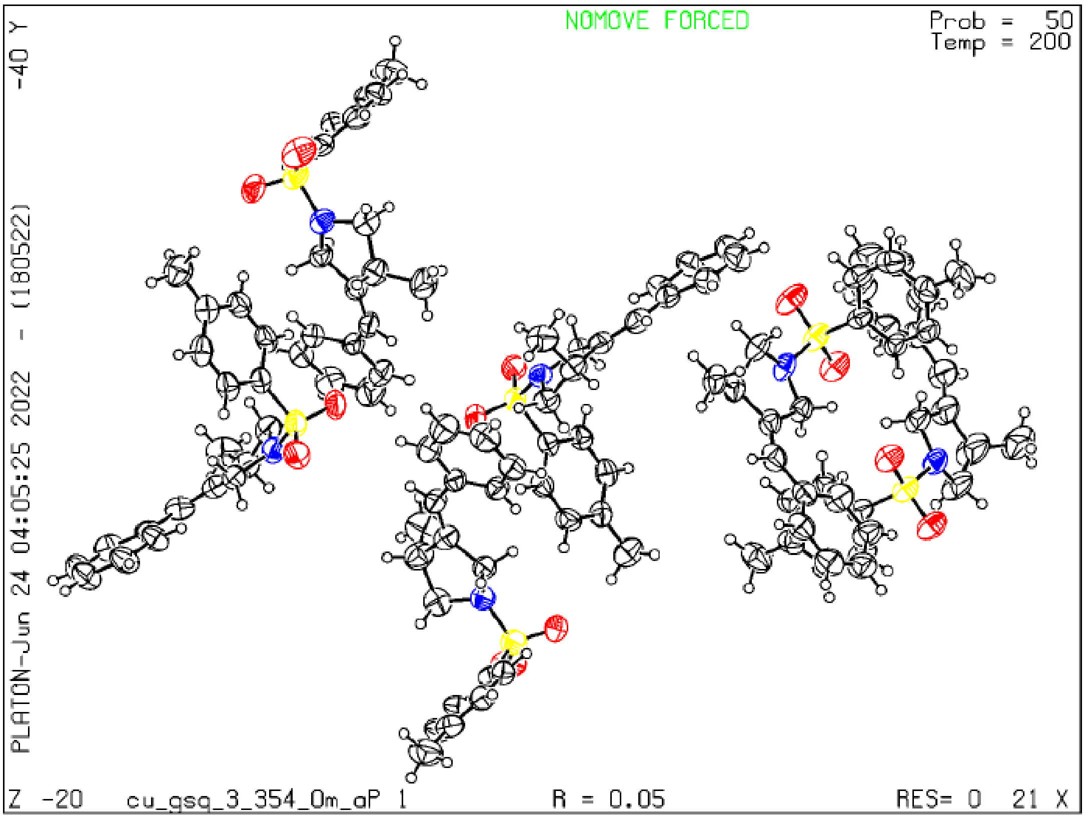

Supplementary Figure 2. ORTEP drawing of **2w** (thermal ellipsoids set at 50% probability). Recrystallization from *n*-hexane/CH_2Cl_2 afforded single crystals suitable for X-ray diffraction analysis.

3.6 DFT calculation

All the density functional theory (DFT) calculations were performed using Gaussian 09 program.[9] Geometry optimizations were performed with the M06-L functional[10] using a combined basis set (SDD effective core potential[11] for cobalt and 6-31G(d) basis[12] for the other atoms) (BS1). Harmonic frequency calculations were performed for each stationary point to ensure that it is either an energy minimum (no imaginary frequency) or a transition state (only one imaginary frequency). For each transition state, intrinsic reaction coordinate (IRC) analysis was performed to ensure that it connects the correct reactant and product. The single-point energy calculations were further performed with the M06-L functional and a combined basis set (SDD effective core potential for cobalt and the 6-311+G(d,p) basis set[13] for all other atoms) (BS2), using a self-consistent reaction field (SCRF) method called IEFPCM[14] in order to obtain energies in solution. The single-point energies corrected by the thermal correction to Gibbs free energies (TCG, obtained from frequency calculations) were used as the Gibbs free energies reported in this work, corresponding to the reference state of 1 mol/L, 298.15 K. The minimum energy crossing points (MECP) between the singlet and triplet states were located with the sobMECP program at the M06-L/BS1 level.[15] For each MECP, the electronic energy gap between it and its precursor was added to the free energy of the precursor to estimate its free energy.[16]

Proposed catalytic cycles

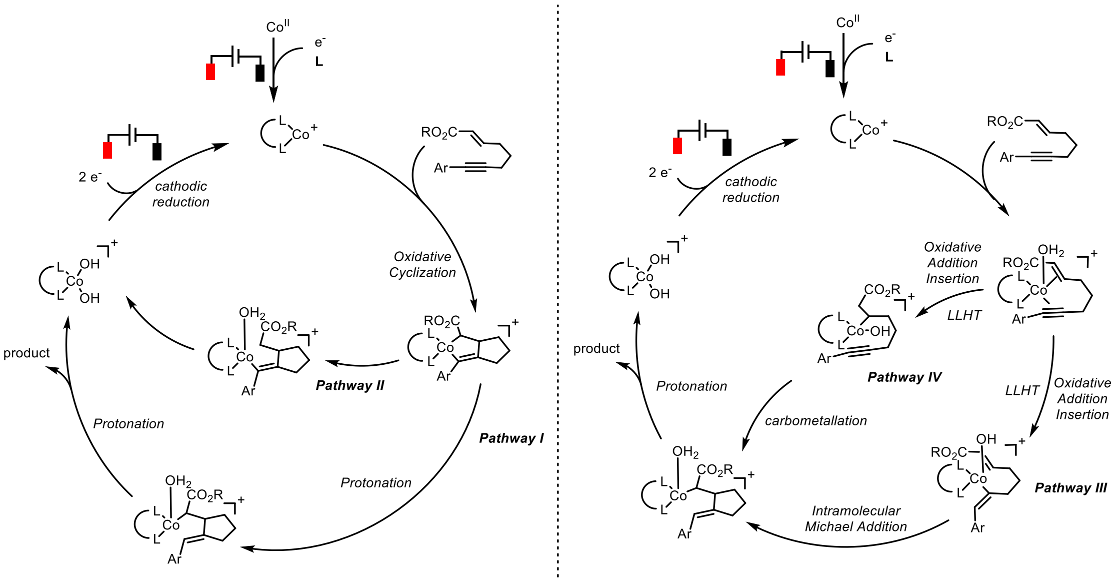

Supplementary Figure 3. Proposed possible reaction pathway.

To better understand the reaction pathway, particularly which pathway is more favorable, DFT calculations were carried out based on the mechanism. To reduce the computational cost, the calculations were performed on a model reaction using carbon tethered enyne: methyl (*E*)-non-2-en-7-ynoate and (dppe)Co(I) (singlet or triplet) as the starting complex. Because the thermal corrections are based on the ideal gas model, this approach ignores the solvent suppression on the rotational and translational freedoms of solutes, resulting in overestimation of entropy contributions to the reaction free energies in solution.[17] To correct the entropy change in solution, we applied an empirical approach proposed by Martin and co-workers.[18] For each component change in a reaction at 298 Kand 1 atm, a correction of 4.3 kcal/mol is applied to the reaction free energy. This approach has been validated through a number of computational and experimental studies.[19]

Evaluation of Precursor Complexes. Among various cationic (dppe)Co–substrate complexes, the one with the alkene coordinated with the Co in the triplet state (Int1A-t) was calculated to be the most stable (**Figure S4**). The triplet enyne-binding complex (Int1-t) and alkyne-binding complex (Int1B-t) were less stable by 3.9 and 2.9 kcal mol^{-1}, respectively. The singlet counterparts (Int1-s, Int1A-s, and Int1B-s) were found to be much less stable.

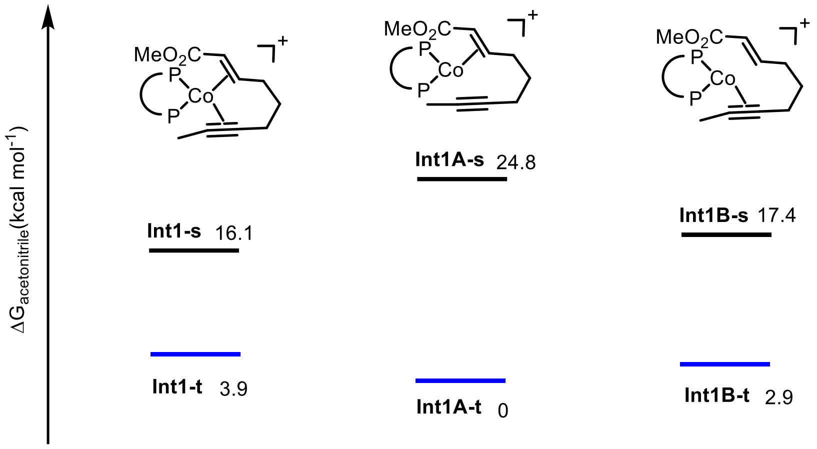

Supplementary Figure 4. Relative stability of cobalt–substrate complexes. The suffixes s and t of the structure numbers refer to the singlet and triplet states, respectively.

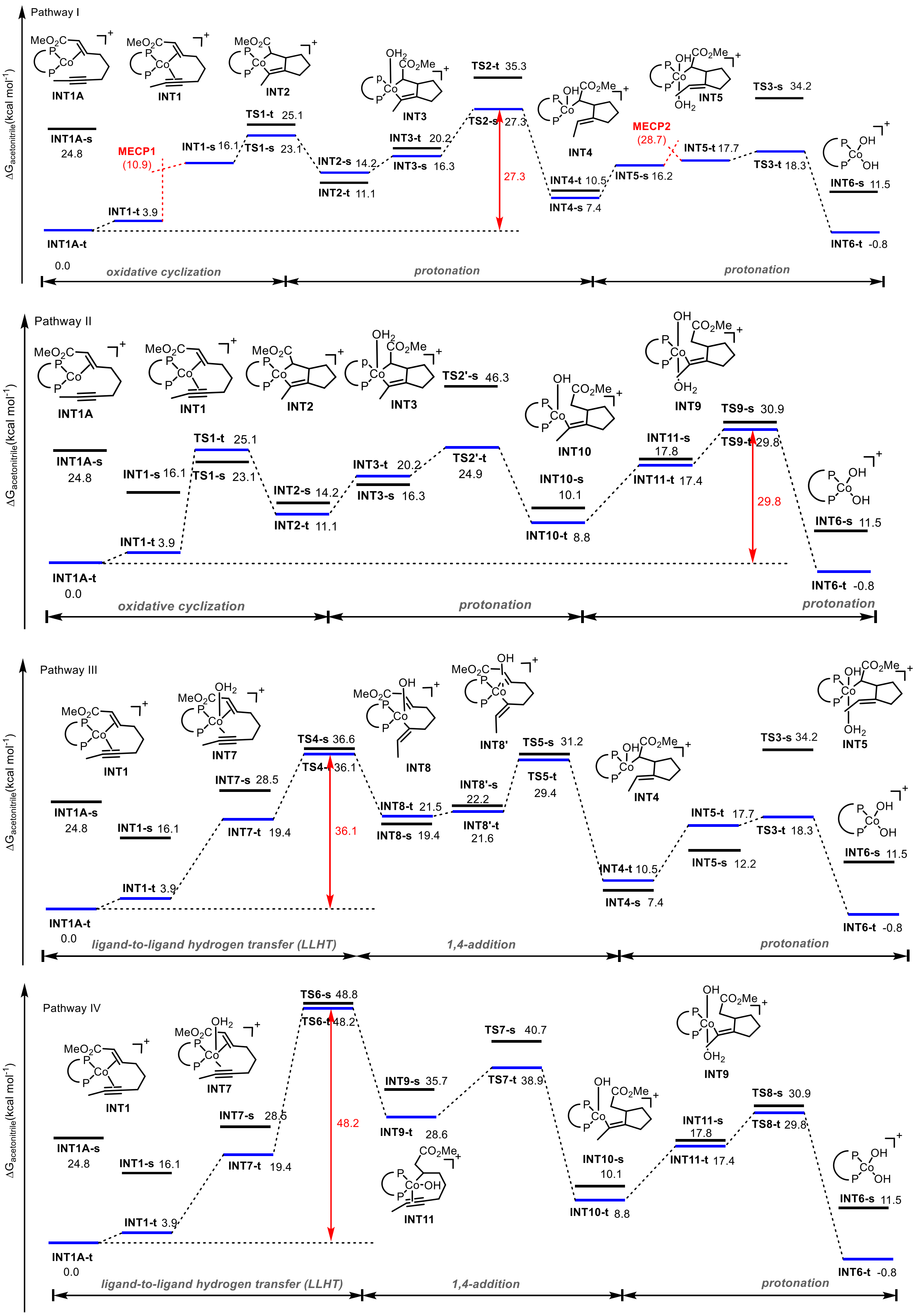

Supplementary Figure 5. Gibbs free energy diagram of Pathway I, Pathway II, Pathway III and Pathway IV.

INT1A-s

C	0.23654000	-1.19554600	3.01036700
C	-1.39034600	1.04644400	2.05648400
C	-1.18699700	0.82546300	3.53966200
C	-0.86689900	-0.62048200	3.91631200
H	-2.39782300	0.82761100	1.68802600
H	-2.08097300	1.15391400	4.08776800
H	-0.36747000	1.46777700	3.88713700
H	-1.75242600	-1.26451500	3.82937900
H	-0.54824900	-0.67489600	4.96451100
H	0.67796200	-2.11749500	3.41475600
H	-0.22234300	-1.52016400	2.03857800
Co	0.08624300	0.17859200	0.91405100
P	-1.27624600	-0.53259800	-0.65416400
P	1.68141300	-0.04355400	-0.62510600
C	-1.92331300	-2.17725700	-0.18614100
C	-1.90063300	-3.27224500	-1.05817900
C	-2.40512300	-4.50501800	-0.65156300
C	-2.94540800	-4.66028300	0.62210600
C	-2.98472300	-3.57493600	1.49446300
C	-2.47494300	-2.34553900	1.09290000
C	-2.76767000	0.38993400	-1.17081200
C	-3.96785300	0.27078600	-0.45771100
C	-5.10794300	0.94745500	-0.87738600
C	-5.06947000	1.73913700	-2.02233500
C	-3.87980100	1.86947000	-2.73408900
C	-2.73031200	1.21460800	-2.30354800
C	-0.33966800	-0.78187900	-2.22879400
C	0.83742400	0.18250400	-2.25254400
C	3.05279900	1.14810600	-0.63108400

C	2.75232400	2.49681000	-0.87494100
C	3.76271300	3.45279800	-0.83357000
C	5.07145400	3.07886800	-0.53268300
C	5.37128400	1.74280900	-0.27469200
C	4.36789300	0.77821900	-0.32162700
C	2.45541900	-1.68265300	-0.84578000
C	3.39624800	-1.90919000	-1.86160500
C	3.91619900	-3.18170900	-2.06746700
C	3.49903400	-4.24494300	-1.26689700
C	2.55973200	-4.03427900	-0.26164900
C	2.04196300	-2.75795400	-0.05290800
H	-1.50114400	-3.17247200	-2.06528800
H	-2.38033900	-5.34727700	-1.33969200
H	-3.33870700	-5.62533300	0.93389800
H	-3.41319100	-3.68522300	2.48884700
H	-2.50540800	-1.49826200	1.77906200
H	-4.02558400	-0.37876800	0.41446600
H	-6.03460300	0.83875200	-0.31784900
H	-5.96681900	2.25294700	-2.36085900
H	-3.84138800	2.49003400	-3.62731700
H	-1.80092400	1.35905000	-2.85258700
H	0.04253900	-1.81101400	-2.21995200
H	-0.99675300	-0.68616900	-3.10076500
H	0.50998700	1.22831500	-2.32160200
H	1.51630900	-0.02414000	-3.08957900
H	1.72141400	2.79583200	-1.07156200
H	3.52510200	4.49566500	-1.03397500
H	5.85861000	3.82929800	-0.49769200
H	6.39188400	1.44767000	-0.03907500
H	4.60878400	-0.26398400	-0.11571900

H	3.73156500	-1.08018000	-2.48600100
H	4.64936400	-3.34559800	-2.85445200
H	3.90810900	-5.23990100	-1.42936800
H	2.23079200	-4.86311700	0.36184700
H	1.29903700	-2.59146100	0.72971800
C	-0.64423800	1.98630000	1.30390700
C	1.25297600	-0.19261300	2.72735900
C	1.97183600	0.78583800	2.56544700
C	2.85446300	1.93363900	2.53462800
H	2.92888600	2.37557000	3.53585100
H	3.86331400	1.67015000	2.19792300
H	2.48151700	2.70652500	1.85079600
C	-1.15308900	2.81035700	0.17570300
O	-0.52294400	3.07374100	-0.83610400
O	-2.37336800	3.30484400	0.44049900
C	-2.86438700	4.27254100	-0.49767800
H	-2.74347500	3.91403000	-1.52170700
H	-3.91852900	4.40795400	-0.25485700
H	-2.31903400	5.21337000	-0.37389400
H	0.18951400	2.49375800	1.80379500

INT1A-t

C	-1.40642600	-1.08435900	3.70422600
C	-0.91284200	1.81952900	2.09075400
C	-1.51677600	1.43464200	3.41150200
C	-2.30712600	0.11979900	3.41391500
H	-1.61506300	2.16444000	1.32777300
H	-2.18723700	2.25818600	3.69614300
H	-0.73594800	1.39546100	4.18418000
H	-2.81147100	-0.01891600	2.44446500
H	-3.09991700	0.14902100	4.16976800

H	-1.16746400	-1.11340500	4.77761700
H	-1.94026600	-2.01977800	3.48558100
Co	-0.06118300	-0.07192700	1.15411100
P	-1.53502700	-0.11499600	-0.65271700
P	1.66464100	-0.28363600	-0.44016600
C	-2.70422800	-1.51194900	-0.58900200
C	-3.74898000	-1.64043000	-1.51359300
C	-4.59433200	-2.74321900	-1.46257200
C	-4.40947500	-3.72405500	-0.48873200
C	-3.37645200	-3.60333700	0.43703800
C	-2.52874600	-2.50045400	0.38653500
C	-2.57628800	1.30607200	-1.11806700
C	-3.69395000	1.62545600	-0.33268900
C	-4.48525600	2.72614700	-0.64432400
C	-4.17115700	3.52519700	-1.74231600
C	-3.06293000	3.21787000	-2.52601100
C	-2.26759900	2.11912900	-2.21282900
C	-0.48985400	-0.44595600	-2.14271500
C	0.86057700	0.25328200	-2.02348100
C	3.32338700	0.46415200	-0.50206000
C	3.50034700	1.76344600	-0.99085500
C	4.75598700	2.36062400	-0.97459900
C	5.85264600	1.66574500	-0.47422100
C	5.69161200	0.36715500	0.00532200
C	4.43683200	-0.23030000	-0.00524000
C	1.95797300	-2.04880600	-0.81976400
C	2.82982800	-2.42263600	-1.85269800
C	2.95882800	-3.75863200	-2.21423300
C	2.21525100	-4.73750200	-1.55530100
C	1.34716600	-4.37845600	-0.52875200

C	1.22190400	-3.04063600	-0.16160900
H	-3.90341500	-0.87054300	-2.26962400
H	-5.40211300	-2.83866100	-2.18507900
H	-5.07515300	-4.58372300	-0.45129200
H	-3.23379000	-4.36563100	1.20048000
H	-1.71714000	-2.39953000	1.11155800
H	-3.95979900	0.99262400	0.51606700
H	-5.35447800	2.95655900	-0.03204100
H	-4.79285400	4.38317100	-1.98784100
H	-2.81630000	3.83293800	-3.38959900
H	-1.39638400	1.90525700	-2.83075100
H	-0.34784100	-1.53457700	-2.18352100
H	-1.02221200	-0.16708000	-3.06089100
H	0.73567700	1.34370700	-1.98853900
H	1.51127100	0.01358300	-2.87487200
H	2.65175200	2.32292800	-1.37863400
H	4.87276700	3.37432800	-1.35143800
H	6.83454100	2.13389800	-0.46092800
H	6.54855100	-0.18627300	0.38375500
H	4.32501200	-1.25422100	0.35097700
H	3.41565000	-1.66214600	-2.36988500
H	3.63949700	-4.03777200	-3.01595100
H	2.31538700	-5.78171700	-1.84386900
H	0.76779200	-5.13962000	-0.00989100
H	0.54884400	-2.76212000	0.65195500
C	0.41826700	2.08455700	1.88374200
C	-0.12870400	-1.04915700	2.97019600
C	1.08420300	-1.08105300	2.69355600
C	2.52280000	-1.31605000	2.72251500
H	2.82729300	-1.70865900	3.70087400

H	2.81801200	-2.05006300	1.96084500
H	3.08759900	-0.39510200	2.53134300
C	0.90962300	2.96131300	0.80953300
O	1.94864000	3.58423800	0.86890300
O	0.03892000	3.05248100	-0.23370200
C	0.34543700	4.09097400	-1.17134900
H	-0.54373800	4.19324800	-1.79558000
H	0.56533000	5.02560700	-0.64907500
H	1.21303100	3.82352500	-1.78659200
H	1.14254500	1.92019400	2.68222400

INT1B-s

C	-0.42714000	-1.38061700	3.27161700
C	0.57982500	-0.43702800	2.73480100
C	-3.17839900	-1.11012000	0.55604100
C	-1.89288100	-1.47746600	1.20613100
C	-1.83285200	-1.19651600	2.70121600
C	1.59793800	0.35626900	2.85034000
C	2.64635900	1.04392700	3.62057300
H	2.20795000	1.62492200	4.44189100
H	-3.67729000	-0.20804500	0.92176300
H	-1.07957100	-0.88209800	0.73822300
H	-1.63228600	-2.52664400	0.99661800
H	-2.52380400	-1.85744500	3.24033700
H	-2.17600300	-0.16921200	2.89508100
H	-0.44370700	-1.29600800	4.36760800
H	-0.08100600	-2.40287700	3.04745400
H	3.32669600	0.30991300	4.07047100
H	3.24798100	1.72188700	3.00404200
Co	1.03537600	0.26176700	1.10153400
P	-0.07442500	1.42811100	-0.37272600

C	-1.73655100	2.00256700	0.06515400
C	-2.73703400	2.14946200	-0.90374400
C	-4.01254300	2.55740800	-0.52964600
C	-4.29971500	2.81807800	0.80812600
C	-3.30723500	2.67802000	1.77681700
C	-2.02941700	2.26894600	1.40864300
C	0.99982200	2.85773300	-0.72060100
C	1.52885200	3.55191300	0.37948200
C	2.38652100	4.62846300	0.18496300
C	2.73514800	5.01642600	-1.10738100
C	2.21773400	4.33156600	-2.20404400
C	1.35348700	3.25690600	-2.01481200
C	-0.23648200	0.50823400	-1.96455400
C	1.08994300	-0.20419800	-2.21230400
C	3.45881900	-1.13176100	-0.74508200
C	4.26701200	-0.03426800	-0.41426700
C	5.64876300	-0.12562100	-0.53645200
C	6.23030100	-1.31331200	-0.97780400
C	5.43187400	-2.40856500	-1.29980700
C	4.04764700	-2.32295600	-1.18470200
C	0.92834300	-2.58458100	-0.48698000
C	-0.02379800	-3.06317000	-1.39354300
C	-0.62588100	-4.30287600	-1.18627700
C	-0.28550200	-5.06997800	-0.07518900
C	0.66445800	-4.59976600	0.83219400
C	1.26293900	-3.36151800	0.63330100
H	-2.52766100	1.94013500	-1.95186400
H	-4.78602900	2.66543100	-1.28664100
H	-5.30187300	3.12758400	1.09784300
H	-3.53110000	2.88121200	2.82200800

H	-1.25515700	2.13674500	2.16693200
H	1.25629700	3.24753500	1.39180200
H	2.78442700	5.16574200	1.04331400
H	3.40982100	5.85593100	-1.26031200
H	2.48403100	4.63617400	-3.21386900
H	0.95210100	2.73940100	-2.88529200
H	-1.05556600	-0.21210400	-1.81878000
H	-0.52275800	1.15020900	-2.80731800
H	1.86610000	0.51589200	-2.50510400
H	1.03276900	-0.94887800	-3.01559300
H	3.80743300	0.89108600	-0.05632500
H	6.27342200	0.72726900	-0.28040200
H	7.31194400	-1.38639300	-1.06729800
H	5.88764100	-3.33476200	-1.64307600
H	3.42405900	-3.18077700	-1.43410100
H	-0.30542900	-2.47686500	-2.26714700
H	-1.36143200	-4.66940200	-1.89941600
H	-0.75756500	-6.03708300	0.08288200
H	0.93890500	-5.20051200	1.69689000
H	1.99939100	-2.98882000	1.34709400
C	-3.73221500	-1.77883200	-0.46536300
P	1.66343400	-0.93332500	-0.60856000
C	-4.97028500	-1.39287300	-1.15629000
O	-5.39029700	-1.94503700	-2.15370500
O	-5.59625400	-0.34908300	-0.55973400
C	-6.82101100	0.03327400	-1.18759800
H	-7.21081000	0.86225900	-0.59430000
H	-6.64645800	0.34519500	-2.22249100
H	-7.52825800	-0.80120600	-1.19150100
H	-3.26614800	-2.68094300	-0.86377300

INT1B-t

C	1.83716600	1.42203900	2.84919400
C	0.71085600	0.47626000	2.94857300
C	3.97895100	-0.37117800	0.14046500
C	2.77570300	0.05667800	0.90333900
C	3.08153100	0.78809600	2.20878700
C	0.01952300	-0.48419500	3.40137400
C	-0.41195700	-1.49988100	4.37544200
H	-0.62607100	-2.46188300	3.89567600
H	4.73958400	-0.94365000	0.67547300
H	2.15444300	-0.82665800	1.13993800
H	2.14947800	0.69535300	0.26180000
H	3.80988700	1.58897700	2.02555100
H	3.55299300	0.09203500	2.91549900
H	2.08673300	1.78524300	3.85617100
H	1.54182100	2.30503600	2.26704400
H	0.35806000	-1.66022100	5.13986900
H	-1.32798500	-1.18245600	4.88638600
Co	-0.65735900	-0.05507300	1.62820100
P	-1.06496800	-1.48431800	-0.13649800
C	-0.60446700	-3.23037500	0.06975800
C	-1.20385400	-4.24817800	-0.68189400
C	-0.79562900	-5.56765900	-0.51503100
C	0.21192600	-5.88004400	0.39479900
C	0.81409300	-4.87199100	1.14490500
C	0.40277200	-3.55262800	0.98910100
C	-2.73644600	-1.48291500	-0.84425000
C	-3.78553500	-1.97524100	-0.05296900
C	-5.09740200	-1.91247100	-0.50609700
C	-5.38024700	-1.34139600	-1.74639800

C	-4.34643200	-0.84699200	-2.53586300
C	-3.02918100	-0.91813600	-2.08953000
C	0.08140900	-0.86158400	-1.44877400
C	0.07380200	0.66293100	-1.62824400
C	-2.17298100	2.04036600	-0.44780900
C	-3.20018100	1.61210300	0.39959200
C	-4.52837200	1.91636300	0.11519200
C	-4.83698300	2.65979100	-1.02083400
C	-3.82040300	3.10207100	-1.86704500
C	-2.49455100	2.79530300	-1.58339400
C	0.45947100	3.12848300	-0.04324900
C	1.76083100	3.27879800	-0.54048800
C	2.45097400	4.47378500	-0.35574700
C	1.85571300	5.52789100	0.33167200
C	0.56328100	5.38675800	0.83360700
C	-0.12991600	4.19659500	0.65106200
H	-1.99350500	-4.00811200	-1.39310400
H	-1.26689800	-6.35547500	-1.09872400
H	0.52693500	-6.91352100	0.52263700
H	1.59863800	-5.11530800	1.85839700
H	0.86099100	-2.76157500	1.58817200
H	-3.56970400	-2.41995900	0.92008300
H	-5.90185900	-2.30812800	0.11055400
H	-6.40823000	-1.28620800	-2.09823500
H	-4.56179900	-0.40308400	-3.50571900
H	-2.23192300	-0.53254700	-2.72362400
H	1.07456400	-1.19599000	-1.11703100
H	-0.11034700	-1.38019100	-2.39751100
H	-0.60623100	0.96853500	-2.43272300
H	1.07171400	1.00333600	-1.92942200

H	-2.96267100	1.02527700	1.29373600
H	-5.31931700	1.56909700	0.77670700
H	-5.87373700	2.90036000	-1.24660700
H	-4.06297300	3.69188500	-2.74847100
H	-1.70193100	3.15431100	-2.24112100
H	2.25252700	2.46803900	-1.07701400
H	3.45632800	4.57970700	-0.75766000
H	2.39677800	6.46095700	0.47309100
H	0.09021800	6.20871300	1.36645600
H	-1.14183000	4.09483200	1.04371800
C	4.17120000	-0.09576900	-1.15556500
P	-0.45327900	1.55496400	-0.08275800
C	5.33374400	-0.50118500	-1.96248000
O	5.45573100	-0.23229800	-3.14065800
O	6.24078500	-1.20047400	-1.24783700
C	7.39390900	-1.60139400	-1.99488200
H	8.01204400	-2.16958500	-1.29969600
H	7.10217700	-2.21847700	-2.84952400
H	7.93413700	-0.72440700	-2.36338100
H	3.43331100	0.47860500	-1.71992200

H_2O

O	0.00000000	0.00000000	0.11917400
H	0.00000000	0.75798200	-0.47669400
H	0.00000000	-0.75798200	-0.47669400

INT1-s

C	10.16405400	1.29058200	-3.87549000
C	11.07783400	1.84637800	-4.89015600
C	10.93314700	4.36353900	-3.71087800
C	10.44789100	3.44674600	-2.62374800
C	9.45779800	2.38196100	-3.07666900

C	11.70481000	1.71495700	-6.02809400
C	11.92034400	0.82741800	-7.18336500
H	12.43636200	1.31163700	-8.01947400
H	10.17919000	5.04813200	-4.11216600
H	9.97384900	4.07689700	-1.85599600
H	11.30832100	2.96472600	-2.13368300
H	8.67620900	2.85121000	-3.69526200
H	8.94380300	1.94045500	-2.21518400
H	10.75479400	0.66281700	-3.19013900
H	9.44177000	0.60970600	-4.35206600
H	12.51547500	-0.04735700	-6.89007500
H	10.95154900	0.45090500	-7.54186100
Co	11.87145700	3.45166800	-5.38497200
P	10.72759300	4.48062800	-6.95075600
P	13.71522000	4.19038500	-6.48679800
C	9.54199000	3.33393700	-7.74733800
C	9.40697300	3.30010300	-9.14058500
C	8.48977200	2.44062000	-9.74084800
C	7.68484800	1.61656100	-8.96088700
C	7.79408100	1.65893500	-7.57214100
C	8.71744700	2.50730200	-6.97289400
C	9.68967500	5.94002300	-6.62180800
C	10.09238000	7.22697200	-6.99681400
C	9.26642000	8.31764100	-6.75463300
C	8.03937100	8.14087300	-6.11943000
C	7.63442400	6.86564000	-5.73167700
C	8.45278200	5.77047600	-5.98333200
C	11.87552900	4.95523700	-8.31964400
C	13.20261200	5.42454000	-7.74636700
C	15.06538600	4.97351600	-5.54500900

C	15.60180500	6.20898100	-5.92453900
C	16.63248500	6.78356400	-5.18746500
C	17.14849200	6.12888700	-4.07332600
C	16.62462700	4.89605400	-3.68909900
C	15.58132800	4.32733600	-4.41240900
C	14.55983300	2.86491600	-7.42190900
C	14.68879600	2.88011500	-8.81492500
C	15.30436400	1.82054300	-9.47916900
C	15.80358800	0.73858400	-8.76165500
C	15.68507500	0.71590900	-7.37290800
C	15.06029600	1.76517500	-6.71055800
H	10.00996200	3.94696900	-9.77430800
H	8.40506800	2.42067600	-10.82520000
H	6.97074700	0.94496100	-9.43208300
H	7.16237000	1.02492900	-6.95303300
H	8.80872200	2.52163900	-5.88742400
H	11.06260800	7.38676200	-7.46131600
H	9.58757000	9.31184900	-7.05719000
H	7.39794800	8.99797700	-5.92634900
H	6.67390700	6.72053700	-5.24204400
H	8.11623600	4.77327600	-5.69843400
H	12.02088500	4.04128900	-8.91317800
H	11.41619200	5.70321200	-8.97804900
H	13.08002600	6.37143600	-7.21061000
H	13.96012300	5.56809400	-8.52627300
H	15.21676500	6.73781900	-6.79365300
H	17.03503400	7.74758900	-5.49053100
H	17.95902000	6.57823900	-3.50376800
H	17.02566600	4.37734400	-2.82011000
H	15.16587100	3.36981700	-4.09365800

H	14.31569700	3.71880600	-9.40049800
H	15.39711500	1.84830000	-10.56282800
H	16.28682000	-0.08522000	-9.28206100
H	16.07545000	-0.12581900	-6.80438900
H	14.94997700	1.72295900	-5.62649800
C	12.27054300	4.77633700	-3.79572300
C	12.62730400	6.16628000	-4.16790400
H	12.97869500	4.25626700	-3.14440500
O	12.26445600	6.71966400	-5.19039200
O	13.39589300	6.86703600	-3.31157800
C	13.70760700	6.35360800	-2.01658800
H	14.43066900	5.53168500	-2.08144300
H	14.16001000	7.18629900	-1.47610500
H	12.80624200	6.02181500	-1.48863600

INT1-t

C	12.08450300	0.00726500	-4.75836000
C	11.65285700	1.01750500	-5.73652700
C	10.85666300	2.62797100	-3.49674900
C	11.54604500	1.49238900	-2.80017200
C	11.35672900	0.11903200	-3.42362700
C	11.26809200	1.43922900	-6.86584500
C	10.96083400	1.16706200	-8.27873800
H	11.88051500	1.22144100	-8.87765600
H	9.77377000	2.54209100	-3.62607900
H	11.15122400	1.47494400	-1.77270500
H	12.61961600	1.71803900	-2.70440200
H	10.28632600	-0.08538600	-3.56577300
H	11.73462900	-0.65223700	-2.74302800
H	13.16855900	0.11511800	-4.59690900
H	11.93577500	-0.98815200	-5.19940000

H	10.56752800	0.14822900	-8.38070900
H	10.22950600	1.85401800	-8.71505200
Co	11.48290200	2.97130900	-5.55460400
P	10.56824100	4.46450000	-7.15326200
P	13.58088300	3.92704600	-6.30442300
C	9.04253300	3.91774400	-7.97030500
C	8.77758900	4.14866000	-9.32652900
C	7.59458900	3.68731500	-9.89689000
C	6.66391400	3.00240500	-9.11827500
C	6.91450100	2.77784100	-7.76633000
C	8.10064600	3.22864500	-7.19539900
C	10.28534300	6.23772400	-6.81173700
C	9.14709200	6.91414200	-7.27273700
C	8.98884700	8.27309000	-7.01770500
C	9.95635200	8.97390000	-6.30000100
C	11.08510500	8.30865400	-5.82883300
C	11.24458700	6.95054700	-6.08195400
C	11.86690000	4.52799700	-8.47346200
C	13.22212100	4.87897000	-7.86160300
C	14.78435000	4.97907500	-5.42635400
C	14.36149500	5.81327600	-4.38182600
C	15.26888500	6.66062400	-3.75070200
C	16.60279000	6.67742700	-4.14806800
C	17.03211800	5.84786200	-5.18285700
C	16.13082400	5.00293900	-5.81971700
C	14.55914300	2.48946400	-6.84154500
C	14.52261900	2.00439700	-8.15275400
C	15.18839700	0.82785500	-8.49016300
C	15.89825900	0.12355100	-7.52203900
C	15.94471500	0.60075000	-6.21278500

C	15.27403000	1.76992800	-5.87245800
H	9.49397800	4.69275500	-9.94192600
H	7.39815400	3.86594100	-10.95203100
H	5.74058100	2.64221000	-9.56684000
H	6.18783200	2.24338600	-7.15794900
H	8.30930100	3.03929400	-6.14053200
H	8.37834900	6.38041800	-7.82895000
H	8.10113600	8.78669700	-7.38155900
H	9.82485700	10.03522000	-6.09998000
H	11.83740600	8.83712100	-5.24668600
H	12.11299400	6.43444100	-5.67461500
H	11.89191700	3.53834100	-8.94897700
H	11.60337400	5.25894600	-9.24845500
H	13.23893700	5.93874900	-7.57847700
H	14.03688200	4.74470400	-8.58552500
H	13.32145300	5.81631700	-4.05410300
H	14.92860900	7.30157800	-2.94003400
H	17.31128800	7.33582200	-3.64968600
H	18.07406100	5.85897600	-5.49558000
H	16.47344100	4.35206300	-6.62398200
H	13.97883000	2.54813800	-8.92592800
H	15.15977900	0.46753800	-9.51670900
H	16.42333800	-0.79124700	-7.78819600
H	16.50882500	0.06146600	-5.45430600
H	15.31880600	2.14183600	-4.84700000
C	11.42409600	3.89270200	-3.56768600
H	12.44033500	4.02740200	-3.19645000
C	10.63606600	5.13935800	-3.58472400
O	11.06724500	6.20104900	-3.17973600
O	9.38166200	4.95577600	-4.05439900

C	8.52916900	6.10851900	-3.97356300
H	9.06313300	7.00436100	-4.30102700
H	7.68356200	5.89641500	-4.62905800
H	8.18992500	6.24290700	-2.94189400

INT2-s

C	-2.07143900	-2.15441000	-1.14375700
C	-1.23497400	-1.37913900	-0.15399100
C	-2.07803600	-0.32859600	0.49952000
C	-3.50714200	-0.87753200	0.33948100
C	-3.46054800	-1.50124100	-1.05722200
C	0.03564100	-1.57700800	0.27810100
C	1.02899400	-2.46942500	-0.39027100
H	1.70977900	-2.96012900	0.31935400
C	-1.54885900	-0.08276300	1.88448600
H	-2.01744300	0.60088300	-0.09077800
H	-4.27600900	-0.10663100	0.45701400
H	-3.69102900	-1.65253600	1.09931600
H	-3.54882900	-0.70897400	-1.81262100
H	-4.27707300	-2.20780100	-1.23871200
H	-2.11269300	-3.21542200	-0.85730500
H	-1.64775700	-2.13641100	-2.15740700
H	0.54013700	-3.25372300	-0.98741300
H	1.67353600	-1.89573200	-1.07390000
Co	0.23061100	-0.88682800	2.01592900
P	0.72115300	-0.59022900	4.22494400
P	1.54574400	0.74838300	1.59055900
C	-0.09299900	-0.93498800	5.81226500
C	-1.10460200	-0.07665800	6.26945300
C	-1.74076400	-0.33786200	7.47777100
C	-1.39039900	-1.45632100	8.23138600

C	-0.39492500	-2.31628400	7.77593300
C	0.25421900	-2.05997200	6.57231300
C	2.05890600	-1.81867600	4.03877000
C	3.37477200	-1.63253300	4.48565900
C	4.36632200	-2.55275200	4.16347000
C	4.06228700	-3.67077900	3.38352800
C	2.76027800	-3.87595100	2.93675400
C	1.76402100	-2.95615000	3.26365100
C	1.60460800	1.01744800	4.40990800
C	2.40854000	1.29235600	3.14430700
C	2.93039800	0.29253200	0.49867800
C	3.68450400	-0.83692600	0.84981600
C	4.74568900	-1.24965700	0.05387900
C	5.06396900	-0.54193400	-1.10432400
C	4.32167200	0.58177700	-1.45718600
C	3.25765900	1.00062500	-0.66228400
C	0.74305600	2.18112100	0.82788700
C	0.67159600	3.43539100	1.44338800
C	0.05619800	4.49862400	0.78667400
C	-0.49716800	4.31751500	-0.47824300
C	-0.44419300	3.06605900	-1.08971400
C	0.16465700	2.00062600	-0.43781600
H	-1.39052900	0.78902400	5.67593200
H	-2.51833000	0.33624800	7.83148600
H	-1.89444200	-1.65770700	9.17439500
H	-0.11632800	-3.18977300	8.36149600
H	1.03816700	-2.73371100	6.22960300
H	3.62399000	-0.76336100	5.09410900
H	5.38176500	-2.40189100	4.52422400
H	4.84273300	-4.38650000	3.13347100

H	2.51468600	-4.75237900	2.34059000
H	0.73405200	-3.14068100	2.93953100
H	0.82076700	1.76748100	4.56027300
H	2.24740300	1.02841300	5.29915500
H	3.33752300	0.70708400	3.15023700
H	2.71202600	2.34317900	3.05903000
H	3.43289700	-1.40492900	1.74722400
H	5.32155800	-2.12900800	0.33678000
H	5.89331800	-0.86508100	-1.72983600
H	4.57401000	1.14289400	-2.35437300
H	2.69271300	1.88662700	-0.94367100
H	1.09053800	3.59204000	2.43630700
H	0.01152500	5.47362000	1.26804500
H	-0.97350300	5.15229200	-0.98796000
H	-0.87963800	2.91955200	-2.07589800
H	0.20547300	1.01620700	-0.90863100
C	-1.76832800	1.24775400	2.51416200
O	-1.31052500	1.60651800	3.59015600
O	-2.56662700	2.03439700	1.77091400
C	-2.90345700	3.29730200	2.35914700
H	-3.44091900	3.84576100	1.58539600
H	-3.54076300	3.14426100	3.23504600
H	-2.00038800	3.83365400	2.66133300
H	-1.90099800	-0.86459300	2.58713900

INT2-t

C	-2.25943100	-2.32548100	-1.18898700
C	-1.65111200	-1.43304100	-0.13964400
C	-2.72526000	-0.67884000	0.56911700
C	-3.91206600	-1.64583200	0.48607300
C	-3.77948900	-2.19606800	-0.94002600

C	-0.37978100	-1.35120500	0.32275800
C	0.80574500	-2.03330900	-0.27881900
H	1.56890400	-2.30784800	0.46347500
C	-2.15470600	-0.26320200	1.90468600
H	-2.98263300	0.22747100	-0.00660200
H	-4.88271000	-1.17666400	0.67948200
H	-3.77968700	-2.45213500	1.22295700
H	-4.20979600	-1.47601400	-1.64828400
H	-4.31513600	-3.13952600	-1.08637500
H	-1.91028300	-3.35992700	-1.05801500
H	-1.97746700	-2.04372200	-2.21267100
H	0.50689900	-2.95717300	-0.79760900
H	1.31713600	-1.39307500	-1.01292000
Co	-0.14438400	-0.42458200	1.99344100
P	0.77579700	-0.67387200	4.16924700
P	1.66670600	0.98235200	1.62122700
C	-0.10283200	-1.04301700	5.71349900
C	-1.19034200	-0.23769500	6.08510300
C	-1.89774200	-0.52183200	7.24796100
C	-1.54059200	-1.61314600	8.03835200
C	-0.46817000	-2.41953000	7.66765700
C	0.25047300	-2.13917300	6.50947000
C	2.14610400	-1.86318300	4.02794900
C	3.38510800	-1.69335800	4.66094800
C	4.42272900	-2.59157100	4.42600000
C	4.23377300	-3.66999700	3.56234200
C	3.00180400	-3.85829900	2.94005500
C	1.96544900	-2.95802000	3.17081000
C	1.59882800	0.95805700	4.44287400
C	2.47685500	1.33387500	3.25041700

C	3.02276600	0.44859700	0.53424000
C	3.83612100	-0.61777800	0.94704300
C	4.81736300	-1.11822700	0.10029800
C	4.99603000	-0.56566700	-1.16715400
C	4.19384400	0.49433800	-1.58124900
C	3.20792900	0.99985100	-0.73855900
C	1.01382400	2.51940900	0.90927900
C	1.39941800	3.79791900	1.32571200
C	0.88048700	4.92504200	0.69285200
C	-0.01673500	4.78629000	-0.36366300
C	-0.40688400	3.51601100	-0.78379100
C	0.09679300	2.38753600	-0.14570400
H	-1.47740900	0.60847900	5.46108100
H	-2.73646100	0.10875600	7.53589100
H	-2.10137600	-1.83577200	8.94365700
H	-0.18655200	-3.27179100	8.28234700
H	1.08951000	-2.77309500	6.22659100
H	3.54312100	-0.85586400	5.34103400
H	5.38204300	-2.45002600	4.91940500
H	5.04828200	-4.36797300	3.37969900
H	2.84846300	-4.70228400	2.27052300
H	1.00172300	-3.10143800	2.67746600
H	0.78665200	1.68143100	4.57875700
H	2.18516900	0.95659400	5.37056200
H	3.39758900	0.73595500	3.25516500
H	2.79750400	2.38069800	3.30967400
H	3.69793200	-1.06891500	1.93081500
H	5.44214300	-1.94557300	0.43247200
H	5.76247200	-0.96039200	-1.83063300
H	4.33642000	0.93518400	-2.56549800

H	2.58683400	1.82991000	-1.07132600
H	2.11316400	3.92661600	2.13782800
H	1.18680000	5.91541600	1.02285200
H	-0.41415100	5.67022500	-0.85840100
H	-1.11250700	3.40215400	-1.60437300
H	-0.21543400	1.39039100	-0.46555100
C	-2.01200300	1.12111100	2.31749200
O	-1.17422000	1.42232700	3.19583800
O	-2.70848700	2.03469200	1.64541600
C	-2.46311200	3.40396100	2.01027000
H	-2.98378400	4.00031400	1.26147200
H	-2.87044000	3.59932800	3.00623800
H	-1.39048900	3.61626600	2.00345200
H	-2.34661100	-0.93535700	2.75059400

INT3-s

C	-2.15816100	-1.76031900	-1.40774900
C	-1.33049900	-1.21312100	-0.26489400
C	-2.14037100	-0.20161100	0.48732100
C	-3.57283400	-0.71997900	0.29685000
C	-3.56354500	-1.16677300	-1.16953600
C	-0.12099400	-1.54920000	0.21454700
C	0.82744500	-2.52830200	-0.39834900
H	1.38694900	-3.10544900	0.35571600
C	-1.60030600	-0.04678800	1.89720600
H	-2.07981700	0.76664900	-0.03503100
H	-4.34328000	0.02557700	0.52343400
H	-3.74479900	-1.58404200	0.96172900
H	-3.71048000	-0.28872700	-1.81176700
H	-4.37045000	-1.86778700	-1.40790900
H	-2.17107600	-2.86015600	-1.39613000

H	-1.75669300	-1.48269600	-2.39224800
H	0.30992200	-3.25175800	-1.04754900
H	1.58763200	-2.02776200	-1.01564200
Co	0.15985900	-0.99398100	2.04116800
P	0.89020400	-0.69647300	4.21926600
P	1.45341200	0.67254300	1.52024400
C	0.01001200	-1.07582800	5.76623800
C	-1.09074700	-0.28936600	6.14301900
C	-1.80964600	-0.60557300	7.29079900
C	-1.45593400	-1.71116300	8.06221400
C	-0.37190100	-2.50000400	7.68716100
C	0.36055000	-2.18702700	6.54561500
C	2.35641600	-1.79509300	4.19524900
C	3.61024100	-1.39577000	4.67848300
C	4.71828300	-2.22685600	4.54812400
C	4.59220900	-3.47380200	3.93735600
C	3.35076600	-3.89054100	3.46409700
C	2.24143200	-3.05715300	3.58722300
C	1.62241000	0.98753300	4.33952500
C	2.36390600	1.26159500	3.03558400
C	2.82917900	0.17417900	0.42835900
C	3.58637800	-0.94284700	0.81163500
C	4.64269600	-1.38354100	0.02393800
C	4.95578100	-0.71430700	-1.15821600
C	4.21482900	0.40047000	-1.54034000
C	3.15572100	0.84581900	-0.75349100
C	0.68651100	2.11463400	0.73069900
C	0.60364400	3.36458700	1.35381000
C	0.00271900	4.43473800	0.69532500
C	-0.52432500	4.26662300	-0.58204300

C	-0.45465700	3.02189200	-1.20496900
C	0.14155300	1.94991000	-0.55188500
H	-1.37502500	0.56889000	5.53684600
H	-2.65408600	0.01547800	7.58313000
H	-2.02511800	-1.95698500	8.95625200
H	-0.08810100	-3.36131600	8.28822600
H	1.21419000	-2.80325400	6.26811200
H	3.72955700	-0.42749300	5.16248300
H	5.68388900	-1.90058000	4.92873100
H	5.46077900	-4.12131600	3.83749700
H	3.24221300	-4.86666600	2.99548300
H	1.27474500	-3.39504300	3.21434900
H	0.78997400	1.68055200	4.48610100
H	2.29089100	1.08410200	5.20353600
H	3.30589100	0.69704800	3.01562600
H	2.63936200	2.31729600	2.92383400
H	3.34410800	-1.47602800	1.73231400
H	5.21907400	-2.25321400	0.33476100
H	5.78136300	-1.05846600	-1.77754700
H	4.46483200	0.93488200	-2.45448900
H	2.59512200	1.72615100	-1.05871800
H	0.99352500	3.51015300	2.36019300
H	-0.05286500	5.40452500	1.18625300
H	-0.99035300	5.10625600	-1.09344100
H	-0.86752800	2.88474800	-2.20227300
H	0.18714800	0.97029800	-1.03001600
C	-1.72873200	1.29543200	2.53341800
O	-1.22129500	1.61683700	3.59899800
O	-2.52628400	2.12555200	1.83849400
C	-2.81048300	3.37965400	2.47081400

H	-3.33074400	3.97467000	1.71990100
H	-3.44912400	3.22057700	3.34497400
H	-1.88721100	3.87096600	2.78710000
H	-2.09220200	-0.74564100	2.59464100
O	-0.81743500	-2.81644200	2.48997500
H	-1.35680200	-2.91937800	1.67978100
H	-1.43470000	-2.78920600	3.24081700

INT3-t

C	-2.38777800	-2.00974200	-1.38177300
C	-1.72920500	-1.23759600	-0.26509500
C	-2.76388900	-0.50647500	0.52795000
C	-3.96793700	-1.44833200	0.40974300
C	-3.89671500	-1.89229000	-1.05676600
C	-0.46904000	-1.26684400	0.21016100
C	0.69307100	-1.99709900	-0.37566400
H	1.38463100	-2.38349800	0.38950400
C	-2.14942500	-0.21393500	1.88127700
H	-3.02890300	0.44439800	0.03384800
H	-4.92569100	-0.98861400	0.67771900
H	-3.82421900	-2.31277900	1.08022000
H	-4.35100700	-1.11843200	-1.68883700
H	-4.44736600	-2.81822200	-1.25221500
H	-2.04890300	-3.05588900	-1.39292200
H	-2.15075400	-1.61110000	-2.37740700
H	0.35418300	-2.85979000	-0.96995400
H	1.29868200	-1.35885100	-1.03488700
Co	-0.14391100	-0.63874900	2.04861700
P	0.87868700	-0.73469700	4.19304000
P	1.59348300	0.85240400	1.55974800
C	-0.04074700	-1.13218600	5.70721200

C	-1.14314600	-0.34134900	6.06819300
C	-1.89234300	-0.66682700	7.19379300
C	-1.56467600	-1.78661500	7.95713300
C	-0.48066900	-2.58140400	7.59491100
C	0.28039800	-2.25920600	6.47474200
C	2.34206900	-1.82364700	4.17397400
C	3.49214500	-1.52406100	4.91799600
C	4.61604100	-2.33922600	4.83753800
C	4.60300200	-3.46844100	4.01943500
C	3.46147100	-3.78315200	3.28628900
C	2.33657700	-2.96451800	3.35814800
C	1.59393600	0.95143500	4.39565000
C	2.42918300	1.28988900	3.16199800
C	2.97347500	0.36003100	0.47897300
C	3.82077100	-0.67399800	0.90356600
C	4.82828200	-1.14360500	0.07032800
C	5.00097100	-0.58980200	-1.19734800
C	4.16547800	0.43900500	-1.62416100
C	3.15245100	0.91236400	-0.79446800
C	0.92994800	2.37522800	0.81893900
C	1.41888000	3.65138700	1.12413500
C	0.92081200	4.76951900	0.46106500
C	-0.06034100	4.62484500	-0.51830300
C	-0.54987200	3.35863300	-0.82988500
C	-0.06317600	2.23914800	-0.16164000
H	-1.40806900	0.52646000	5.46477300
H	-2.73900300	-0.04399000	7.47545000
H	-2.15603200	-2.03928400	8.83458700
H	-0.21998900	-3.45479300	8.18897200
H	1.13164200	-2.88132800	6.20229400

H	3.51262400	-0.64976900	5.56804000
H	5.50322300	-2.09502200	5.41794000
H	5.48319200	-4.10516000	3.95933800
H	3.44576700	-4.66733800	2.65226300
H	1.44322500	-3.21170700	2.78520400
H	0.75223400	1.64222000	4.50469100
H	2.20100000	1.02876400	5.30579700
H	3.36442200	0.71456100	3.17183400
H	2.72368900	2.34561000	3.16588500
H	3.68910100	-1.12259900	1.88856900
H	5.47824200	-1.94652700	0.41429800
H	5.78867700	-0.95863400	-1.85088100
H	4.30273400	0.88060700	-2.60891200
H	2.50436300	1.71680500	-1.13920100
H	2.20008100	3.78376100	1.87071000
H	1.30763200	5.75660500	0.70569700
H	-0.44248300	5.50127200	-1.03788800
H	-1.31839800	3.23994500	-1.59128300
H	-0.45020800	1.24586800	-0.39695200
C	-1.95398500	1.14630100	2.35752100
O	-1.12259000	1.38572200	3.25329200
O	-2.62766000	2.11037900	1.73059800
C	-2.31929800	3.45292800	2.14044200
H	-2.90853100	4.09655900	1.48776400
H	-2.60261700	3.60081900	3.18613400
H	-1.24979200	3.65167600	2.01892000
H	-2.44883400	-0.87695400	2.70418100
O	-0.79576000	-3.06645600	2.48868600
H	-1.39656000	-3.04819200	1.72455700
H	-1.36222900	-3.21043200	3.26250400

INT4-s

C	-3.43204700	-2.12756200	2.75900400
C	-2.51409700	-1.66191100	1.66707700
C	-2.88660600	-0.21535500	1.35552000
C	-4.30332200	-0.03242400	1.96562900
C	-4.74445100	-1.43732300	2.37665200
C	-1.60535900	-2.40103000	0.98727400
C	-1.28478300	-3.83129700	1.27507200
H	-0.20378900	-4.01810600	1.29102300
C	-1.85902900	0.67974200	2.01564300
H	-2.87907900	-0.04058400	0.27580200
H	-4.98948800	0.45155400	1.26293600
H	-4.24810000	0.61595600	2.85205800
H	-5.20345100	-1.95961600	1.52696700
H	-5.47527400	-1.43593700	3.19199800
H	-3.10006800	-1.74418000	3.73760100
H	-3.50597500	-3.21625000	2.84590600
H	-1.68909700	-4.17082500	2.23427900
H	-1.69239400	-4.48354000	0.49064600
Co	-0.20221300	-0.24241100	1.44812200
P	0.52722500	-0.87486100	3.47685400
P	1.27992200	1.33375500	1.62731600
C	-0.61547700	-1.45791400	4.76004300
C	-1.38806100	-0.53376200	5.47834600
C	-2.34556400	-0.97635800	6.38618800
C	-2.54228100	-2.34219700	6.58252000
C	-1.77841600	-3.26539000	5.87181500
C	-0.82208800	-2.82873700	4.96009100
C	1.77847700	-2.18085300	3.29674700
C	2.55842100	-2.59822500	4.38372900

C	3.54992500	-3.55574800	4.20375900
C	3.76904800	-4.10718000	2.94108500
C	2.99570000	-3.70267100	1.85692800
C	2.00668800	-2.73895000	2.03527900
C	1.45385400	0.54011100	4.26420600
C	1.39645200	1.79529100	3.39464900
C	2.82488900	0.49140900	1.17013400
C	3.95298400	0.51516400	1.99981700
C	5.09579000	-0.19385200	1.64337100
C	5.12931300	-0.91339600	0.45073900
C	4.01619000	-0.92810000	-0.38668400
C	2.86130900	-0.24268100	-0.02597400
C	1.21002300	2.86153700	0.65835500
C	1.15954300	4.11664800	1.27916400
C	1.17296000	5.27447000	0.50677200
C	1.21685300	5.19065300	-0.88272900
C	1.24993300	3.94414000	-1.50617000
C	1.25063300	2.78297600	-0.74199300
H	-1.24419700	0.53823200	5.33218700
H	-2.93575800	-0.25251700	6.94406600
H	-3.28891400	-2.68726500	7.29434000
H	-1.92525000	-4.33204800	6.02896900
H	-0.22566200	-3.55446100	4.40793400
H	2.38252000	-2.17803200	5.37466500
H	4.15234500	-3.87604600	5.05104200
H	4.54482500	-4.85758000	2.80463600
H	3.16672100	-4.12755800	0.86982600
H	1.41216800	-2.40593700	1.18038800
H	1.05057300	0.73759200	5.26372800
H	2.48771200	0.20234600	4.40044200

H	2.24401900	2.46587900	3.58711100
H	0.47650300	2.36308000	3.58983000
H	3.95086500	1.09406900	2.92263200
H	5.96591900	-0.17640900	2.29593700
H	6.02599500	-1.46442400	0.17483800
H	4.04007700	-1.48916300	-1.31891600
H	1.96994600	-0.29483900	-0.65285600
H	1.09573200	4.19786800	2.36139600
H	1.14180000	6.24539400	0.99635900
H	1.22527900	6.09852600	-1.48189500
H	1.28479700	3.87502000	-2.59105700
H	1.29820300	1.81150000	-1.23255500
C	-1.82945700	2.11579600	1.65226100
O	-1.45732900	3.00305500	2.40548500
O	-2.23852300	2.34708400	0.38697500
C	-2.28546500	3.73022600	0.00468100
H	-2.63106000	3.73447600	-1.02900600
H	-2.98355900	4.27390900	0.64723900
H	-1.29518900	4.18701000	0.08620200
H	-1.91271500	0.61019400	3.10973500
O	-0.43049200	-0.00864700	-0.34910800
H	-0.64626800	0.90298800	-0.60675000
H	-1.18381100	-1.97504800	0.07271000

INT4-t

C	-2.83146700	-2.62066000	1.94008000
C	-2.50255900	-1.39999300	1.12955000
C	-3.02877400	-0.15794100	1.84424500
C	-3.79932400	-0.71767100	3.05672600
C	-4.08327900	-2.17775500	2.70382200
C	-2.00702400	-1.34346400	-0.13526200

C	-1.63780600	-2.50078000	-0.98705300
H	-0.64530900	-2.35520200	-1.42497600
C	-1.83229300	0.72353700	2.17352200
H	-3.70791400	0.37633100	1.16698900
H	-4.70518200	-0.14241300	3.27396600
H	-3.17029200	-0.66912600	3.95497200
H	-4.95905000	-2.25137000	2.04516500
H	-4.28594200	-2.79750800	3.58390200
H	-2.02253400	-2.84159800	2.65514600
H	-2.97086700	-3.51972400	1.32981500
H	-1.62332200	-3.44031100	-0.42557900
H	-2.35637200	-2.60483900	-1.81103400
Co	-0.31608200	-0.34717500	1.24362900
P	0.42574400	-1.14561800	3.49977000
P	1.27792100	1.26243600	1.53236600
C	-0.70729000	-1.34649800	4.90817700
C	-1.13189000	-0.23587600	5.65198400
C	-2.06165100	-0.38864000	6.67609400
C	-2.58217200	-1.64790300	6.96484400
C	-2.16227600	-2.75806100	6.23405000
C	-1.23116400	-2.61130700	5.21139200
C	1.43940500	-2.65262000	3.51078100
C	2.34713100	-2.90771700	4.54855800
C	3.12380300	-4.05998600	4.52592300
C	2.99173600	-4.97009400	3.47705800
C	2.08343100	-4.72812200	2.45002000
C	1.30933300	-3.56961300	2.46025700
C	1.56156300	0.22675800	4.06026400
C	1.32379800	1.55966600	3.34411400
C	2.98861600	0.75169100	1.12818800

C	3.91146900	1.66831600	0.60390400
C	5.22748800	1.28134800	0.36938600
C	5.64155400	-0.01878400	0.64965600
C	4.73375600	-0.93828700	1.16785700
C	3.41623900	-0.55751900	1.40237900
C	1.07229600	2.81796800	0.63763100
C	1.15064700	4.07576100	1.24455700
C	1.08043800	5.22654100	0.46381100
C	0.92845200	5.12936100	-0.91804000
C	0.83223200	3.87801100	-1.52573400
C	0.89916800	2.72548800	-0.75120500
H	-0.74075600	0.75862900	5.43293900
H	-2.37767400	0.47973100	7.24987700
H	-3.30970600	-1.76594400	7.76470400
H	-2.55499000	-3.74586600	6.46657900
H	-0.89245600	-3.48678500	4.65678200
H	2.44315900	-2.20658800	5.37795200
H	3.83098700	-4.25156500	5.33022400
H	3.60035600	-5.87175800	3.46286900
H	1.98107700	-5.43868700	1.63250600
H	0.62798500	-3.35233200	1.63709100
H	1.47164200	0.35435300	5.14589600
H	2.58606400	-0.11829200	3.86929100
H	2.12123400	2.27000000	3.59929200
H	0.36331200	2.02120800	3.61461200
H	3.60397000	2.68807900	0.37867300
H	5.93279000	2.00261400	-0.03742500
H	6.67073100	-0.31529300	0.45925200
H	5.04332700	-1.95865500	1.38635900
H	2.71534500	-1.29276100	1.79512200

H	1.25300900	4.16090600	2.32438300
H	1.14400500	6.20285200	0.93915300
H	0.88020100	6.03209600	-1.52319600
H	0.70503800	3.79984600	-2.60321200
H	0.82793600	1.74506600	-1.22624800
C	-1.85244900	2.13361600	1.74923900
O	-1.42958400	3.06134300	2.42165100
O	-2.36600200	2.29053500	0.50678800
C	-2.44661200	3.64881600	0.05352400
H	-2.69700300	3.59138900	-1.00601900
H	-3.22977700	4.18029400	0.60266800
H	-1.49237400	4.16218800	0.20310800
H	-1.57913300	0.69270800	3.23787300
O	0.70095300	-1.35272100	0.10334600
H	1.61832900	-1.05030000	-0.00028700
H	-2.00934300	-0.35931300	-0.62399700

INT5-s

C	-3.59946800	-2.92896700	-0.94740100
C	-2.63198000	-2.67882900	0.18799800
C	-1.22453200	-2.91362900	-0.32668900
C	-1.36898500	-2.81158600	-1.84865500
C	-2.74498200	-3.40111600	-2.12890300
C	-2.97819400	-2.35046500	1.43790000
C	-4.37851600	-2.15657800	1.91395800
H	-4.51782000	-1.17645400	2.39625300
C	-0.05436300	-2.10778800	0.19616300
H	-0.98848800	-3.97417800	-0.10737300
H	-0.55335800	-3.31769700	-2.38263300
H	-1.34284300	-1.74999100	-2.13909900
H	-2.67841500	-4.49728700	-2.13712700

H	-3.15585900	-3.09972400	-3.09930400
H	-4.12803000	-2.00004900	-1.20045100
H	-4.37852200	-3.65434500	-0.67928300
H	-5.10694800	-2.22515600	1.09807700
H	-4.65800700	-2.90277500	2.67066900
Co	0.34285500	-0.15287400	0.94237800
P	-0.90503300	1.22240100	-0.35203900
P	1.96154000	0.12607900	-0.54124200
C	-2.52616500	0.67860400	-1.00287500
C	-2.74280500	0.38929400	-2.35467700
C	-4.03398300	0.18941000	-2.83895200
C	-5.12293100	0.26206500	-1.97590400
C	-4.91624600	0.51049800	-0.61892500
C	-3.62902500	0.71724500	-0.13907900
C	-1.35869200	2.88569400	0.26684900
C	-2.19248900	3.68331500	-0.53619600
C	-2.54787200	4.96110800	-0.12467000
C	-2.09012700	5.45522600	1.09645200
C	-1.26677600	4.67038800	1.89613900
C	-0.89486000	3.38990200	1.48776200
C	0.11885300	1.57971500	-1.85650000
C	1.07702500	0.42759300	-2.12721900
C	3.06464800	1.56425000	-0.38727200
C	4.34197800	1.52313700	-0.96814800
C	5.14306700	2.65975300	-0.97170000
C	4.68029000	3.84514600	-0.40460500
C	3.41409500	3.89239400	0.17247500
C	2.60717500	2.75850400	0.18758300
C	3.06490700	-1.28807500	-0.77590500
C	3.01856200	-2.09888200	-1.91651800

C	3.89430200	-3.17430600	-2.04283000
C	4.81612800	-3.44607900	-1.03548900
C	4.86329100	-2.64500100	0.10507200
C	3.98968600	-1.57381800	0.23864700
H	-1.90724800	0.33513400	-3.05109600
H	-4.18534100	-0.01488300	-3.89689400
H	-6.13171600	0.11961300	-2.35712700
H	-5.76105900	0.55942000	0.06509100
H	-3.48833300	0.96953300	0.91278500
H	-2.57636400	3.29907700	-1.48140800
H	-3.19089400	5.57072200	-0.75587900
H	-2.37904500	6.45270900	1.42157200
H	-0.90642300	5.05223100	2.84927900
H	-0.22647800	2.79111900	2.10241400
H	-0.51674500	1.81376400	-2.71774500
H	0.68315200	2.49419300	-1.63084300
H	1.78299500	0.66265600	-2.93423300
H	0.53020000	-0.48768400	-2.39903900
H	4.71282500	0.60382900	-1.41869800
H	6.13294100	2.61717800	-1.42102900
H	5.31065000	4.73182800	-0.40883900
H	3.05067900	4.81400200	0.62249500
H	1.63728400	2.78072100	0.67877700
H	2.30667700	-1.88974200	-2.71477200
H	3.85990100	-3.79549100	-2.93497600
H	5.50230800	-4.28379200	-1.13982300
H	5.58456700	-2.85536800	0.89162400
H	4.02580400	-0.94939000	1.13068800
C	0.51132300	-2.40104400	1.49357900
O	1.34969300	-1.56502200	1.94800100

O	0.07511700	-3.41637300	2.20971400
C	0.54081500	-3.49092000	3.57201900
H	0.03130100	-4.35232400	4.00111200
H	1.62404700	-3.63298800	3.59263200
H	0.27638700	-2.57321900	4.10342500
H	0.76272700	-2.16035100	-0.53106200
O	0.97349900	1.18810300	2.19158400
H	1.78551300	0.81438000	2.56807200
H	-2.19500100	-2.24130600	2.18709700
O	-0.96723600	-0.29611600	2.56657600
H	-0.37873400	0.45383900	2.89996100
H	-1.86046700	0.05696000	2.43380900

INT5-t

C	6.50928000	-0.62232900	-0.40441600
C	5.14346100	-0.44910000	-1.02696800
C	4.23393800	-1.56654900	-0.54493300
C	4.98670400	-2.11496400	0.69431600
C	6.44914200	-1.98616800	0.28731300
C	4.76599700	0.53792600	-1.84823700
C	5.62195000	1.65809700	-2.32628700
H	5.18001200	2.63694100	-2.08899400
C	2.84398500	-1.13682600	-0.27481900
H	4.22401700	-2.37951400	-1.28995700
H	4.67203500	-3.13246000	0.95035100
H	4.77276600	-1.46868000	1.55865500
H	6.70394900	-2.78165300	-0.42607900
H	7.14171700	-2.07430600	1.13098400
H	6.68865100	0.17230800	0.33790100
H	7.32441800	-0.54418300	-1.13437600
H	6.62131700	1.63133300	-1.87890500

H	5.74646300	1.63864100	-3.41750800
Co	-0.62291300	-0.11661900	-0.81650600
P	-0.32287000	1.49561100	0.76239700
P	-2.31509400	-0.78276400	0.40182400
C	1.33357200	1.60244800	1.49379800
C	1.68886900	0.77167000	2.56482200
C	3.00126100	0.74080900	3.02504400
C	3.97632000	1.52527100	2.41235400
C	3.63551000	2.34694400	1.33995400
C	2.32265200	2.38647600	0.88046100
C	-0.70553800	3.15051500	0.12913400
C	-0.44960000	4.31326800	0.86941400
C	-0.80050700	5.55507900	0.35263000
C	-1.40319200	5.64686000	-0.90237600
C	-1.65596300	4.49709100	-1.64542800
C	-1.30682300	3.25081300	-1.13266800
C	-1.49269800	1.14080500	2.15725000
C	-1.87036200	-0.33902000	2.13000300
C	-3.87203400	0.08559100	0.06018600
C	-4.87065600	0.17622900	1.04047800
C	-6.04962700	0.86155000	0.77077100
C	-6.24253400	1.45435600	-0.47637200
C	-5.25912500	1.35504900	-1.45677500
C	-4.07299600	0.67492400	-1.19457900
C	-2.62906400	-2.56039700	0.34983100
C	-2.42010400	-3.38404100	1.46250300
C	-2.64847200	-4.75367900	1.36506600
C	-3.07902100	-5.30581100	0.16114300
C	-3.28373200	-4.48813200	-0.94967800
C	-3.05841300	-3.11966700	-0.86259600

H	0.94049800	0.13796700	3.04091100
H	3.26384400	0.09913800	3.86360000
H	5.00260700	1.49727500	2.77263500
H	4.39416000	2.96354400	0.86162300
H	2.06060100	3.04567800	0.05126000
H	0.02910200	4.24369700	1.84643900
H	-0.60227500	6.45579200	0.92955700
H	-1.67547300	6.62160700	-1.30169100
H	-2.12371100	4.57019000	-2.62488800
H	-1.51046300	2.34182900	-1.70587700
H	-1.06055200	1.44008200	3.12008500
H	-2.38200400	1.76330300	1.99172000
H	-2.67710800	-0.56315000	2.83993300
H	-1.01026200	-0.96685600	2.39951800
H	-4.73475500	-0.29458900	2.01442300
H	-6.82075800	0.93190600	1.53491400
H	-7.16645800	1.99030900	-0.68386400
H	-5.41610000	1.80669800	-2.43418500
H	-3.30470000	0.56886800	-1.96019900
H	-2.08407700	-2.96612100	2.41024100
H	-2.49227400	-5.38931600	2.23423100
H	-3.25879300	-6.37653600	0.08907500
H	-3.62013800	-4.91888900	-1.89033200
H	-3.18731900	-2.48102500	-1.73460900
C	1.71547600	-1.97564500	-0.29273000
O	0.53962000	-1.60529100	0.00853300
O	1.94175700	-3.25311000	-0.65681400
C	0.78365800	-4.08724700	-0.76016000
H	1.14466900	-5.03165500	-1.16775300
H	0.33103700	-4.24663300	0.22408300

H	0.03673100	-3.63796100	-1.42361400
H	2.69430400	-0.15462700	0.17434500
O	-1.48349000	-0.73024100	-2.32070900
H	-0.81591000	-0.65452000	-3.02379700
H	3.73845800	0.52411000	-2.22962100
O	0.95702000	0.45957300	-2.06634100
H	1.17096600	1.40583500	-2.06396400
H	1.76178900	0.01099200	-1.71600300

INT6-s

C	-4.87113900	1.23047800	-1.58323200
C	-4.26129400	1.71520000	-0.28474900
C	-3.80057800	0.50606400	0.51143300
C	-3.79505300	-0.62565800	-0.51854400
C	-4.96810300	-0.29028200	-1.42942600
C	-4.15918600	2.98716900	0.11449300
C	-4.67108900	4.17189400	-0.62888100
H	-3.89754600	4.94321700	-0.74496000
C	-2.46038600	0.67900100	1.21433700
H	-4.55940900	0.27738900	1.27733500
H	-3.84132900	-1.62851100	-0.07328400
H	-2.85430900	-0.57240600	-1.09400500
H	-5.91057200	-0.55992800	-0.93358900
H	-4.94300100	-0.82641500	-2.38478400
H	-4.20824600	1.48285100	-2.42815700
H	-5.83712300	1.69935700	-1.80600500
H	-5.03686500	3.91073800	-1.62834300
H	-5.49843100	4.65495200	-0.09109600
Co	0.51559400	-0.00733500	1.06796800
P	1.64539400	1.22938300	-0.36294600
P	0.58089400	-1.64100700	-0.53966900

C	0.61849800	2.69611900	-0.67491400
C	-0.49908100	2.62491700	-1.51808700
C	-1.32848100	3.72910300	-1.68165700
C	-1.05192400	4.91384800	-1.00454800
C	0.04857700	4.99003200	-0.15348600
C	0.87631900	3.88711900	0.01848900
C	3.30092100	1.85162100	0.00950900
C	3.88291900	2.79945600	-0.84753800
C	5.16719500	3.26302100	-0.59415000
C	5.87323300	2.79156700	0.51283700
C	5.29734800	1.85356600	1.36489600
C	4.01133700	1.37666000	1.11841500
C	1.77104100	0.34632300	-1.99006100
C	0.68961100	-0.71986300	-2.13628000
C	2.06324800	-2.69337700	-0.58447100
C	2.00039100	-3.99424800	-1.10105900
C	3.16140900	-4.74983200	-1.22971400
C	4.39042400	-4.21653600	-0.84907500
C	4.45909100	-2.92415400	-0.33538900
C	3.30218500	-2.16457300	-0.19801500
C	-0.82551800	-2.77167200	-0.70048600
C	-1.55428300	-2.90312600	-1.88822900
C	-2.62680500	-3.78807700	-1.95833400
C	-2.97402400	-4.55297900	-0.84785600
C	-2.24452900	-4.43708300	0.33505000
C	-1.17658500	-3.54976600	0.41303600
H	-0.72845800	1.70553000	-2.05763100
H	-2.19361500	3.66300200	-2.33887200
H	-1.69288400	5.78235900	-1.14249400
H	0.26826400	5.91522000	0.37480400

H	1.74366400	3.96086800	0.67374000
H	3.32570000	3.18381400	-1.70228300
H	5.61719200	3.99717400	-1.25879300
H	6.87743800	3.16092300	0.71024300
H	5.84993100	1.48949500	2.22836700
H	3.55006900	0.62656600	1.76211300
H	1.74643900	1.07487200	-2.81036600
H	2.76344000	-0.12337800	-2.00446900
H	0.91727800	-1.39344500	-2.97218100
H	-0.29288400	-0.26937000	-2.33392300
H	1.04425400	-4.41996300	-1.40264400
H	3.10325000	-5.76068700	-1.62776100
H	5.29551600	-4.81242900	-0.94693300
H	5.41537200	-2.50718700	-0.02610800
H	3.35376200	-1.17141800	0.24369800
H	-1.28969100	-2.31692200	-2.76709100
H	-3.18732100	-3.88408800	-2.88609000
H	-3.80999200	-5.24690800	-0.90573500
H	-2.50539900	-5.04714500	1.19782200
H	-0.60323100	-3.45785500	1.33543100
C	-2.07263500	-0.46744000	2.09975100
O	-0.95083800	-0.98697500	2.14059100
O	-3.05902200	-0.89907800	2.86291200
C	-2.74468000	-1.96875600	3.77556200
H	-3.65072700	-2.12103500	4.35973200
H	-2.48791300	-2.87075600	3.21387600
H	-1.90638800	-1.68225400	4.41430600
H	-1.66098200	0.82266700	0.46452800
O	1.86373900	-0.84195500	1.98326700
H	1.69028200	-0.46527900	2.86694100

H	-3.68570400	3.19917400	1.07810400
O	0.26478600	1.32261100	2.29909700
H	0.91413800	2.04142900	2.25333800
H	-2.42887600	1.58694600	1.83262200

INT6-t

C	6.03176200	-1.00745900	1.08890000
C	5.41354400	-0.57709700	-0.22287100
C	4.18167000	-1.42182700	-0.48008500
C	3.84803100	-2.00143100	0.90140700
C	5.21917200	-2.22976600	1.52909700
C	5.84000100	0.42137400	-1.00350400
C	7.03490700	1.27178100	-0.74273800
H	6.78105500	2.34162500	-0.75557900
C	3.03921000	-0.63658000	-1.13502600
H	4.44587500	-2.26325800	-1.14324200
H	3.23189200	-2.90961900	0.84788900
H	3.27809200	-1.25576000	1.47921600
H	5.66410700	-3.14472000	1.11499100
H	5.18463800	-2.35624300	2.61662000
H	5.93913300	-0.19817300	1.83200000
H	7.10536000	-1.21877400	1.00846900
H	7.49784100	1.05007400	0.22573000
H	7.80549600	1.13793700	-1.51409100
Co	-0.80156600	0.06712700	-1.12426100
P	-0.51020600	1.68210500	0.49026000
P	-2.02186600	-0.98144600	0.51319600
C	1.23258600	1.98148500	0.85745900
C	1.87345500	1.32726400	1.91594800
C	3.24017700	1.49414600	2.11801200
C	3.97919000	2.30345600	1.25911300

C	3.34765100	2.95817000	0.20231700
C	1.98304300	2.79588400	-0.00385700
C	-1.23193900	3.27089000	0.01378000
C	-0.88050600	4.46139700	0.66519600
C	-1.48460100	5.65744900	0.29514500
C	-2.43595700	5.67590900	-0.72405600
C	-2.78622200	4.49781200	-1.37880000
C	-2.18537900	3.29751500	-1.01247600
C	-1.30973700	1.13668900	2.06565100
C	-1.34313300	-0.38932300	2.11541000
C	-3.77563500	-0.52519200	0.53275000
C	-4.55218800	-0.74838100	1.67906200
C	-5.89486400	-0.38939700	1.69075200
C	-6.47297100	0.18784500	0.56080900
C	-5.70992000	0.40029100	-0.58378100
C	-4.36301200	0.04667500	-0.60216900
C	-1.91936700	-2.77976200	0.47819400
C	-1.02254000	-3.48441300	1.29156800
C	-0.93239600	-4.86880100	1.18947800
C	-1.71867400	-5.55443000	0.26537400
C	-2.60117100	-4.85513700	-0.55625400
C	-2.70603500	-3.47279100	-0.45319500
H	1.31090200	0.67937600	2.58738200
H	3.72910700	0.98378700	2.94614500
H	5.04997200	2.42399900	1.41169900
H	3.92385200	3.59044800	-0.47014400
H	1.49539800	3.30304400	-0.83539500
H	-0.12868300	4.45098100	1.45403300
H	-1.21038100	6.57979100	0.80257900
H	-2.90232900	6.61549600	-1.01254700

H	-3.52263400	4.51430100	-2.17913400
H	-2.45565100	2.37211200	-1.52773700
H	-0.80052500	1.57181800	2.93460500
H	-2.33237300	1.53704600	2.05677500
H	-1.92669100	-0.75339600	2.97053800
H	-0.32966300	-0.80144700	2.20832600
H	-4.11319700	-1.21495600	2.56128100
H	-6.49329100	-0.56640900	2.58176300
H	-7.52464200	0.46555600	0.57256300
H	-6.16547000	0.83669200	-1.47033800
H	-3.76389400	0.16975700	-1.50384000
H	-0.38940700	-2.95999000	2.00545000
H	-0.24554300	-5.41335300	1.83403100
H	-1.64415100	-6.63683800	0.18661000
H	-3.21399700	-5.38885100	-1.27941700
H	-3.38821000	-2.92512800	-1.10098200
C	1.76708200	-1.41372900	-1.13364800
O	0.82167800	-1.19726100	-0.36061400
O	1.76821100	-2.41204400	-1.99696100
C	0.59944100	-3.26114900	-2.01289700
H	0.78805400	-3.98457500	-2.80528200
H	0.49833000	-3.76343400	-1.04533900
H	-0.29642200	-2.66532500	-2.21871700
H	2.86882000	0.29557600	-0.58742000
O	-1.80505800	-0.96094200	-2.29303800
H	-1.58700500	-0.54729600	-3.14692100
H	5.27876200	0.65054100	-1.91394100
O	0.33688100	0.85451600	-2.31649900
H	0.08528300	1.76548700	-2.53853900
H	3.29381300	-0.38058000	-2.17035500

INT7-s

C	0.49993700	1.22297100	3.74519000
C	0.10802700	1.35300000	2.32771400
C	0.74984600	-1.36429300	2.03670100
C	0.57776800	-1.29066400	3.52456700
C	1.24977600	-0.05473100	4.10494000
C	-0.30826200	1.99536000	1.31877800
C	-0.43872000	3.29300600	0.62642100
H	-1.33128900	3.84022100	0.95360700
H	1.80357400	-1.31502100	1.74454800
H	1.04488400	-2.18686900	3.96541200
H	-0.48353200	-1.32889700	3.80350500
H	2.28382000	-0.00228800	3.72657200
H	1.32930200	-0.12667400	5.19572500
H	-0.41478600	1.27158800	4.35743300
H	1.08924500	2.10865900	4.02925800
H	0.43645200	3.91792200	0.84824300
H	-0.51761900	3.19514100	-0.46313600
Co	-0.30420800	-0.00011200	0.88603900
P	1.33874700	0.38939100	-0.54417000
P	-1.68872600	0.01296100	-0.95212100
C	2.36967800	1.85436200	-0.18549800
C	2.68654100	2.78392200	-1.18170100
C	3.49185800	3.88295800	-0.88973700
C	3.99664600	4.05995500	0.39420500
C	3.70098100	3.12997300	1.39001900
C	2.89025700	2.03890700	1.10251000
C	2.60834400	-0.88615200	-0.87421400
C	3.64771600	-1.07261000	0.04967900
C	4.63601100	-2.02318400	-0.17812900

C	4.60155200	-2.80068600	-1.33331400
C	3.57851700	-2.62008000	-2.25953700
C	2.58620700	-1.67193600	-2.03258400
C	0.58173200	0.75248100	-2.19324000
C	-0.63756800	-0.12832200	-2.44862700
C	-3.02983700	-1.23005800	-1.01928800
C	-2.91982900	-2.43155200	-1.73041200
C	-3.94328400	-3.37369300	-1.67630200
C	-5.08021300	-3.14632500	-0.90505200
C	-5.20550500	-1.95268700	-0.20082700
C	-4.19260100	-0.99988600	-0.26305900
C	-2.62890900	1.57306000	-1.19566300
C	-2.79427100	2.17446900	-2.44921500
C	-3.54397400	3.34026700	-2.58737700
C	-4.15865400	3.91692000	-1.48031000
C	-4.01105900	3.32797200	-0.22687200
C	-3.24353200	2.17785900	-0.09074700
H	2.31395400	2.66053300	-2.19659000
H	3.72430200	4.60099800	-1.67328600
H	4.62179100	4.92071600	0.62118200
H	4.09734200	3.25937800	2.39525300
H	2.63859500	1.32869500	1.89065700
H	3.70682600	-0.44816300	0.94067700
H	5.44125800	-2.14691600	0.54319500
H	5.37571500	-3.54314000	-1.51423100
H	3.54659800	-3.22244900	-3.16440900
H	1.78471200	-1.56140600	-2.75933100
H	0.27787100	1.80923400	-2.16022900
H	1.33191500	0.65949500	-2.98896300
H	-0.35507700	-1.18036200	-2.54352600

H	-1.15873700	0.16213700	-3.36734400
H	-2.03261800	-2.64340300	-2.31740200
H	-3.84690800	-4.29603800	-2.24542500
H	-5.87165300	-3.89137600	-0.86369500
H	-6.10079500	-1.74959700	0.38315400
H	-4.34683800	-0.03799300	0.22766100
H	-2.34395500	1.73822800	-3.33845300
H	-3.65385400	3.79317200	-3.57033500
H	-4.74903300	4.82359900	-1.59180700
H	-4.48171900	3.77326300	0.64763700
H	-3.09081000	1.75495500	0.90265800
C	-0.09490900	-2.03409300	1.12556100
O	-2.07096800	-0.32811200	2.14008500
H	-2.34223600	0.56888700	2.40027100
H	-2.82317200	-0.70583000	1.64251800
C	0.39413500	-2.86129800	-0.01480000
O	-0.05182500	-2.78375500	-1.14480000
O	1.35005600	-3.78876300	0.19777800
C	1.82490500	-4.10949500	1.50358800
H	1.01614500	-4.11513800	2.24158300
H	2.25265800	-5.11012300	1.41888800
H	2.60713000	-3.40784600	1.81595500
H	-1.06766300	-2.38355400	1.48704200

INT7-t

C	-0.60624800	1.09373000	3.86081800
C	-0.33424000	1.41015600	2.45263300
C	0.76767900	-1.36556700	2.33393200
C	0.22319200	-1.29306600	3.73469600
C	0.34882500	0.05039700	4.43752000
C	-0.13758500	2.06554500	1.39980600

C	-0.02752200	3.28120300	0.58450700
H	-0.89084400	3.92761400	0.78750600
H	1.84443700	-1.19197900	2.23113400
H	0.76664500	-2.04979500	4.32322800
H	-0.82907500	-1.60937000	3.72438700
H	1.38288700	0.41988400	4.37051700
H	0.13098500	-0.07176500	5.50519100
H	-1.64059600	0.73102600	3.95072900
H	-0.54747500	2.02688700	4.43825600
H	0.88619200	3.83531600	0.83005100
H	-0.01477700	3.08273800	-0.49278600
Co	-0.10067200	0.02939000	1.01613200
P	1.49224400	0.40017000	-0.76891100
P	-1.69687400	-0.07717400	-0.78824300
C	2.54734100	1.88002200	-0.60694600
C	2.89512600	2.68492200	-1.69706900
C	3.72644100	3.78702000	-1.51827600
C	4.23166900	4.08659400	-0.25522500
C	3.90053500	3.28435200	0.83482700
C	3.05634600	2.19266800	0.66069200
C	2.74363100	-0.86949300	-1.16743200
C	3.81069500	-1.02695300	-0.27168700
C	4.78392200	-1.99374300	-0.49404100
C	4.69758800	-2.82365400	-1.60964300
C	3.63990700	-2.67620200	-2.50220500
C	2.66564600	-1.70610400	-2.28423900
C	0.50949400	0.66935800	-2.31245800
C	-0.74743900	-0.20178200	-2.36407800
C	-3.11073600	-1.23392700	-0.97096500
C	-2.87885200	-2.59025500	-1.25088100

C	-3.94383500	-3.48159200	-1.33828200
C	-5.25070000	-3.04693500	-1.13108700
C	-5.49030200	-1.70652900	-0.84339800
C	-4.43262100	-0.80375700	-0.76583300
C	-2.49461300	1.56510600	-0.83855200
C	-2.52055100	2.38599800	-1.97113400
C	-3.12935300	3.63890100	-1.92070200
C	-3.73206500	4.07800200	-0.74535000
C	-3.72544300	3.26160600	0.38557200
C	-3.10082600	2.02038800	0.34365400
H	2.52258600	2.45155700	-2.69407700
H	3.98741400	4.40922400	-2.37177200
H	4.88524300	4.94581900	-0.12074000
H	4.29228000	3.51575600	1.82337800
H	2.76371300	1.58867200	1.52254300
H	3.89718700	-0.36773600	0.59379100
H	5.61509500	-2.09457100	0.20179000
H	5.45734100	-3.58205700	-1.78590300
H	3.56749900	-3.32170500	-3.37484300
H	1.83373700	-1.61976500	-2.98133300
H	0.22902800	1.73348100	-2.30497500
H	1.12798300	0.51438900	-3.20637900
H	-0.48155300	-1.25884000	-2.46967200
H	-1.37415900	0.06200500	-3.22544800
H	-1.86195800	-2.94643500	-1.39416500
H	-3.74611400	-4.52662200	-1.56847600
H	-6.07906100	-3.74917200	-1.19679600
H	-6.50771600	-1.35171400	-0.69012700
H	-4.64418300	0.24381800	-0.56121400
H	-2.07077600	2.05353700	-2.90593500

H	-3.13872200	4.26921500	-2.80738900
H	-4.21066500	5.05456200	-0.71050200
H	-4.20227100	3.59822700	1.30443500
H	-3.07809400	1.38340600	1.23034800
C	0.10872900	-2.06078800	1.31827400
O	-2.86271700	-0.90312400	2.25560500
H	-3.55761200	-0.95448100	2.92675200
H	-3.29513700	-1.22313500	1.44601800
C	0.70551900	-2.77981400	0.16180000
O	0.19008700	-2.77996000	-0.94261100
O	1.82171800	-3.51263300	0.31478300
C	2.42154300	-3.78123100	1.57987600
H	1.67468700	-3.90983600	2.36963800
H	2.97673500	-4.71144200	1.44449100
H	3.12311200	-2.98327400	1.84875700
H	-0.92450000	-2.36364000	1.51435900

INT8-s

C	-1.83281200	-0.09731700	-0.60151500
C	-1.53642300	-0.80561200	0.68148400
C	-3.29409600	0.37414200	-3.12019900
C	-3.41125600	1.31997800	-1.97634400
C	-3.17460000	0.63391300	-0.62795700
C	-2.18694500	-1.89260600	1.13332900
C	-3.20851400	-2.66796000	0.35512200
H	-3.26818500	-2.35733500	-0.69447400
H	-3.92272400	-0.52235700	-3.10608000
H	-4.41271600	1.77573500	-1.96953300
H	-2.69403800	2.14480600	-2.10406200
H	-3.98188800	-0.08473600	-0.42748700
H	-3.21860900	1.37912100	0.17846500

H	-1.81076400	-0.83374900	-1.42399500
H	-1.04016800	0.62112500	-0.86456800
H	-2.97754900	-3.74106000	0.37460200
H	-4.21008100	-2.55964400	0.79368300
Co	-0.04925900	-0.39686900	1.77238400
P	0.54690100	1.58364900	1.23372600
C	-0.77879700	2.79710200	1.06688900
C	-1.64337700	2.98290800	2.15569600
C	-2.69461000	3.88544900	2.05943900
C	-2.89994500	4.59562500	0.87625600
C	-2.04801800	4.40882400	-0.20880500
C	-0.98782100	3.51067200	-0.11855300
C	1.68307300	2.10673500	2.55514400
C	1.38494900	1.76063000	3.88298100
C	2.23142400	2.16521200	4.91113100
C	3.37811100	2.90207700	4.62791300
C	3.68202200	3.24152500	3.31069000
C	2.84186600	2.84685200	2.27611100
C	1.55063200	1.57600900	-0.30836600
C	2.48544100	0.37270700	-0.22139000
C	2.61931200	-2.28994700	1.01702400
C	2.89853500	-2.15742200	2.38505100
C	3.78884400	-3.02669300	3.00475200
C	4.39851700	-4.03721700	2.26362800
C	4.12039400	-4.17782300	0.90540200
C	3.23222700	-3.30963800	0.27895600
C	0.84500100	-1.82185200	-1.25872600
C	0.91447600	-1.14726600	-2.48346200
C	0.26734800	-1.65759500	-3.60539000
C	-0.46111600	-2.84064900	-3.51446900

C	-0.52051500	-3.52803500	-2.30208100
C	0.12284300	-3.02250100	-1.17885500
H	-1.49005200	2.40783000	3.06981100
H	-3.36044700	4.03207100	2.90694300
H	-3.72728600	5.29781100	0.80135700
H	-2.20654200	4.96375800	-1.13121000
H	-0.32654800	3.37735300	-0.97407000
H	0.49274400	1.17327200	4.10417900
H	1.99279000	1.89782400	5.93808000
H	4.03930900	3.21255300	5.43410200
H	4.57529900	3.82011500	3.08610400
H	3.09060700	3.13147900	1.25522800
H	0.85958100	1.46573300	-1.15509500
H	2.08703500	2.52133300	-0.45737100
H	3.23204300	0.52108500	0.57085600
H	3.03902100	0.20133100	-1.15230700
H	2.41280000	-1.37076600	2.96962900
H	4.00001400	-2.92178600	4.06655600
H	5.09092600	-4.72209300	2.74821300
H	4.59726800	-4.96861800	0.33058100
H	3.01024900	-3.42394600	-0.78140300
H	1.46562000	-0.21203000	-2.57372800
H	0.32186700	-1.11626100	-4.54831600
H	-0.99524300	-3.22046100	-4.38239100
H	-1.08151600	-4.45760300	-2.22986000
H	0.05503200	-3.55310500	-0.22875900
C	-2.45423400	0.51650700	-4.15304100
O	-0.96888600	-0.26902000	3.28683000
H	-1.93126600	-0.19669100	3.17145000
H	-1.93477800	-2.30303100	2.11316800

P	1.47559200	-1.09833700	0.27522000
H	-1.79942100	1.38407100	-4.24146100
C	-2.38844500	-0.49295900	-5.21902000
O	-2.99353300	-1.54742100	-5.24249800
O	-1.52905600	-0.09995800	-6.19117100
C	-1.41751100	-1.01084300	-7.28796600
H	-0.78616700	-0.51314400	-8.02411600
H	-0.95709000	-1.95067300	-6.96352900
H	-2.40323800	-1.23204000	-7.70580100

INT8-t

C	-1.93411300	0.18215600	-0.82822100
C	-1.60721700	-0.46334900	0.46666400
C	-3.35578100	0.59617000	-3.35292500
C	-3.46930800	1.58851800	-2.24867200
C	-3.26081100	0.94406300	-0.87367000
C	-2.22147000	-1.48788700	1.08186500
C	-3.43060100	-2.25538700	0.64989800
H	-4.30538500	-2.00266400	1.26311200
H	-4.02955400	-0.26647400	-3.33211700
H	-4.46013600	2.06540800	-2.26815000
H	-2.73048900	2.39111700	-2.39205500
H	-4.09060800	0.25892500	-0.64934100
H	-3.27938700	1.72040600	-0.09698500
H	-1.94315400	-0.60122100	-1.60583300
H	-1.13392200	0.87326000	-1.13766200
H	-3.67339000	-2.04618200	-0.39770400
H	-3.26888900	-3.33414800	0.76070000
Co	-0.29614400	-0.46026900	1.81540100
P	0.42500600	1.58907800	1.40363500
C	-0.80918200	2.85749400	1.03094500

C	-1.83101500	3.05258300	1.97261400
C	-2.82795400	3.99233500	1.74025200
C	-2.82424100	4.73505300	0.55965600
C	-1.81606600	4.54407200	-0.38142700
C	-0.80760200	3.61056800	-0.14864000
C	1.51676900	2.27912600	2.68609400
C	2.45562800	1.43315400	3.29907200
C	3.34867300	1.93777700	4.23864500
C	3.30433700	3.28595000	4.58730800
C	2.36736300	4.12794100	3.99404500
C	1.47590300	3.63188300	3.04669100
C	1.50963000	1.42890900	-0.08344600
C	2.39923800	0.18860900	0.02473900
C	2.57970800	-2.56194600	1.00242800
C	2.64357800	-2.81545300	2.37933600
C	3.56851800	-3.73408100	2.86928000
C	4.41937200	-4.40314900	1.99347700
C	4.34879900	-4.16114900	0.62186200
C	3.43069200	-3.24464100	0.12241200
C	0.75249000	-1.90143200	-1.18911800
C	0.88322400	-1.17740000	-2.37953100
C	0.20671700	-1.58367800	-3.52856500
C	-0.60929600	-2.71161600	-3.49909200
C	-0.72871800	-3.45049100	-2.32116600
C	-0.05719700	-3.04798200	-1.17415000
H	-1.84365300	2.46308600	2.89055800
H	-3.61293900	4.14158300	2.47816300
H	-3.60729900	5.46736400	0.37574300
H	-1.80802000	5.12775400	-1.29972600
H	-0.01897800	3.47795600	-0.88853600

H	2.48412500	0.37198700	3.04468500
H	4.07457500	1.27450900	4.70379300
H	3.99830100	3.67956100	5.32656500
H	2.32853100	5.17985800	4.26816200
H	0.74890900	4.29864900	2.58545400
H	0.85275300	1.34954900	-0.96096700
H	2.11581400	2.33598200	-0.20638800
H	3.11822000	0.30361600	0.84625400
H	2.99775400	0.04973600	-0.88527800
H	1.95664000	-2.30329500	3.05615100
H	3.61476700	-3.93613300	3.93734200
H	5.13605300	-5.12505000	2.37928500
H	5.00896300	-4.69193900	-0.06081800
H	3.36764000	-3.06362300	-0.95082700
H	1.50802200	-0.28581700	-2.42197900
H	0.30964500	-1.00597100	-4.44573600
H	-1.16589400	-3.00659400	-4.38597200
H	-1.35497500	-4.33998000	-2.29771600
H	-0.16648300	-3.62120400	-0.25186600
C	-2.45658000	0.64574500	-4.34334600
O	0.09225400	-0.73141400	3.55534900
H	0.54374800	-0.02384200	4.04107800
H	-1.77938900	-1.86214000	2.03455800
P	1.40563400	-1.32383900	0.40090500
H	-1.75241900	1.47306000	-4.43641900
C	-2.38017600	-0.42474800	-5.34837600
O	-3.04793900	-1.44103700	-5.36007200
O	-1.42573000	-0.14418900	-6.26836000
C	-1.28286100	-1.13195200	-7.29302000
H	-2.24395300	-1.32609300	-7.77654200

H	-0.56581300	-0.72099600	-8.00357900
H	-0.90728800	-2.07133300	-6.87175200

INT8'-s

C	1.37816800	-0.61226400	2.95979900
C	0.22325900	0.22616300	2.49639800
C	2.05117800	1.97887600	1.43758100
C	3.06412100	1.04636000	2.00116800
C	2.61917900	0.24120200	3.21801900
C	-0.62510400	0.83653800	3.34034100
C	-0.62130600	0.74121900	4.83390800
H	-0.38720800	1.71542400	5.28414500
H	1.53900200	2.65034400	2.13172800
H	3.90986700	1.68452400	2.30597900
H	3.46085200	0.39148900	1.21619700
H	2.39743000	0.92779000	4.04775000
H	3.45260400	-0.39285300	3.54762700
H	1.61688000	-1.40549900	2.23833100
H	1.10815800	-1.13986500	3.88851100
H	-1.61747000	0.46447600	5.20694300
H	0.10056800	0.01786900	5.22645500
Co	0.09039200	0.66581000	0.60130500
P	-1.61760100	-0.69492700	0.51625700
C	-3.03641900	-0.38551800	1.60000800
C	-4.03618000	0.50261000	1.18031700
C	-5.09878700	0.81185500	2.02361100
C	-5.17850800	0.23073100	3.28609800
C	-4.19279000	-0.66025700	3.70678700
C	-3.11966100	-0.96107900	2.87548500
C	-2.31364200	-0.76742000	-1.16189300
C	-2.48114400	0.42379600	-1.88288400

C	-3.06809400	0.39766800	-3.14316000
C	-3.47369000	-0.81226000	-3.70143900
C	-3.30302600	-1.99996300	-2.99373600
C	-2.72734500	-1.98008700	-1.72720100
C	-0.97203500	-2.36500500	0.90462700
C	0.23216400	-2.66799900	0.01320900
C	1.01815200	-0.88528600	-2.15294800
C	0.61460900	0.35549000	-2.64939200
C	0.48767500	0.55583900	-4.02198400
C	0.75768100	-0.48556500	-4.90310600
C	1.16773600	-1.72746100	-4.41515200
C	1.30474800	-1.92691500	-3.04707100
C	2.95989200	-1.59648000	-0.21535800
C	3.42728000	-2.49187200	0.75544700
C	4.79032800	-2.73600000	0.89374600
C	5.70549900	-2.08497500	0.07068300
C	5.25214900	-1.19757200	-0.90283200
C	3.89096700	-0.95447300	-1.04735500
H	-3.99978900	0.93457500	0.18023500
H	-5.87048600	1.50023800	1.68686900
H	-6.01276300	0.46787100	3.94246700
H	-4.25709000	-1.12189600	4.68999300
H	-2.34280900	-1.63760200	3.22911900
H	-2.12409200	1.36433700	-1.45670800
H	-3.19681700	1.32575500	-3.69699800
H	-3.92551500	-0.83093800	-4.69101500
H	-3.62245600	-2.94568200	-3.42598400
H	-2.61684100	-2.91492600	-1.17848500
H	-0.66621600	-2.32666000	1.95804100
H	-1.75045300	-3.13408600	0.82708300

H	-0.09266400	-3.08097500	-0.95022100
H	0.88012800	-3.42014100	0.47622500
H	0.39102400	1.17867000	-1.96778700
H	0.17179400	1.52767000	-4.39754000
H	0.65737600	-0.33166400	-5.97556800
H	1.38862900	-2.53889500	-5.10537700
H	1.64913200	-2.89141900	-2.67193900
H	2.73199800	-3.00944600	1.41563100
H	5.13686400	-3.44182900	1.64553300
H	6.77061900	-2.27519400	0.18235100
H	5.96052800	-0.69545600	-1.55826100
H	3.54643500	-0.26948200	-1.82226600
C	1.84475900	2.22166000	0.11374800
O	-0.97060600	2.14121400	0.56364900
H	-1.88082000	2.02463900	0.88370900
H	-1.40100800	1.48727100	2.93879400
P	1.20247500	-1.11803000	-0.35548300
H	2.38470200	1.66346000	-0.65598000
C	1.13443100	3.46135800	-0.29315800
O	0.89324600	4.38832900	0.44425400
O	0.82628700	3.43142900	-1.60948000
C	0.08462000	4.57171800	-2.05630200
H	-0.04040400	4.44239400	-3.13180100
H	-0.88583100	4.60431900	-1.55056100
H	0.62929800	5.49433400	-1.83837300

INT8’-t

C	0.80763700	-0.27462800	3.05946400
C	-0.15877100	0.67557300	2.41192600
C	1.98760500	2.04033300	1.48583200
C	2.80900100	1.02561300	2.19965300

C	2.12229600	0.41546500	3.41899400
C	-0.96086200	1.55080700	3.02424500
C	-1.08727500	1.73252500	4.50396600
H	-0.69319400	2.70753500	4.82198700
H	1.48200000	2.79356400	2.09443600
H	3.72094000	1.54360800	2.53619400
H	3.14821500	0.24401800	1.50739600
H	1.91997400	1.20297500	4.15899700
H	2.80446300	-0.30039700	3.89518900
H	1.03364000	-1.12607800	2.39263700
H	0.35218300	-0.71786300	3.96009900
H	-2.14213500	1.70968100	4.80771800
H	-0.55606000	0.95854200	5.06945200
Co	-0.01945500	0.70526400	0.44940800
P	-1.66877400	-0.83428400	0.56510500
C	-2.77687100	-0.95008200	1.99063300
C	-3.78983000	0.00881200	2.13450400
C	-4.61276000	-0.00660300	3.25392000
C	-4.42851300	-0.97199200	4.24363500
C	-3.42066000	-1.92245500	4.10952700
C	-2.59484600	-1.91231400	2.98822700
C	-2.77320400	-0.85070600	-0.88992200
C	-2.31042900	-0.38378000	-2.12803700
C	-3.10148600	-0.49815200	-3.26695300
C	-4.36642700	-1.07325800	-3.18088400
C	-4.83491700	-1.54115800	-1.95474300
C	-4.04628000	-1.43620200	-0.81351900
C	-0.81546900	-2.46874100	0.43644400
C	0.05333600	-2.49436700	-0.81837200
C	1.32762700	-0.59641500	-2.68237700

C	0.98166000	0.67459500	-3.15598100
C	1.16240200	0.98345900	-4.50244700
C	1.68215100	0.03120000	-5.37440400
C	2.02886800	-1.23610400	-4.90469700
C	1.85791500	-1.55178500	-3.56226300
C	2.75118400	-1.50512200	-0.35603900
C	2.88567200	-2.41898400	0.69840700
C	4.14136000	-2.71410000	1.21955200
C	5.27660800	-2.09813300	0.69651400
C	5.15385300	-1.19520800	-0.35728000
C	3.89994300	-0.89576100	-0.88081400
H	-3.93600900	0.76713600	1.36420200
H	-5.40032000	0.73694200	3.35523900
H	-5.07335500	-0.98273600	5.11950700
H	-3.27882100	-2.68100600	4.87617600
H	-1.81153700	-2.66328600	2.89669200
H	-1.32831600	0.07975800	-2.20197900
H	-2.72449200	-0.13142200	-4.21964500
H	-4.98979500	-1.15607200	-4.06854200
H	-5.82210300	-1.99265000	-1.88328000
H	-4.42200700	-1.81018800	0.13722100
H	-0.20274600	-2.60194100	1.33928100
H	-1.56191500	-3.27436300	0.42389900
H	-0.58078100	-2.50471200	-1.71417700
H	0.67435400	-3.39808100	-0.85263700
H	0.56769100	1.41524600	-2.47009900
H	0.89767500	1.97377700	-4.86835900
H	1.82400500	0.27507100	-6.42540100
H	2.43909100	-1.97763000	-5.58682800
H	2.14937000	-2.53584300	-3.19382100

H	2.00583700	-2.90703700	1.12079800
H	4.23426100	-3.43175100	2.03212700
H	6.25779200	-2.32801400	1.10590000
H	6.03921400	-0.72343300	-0.77832300
H	3.81090200	-0.19547700	-1.71176100
C	1.92157400	2.23686100	0.14350800
O	-0.86158600	1.94609700	-0.62626200
H	-1.77947800	1.74224100	-0.86386300
H	-1.58397200	2.20905200	2.41211900
P	1.10877300	-0.96877700	-0.92082200
H	2.48295100	1.61984600	-0.56134500
C	1.27944400	3.47690100	-0.35795200
O	0.73234400	4.29690000	0.34671900
O	1.41973300	3.57974900	-1.69259100
C	0.74027000	4.70281000	-2.26403100
H	1.01780100	4.71252900	-3.31836000
H	-0.33993400	4.57329300	-2.14376600
H	1.04742000	5.63002100	-1.77329700

INT9-s

C	0.66005700	-1.72824000	3.53726300
C	0.47208600	-1.85726200	2.10082500
C	-0.87115500	0.25346000	3.74443900
C	-0.53583400	-1.12862400	4.26558100
C	0.46921500	-2.24738500	0.92491700
C	0.57462200	-3.23881500	-0.14867500
H	1.07242100	-4.12942500	0.25575400
H	-1.67382000	0.67401100	4.37634400
H	0.00125300	0.91247500	3.85990000
H	-1.40804100	-1.79197400	4.15566300
H	-0.30643000	-1.08899500	5.33699700

H	1.54690600	-1.10276300	3.70106600
H	0.88532400	-2.73358300	3.92206900
H	-0.40436400	-3.55057500	-0.52981600
H	1.18294000	-2.89543800	-0.99086000
Co	0.14336200	-0.06802000	0.89017300
P	-1.36311300	-0.53233300	-0.80570600
P	1.78938800	0.06063000	-0.80099400
C	-2.45454200	-1.99171000	-0.77183400
C	-3.06414400	-2.44515600	-1.95049800
C	-3.91229000	-3.54685300	-1.92309100
C	-4.16984000	-4.20078600	-0.71945500
C	-3.57680800	-3.75126900	0.45710100
C	-2.72177600	-2.65251500	0.43249300
C	-2.54248900	0.80959300	-1.20914000
C	-3.89669800	0.69688000	-0.85810900
C	-4.78074900	1.74292700	-1.10551400
C	-4.33102000	2.91495300	-1.71097200
C	-2.98984000	3.03765200	-2.06597200
C	-2.10207600	1.99642200	-1.81143300
C	-0.38283700	-0.75594700	-2.37619300
C	0.90034400	0.07314400	-2.42217800
C	2.76105400	1.60525600	-0.77643800
C	2.22128300	2.81790300	-1.22980900
C	2.96060900	3.99282800	-1.15389200
C	4.24114100	3.98185200	-0.60607600
C	4.77883500	2.78818100	-0.13456800
C	4.04704700	1.60783400	-0.21869800
C	3.03882700	-1.25425300	-0.83768700
C	3.49593800	-1.85439400	-2.01614200
C	4.42877600	-2.88614400	-1.95852100

C	4.91526500	-3.32043300	-0.72776200
C	4.47419300	-2.71737400	0.44885700
C	3.53490000	-1.69225700	0.40055700
H	-2.88410700	-1.93077900	-2.89439500
H	-4.37490500	-3.89468300	-2.84431900
H	-4.83286900	-5.06306300	-0.70082200
H	-3.77375500	-4.26057200	1.39816400
H	-2.22844100	-2.32448500	1.34594300
H	-4.26416500	-0.21741800	-0.39341300
H	-5.82898200	1.63567100	-0.83371200
H	-5.02623300	3.72762300	-1.91134800
H	-2.62899500	3.94781800	-2.54133300
H	-1.05180300	2.12133300	-2.07196900
H	-0.14874500	-1.82729600	-2.42988600
H	-1.01635600	-0.53106600	-3.24370100
H	0.66850800	1.12420200	-2.63122600
H	1.53938000	-0.25206200	-3.25236300
H	1.20938600	2.85732300	-1.63024900
H	2.53121900	4.92250300	-1.52163800
H	4.81746000	4.90273500	-0.54759600
H	5.77810900	2.76927200	0.29527800
H	4.48640800	0.67959300	0.14117700
H	3.13228300	-1.52169100	-2.98761300
H	4.78005200	-3.34758200	-2.87916100
H	5.64391700	-4.12745400	-0.68612400
H	4.86102500	-3.05024500	1.41003900
H	3.16841300	-1.21482000	1.31141600
C	-1.33713000	0.26982600	2.30102800
H	-2.12801900	-0.48412500	2.17118500
C	-1.91963500	1.64951100	1.96224000

H	-2.87669500	1.57066300	1.42223000
H	-2.15693000	2.23480800	2.86551300
C	-1.01277300	2.42587400	1.08387100
O	-0.07317000	1.86088700	0.49640800
O	-1.15834900	3.71273500	0.84014700
C	-2.27773300	4.40775200	1.41315500
H	-2.17146800	4.47854200	2.49966600
H	-2.25029800	5.40442300	0.97396400
H	-3.21606100	3.90852700	1.14977300
O	1.56550700	0.45685400	2.06787800
H	1.84075800	1.32226500	1.72177400

INT9-t

C	0.24429800	-1.66295100	3.68015000
C	0.42771400	-2.18898900	2.33424000
C	-1.38295500	0.33996100	3.43810200
C	-1.13717800	-1.06362900	3.97027100
C	0.60694900	-2.76725300	1.27748300
C	0.82027600	-3.58215000	0.09537800
H	1.84217600	-3.98366200	0.07588000
H	-2.27781900	0.72704100	3.96009300
H	-0.55156500	0.99378700	3.73804800
H	-1.92306100	-1.74662000	3.61004100
H	-1.25156300	-1.03065700	5.06118900
H	1.01605100	-0.90918900	3.88414600
H	0.42083500	-2.49322900	4.37888800
H	0.11861400	-4.42597700	0.06566400
H	0.68474700	-3.02049100	-0.83803600
Co	0.10248100	0.19969500	0.89125200
P	-1.20188700	-0.55974100	-0.87887800
P	1.81705100	0.26576500	-0.73672700

C	-2.03613100	-2.17771800	-0.80902200
C	-2.34733000	-2.85065300	-1.99925900
C	-2.99989300	-4.07812800	-1.96259700
C	-3.36046400	-4.64100800	-0.74005400
C	-3.07012000	-3.97172600	0.44571700
C	-2.40913500	-2.74696500	0.41400600
C	-2.55074100	0.59260500	-1.31001800
C	-3.88128300	0.28345100	-0.99619800
C	-4.89008100	1.21869900	-1.21344800
C	-4.58522100	2.46911800	-1.74704400
C	-3.26608000	2.78082200	-2.07283300
C	-2.25474500	1.85084600	-1.85240800
C	-0.16266900	-0.66744100	-2.42054100
C	1.01440000	0.29905900	-2.39111700
C	2.88433500	1.73891500	-0.67658600
C	2.41622700	2.97504000	-1.14584200
C	3.22345000	4.10415500	-1.07531900
C	4.49818900	4.02178400	-0.51992200
C	4.96466700	2.80248000	-0.03727200
C	4.16560400	1.66611100	-0.11396200
C	2.93507300	-1.15857800	-0.68792900
C	3.31863200	-1.87320000	-1.82883800
C	4.17937200	-2.96116800	-1.71434700
C	4.66767500	-3.33740300	-0.46485100
C	4.28997000	-2.62907200	0.67516800
C	3.41774400	-1.55095600	0.57050800
H	-2.08732200	-2.41610700	-2.96371700
H	-3.23067400	-4.59391100	-2.89227200
H	-3.87088700	-5.60150300	-0.71294900
H	-3.34953100	-4.40715500	1.40292200

H	-2.14615000	-2.24979100	1.34483900
H	-4.13026600	-0.69225300	-0.58084900
H	-5.91947500	0.96483300	-0.96961800
H	-5.37550900	3.19732400	-1.91801000
H	-3.02056100	3.75319700	-2.49612700
H	-1.22625500	2.12187500	-2.08762400
H	0.20343400	-1.70293400	-2.47026300
H	-0.78566400	-0.50450400	-3.30873400
H	0.67519900	1.33048200	-2.54648200
H	1.72421400	0.09378300	-3.20215200
H	1.41166900	3.06993700	-1.55217000
H	2.85073700	5.05417900	-1.45203600
H	5.12688800	4.90782400	-0.46487900
H	5.96000600	2.72885700	0.39551700
H	4.54918700	0.71573900	0.25161600
H	2.95585600	-1.58443900	-2.81483200
H	4.47347600	-3.51233900	-2.60501100
H	5.34423600	-4.18533600	-0.38008700
H	4.66619500	-2.92525200	1.65223200
H	3.08991600	-1.01106600	1.45974300
C	-1.65175100	0.49147900	1.94508000
H	-2.42773000	-0.23048800	1.65285000
C	-2.16432800	1.90104100	1.62609800
H	-3.08248700	1.86812400	1.01901300
H	-2.46705300	2.42118300	2.55023200
C	-1.20087500	2.77764600	0.88819000
O	-0.12951700	2.36244700	0.44847800
O	-1.49699000	4.05362600	0.65472000
C	-2.74281800	4.59448300	1.10995700
H	-2.79605000	4.59367100	2.20336100

H	-2.76070800	5.62158100	0.74579700
H	-3.58716100	4.03681100	0.68976200
O	1.34050900	0.64288000	2.16124400
H	1.56294100	1.58067800	2.02312000

INT10-s

C	-1.98089800	-3.76137100	0.55425600
C	-1.24751200	-2.44907900	0.73820900
C	-2.26253000	-1.42447200	1.22609700
C	-3.64258700	-2.05087200	0.89949300
C	-3.35094400	-3.29495100	0.06415000
C	0.01793000	-2.20093700	0.38106400
C	1.05021000	-3.17310000	-0.06629000
H	1.89355200	-3.23463500	0.63694300
H	-2.13043700	-0.47446400	0.67557500
H	-4.31069100	-1.34403600	0.39241700
H	-4.14073800	-2.33184800	1.83846300
H	-3.28328900	-3.03083700	-0.99901800
H	-4.12418000	-4.06506500	0.16287600
H	-2.07918700	-4.28288900	1.52047100
H	-1.48123800	-4.44879700	-0.13714700
H	0.63463000	-4.18594400	-0.15964800
H	1.48641800	-2.91228600	-1.04300500
Co	0.51971600	-0.37948300	0.62386700
P	-0.42104100	0.34722900	-1.28074200
P	2.37582500	-0.11524300	-0.39569600
C	-1.76733800	-0.55192700	-2.10526600
C	-1.46739300	-1.68040900	-2.88066100
C	-2.47850100	-2.35982200	-3.55020300
C	-3.79968100	-1.92823800	-3.43729600
C	-4.10817200	-0.82620500	-2.64315300

C	-3.09766100	-0.13890100	-1.97525600
C	-1.00547900	2.04599500	-1.01966800
C	-1.30809800	2.89289800	-2.09521200
C	-1.69101700	4.20754900	-1.85939000
C	-1.77379400	4.68698000	-0.55135000
C	-1.48408300	3.84978700	0.52195100
C	-1.10034800	2.53120500	0.28954300
C	0.88832600	0.50384000	-2.60373500
C	2.14874400	-0.26158900	-2.20861200
C	2.64255000	1.64906800	-0.05184900
C	3.01705600	2.54850500	-1.06031900
C	3.09249000	3.90933900	-0.78666300
C	2.79714700	4.38399400	0.49033400
C	2.44036200	3.49520800	1.50033700
C	2.35336100	2.13175500	1.23560000
C	3.80831300	-1.09460700	0.09097300
C	4.50826900	-1.87754000	-0.83560400
C	5.59365600	-2.64040700	-0.41614500
C	5.98063200	-2.62849700	0.92139500
C	5.28271100	-1.85345600	1.84737300
C	4.19520000	-1.09187000	1.43918100
H	-0.43559600	-2.02498900	-2.97106300
H	-2.23586600	-3.22724500	-4.16010900
H	-4.59076600	-2.45684700	-3.96436700
H	-5.13959600	-0.49313200	-2.54765700
H	-3.34179700	0.72708600	-1.35847800
H	-1.25006600	2.51990800	-3.11847800
H	-1.92738600	4.86102600	-2.69638500
H	-2.07061000	5.71808400	-0.37079700
H	-1.55333300	4.22031300	1.54315800

H	-0.88376000	1.87432400	1.13582100
H	0.49863800	0.15777100	-3.56752900
H	1.10725800	1.57317500	-2.70519000
H	3.03394900	0.08250800	-2.75980600
H	2.03906800	-1.33227000	-2.42672100
H	3.26291000	2.19085800	-2.05963500
H	3.38752300	4.60095500	-1.57284300
H	2.85124000	5.45063300	0.69738300
H	2.21664600	3.86303200	2.49954400
H	2.05367700	1.43964900	2.02361900
H	4.21777000	-1.89362700	-1.88482200
H	6.13863300	-3.24428000	-1.13818100
H	6.82888600	-3.22771400	1.24464600
H	5.58436900	-1.84694200	2.89214500
H	3.63070700	-0.50333800	2.16230000
C	-3.22058800	-0.11370200	3.14623500
O	-4.01487200	-0.26530500	4.04403600
O	-3.19215000	0.99345000	2.35560400
C	-4.20179500	1.96060700	2.66160800
H	-4.10752600	2.73757700	1.90094200
H	-5.19425000	1.50216600	2.62537100
H	-4.04648600	2.37664900	3.66171000
O	0.99834200	-0.51869700	2.33878000
H	1.18022400	-1.42278600	2.64466100
C	-2.15530100	-1.08879700	2.72220700
H	-1.16804700	-0.66354100	2.94836200
H	-2.27536400	-1.99341500	3.32926000

INT10-t

C	-2.55946200	-3.61151600	0.48265300
C	-1.78913800	-2.34601100	0.78841400

C	-2.78383400	-1.23907200	1.08425900
C	-4.16281900	-1.85241300	0.76429200
C	-3.87163100	-3.07915000	-0.09167200
C	-0.45595500	-2.21792000	0.69252000
C	0.57086900	-3.28142400	0.50086000
H	1.34908500	-3.23905400	1.27442000
H	-2.58216700	-0.39377500	0.40182400
H	-4.83320500	-1.13216100	0.28108100
H	-4.64923700	-2.15693500	1.70292200
H	-3.71883000	-2.78580700	-1.13894000
H	-4.67611200	-3.82208000	-0.07226700
H	-2.74855700	-4.15750300	1.42222400
H	-2.02323300	-4.29478600	-0.18573100
H	0.11890000	-4.28186200	0.55751900
H	1.08300200	-3.20661000	-0.47044100
Co	0.34273000	-0.49779600	0.84229200
P	-0.33967700	0.28127900	-1.22296700
P	2.56176400	-0.29517300	-0.07714100
C	-1.64983700	-0.53482000	-2.17578000
C	-1.37059300	-1.74395000	-2.82755800
C	-2.35256800	-2.37333300	-3.58384100
C	-3.62339200	-1.80782000	-3.68540300
C	-3.91352700	-0.61970900	-3.01898600
C	-2.93297200	0.01746900	-2.26320500
C	-0.83936200	2.00552200	-0.96697200
C	-0.71809200	2.97144900	-1.97541500
C	-1.06347500	4.29270800	-1.71543700
C	-1.53655800	4.65872900	-0.45520000
C	-1.67241000	3.70155900	0.54669100
C	-1.31923100	2.37902700	0.29501000

C	1.13099700	0.32707900	-2.36231200
C	2.26517600	-0.57617200	-1.88417700
C	3.04474900	1.45679600	-0.02105100
C	4.14430400	1.94391800	-0.74349300
C	4.42800300	3.30422700	-0.74695600
C	3.61781700	4.19073800	-0.03556600
C	2.53007800	3.71619400	0.69048500
C	2.24697200	2.35202400	0.70242400
C	4.05363100	-1.24758100	0.31602900
C	4.27240300	-2.50940000	-0.25405200
C	5.38557400	-3.25951700	0.10993800
C	6.28397100	-2.76575700	1.05317100
C	6.06849600	-1.51690900	1.63155600
C	4.95929400	-0.76007700	1.26894800
H	-0.37898300	-2.19345800	-2.75031300
H	-2.12583700	-3.30557200	-4.09694700
H	-4.39023400	-2.29926500	-4.28012200
H	-4.90625600	-0.18068900	-3.09198400
H	-3.16270500	0.94830500	-1.74302000
H	-0.35963000	2.69298500	-2.96634100
H	-0.96587400	5.03980200	-2.49999900
H	-1.80196900	5.69503700	-0.25687100
H	-2.04551400	3.98053700	1.53024400
H	-1.41967400	1.63469800	1.08721000
H	0.81507400	0.06369600	-3.37879600
H	1.47596200	1.36909500	-2.38493300
H	3.17808100	-0.39320300	-2.46610600
H	2.00474500	-1.63462300	-2.02588300
H	4.78451200	1.25463100	-1.29507600
H	5.28415900	3.67658500	-1.30559000

H	3.84381000	5.25497000	-0.04364400
H	1.90294400	4.40413300	1.25398100
H	1.40378400	1.97766200	1.28827200
H	3.57820200	-2.91030300	-0.99240000
H	5.55256300	-4.23238400	-0.34734500
H	7.15364100	-3.35434300	1.33615800
H	6.77006600	-1.12579400	2.36510900
H	4.80540900	0.22320900	1.71314400
C	-3.76801400	0.30791300	2.83109900
O	-4.44385400	0.34171400	3.83136900
O	-3.87987300	1.21589100	1.82451800
C	-4.88745700	2.20983700	2.04256000
H	-4.87644000	2.84269800	1.15369900
H	-5.86620400	1.73878500	2.17043100
H	-4.66312800	2.79581700	2.93897000
O	0.50708000	-0.22830000	2.60343700
H	1.41110700	-0.35019400	2.93459800
C	-2.70335200	-0.71265500	2.52200800
H	-1.71694800	-0.27453000	2.73324500
H	-2.82809100	-1.53312800	3.23782700

INT11-s

C	-2.84260400	-2.24295300	-2.14227700
C	-1.91678700	-1.73700200	-1.03154600
C	-2.61897000	-1.98927200	0.28627400
C	-4.10126200	-1.91244200	-0.09706400
C	-4.15323500	-2.63192800	-1.44473300
C	-0.78193400	-1.04996200	-1.27189200
C	-0.25286500	-0.89846400	-2.66185000
H	0.72138400	-1.39149100	-2.78629400
H	-2.33337600	-1.23532300	1.02912600

H	-4.39964900	-0.86009000	-0.21267900
H	-4.75948300	-2.35201900	0.66278500
H	-5.03799500	-2.37069800	-2.03542700
H	-4.19674400	-3.71820600	-1.28889800
H	-2.40730000	-3.07903000	-2.70625300
H	-3.01225700	-1.43864100	-2.87312700
H	-0.92303200	-1.33824200	-3.41219900
H	-0.11845200	0.15440300	-2.94611600
Co	0.05047300	-0.18176000	0.27495800
P	-0.31454000	1.87093100	-0.44818900
P	2.07369100	-0.01692600	-0.58673900
C	-1.54323000	2.02747600	-1.77023200
C	-1.28051600	2.65131800	-2.99397600
C	-2.25523600	2.67939100	-3.98844600
C	-3.49563300	2.08757700	-3.76788100
C	-3.76881800	1.47230600	-2.54666400
C	-2.79636900	1.43651000	-1.55421300
C	-0.79293700	3.07088700	0.83319000
C	-1.87607600	3.94636200	0.69652800
C	-2.15084400	4.87140900	1.70122400
C	-1.35439300	4.92817700	2.84180900
C	-0.27779100	4.05446300	2.98630900
C	-0.00043900	3.12650200	1.99082200
C	1.29963900	2.53405300	-1.06708100
C	2.12767000	1.43437800	-1.72164500
C	3.12121200	0.51173000	0.81412900
C	4.42773500	0.96087500	0.57257500
C	5.22857900	1.38533800	1.62617800
C	4.73759100	1.35812100	2.93126500
C	3.44756000	0.90104600	3.18038000

C	2.63776000	0.47728500	2.12783700
C	3.03126900	-1.35020500	-1.35831300
C	3.32143200	-1.37285200	-2.72849700
C	4.05625100	-2.42289700	-3.27075400
C	4.50056000	-3.45977000	-2.45441000
C	4.21491300	-3.44467600	-1.09029900
C	3.48502700	-2.39684200	-0.54104500
H	-0.31360500	3.11394600	-3.18647400
H	-2.04222100	3.16500300	-4.93823100
H	-4.25336800	2.10777800	-4.54822100
H	-4.73982800	1.01226500	-2.37022600
H	-2.99931100	0.93776700	-0.60519800
H	-2.49798400	3.91393300	-0.19661600
H	-2.99027800	5.55394700	1.58719800
H	-1.57306800	5.65456400	3.62158100
H	0.34390200	4.09238900	3.87808500
H	0.83523700	2.43380900	2.10921700
H	1.15121300	3.40087300	-1.72184100
H	1.81766300	2.90886200	-0.17339800
H	3.16096500	1.76115700	-1.89427000
H	1.70696800	1.14804100	-2.69308000
H	4.82816900	0.96487400	-0.44207700
H	6.24081300	1.73239700	1.42955600
H	5.36762000	1.68719800	3.75501800
H	3.06613400	0.86605600	4.19895500
H	1.62634800	0.12204500	2.33149800
H	2.98264000	-0.56717600	-3.37890100
H	4.28608500	-2.42696300	-4.33395900
H	5.07535000	-4.27888500	-2.88115700
H	4.56877900	-4.24910400	-0.44889800

H	3.27722800	-2.37744200	0.52835900
C	-2.16372900	-3.32653200	2.35681200
O	-1.15090600	-2.94601700	2.93169100
O	-3.28199800	-3.66058900	2.99639700
C	-3.23400600	-3.53594900	4.42899300
H	-4.23010000	-3.80779900	4.77589700
H	-2.48152900	-4.21396300	4.83943400
H	-2.98537300	-2.50941500	4.70948700
C	-2.30269500	-3.40315300	0.86695000
H	-1.36256800	-3.79514600	0.46216200
H	-3.08720800	-4.11803600	0.59616800
O	-0.81589000	0.07518100	1.86069600
H	-1.25100300	0.93210400	1.99699200
O	0.58895200	-1.97192000	1.08570600
H	0.43294600	-2.63855700	0.39584200
H	-0.02598600	-2.21483000	1.83957100

INT11-t

C	-2.79461000	-3.57880000	-0.07648000
C	-1.89875600	-2.41599100	0.28178100
C	-2.71598900	-1.42525400	1.09208000
C	-4.18793200	-1.81951900	0.83840200
C	-4.14924900	-2.88892600	-0.25745500
C	-0.63539500	-2.21322400	-0.10649800
C	0.22656000	-3.14216900	-0.90276100
H	1.28645300	-3.10668700	-0.60131900
H	-2.51335800	-0.40708900	0.71940800
H	-4.80593500	-0.95656700	0.56698100
H	-4.62515700	-2.22953700	1.75937000
H	-4.17890300	-2.41682500	-1.24815500
H	-4.99158700	-3.58769400	-0.20483000

H	-2.84469600	-4.29930200	0.75692300
H	-2.46116500	-4.12866100	-0.96419400
H	-0.09754900	-4.18787600	-0.79967000
H	0.20325800	-2.90903200	-1.97816300
Co	0.29592100	-0.63620300	0.57149700
P	-0.52499100	0.55289900	-1.18448000
P	2.45419000	-0.17091600	-0.33011200
C	-1.89879900	-0.08703300	-2.19582400
C	-1.66270100	-1.05657000	-3.17932300
C	-2.71096400	-1.55180900	-3.94647600
C	-4.00826800	-1.08590300	-3.73786700
C	-4.25228900	-0.12867200	-2.75626700
C	-3.20566200	0.36731800	-1.98441700
C	-1.02032700	2.26077600	-0.79529300
C	-0.71821600	3.31095800	-1.67429700
C	-1.12787400	4.60793300	-1.38619600
C	-1.85336300	4.86929300	-0.22695700
C	-2.17834400	3.82723700	0.63734800
C	-1.76553400	2.52668100	0.36182100
C	0.90125400	0.72616400	-2.37602300
C	2.04264900	-0.25835700	-2.12446000
C	3.10422000	1.50465900	-0.09542200
C	4.38595500	1.85438400	-0.54629100
C	4.82231800	3.16885600	-0.43512500
C	3.98368000	4.14029700	0.11284600
C	2.71314800	3.79577700	0.56464700
C	2.27080800	2.47793100	0.47242900
C	3.80910300	-1.32682700	-0.00989800
C	4.20158000	-2.32006800	-0.91703600
C	5.19134200	-3.23551400	-0.56702100

C	5.79102000	-3.17674400	0.68840000
C	5.40199600	-2.19645000	1.60083900
C	4.41480600	-1.27903400	1.25859200
H	-0.64938400	-1.41717100	-3.36246200
H	-2.51421900	-2.29563600	-4.71563700
H	-4.82742000	-1.46701000	-4.34388700
H	-5.26250700	0.24095800	-2.59163500
H	-3.40459100	1.12484700	-1.22683200
H	-0.17301300	3.12678200	-2.59826800
H	-0.88412000	5.41427900	-2.07472400
H	-2.17059800	5.88510600	-0.00040900
H	-2.75092600	4.03003400	1.53999000
H	-1.99571900	1.72225000	1.05668300
H	0.53154900	0.65586900	-3.40604800
H	1.28697700	1.74423800	-2.24037800
H	2.90826900	-0.01129800	-2.75369200
H	1.74246900	-1.28795000	-2.36384100
H	5.04220800	1.09697900	-0.97634200
H	5.81900900	3.43853300	-0.77822600
H	4.32971400	5.16882800	0.19350100
H	2.06160200	4.54993600	1.00212000
H	1.29051900	2.19435600	0.85989000
H	3.74672300	-2.37439600	-1.90575300
H	5.49961500	-3.99403000	-1.28345000
H	6.56528700	-3.89236000	0.95551200
H	5.87411500	-2.14116000	2.57937900
H	4.13039500	-0.49753900	1.96620200
C	-2.56771600	-0.20917600	3.37538900
O	-1.97690600	0.07407300	4.39358100
O	-3.53798300	0.55987000	2.83285000

C	-3.79718300	1.76383100	3.56443200
H	-4.58939100	2.27724900	3.01812900
H	-4.11718100	1.53115700	4.58363600
H	-2.89087400	2.37670400	3.61406100
C	-2.31554300	-1.45818000	2.57458700
H	-1.24528600	-1.65515000	2.69417300
H	-2.82820900	-2.29005100	3.08204800
O	-0.09343800	0.63830000	1.84464100
H	0.20916400	0.31317000	2.71123600
O	1.09885000	-1.91011200	2.09462500
H	2.03928800	-2.11590900	1.94339200
H	0.62882200	-2.75888300	2.02092800

TS1-s

C	12.37997600	0.76614900	-6.56257000
C	12.37732100	2.23784600	-6.26458300
C	11.36828900	2.25950900	-4.65014500
C	11.70450600	0.84046400	-4.24768000
C	11.61997000	-0.01081000	-5.49751000
C	12.86363400	3.27152200	-6.94090100
C	13.39854500	3.46280800	-8.30266000
H	14.22087500	4.18896300	-8.33237900
H	10.38670700	2.32517500	-5.13322500
H	11.00262000	0.51425700	-3.47046200
H	12.71138700	0.80869500	-3.80608400
H	10.56840100	-0.13085600	-5.78884200
H	12.02649000	-1.01650600	-5.35347200
H	13.43940100	0.46483700	-6.57995300
H	12.00110900	0.58664300	-7.57721000
H	13.76184700	2.51995200	-8.73486900
H	12.61145100	3.84703900	-8.96690700

Co	12.54555900	3.97763800	-5.28389600
P	12.20689800	5.98250600	-5.99046800
P	14.34125200	4.69687100	-4.37546000
C	11.36640100	6.05647800	-7.59553900
C	10.19938400	5.29719600	-7.76297600
C	9.53002100	5.30403400	-8.98116700
C	10.02236400	6.06100600	-10.04403100
C	11.17973500	6.81770200	-9.88379400
C	11.85115600	6.81975500	-8.66260100
C	11.33752200	7.20667100	-4.95651400
C	11.62218200	7.26570100	-3.58737500
C	11.00612500	8.21071700	-2.77519600
C	10.09267100	9.10707100	-3.32472500
C	9.79651700	9.05291800	-4.68571900
C	10.41020500	8.10743400	-5.50136500
C	13.88867100	6.70877100	-6.23128400
C	14.68842300	6.41871100	-4.96797700
C	14.73261600	4.75199800	-2.59733900
C	13.74065900	4.91189800	-1.62076100
C	14.09788500	5.03279600	-0.27997400
C	15.43720600	4.99558900	0.09699000
C	16.42867600	4.83948500	-0.86983300
C	16.08191400	4.71619400	-2.21016300
C	15.62441300	3.62110400	-5.08082200
C	16.35170900	3.97720900	-6.22135800
C	17.23913200	3.07104100	-6.79674300
C	17.39987000	1.80299400	-6.24386200
C	16.67824600	1.44074500	-5.10637900
C	15.79317700	2.34257600	-4.52869000
H	9.82631300	4.69396600	-6.93318900

H	8.62445800	4.71372300	-9.10503300
H	9.50136800	6.06072300	-10.99912900
H	11.56367800	7.41096200	-10.71103300
H	12.75281000	7.42053800	-8.54866900
H	12.31111000	6.54878400	-3.14333300
H	11.22735900	8.23031500	-1.71031600
H	9.60313100	9.84300700	-2.69036000
H	9.07987000	9.74908500	-5.11628200
H	10.16673300	8.07015100	-6.56191600
H	14.33173400	6.21140900	-7.10532100
H	13.84128200	7.78493300	-6.43985600
H	14.37654400	7.08912500	-4.15669100
H	15.76748900	6.56966900	-5.10019000
H	12.68445800	4.93207500	-1.88747700
H	13.31907600	5.14878400	0.47050000
H	15.71087800	5.08394600	1.14631700
H	17.47676800	4.80996700	-0.58013200
H	16.86212100	4.58622600	-2.95955100
H	16.23361200	4.96611200	-6.66494200
H	17.81119500	3.35960800	-7.67600600
H	18.09567400	1.09829100	-6.69373900
H	16.81314400	0.45573100	-4.66440100
H	15.23793600	2.06519700	-3.63128500
C	11.57709600	3.31297900	-3.66006500
C	10.51352200	4.25441200	-3.30595400
O	10.42230000	4.83277000	-2.23689700
O	9.62277000	4.44485900	-4.32362600
C	8.56029700	5.35678300	-4.01988000
H	8.01892000	5.50088700	-4.95683600
H	7.89931600	4.92881400	-3.26013400

H	8.95786400	6.30810900	-3.65275700
H	12.18228600	3.02352100	-2.79969800

TS1-t

C	11.98454200	-0.08931200	-4.59489300
C	11.48296100	1.18200200	-5.22533900
C	11.02936600	2.16062800	-3.74192700
C	12.02314300	1.53495400	-2.78664400
C	11.99337700	0.04409100	-3.07305400
C	11.26031100	1.57304300	-6.46050200
C	11.19269300	1.01546600	-7.82042400
H	11.86953000	1.54333600	-8.50621500
H	10.03729600	1.69114900	-3.69412700
H	11.74259900	1.76591700	-1.75216400
H	13.02349300	1.96389600	-2.95371600
H	11.07630300	-0.38566100	-2.65074400
H	12.83196100	-0.49284500	-2.61931100
H	13.00029600	-0.25618900	-4.98485200
H	11.38798400	-0.94172600	-4.94342000
H	11.45687300	-0.05148100	-7.83753400
H	10.17933000	1.12019800	-8.23235100
Co	11.54009500	3.21895200	-5.59336200
P	10.58601600	4.60131900	-7.03415600
P	13.63903600	3.99257200	-6.36240700
C	9.26034200	3.90421900	-8.06016400
C	8.21864100	3.21589500	-7.42112500
C	7.19643100	2.64944800	-8.17533800
C	7.20710300	2.75416200	-9.56539100
C	8.23827000	3.43788800	-10.20402900
C	9.26098700	4.01566000	-9.45614800
C	9.98092600	6.22301100	-6.46374700

C	8.87777300	6.84730600	-7.06060000
C	8.48095400	8.11292600	-6.63982200
C	9.18102400	8.76697000	-5.62781200
C	10.27702400	8.15135000	-5.02705700
C	10.67005700	6.88205800	-5.43813300
C	11.92390600	5.05344200	-8.23700100
C	13.21857700	5.38075400	-7.49831300
C	14.95909300	4.59243900	-5.27197800
C	14.59952700	5.33493000	-4.13795700
C	15.57855700	5.82908000	-3.28356700
C	16.92307200	5.57323500	-3.54673500
C	17.28692400	4.82817800	-4.66628300
C	16.31189400	4.33737600	-5.52880600
C	14.37818300	2.69870100	-7.39944300
C	14.56128700	2.81963400	-8.78148600
C	15.04712500	1.74345200	-9.52223000
C	15.35792200	0.54219700	-8.89222900
C	15.19048700	0.41604100	-7.51291600
C	14.69589900	1.48316800	-6.77451300
H	8.21593100	3.12657500	-6.33412000
H	6.38945700	2.11971700	-7.67358500
H	6.40957600	2.30272300	-10.15188800
H	8.24734300	3.52631700	-11.28838200
H	10.05402000	4.55539300	-9.97100800
H	8.32717000	6.34087200	-7.85229000
H	7.62089500	8.58994200	-7.10526300
H	8.86636800	9.75588600	-5.30056800
H	10.81566800	8.65262300	-4.22542900
H	11.49743000	6.37678700	-4.93504800
H	12.06682900	4.18368900	-8.89530900

H	11.60587800	5.89593400	-8.86453400
H	13.09145300	6.27488700	-6.87162700
H	14.04106400	5.60598600	-8.18956900
H	13.54523100	5.52521100	-3.92614800
H	15.29291800	6.40738400	-2.40767100
H	17.68900000	5.95333500	-2.87426000
H	18.33655400	4.62885800	-4.87155500
H	16.59997700	3.75423800	-6.40248300
H	14.33157500	3.75352100	-9.29307800
H	15.18675100	1.84915400	-10.59591100
H	15.73502800	-0.29592100	-9.47411800
H	15.44399600	-0.51759800	-7.01401300
H	14.56150500	1.38487400	-5.69562900
C	10.87634400	3.62034800	-3.74598200
C	9.51261600	4.15357100	-3.73529900
O	8.53854500	3.58343700	-4.20772000
O	9.45496100	5.36653700	-3.15153100
C	8.17094900	5.99899300	-3.21085600
H	7.41138800	5.36941800	-2.73978700
H	8.28290000	6.94112800	-2.67335400
H	7.88960400	6.18503600	-4.25348600
H	11.62831000	4.19605000	-3.19995600

TS2-s

C	-2.58774500	-0.96277700	-3.77229600
C	-1.87819000	-0.86893200	-2.45279700
C	-2.60588900	0.10436400	-1.59298500
C	-4.06607700	-0.10155600	-2.02478500
C	-3.91907900	-0.21477000	-3.54856000
C	-0.83617000	-1.58158400	-1.95063300
C	-0.00451700	-2.50668600	-2.79282800

H	0.53167500	-3.24658200	-2.18452800
C	-2.19170800	0.02533300	-0.13630200
H	-2.32742100	1.10567900	-1.96250500
H	-4.72589600	0.71313500	-1.71125100
H	-4.45686800	-1.03762500	-1.59788300
H	-3.85935200	0.79294600	-3.98100700
H	-4.76890100	-0.71394200	-4.02435500
H	-2.73866200	-2.01903200	-4.03835100
H	-1.99069100	-0.53950500	-4.59367400
H	-0.62092200	-3.06698200	-3.51199000
H	0.74998300	-1.96228700	-3.37727100
Co	-0.38149200	-0.84424000	-0.05116400
P	0.35715300	-0.31891500	1.99812700
P	0.86966900	0.84002900	-0.75304900
C	-0.44103500	-0.70461800	3.57509800
C	-0.90704500	0.30315500	4.42706700
C	-1.53261000	-0.03356900	5.62253100
C	-1.71228700	-1.37051600	5.96898000
C	-1.26036300	-2.37736500	5.11905200
C	-0.62535000	-2.04933400	3.92655900
C	1.88178900	-1.33152400	1.92731700
C	3.13991700	-0.82325300	2.28254100
C	4.27988500	-1.60923900	2.15771200
C	4.18375800	-2.91355800	1.67415300
C	2.94135100	-3.43259000	1.32337500
C	1.79452700	-2.64966600	1.44380600
C	0.96264900	1.41462200	2.02688100
C	1.65234400	1.71973300	0.69841000
C	2.31445700	0.29796100	-1.71738100
C	2.74500600	-1.02835400	-1.61531900

C	3.90060100	-1.44683900	-2.26746800
C	4.63215100	-0.54104200	-3.03090000
C	4.21069300	0.78369200	-3.13745300
C	3.05945500	1.20653000	-2.48215200
C	0.08660600	2.11892700	-1.77046800
C	-0.44806900	3.27929000	-1.20247100
C	-1.13170300	4.19786100	-1.99491700
C	-1.29317300	3.96047500	-3.35669200
C	-0.76328800	2.80471500	-3.93123600
C	-0.07793900	1.88720300	-3.14494900
H	-0.79385800	1.34980400	4.15440100
H	-1.88380200	0.75431100	6.28550000
H	-2.20494400	-1.62834300	6.90418300
H	-1.39586700	-3.42290200	5.38774300
H	-0.25647900	-2.84065900	3.27428000
H	3.23532900	0.18671500	2.67914900
H	5.24729000	-1.20462600	2.44804700
H	5.07865500	-3.52525700	1.58027000
H	2.85831600	-4.45332200	0.95477400
H	0.82448500	-3.06224000	1.16543400
H	0.07885200	2.04357400	2.16681700
H	1.63204400	1.59932300	2.87657200
H	2.68969000	1.36470100	0.71524400
H	1.69711300	2.79795700	0.50090900
H	2.16777400	-1.74199700	-1.02628100
H	4.22414900	-2.48251700	-2.18218800
H	5.53307000	-0.86637300	-3.54683600
H	4.78237800	1.49173700	-3.73367800
H	2.73488700	2.24331100	-2.56977000
H	-0.33535400	3.47008700	-0.13405300

H	-1.53555400	5.10392700	-1.54696800
H	-1.82677600	4.67959500	-3.97443800
H	-0.88099100	2.62141500	-4.99764300
H	0.33437500	0.98279000	-3.59459900
C	-2.40793700	1.26842000	0.66026500
O	-2.01693900	1.45975300	1.80009500
O	-3.15242500	2.17622600	-0.00330500
C	-3.47487100	3.35739800	0.73815100
H	-4.07187300	3.97192100	0.06420100
H	-4.04338800	3.10074900	1.63624400
H	-2.56409400	3.88642700	1.03733400
H	-2.73454700	-0.76925400	0.39795800
O	-1.15258400	-2.62533300	0.36255000
H	-2.00039000	-2.56970900	0.83274400
H	-1.26590700	-2.23241000	-0.88406600

TS2-t

C	-2.84332000	-0.78584600	-3.79818500
C	-2.12692900	-0.73205100	-2.48054400
C	-2.85520000	0.19620400	-1.57450700
C	-4.30811100	0.09023400	-2.05982500
C	-4.12093400	0.04813200	-3.58100700
C	-1.03075500	-1.41528500	-2.04961100
C	-0.11930200	-2.18286800	-2.96411200
H	0.43314500	-2.96456400	-2.42885800
C	-2.45432300	0.03739700	-0.12220300
H	-2.50995000	1.20217900	-1.88743400
H	-4.93316600	0.92170300	-1.72052500
H	-4.76047200	-0.84326500	-1.69366300
H	-3.96214700	1.06949700	-3.95367300
H	-4.98627200	-0.35644400	-4.11518400

H	-3.07353300	-1.83435900	-4.03988200
H	-2.21792300	-0.42917900	-4.62952000
H	-0.67646600	-2.67428300	-3.77539000
H	0.62870100	-1.53057500	-3.43879500
Co	-0.53125500	-0.78588900	-0.13330600
P	0.33955300	-0.35515400	1.94448100
P	1.07095200	0.70872400	-0.82490100
C	-0.43488100	-0.71644600	3.54086200
C	-0.92685200	0.29055600	4.37839400
C	-1.51327100	-0.04516600	5.59353300
C	-1.62952300	-1.38015200	5.97367500
C	-1.15106900	-2.38709000	5.13856800
C	-0.55204700	-2.05914900	3.92788800
C	1.91225300	-1.29332700	1.97805900
C	3.09028500	-0.74789000	2.50733500
C	4.27328500	-1.47997700	2.49645800
C	4.29350900	-2.77209900	1.97483000
C	3.12442600	-3.33272000	1.46958500
C	1.94081700	-2.59818900	1.46198100
C	0.87110400	1.40452600	1.90453700
C	1.71957100	1.63461500	0.65263100
C	2.52706200	0.18303300	-1.76157600
C	2.98191800	-1.13341200	-1.62269100
C	4.14705200	-1.53901400	-2.26488800
C	4.85625900	-0.63664600	-3.05391500
C	4.40411000	0.67408900	-3.20025000
C	3.24434500	1.08905400	-2.55545700
C	0.18669000	1.92024700	-1.84130300
C	-0.43933700	3.02803300	-1.25882600
C	-1.20820600	3.88940300	-2.03759900

C	-1.36671900	3.64593600	-3.39831400
C	-0.75111600	2.53947800	-3.98526900
C	0.01789700	1.67763300	-3.21337100
H	-0.87123800	1.33333000	4.07663900
H	-1.88587500	0.74210100	6.24542300
H	-2.09314900	-1.63623100	6.92406100
H	-1.23459300	-3.43069900	5.43416600
H	-0.15282800	-2.84920000	3.29060300
H	3.09161900	0.25219800	2.94065000
H	5.18130300	-1.04230300	2.90616200
H	5.21978400	-3.34290000	1.97262900
H	3.13032200	-4.34626500	1.07263700
H	1.03425500	-3.03233100	1.04012600
H	-0.05166600	1.99778100	1.88681100
H	1.42083400	1.69128600	2.81042900
H	2.73832500	1.25998800	0.80806700
H	1.80780700	2.70106400	0.40905600
H	2.41990900	-1.83924800	-1.01036300
H	4.49733900	-2.56305700	-2.15172600
H	5.76475300	-0.95502800	-3.56077500
H	4.95885900	1.37716100	-3.81780600
H	2.89277600	2.11417000	-2.67222300
H	-0.33548600	3.22124100	-0.19031700
H	-1.68415100	4.75288100	-1.57749600
H	-1.96709900	4.31965600	-4.00569000
H	-0.86731600	2.35156500	-5.05104800
H	0.49714900	0.81390600	-3.67620800
C	-2.63847600	1.20346400	0.75689800
O	-2.30278000	1.27435100	1.93185100
O	-3.22953000	2.24953900	0.12412700

C	-3.42531100	3.40693800	0.93775400
H	-3.95399100	4.12431900	0.30895900
H	-4.01711500	3.16103100	1.82364400
H	-2.46372800	3.81952800	1.26572600
H	-2.89576100	-0.84365000	0.36855300
O	-0.92640200	-2.74448200	0.10794200
H	-1.67177200	-2.81999500	0.72694400
H	-1.30556700	-2.21777600	-1.04340600

TS2'-s

C	3.25862400	-0.09535000	3.45227400
C	2.20781400	-0.20255100	2.37000500
C	2.26521300	-1.57422900	1.76458800
C	2.79706900	-2.42683500	2.92282800
C	3.86140100	-1.51230200	3.53302200
C	1.24828500	0.65727300	2.00305800
C	0.99763900	2.01906000	2.55933400
H	-0.05220300	2.33416900	2.43648500
C	0.92399200	-1.97582700	1.14336000
H	3.03036900	-1.59819800	0.97377600
H	3.18587800	-3.40084200	2.60335400
H	1.99052600	-2.61170100	3.65078400
H	4.77375800	-1.56423000	2.92332600
H	4.14498700	-1.80007900	4.55036800
H	2.79897600	0.20342900	4.40634900
H	4.01910600	0.66846700	3.23871300
H	1.22332900	2.07015200	3.63582100
H	1.60045300	2.79015300	2.05847200
Co	-0.14592200	-0.02776700	0.86760600
P	-2.04258200	-0.39192900	-0.45467500
P	0.74706400	0.82842900	-0.99936100

C	-3.27031300	-1.70855300	-0.27317000
C	-3.26760300	-2.83683600	-1.10276600
C	-4.21688800	-3.83677800	-0.91554500
C	-5.16032700	-3.72829800	0.10345800
C	-5.15358200	-2.61678900	0.94303000
C	-4.21310700	-1.61062800	0.75891900
C	-2.93344300	1.18056200	-0.16610100
C	-3.55702500	1.90302700	-1.19193800
C	-4.11427600	3.15216500	-0.93671100
C	-4.06288600	3.69461800	0.34732200
C	-3.46067500	2.98036700	1.37955800
C	-2.89498200	1.73234800	1.12598000
C	-1.53836600	-0.29921500	-2.22047000
C	-0.52024900	0.82739800	-2.35811900
C	1.08706100	2.60913400	-0.79209900
C	-0.00261000	3.41202000	-0.42032100
C	0.16647900	4.77160600	-0.19203700
C	1.42772800	5.34830500	-0.33035400
C	2.51249400	4.56002400	-0.70558700
C	2.34862900	3.19566000	-0.93486300
C	2.25568000	0.06010900	-1.64962000
C	2.28544000	-0.61056900	-2.87786400
C	3.46971800	-1.18243300	-3.33627100
C	4.62908700	-1.10481900	-2.56956300
C	4.60451700	-0.45357000	-1.33763300
C	3.42676300	0.12366200	-0.87770100
H	-2.52487800	-2.93879000	-1.89121000
H	-4.21917200	-4.70486200	-1.57143200
H	-5.90183000	-4.51188200	0.24372900
H	-5.88692900	-2.52882800	1.74172300

H	-4.21444800	-0.74087900	1.41463400
H	-3.60934300	1.49281600	-2.20000500
H	-4.59486200	3.70321700	-1.74236700
H	-4.49876100	4.67254700	0.54101800
H	-3.42745700	3.39437600	2.38560700
H	-2.42401900	1.17050200	1.93422300
H	-1.09296400	-1.26610500	-2.47405200
H	-2.39333400	-0.14274100	-2.88996400
H	-1.02411600	1.79900400	-2.26976600
H	-0.03012700	0.83158600	-3.33907800
H	-0.99484100	2.97229200	-0.30735900
H	-0.68916100	5.38035200	0.09377700
H	1.56404800	6.41281500	-0.15230800
H	3.49568000	5.00884700	-0.82945000
H	3.20376700	2.59741100	-1.24158600
H	1.38695300	-0.69211300	-3.48782600
H	3.48256600	-1.69290000	-4.29746500
H	5.55306400	-1.54997700	-2.93247600
H	5.50648100	-0.39348700	-0.73184800
H	3.40432300	0.62647700	0.09005500
C	0.91677200	-2.57753900	-0.23389600
O	-0.03553600	-2.59819200	-0.99277500
O	2.07974800	-3.19100500	-0.48958500
C	2.11948700	-3.96133600	-1.70060800
H	3.16503800	-4.23838500	-1.83399500
H	1.49422300	-4.85223400	-1.59205500
H	1.76417100	-3.36860900	-2.54663600
H	0.52389300	-2.82140100	1.74491200
O	-1.08812600	-0.71079400	2.41421700
H	-0.52458600	-0.48892900	3.17951100

H	-0.14594200	-1.49661200	1.71357000

TS2’-t

C	3.81144500	-0.90108600	2.62898100
C	2.51266500	-0.99228300	1.84025000
C	2.46168400	-2.34094700	1.12519200
C	3.58989800	-3.13250800	1.80032800
C	4.64067000	-2.08329500	2.12565200
C	1.64579300	0.02632900	1.74259200
C	1.75008900	1.33327000	2.46071200
H	0.77059200	1.80695300	2.62384000
C	1.15729600	-3.14131000	1.08512500
H	2.77671800	-2.13757100	0.08306500
H	3.95597000	-3.95158100	1.17170200
H	3.20819300	-3.58333500	2.72927800
H	5.17874500	-1.80012700	1.20985400
H	5.38772700	-2.41947600	2.85247700
H	3.60193500	-1.01186100	3.70533900
H	4.31128900	0.06990900	2.51296200
H	2.21162000	1.20467000	3.45014200
H	2.35053500	2.07316000	1.90908100
Co	0.05789700	-0.33720100	0.67331800
P	-2.08776900	0.04745200	-0.28529000
P	0.83761700	1.05109400	-0.91777500
C	-3.36541500	-1.23948300	-0.22089800
C	-4.35552900	-1.35625000	-1.20696900
C	-5.27984700	-2.39350000	-1.14176500
C	-5.22749400	-3.31335100	-0.09489600
C	-4.25545600	-3.19184000	0.89381300
C	-3.32370100	-2.15973300	0.83479400
C	-2.84980900	1.62792800	0.19174500

C	-3.32514700	2.56593500	-0.73138400
C	-3.81433400	3.79411300	-0.29382400
C	-3.84202400	4.09195500	1.06653800
C	-3.38444600	3.15825700	1.99527500
C	-2.88440100	1.93544400	1.56056100
C	-1.61528100	0.21108200	-2.05657800
C	-0.48738800	1.22565300	-2.21301800
C	1.23567300	2.77248100	-0.50087700
C	0.31347400	3.47117600	0.29237100
C	0.53455100	4.80288300	0.61964200
C	1.68246300	5.44975900	0.16453700
C	2.59677800	4.76581700	-0.63160700
C	2.37647500	3.43304300	-0.97062900
C	2.29317200	0.29312700	-1.67458500
C	2.17426400	-0.53292100	-2.79798500
C	3.28636300	-1.20803600	-3.29111500
C	4.52301800	-1.06907900	-2.66606900
C	4.64778100	-0.25637600	-1.54039700
C	3.53848500	0.41423300	-1.03901300
H	-4.40916300	-0.63823100	-2.02557000
H	-6.04475900	-2.48359700	-1.91018000
H	-5.95138600	-4.12451200	-0.05109400
H	-4.21569900	-3.90707100	1.71265600
H	-2.55200700	-2.07359400	1.59797900
H	-3.31536500	2.34538700	-1.79886700
H	-4.17879200	4.51894900	-1.01854700
H	-4.22457200	5.05247200	1.40490400
H	-3.41224700	3.38558200	3.05885100
H	-2.51131400	1.20919800	2.28627600
H	-1.28646400	-0.79292300	-2.35424100

H	-2.46414000	0.47964600	-2.69874400
H	-0.87403300	2.24493800	-2.08305500
H	-0.03897500	1.18837200	-3.21372200
H	-0.58233400	2.96744000	0.65969000
H	-0.18804100	5.33344500	1.23684600
H	1.86216400	6.48996000	0.42751200
H	3.48707800	5.27204900	-0.99854600
H	3.09121800	2.91329300	-1.60567200
H	1.20754400	-0.66845000	-3.28187500
H	3.18579900	-1.84573900	-4.16651600
H	5.39130400	-1.59642300	-3.05529600
H	5.61277400	-0.14423000	-1.04992200
H	3.63658800	1.03290400	-0.14707800
C	0.33835400	-3.00572200	-0.04587300
O	0.17284900	-1.87832800	-0.64199000
O	-0.36603600	-4.07062900	-0.44778700
C	-1.23123200	-3.89901900	-1.57146300
H	-1.73483000	-4.85811400	-1.69569800
H	-1.96865300	-3.10947300	-1.39399400
H	-0.64936600	-3.66288500	-2.46964900
H	1.24025800	-4.16205900	1.46125700
O	-0.54908000	-1.48434600	2.26187200
H	-0.07455700	-1.12991800	3.03406000
H	0.12250900	-2.32897300	1.94513600

TS3-s

C	-4.95059800	-0.00945800	2.81038500
C	-4.01325200	-0.34945500	1.67280000
C	-3.13570900	0.86099400	1.39242700
C	-3.22112700	1.66960500	2.69347200
C	-4.65770600	1.45527100	3.15816800

C	-3.99899600	-1.50472300	0.99876100
C	-4.92158200	-2.65138100	1.23629900
H	-4.37692100	-3.59995000	1.33971900
C	-1.73509000	0.60686600	0.86803100
H	-3.64622400	1.45312600	0.61331400
H	-2.95541700	2.72603100	2.55594200
H	-2.51392000	1.24432900	3.42869000
H	-5.32363200	2.11760100	2.58913000
H	-4.81190100	1.68627400	4.21793300
H	-4.74984700	-0.66082400	3.67434700
H	-6.00431800	-0.17361800	2.55140700
H	-5.52689100	-2.51547600	2.13998500
H	-5.61529000	-2.78942600	0.39499500
Co	0.56189800	-0.24721400	0.78321000
P	1.01238400	-1.47157400	2.60009700
P	1.72003900	1.38398800	1.77849300
C	-0.54193600	-2.09263800	3.30340200
C	-1.29492300	-1.32409800	4.19835400
C	-2.51024400	-1.79939200	4.68021700
C	-2.99484800	-3.03336100	4.25490300
C	-2.25344300	-3.80262200	3.36016600
C	-1.03320700	-3.33704400	2.88302600
C	2.09147800	-2.92179400	2.52105300
C	2.39996900	-3.59170700	3.71586800
C	3.18896900	-4.73424100	3.68405000
C	3.66284900	-5.22083700	2.46530200
C	3.35068800	-4.56284000	1.27971600
C	2.56544600	-3.41143900	1.29876200
C	1.71044200	-0.33163400	3.88688200
C	1.34684800	1.11755000	3.56681300

C	3.52917700	1.37556800	1.71892700
C	4.23757900	2.54140300	2.04957200
C	5.62470400	2.50958200	2.13677800
C	6.31302900	1.32031600	1.90408700
C	5.61344300	0.16056000	1.58280400
C	4.22520800	0.18191200	1.48638000
C	1.15038200	3.02846400	1.30416800
C	0.01643800	3.60101700	1.89342600
C	-0.50409500	4.79141100	1.39330500
C	0.09955800	5.41376700	0.30330500
C	1.22525300	4.84474100	-0.29135300
C	1.74423000	3.65170700	0.19695200
H	-0.92517900	-0.35790800	4.54289900
H	-3.07835200	-1.20157100	5.39098500
H	-3.94946200	-3.40075800	4.62626000
H	-2.62208600	-4.77502800	3.04031000
H	-0.43624100	-3.96128200	2.21610600
H	2.01269100	-3.22931600	4.66851800
H	3.42974400	-5.25076500	4.61073900
H	4.27690400	-6.11868800	2.44415200
H	3.72038100	-4.94420100	0.33034900
H	2.33693200	-2.86318500	0.38590100
H	1.37171600	-0.62829700	4.88719200
H	2.80032900	-0.46216400	3.86148700
H	1.90182000	1.82489200	4.19597200
H	0.27381300	1.29749300	3.72953000
H	3.70504800	3.47371400	2.23594200
H	6.16919400	3.41733300	2.38753400
H	7.39874100	1.30029300	1.97087300
H	6.14892100	-0.76744300	1.39381400

H	3.68380900	-0.71464100	1.19538700
H	-0.47020900	3.12084500	2.74230300
H	-1.38077100	5.23499100	1.86101600
H	-0.30753900	6.34554900	-0.08337900
H	1.69846000	5.32978000	-1.14256700
H	2.60919700	3.19604400	-0.28393300
C	-1.21452700	1.27503300	-0.29234900
O	-0.01917200	1.04322300	-0.64688400
O	-2.01506800	2.01320200	-1.04135900
C	-1.46066000	2.49084900	-2.28058600
H	-2.25015100	3.08718100	-2.73590800
H	-0.57453800	3.10031500	-2.08298300
H	-1.19069800	1.64892000	-2.92322400
H	-0.99296900	0.71663000	1.70234900
O	2.00865900	-0.78901800	-0.20292600
H	1.55971300	-0.95669600	-1.05199600
H	-3.29304600	-1.62243600	0.17253200
O	-0.67500200	-1.56441700	-0.08475000
H	-0.83903700	-2.35068300	0.46210100
H	-1.44292400	-0.72806900	0.34431800

TS3-t

C	-5.94503700	-1.47114800	1.94425700
C	-4.59354400	-1.48016300	1.26739300
C	-4.10779900	-0.04604800	1.10934600
C	-5.05510500	0.74838500	2.03059100
C	-6.37553100	-0.00257700	1.92606100
C	-3.91662900	-2.56851500	0.88327400
C	-4.37038500	-3.97986800	1.03463500
H	-3.61208100	-4.60096600	1.53478500
C	-2.64953000	0.12214800	1.41971900

H	-4.30063900	0.28307700	0.07428800
H	-5.11439000	1.80752900	1.75566300
H	-4.67490600	0.69791400	3.06392500
H	-6.86165600	0.23169800	0.96874400
H	-7.08776500	0.24880400	2.71938000
H	-5.84275900	-1.82089500	2.98596800
H	-6.67006400	-2.14283900	1.46822800
H	-5.29550800	-4.05586000	1.61703200
H	-4.55370600	-4.45711300	0.06178400
Co	0.69551600	0.19873300	0.92962300
P	1.05137600	-0.91832900	2.95536100
P	2.21111600	1.62750300	1.68917600
C	-0.46920700	-1.28929300	3.85577300
C	-1.03089500	-0.32688000	4.70644300
C	-2.28222700	-0.53957000	5.27402400
C	-2.99138200	-1.70315900	4.98356100
C	-2.44450700	-2.66014600	4.13079500
C	-1.18911000	-2.45823100	3.56712600
C	1.96092100	-2.46038900	2.71602300
C	2.08366700	-3.42599700	3.72577200
C	2.82033000	-4.58096300	3.48952800
C	3.43640300	-4.77974000	2.25385200
C	3.31518000	-3.82594700	1.24631200
C	2.57578800	-2.66989700	1.47376400
C	2.05040200	0.22151600	4.02578400
C	1.93576100	1.65491200	3.50388000
C	3.92364500	1.08118700	1.44474100
C	4.91712600	1.40625700	2.37988200
C	6.22556400	0.98005200	2.18211400
C	6.55362700	0.23210400	1.05237800

C	5.57265700	-0.08617600	0.11755000
C	4.25873600	0.33209000	0.30803900
C	2.03566900	3.29046000	1.02484900
C	1.39319000	4.31082000	1.73699000
C	1.23576700	5.56523500	1.15545800
C	1.70045700	5.80061800	-0.13663900
C	2.32952900	4.78254500	-0.85178000
C	2.49823900	3.52884400	-0.27719900
H	-0.49923900	0.60076700	4.91725600
H	-2.70759400	0.20947600	5.93824300
H	-3.97521100	-1.86310700	5.41962600
H	-3.00201100	-3.56495300	3.89829200
H	-0.76651700	-3.21344900	2.90275500
H	1.59582500	-3.27630200	4.68882900
H	2.91551900	-5.33005600	4.27237400
H	4.01010700	-5.68665200	2.07538200
H	3.79070300	-3.98379700	0.28079700
H	2.47671600	-1.92054200	0.68373300
H	1.73567300	0.14163100	5.07322700
H	3.09101600	-0.12348400	3.97415100
H	2.62791600	2.33216900	4.02024700
H	0.92065100	2.04564300	3.65494500
H	4.67658200	1.99921500	3.26213100
H	6.99172200	1.23527800	2.91102000
H	7.57875500	-0.09979000	0.90161500
H	5.83040000	-0.66258200	-0.76859800
H	3.48395200	0.09190500	-0.41962800
H	1.01499000	4.13406400	2.74276100
H	0.74712900	6.36050600	1.71412800
H	1.57314700	6.78228000	-0.58779400

H	2.69126600	4.96570700	-1.86103100
H	2.97781700	2.72887500	-0.83911100
C	-1.92542600	1.28286800	1.11664900
O	-0.71288900	1.48266900	1.51966700
O	-2.47096300	2.15771100	0.26324700
C	-1.61354000	3.17772500	-0.26192500
H	-2.21652400	3.70471500	-1.00176100
H	-1.29862200	3.86467900	0.52963700
H	-0.72681600	2.73873900	-0.73295100
H	-2.33822500	-0.33159000	2.36218700
O	1.34266400	0.41149400	-0.76014600
H	0.73211800	-0.14327600	-1.28067500
H	-2.95021700	-2.42614600	0.39054200
O	-0.66149200	-1.09080400	0.18434500
H	-0.57269600	-2.01012300	0.48140900
H	-1.64565400	-0.68812800	0.62636400

TS4-s

C	9.96299800	2.69087400	-8.84225000
C	10.35380500	2.20570700	-7.51033300
C	8.45027600	2.86471100	-11.36944500
C	8.21100700	3.83238100	-10.26404500
C	8.53456800	3.23912900	-8.89031000
C	10.13495700	1.29103800	-6.58205300
C	9.66310700	-0.13018900	-6.64524600
H	9.44922600	-0.45162400	-7.67296400
H	7.91098900	1.91300200	-11.33646000
H	7.15858600	4.15093800	-10.26705300
H	8.81331300	4.73870400	-10.42931900
H	7.83321800	2.42808100	-8.64971800
H	8.39927900	4.00725100	-8.11696700

H	10.05454600	1.84706600	-9.55065100
H	10.67251500	3.44868500	-9.21473500
H	10.41775000	-0.80725100	-6.22445300
H	8.75551500	-0.27110800	-6.04720200
Co	11.73079800	2.60921000	-6.33419200
P	12.60183700	4.61658300	-6.45892600
C	11.41016600	5.98050600	-6.53522700
C	11.09736300	6.71190800	-5.38129300
C	10.10742100	7.68971600	-5.42146400
C	9.42147000	7.94471900	-6.60627100
C	9.72308600	7.21744100	-7.75653200
C	10.70611400	6.23528500	-7.72084300
C	13.75797300	4.99766300	-5.11308700
C	13.87016000	4.09294100	-4.05024600
C	14.78674500	4.32500700	-3.02812000
C	15.59089600	5.46079800	-3.06169800
C	15.47620600	6.37268100	-4.11134000
C	14.56346000	6.14481700	-5.13454300
C	13.62971600	4.69208600	-7.99260300
C	14.44377500	3.40159300	-8.04050000
C	14.29130600	0.68624300	-6.88337500
C	13.96724500	0.34489100	-5.56408600
C	14.69373700	-0.63467100	-4.89364200
C	15.74381500	-1.27973600	-5.54214100
C	16.06926800	-0.94829800	-6.85746500
C	15.34646700	0.03019500	-7.53049300
C	12.84244800	1.29282700	-9.31745800
C	12.89695300	2.05361900	-10.49085100
C	12.36580400	1.55260600	-11.67690900
C	11.76944700	0.29380700	-11.69695800

C	11.72010300	-0.47377000	-10.53305300
C	12.25110900	0.02144100	-9.34799100
H	11.63841900	6.52157500	-4.45452700
H	9.87537200	8.25803100	-4.52344100
H	8.65114200	8.71215800	-6.63544100
H	9.19125900	7.41794900	-8.68464000
H	10.92645200	5.66520200	-8.62482900
H	13.21684400	3.21955100	-4.01460100
H	14.86877600	3.62080500	-2.20310700
H	16.30860800	5.64136500	-2.26426100
H	16.09921500	7.26416600	-4.13022900
H	14.46854700	6.86655100	-5.94669400
H	12.94856700	4.75765700	-8.85325700
H	14.26812800	5.58418000	-8.01238700
H	15.19626500	3.39985900	-7.24052100
H	14.98330000	3.27848300	-8.98776200
H	13.12723800	0.83838500	-5.06491000
H	14.43523000	-0.89943700	-3.87090100
H	16.30919300	-2.05057500	-5.02288600
H	16.88653000	-1.45912200	-7.36180800
H	15.59223100	0.27689300	-8.56348100
H	13.34656200	3.04703700	-10.48660300
H	12.40671300	2.15175200	-12.58490200
H	11.32917500	-0.08914500	-12.61490500
H	11.26332000	-1.46116200	-10.55085900
H	12.20800500	-0.57804000	-8.43780600
C	9.30038700	3.05605700	-12.38554100
O	10.65378600	2.89119100	-4.71033100
H	10.08386500	3.67706400	-4.71807000
H	10.11927800	1.95412100	-5.41761400

P	13.31326100	1.97326000	-7.70579700
H	9.86815400	3.98132100	-12.48986800
C	9.49442700	2.02199300	-13.41297600
O	8.98187400	0.91963100	-13.42154100
O	10.35723900	2.45620900	-14.36312000
C	10.58620300	1.53005500	-15.42887000
H	11.18293300	2.06745600	-16.16597500
H	11.13058900	0.65098000	-15.06636100
H	9.63798000	1.19853400	-15.86036800

TS4-t

C	9.97271200	3.06639000	-8.54084700
C	10.24018000	2.41049100	-7.25813600
C	8.62069500	3.40257400	-11.15155200
C	8.42341000	4.37315300	-10.03950100
C	8.60088600	3.73002400	-8.66200600
C	9.82879500	1.48728600	-6.42916400
C	9.19932300	0.14420700	-6.65701300
H	8.18337700	0.14769500	-6.24403600
H	7.97878500	2.51612200	-11.17292200
H	7.41256500	4.80244200	-10.09508800
H	9.12613300	5.21268700	-10.14633600
H	7.82000400	2.97678100	-8.48564200
H	8.48605000	4.49438000	-7.88217000
H	10.05490300	2.25480400	-9.29154800
H	10.76038500	3.78052900	-8.82263500
H	9.14423100	-0.11399400	-7.72067500
H	9.74735800	-0.64111100	-6.12424300
Co	11.79822000	2.26171900	-6.02814100
P	12.43381100	4.39022300	-6.56144700
C	11.24694200	5.71649900	-6.91327700

C	10.25257500	5.95520200	-5.95358100
C	9.28772000	6.93203900	-6.16575200
C	9.29833100	7.67599100	-7.34563500
C	10.28168100	7.44704700	-8.30381200
C	11.25530700	6.47145000	-8.09172600
C	13.50491200	5.03010300	-5.23736000
C	14.01324200	4.15101600	-4.27168900
C	14.87812500	4.61879000	-3.28620600
C	15.23427900	5.96452400	-3.25620800
C	14.72483200	6.84663300	-4.20862200
C	13.86284100	6.38460600	-5.19588800
C	13.54675700	4.26881000	-8.03069200
C	14.45510200	3.04973800	-7.87508900
C	14.67121600	0.29632000	-6.93626900
C	14.69558900	-0.10603100	-5.59536300
C	15.62302500	-1.04986300	-5.16351400
C	16.52576700	-1.59871100	-6.07023400
C	16.50201500	-1.20868400	-7.40931400
C	15.57792200	-0.26598300	-7.84469800
C	12.74281900	0.97857800	-9.01233100
C	12.88943100	1.65830400	-10.22626100
C	12.18561100	1.24369200	-11.35632600
C	11.33298000	0.14493500	-11.28885300
C	11.19904700	-0.55277200	-10.08797400
C	11.89215100	-0.13627500	-8.95806600
H	10.23932100	5.37137300	-5.03197200
H	8.52429800	7.11177100	-5.41209700
H	8.54180700	8.43907800	-7.51413200
H	10.29706200	8.03220300	-9.22112200
H	12.02207900	6.30667900	-8.84838200

H	13.72115200	3.09844300	-4.28000400
H	15.26799700	3.93206400	-2.53824200
H	15.90755600	6.33045100	-2.48400200
H	14.99908600	7.89882900	-4.18004400
H	13.45848400	7.07769700	-5.93414200
H	12.91394800	4.16308400	-8.92385700
H	14.13340400	5.19001000	-8.14857200
H	15.15658900	3.20287400	-7.04338500
H	15.06735900	2.88623000	-8.77163700
H	13.97542300	0.31467600	-4.89006700
H	15.63480700	-1.36186000	-4.12162500
H	17.24797500	-2.33993700	-5.73459600
H	17.20435500	-1.64447100	-8.11654700
H	15.55136500	0.02700200	-8.89459300
H	13.55285800	2.51925200	-10.30331000
H	12.30093200	1.79192000	-12.29011200
H	10.76186100	-0.15850200	-12.16307700
H	10.54570800	-1.42165600	-10.03284100
H	11.77302800	-0.67778300	-8.01775300
C	9.54243900	3.51772500	-12.11568500
O	10.92283400	2.10191800	-4.21399700
H	10.66972400	2.92473700	-3.76896500
H	10.03771200	1.77780200	-5.18075600
P	13.46021700	1.55021400	-7.44028500
H	10.21363300	4.37568500	-12.16496500
C	9.67232400	2.49534000	-13.16519100
O	9.03940900	1.45947000	-13.23306100
O	10.62418600	2.85385100	-14.05916200
C	10.81209600	1.92687600	-15.13287800
H	9.86454600	1.73167400	-15.64219800

H	11.52695300	2.39687900	-15.80827100
H	11.20903900	0.97753500	-14.75633300

TS5-s

C	7.80587500	5.07287800	-15.11254700
C	7.02630100	6.20786800	-15.72906400
C	8.41408500	7.37118300	-16.34951500
C	9.57166500	6.56727200	-15.80015800
C	9.11617400	5.69482700	-14.63991100
C	6.17877600	6.98218800	-14.98163800
C	5.84681400	6.82907400	-13.54429300
H	6.33947200	7.62401400	-12.96291900
H	8.16043500	8.26204700	-15.77525800
H	10.35671600	7.27469100	-15.50294700
H	9.99251200	5.94238300	-16.59759000
H	8.92700000	6.30920800	-13.74833800
H	9.87083500	4.94664100	-14.37029100
H	8.02424800	4.29351700	-15.85500600
H	7.25266400	4.58546400	-14.29805600
H	4.77067500	6.97068400	-13.38098700
H	6.14312300	5.86721600	-13.11389800
Co	6.67364800	6.33131700	-17.70257900
P	4.96139400	4.83692700	-17.67783200
C	3.65413600	5.12310500	-16.45942600
C	2.54880500	5.91792800	-16.79791600
C	1.58372700	6.21876100	-15.84298500
C	1.70770900	5.72854300	-14.54425600
C	2.80235700	4.93870800	-14.20017700
C	3.77771400	4.64580000	-15.14753100
C	4.14929300	4.66727100	-19.29471900
C	4.04372500	5.80152500	-20.11118800

C	3.43403800	5.70986900	-21.35898900
C	2.94726200	4.48595200	-21.80964300
C	3.05635700	3.35197300	-21.00654300
C	3.65027500	3.44033100	-19.75199700
C	5.69386400	3.19441800	-17.31011900
C	6.85395600	2.95915800	-18.27518800
C	7.60691600	4.84147100	-20.37701900
C	7.28175100	6.12942000	-20.81542200
C	7.12010100	6.38811700	-22.17394600
C	7.27582000	5.36114000	-23.09927900
C	7.60300900	4.07421500	-22.66927500
C	7.77094800	3.81320000	-21.31433300
C	9.54708200	4.13999200	-18.37329100
C	9.99036100	3.25838100	-17.37772700
C	11.35133800	3.07740800	-17.15280800
C	12.28496900	3.77971800	-17.91163600
C	11.85448200	4.65531800	-18.90594600
C	10.49510100	4.83663100	-19.13760800
H	2.42871400	6.27551100	-17.82157600
H	0.72549700	6.82775000	-16.11764900
H	0.94634400	5.95616800	-13.80152000
H	2.89902200	4.54760700	-13.18904800
H	4.63098900	4.03194600	-14.85954800
H	4.46328700	6.74945300	-19.76863900
H	3.35734000	6.59477200	-21.98754100
H	2.48214500	4.41218600	-22.79040300
H	2.67443300	2.39556300	-21.35690800
H	3.71542900	2.54921300	-19.12823000
H	6.06088300	3.23506700	-16.27727900
H	4.95371200	2.38564100	-17.35006900

H	6.47574000	2.61372200	-19.24590500
H	7.53105500	2.18120700	-17.90554200
H	7.15966100	6.94701600	-20.10144500
H	6.87096700	7.39555100	-22.50316100
H	7.14903500	5.56043000	-24.16149400
H	7.73390400	3.27395900	-23.39455500
H	8.04467800	2.80967000	-20.98649800
H	9.27591300	2.70513200	-16.76768400
H	11.68318400	2.38241000	-16.38426500
H	13.34857300	3.63823700	-17.73298900
H	12.57997800	5.19705700	-19.50894000
H	10.16462100	5.51293700	-19.92653100
C	8.29919800	7.60165400	-17.76226800
O	5.54564400	7.77658100	-17.82527500
H	4.61826400	7.56977300	-17.62440500
H	5.76662100	7.86740000	-15.46908700
P	7.78586300	4.54421500	-18.58877500
H	9.01231200	7.10163400	-18.42449000
C	7.87770600	8.95837800	-18.16889400
O	7.57476500	9.85371100	-17.40810500
O	7.86914300	9.07953600	-19.51794400
C	7.38948300	10.34285600	-19.98758100
H	7.42953100	10.28779000	-21.07624500
H	6.36440100	10.50691400	-19.64098100
H	8.02177900	11.15665200	-19.62121100

TS5-t

C	7.30521700	5.43629200	-14.95123500
C	6.64830500	6.55414300	-15.70316100
C	8.39077900	7.50123400	-16.47280700
C	9.33027800	6.56965000	-15.76592400

C	8.68554800	5.93949400	-14.53665800
C	5.80827400	7.47705900	-15.19936800
C	5.21315100	7.48004500	-13.83228200
H	5.68347500	8.25453600	-13.20855000
H	8.09485700	8.40229900	-15.93998600
H	10.22200800	7.14881400	-15.48708400
H	9.67182000	5.79046900	-16.45958400
H	8.56436300	6.69427200	-13.74688800
H	9.31489600	5.13882900	-14.12967400
H	7.42356600	4.54277900	-15.59134400
H	6.70735800	5.12331100	-14.08415200
H	4.14467000	7.72459100	-13.86946100
H	5.32035600	6.52057300	-13.31471700
Co	6.72136900	6.37299000	-17.74140400
P	5.04570000	4.76305800	-17.57733900
C	4.03170700	4.65684400	-16.08265500
C	3.06888200	5.65504500	-15.86976500
C	2.27552400	5.63321900	-14.72934100
C	2.44742400	4.62786000	-13.77850400
C	3.41351500	3.64445100	-13.97289000
C	4.20332700	3.65611500	-15.12049900
C	3.85150100	4.75060700	-18.95822200
C	4.26787700	5.18605100	-20.22509900
C	3.41140000	5.10340700	-21.31854200
C	2.12699500	4.59131200	-21.15811800
C	1.70369800	4.15572600	-19.90369600
C	2.55731800	4.23038900	-18.80813000
C	5.88104500	3.12401400	-17.76664100
C	6.74207100	3.15383100	-19.02612800
C	8.00023100	5.09617800	-20.84561000

C	7.55033500	6.32593000	-21.34076600
C	7.70481100	6.62132900	-22.69361300
C	8.30388000	5.69987600	-23.54774300
C	8.75901100	4.47659500	-23.05445200
C	8.61190500	4.17248700	-21.70691800
C	9.41282000	4.18817300	-18.49823000
C	9.54349100	3.24320400	-17.47067700
C	10.79227200	2.96821800	-16.92251100
C	11.92242100	3.63703200	-17.38888300
C	11.80313600	4.57288800	-18.41413500
C	10.55674500	4.85134700	-18.96627200
H	2.93284800	6.44612100	-16.60904400
H	1.52223400	6.40410000	-14.58068100
H	1.82650100	4.61145800	-12.88559700
H	3.54761600	2.85658200	-13.23479100
H	4.94717500	2.87334500	-15.26318200
H	5.26658600	5.60089100	-20.35714800
H	3.75178100	5.44660800	-22.29350700
H	1.45307300	4.53275500	-22.01004700
H	0.70162300	3.75183100	-19.77598600
H	2.21652300	3.88281400	-17.83419200
H	6.50041600	2.95390400	-16.87471700
H	5.13826600	2.31635500	-17.80924200
H	6.10338900	3.16445500	-19.91892500
H	7.38011200	2.26423300	-19.09890600
H	7.08802200	7.04634100	-20.66450400
H	7.35669900	7.57785000	-23.07855100
H	8.42331600	5.93459000	-24.60359600
H	9.23134300	3.75973400	-23.72265300
H	8.98212500	3.22284400	-21.31965100

H	8.66776900	2.71503900	-17.09152000
H	10.88416700	2.22566300	-16.13260900
H	12.89780700	3.42307200	-16.95769800
H	12.68523700	5.08658800	-18.79045600
H	10.47005800	5.57531400	-19.77692100
C	8.39624600	7.64912800	-17.87958600
O	5.73020200	7.54845500	-18.77154600
H	4.81998400	7.24861900	-18.92800000
H	5.54800500	8.32805800	-15.83553000
P	7.77420300	4.69736400	-19.09218400
H	9.12538200	7.09667300	-18.47492400
C	7.92937500	8.94855700	-18.41076900
O	7.39565100	9.81001700	-17.74201600
O	8.18411500	9.05112100	-19.72862000
C	7.67024000	10.24115700	-20.33589800
H	7.99488200	10.20423800	-21.37636000
H	6.57795600	10.25031300	-20.26680100
H	8.06706600	11.13030000	-19.83845800

TS6-s

C	-0.84377400	-0.87420300	3.68340000
C	-0.69337100	-1.30075000	2.29263100
C	-1.05456300	1.41850700	1.66358800
C	-0.96838300	1.55884500	3.15186500
C	-1.64228300	0.40804100	3.88122100
C	-0.52294200	-2.05747100	1.31265900
C	-0.65696200	-3.33405300	0.59979900
H	-0.49804300	-4.16208400	1.30246000
H	-2.08463000	1.38682200	1.29006400
H	-1.44254500	2.51070200	3.43951400
H	0.07671100	1.62800700	3.46851600

H	-2.67660000	0.27475500	3.52730900
H	-1.70797400	0.62324900	4.95398000
H	0.16472600	-0.73162000	4.09982800
H	-1.29957000	-1.70718100	4.23778300
H	-1.66812300	-3.44730400	0.18541900
H	0.06431800	-3.46301000	-0.21426300
Co	-0.00360100	-0.10290100	0.71470600
P	-1.54634600	-0.35112300	-0.90195200
P	1.52211700	-0.79085000	-0.83109700
C	-3.06191000	-1.28254100	-0.51805700
C	-3.69120500	-2.07051600	-1.49055200
C	-4.86714600	-2.75025300	-1.18781800
C	-5.43128700	-2.64156300	0.08109200
C	-4.81936000	-1.84902800	1.04930000
C	-3.63825400	-1.17645400	0.75342000
C	-2.19282900	1.19312600	-1.63045200
C	-3.32647100	1.79536000	-1.06825900
C	-3.78448200	3.01844700	-1.54671000
C	-3.11727500	3.65696300	-2.58890900
C	-1.99135800	3.06606300	-3.15685500
C	-1.52746100	1.84510000	-2.67771300
C	-0.77432300	-1.27192500	-2.31315600
C	0.70179600	-0.92828500	-2.47934900
C	2.89140100	0.38589300	-1.08182400
C	2.56947200	1.71499000	-1.39574700
C	3.57046500	2.66779700	-1.53897200
C	4.90519300	2.30423000	-1.36551700
C	5.23372100	0.98961700	-1.04644900
C	4.23408200	0.03021300	-0.90079100
C	2.34173400	-2.37008000	-0.46000000

C	2.76952500	-3.25374100	-1.45733700
C	3.39975000	-4.44632100	-1.11331800
C	3.61942000	-4.75927900	0.22587100
C	3.21049600	-3.87735200	1.22412300
C	2.57037600	-2.68937400	0.88713800
H	-3.27044000	-2.15552800	-2.49135000
H	-5.34568800	-3.36309000	-1.94862500
H	-6.35009100	-3.17497500	0.31520700
H	-5.25897800	-1.75855800	2.04043300
H	-3.14406700	-0.57691400	1.51727200
H	-3.87040000	1.29284200	-0.26878400
H	-4.67238500	3.46899900	-1.10828300
H	-3.47735900	4.61268400	-2.96274200
H	-1.47030800	3.55595100	-3.97626500
H	-0.64452200	1.39993300	-3.13458400
H	-0.89410500	-2.33971400	-2.08461500
H	-1.33177200	-1.08104000	-3.23925500
H	0.83180900	0.05316700	-2.95230600
H	1.20354300	-1.64814500	-3.13671800
H	1.52730000	2.02057500	-1.50781700
H	3.29976200	3.69622600	-1.76615900
H	5.68982400	3.05002500	-1.47336800
H	6.27469100	0.70478700	-0.90830300
H	4.50129000	-0.99559600	-0.65199200
H	2.62367300	-3.01525000	-2.51019100
H	3.72498000	-5.12941900	-1.89508000
H	4.11288000	-5.69188400	0.49143800
H	3.38457100	-4.11827900	2.27078600
H	2.21734100	-2.01308000	1.66714500
C	0.69506200	3.29726100	1.02268900

O	1.00867200	4.05129200	0.12612700
O	0.99957400	3.47975800	2.31100800
C	1.75642600	4.67150300	2.57911400
H	1.93251100	4.66594400	3.65417800
H	2.70120500	4.64825000	2.02921600
H	1.18945800	5.55739800	2.28165000
C	-0.09509700	2.03357300	0.74982700
H	-0.44711600	2.15124700	-0.27306700
O	1.55185100	0.34371500	1.94124500
H	0.99443800	1.28771400	1.03756900
H	2.41048300	0.15914600	1.52594400

TS6-t

C	-0.10335500	-0.53682300	3.95283400
C	-0.24464600	-1.11558400	2.61872200
C	-0.94947700	1.61034100	1.84312000
C	-0.67675400	1.82150400	3.29158500
C	-1.04097200	0.64705300	4.18849800
C	-0.38452300	-1.92528500	1.66602900
C	-0.66424200	-3.22768000	1.05118100
H	-1.66845100	-3.57769100	1.32108900
H	-1.97283900	1.31388900	1.58581100
H	-1.25365300	2.70855800	3.60072500
H	0.37769100	2.08445600	3.43322500
H	-2.08451400	0.33865300	4.02583000
H	-0.96513500	0.95121000	5.23854800
H	0.93806800	-0.20156400	4.06732700
H	-0.28096900	-1.32548700	4.69704400
H	-0.59671300	-3.21054900	-0.04160900
H	0.06643200	-3.96520100	1.40792000
Co	0.11155900	-0.10507500	0.91864500

P	-1.60261000	-0.38718200	-0.89983900
P	1.57912000	-0.78286500	-0.85483400
C	-3.01623100	-1.43423000	-0.44309400
C	-3.57097200	-2.36653400	-1.32876700
C	-4.64059600	-3.16310800	-0.93135400
C	-5.17558300	-3.02840500	0.34789600
C	-4.64018800	-2.09441400	1.23248600
C	-3.56199900	-1.30643700	0.84144600
C	-2.34144700	1.05354000	-1.74538000
C	-3.71956800	1.30207400	-1.71429600
C	-4.24078100	2.44155000	-2.32132300
C	-3.39874300	3.34869900	-2.95827400
C	-2.02524200	3.11666500	-2.98797400
C	-1.50049200	1.98075800	-2.38383100
C	-0.76306600	-1.33382500	-2.26407200
C	0.68257700	-0.88944200	-2.46262300
C	3.01467100	0.27822800	-1.19889100
C	2.79604600	1.66236100	-1.27578600
C	3.85304500	2.52771200	-1.53013800
C	5.13983800	2.01930700	-1.70127700
C	5.36540000	0.64703700	-1.62032000
C	4.30981900	-0.22531800	-1.36825900
C	2.24478200	-2.42781000	-0.48040300
C	2.18437900	-3.52014500	-1.35133300
C	2.68277100	-4.76001400	-0.95551500
C	3.25229600	-4.91581000	0.30535500
C	3.31872600	-3.83084000	1.17942600
C	2.80815100	-2.59712300	0.79531900
H	-3.17232800	-2.47151100	-2.33682500
H	-5.06124200	-3.88706500	-1.62585400

H	-6.01322100	-3.65090100	0.65493400
H	-5.05830600	-1.98468300	2.23088000
H	-3.12511300	-0.59623600	1.54570200
H	-4.39121300	0.59824300	-1.22533200
H	-5.31411700	2.61822100	-2.29572500
H	-3.81092500	4.24020000	-3.42595000
H	-1.35633700	3.82597900	-3.47026000
H	-0.41952800	1.83177200	-2.39281100
H	-0.79621300	-2.39541300	-1.97999300
H	-1.33010000	-1.23640900	-3.19845600
H	0.72645900	0.11673700	-2.89950600
H	1.20717200	-1.53960100	-3.17482600
H	1.79632600	2.07584100	-1.12623300
H	3.66627500	3.59860400	-1.57822600
H	5.97053200	2.69565500	-1.89159200
H	6.36974900	0.25030100	-1.75275900
H	4.49197000	-1.29734700	-1.30257800
H	1.75325500	-3.41422500	-2.34623700
H	2.63162300	-5.60459400	-1.63940200
H	3.64412900	-5.88415300	0.60919600
H	3.76291900	-3.94923700	2.16547000
H	2.83274600	-1.75382000	1.48938100
C	0.37642600	3.65619400	0.84836100
O	0.82437000	4.19638400	-0.14228700
O	0.32295300	4.21173100	2.06423500
C	0.86010100	5.54343400	2.13412500
H	0.74260700	5.84854100	3.17350600
H	1.91446500	5.54103900	1.84620700
H	0.30650200	6.21004800	1.46810700
C	-0.15715700	2.24828000	0.80820800

H	-0.55070000	2.10977100	-0.19837500
O	1.77418200	0.81101000	1.82917900
H	0.97014900	1.69814600	1.05405200
H	2.58281300	0.68909700	1.30517800

TS7-s

C	-2.40753400	-2.29299600	2.03116400
C	-1.07631200	-2.00674200	1.44602200
C	-0.88233600	0.02234800	2.31191600
C	-2.37453200	0.12911100	2.45210200
C	-2.92310700	-1.19507000	2.95879300
C	-0.14990600	-2.44958800	0.68642300
C	0.60154400	-3.57119100	0.12067700
H	1.64370300	-3.57195900	0.46601900
H	-0.45433900	0.91025600	1.79354800
H	-2.83493200	0.39229500	1.48900000
H	-2.62094800	0.94403100	3.15044600
H	-4.01822800	-1.19625500	2.99220600
H	-2.57789100	-1.37190100	3.98553400
H	-2.35061500	-3.26101500	2.54707400
H	-3.11026400	-2.43526400	1.19730200
H	0.15019100	-4.52808200	0.41452600
H	0.62916400	-3.53560200	-0.97787500
Co	0.13156000	-0.54193100	0.59024000
P	-1.45428000	0.07533400	-0.97359200
P	1.53218200	-0.37260800	-1.13038300
C	-2.87968900	-1.04562100	-1.11603700
C	-2.65312600	-2.34782100	-1.58344300
C	-3.68281900	-3.28118400	-1.60078500
C	-4.95295100	-2.92557800	-1.14769400
C	-5.18904700	-1.63399900	-0.68405000

C	-4.15748200	-0.69845400	-0.66122500
C	-2.15334500	1.75833400	-0.93992300
C	-3.14483500	2.13007600	-1.86177800
C	-3.62447200	3.43431100	-1.88877900
C	-3.11687400	4.38527500	-1.00409400
C	-2.12707700	4.02934500	-0.09371900
C	-1.64872200	2.72235600	-0.06052500
C	-0.66815200	0.01725400	-2.66910200
C	0.61923900	-0.79564000	-2.66833800
C	1.97976400	1.38107100	-1.31426100
C	2.42851100	1.90306300	-2.53441800
C	2.73141400	3.25509700	-2.64504400
C	2.59390600	4.09434200	-1.53958400
C	2.16095300	3.57983700	-0.32093000
C	1.85597200	2.22580900	-0.20370100
C	3.09895600	-1.28339200	-1.16402700
C	3.26926800	-2.43752900	-1.93930000
C	4.47010200	-3.13875700	-1.89461800
C	5.50571500	-2.70167300	-1.07209200
C	5.34539400	-1.55419000	-0.29840500
C	4.14974500	-0.84498900	-0.34259400
H	-1.65829800	-2.63658700	-1.92772000
H	-3.49617400	-4.28771600	-1.96947800
H	-5.75891200	-3.65607100	-1.16069700
H	-6.18067600	-1.35005900	-0.33786500
H	-4.35057300	0.30906300	-0.29444900
H	-3.54876400	1.39168700	-2.55472900
H	-4.39673800	3.71004000	-2.60380500
H	-3.49384200	5.40549800	-1.02855200
H	-1.72490100	4.76825100	0.59620500

H	-0.86597300	2.46185100	0.65147500
H	-1.39077200	-0.36382000	-3.40051400
H	-0.44922600	1.05771600	-2.94000100
H	1.21661800	-0.61459300	-3.57147900
H	0.40391200	-1.87261300	-2.63810500
H	2.54864600	1.25114400	-3.39994500
H	3.07962000	3.65563500	-3.59456100
H	2.83064800	5.15239100	-1.62988300
H	2.06183700	4.23612300	0.54187200
H	1.53504700	1.81272000	0.75695900
H	2.47013000	-2.79061300	-2.59065000
H	4.59788100	-4.02793300	-2.50815200
H	6.44227300	-3.25379300	-1.03830100
H	6.15512200	-1.20593700	0.33883900
H	4.03325100	0.06338600	0.25025300
C	1.08019300	0.60078500	3.87945000
O	1.94938200	0.30426900	4.66064600
O	1.05116000	1.78587100	3.20837600
C	2.20581200	2.59964200	3.43079800
H	1.98617500	3.56420000	2.96966500
H	2.39577000	2.71772900	4.50039300
H	3.08362400	2.13836200	2.96292300
O	1.59427400	-0.75577500	1.71756900
H	2.35572600	-1.17715300	1.28953900
C	-0.14607600	-0.23704200	3.60836900
H	0.18804900	-1.27686700	3.68614600
H	-0.83305300	-0.07181400	4.45376700

TS7-t

C	2.10904000	-1.67162400	2.47377700
C	0.83296800	-1.50903100	1.73299900

C	1.76301400	-1.81447500	-0.32948200
C	3.12184700	-1.57472000	0.25047500
C	3.26982100	-2.17977900	1.62952500
C	-0.43937700	-1.42184000	1.91235100
C	-1.50110400	-1.61995200	2.91537300
H	-2.30715600	-2.26281000	2.54199500
H	1.62459300	-1.25536600	-1.25546900
H	3.32522400	-0.49433500	0.28039500
H	3.84776100	-2.00483100	-0.45674700
H	4.22574700	-1.90956000	2.09130200
H	3.25146900	-3.27555300	1.57118300
H	1.91648100	-2.31564200	3.34279600
H	2.36224300	-0.67994700	2.87954500
H	-1.08493000	-2.08291000	3.81967200
H	-1.97489500	-0.67334900	3.20610900
Co	-0.11292500	-0.68249600	0.15843400
P	0.85368200	1.54086000	0.32135300
P	-2.10890100	0.45854500	0.18740800
C	1.90663700	1.76744200	1.78787300
C	1.32443000	1.83349100	3.06218100
C	2.12053200	1.88964800	4.20148700
C	3.50932900	1.87284200	4.08567100
C	4.09804700	1.81472500	2.82455400
C	3.30409700	1.76341000	1.68190700
C	1.78843100	2.26013100	-1.05833800
C	2.33736500	3.54751500	-0.96030400
C	3.03108000	4.09188400	-2.03421600
C	3.18616700	3.35744600	-3.20974400
C	2.64519900	2.07880800	-3.31426000
C	1.94811400	1.52737700	-2.24131100

C	-0.56930100	2.70856200	0.61379200
C	-1.80140900	1.99289500	1.16412200
C	-2.64602600	1.10193200	-1.43452100
C	-4.00170500	1.27213300	-1.74739500
C	-4.36936600	1.83775000	-2.96469000
C	-3.39640000	2.24191700	-3.87629400
C	-2.04717000	2.07436100	-3.57451600
C	-1.67419600	1.50143800	-2.36372200
C	-3.58725800	-0.32092700	0.88265200
C	-4.36392400	0.28130300	1.87821000
C	-5.46765200	-0.38601700	2.40338200
C	-5.80754200	-1.65156600	1.93377400
C	-5.04421100	-2.25367900	0.93403500
C	-3.93545600	-1.59600800	0.41353000
H	0.23953200	1.83716400	3.17381600
H	1.65466000	1.94966700	5.18295500
H	4.13102900	1.91262700	4.97716300
H	5.18157200	1.81674200	2.72610200
H	3.77365000	1.73299200	0.69932500
H	2.23119000	4.11762500	-0.03696800
H	3.45487200	5.09066500	-1.95379500
H	3.73306100	3.78575800	-4.04714900
H	2.76916000	1.50391700	-4.22939100
H	1.50860400	0.53144100	-2.32738700
H	-0.25930700	3.52732200	1.27535300
H	-0.80799800	3.15507800	-0.36027800
H	-2.67572700	2.65620400	1.13224700
H	-1.65619300	1.68962900	2.21082500
H	-4.77110600	0.96053700	-1.04230500
H	-5.42391300	1.96218500	-3.20158200

H	-3.69188200	2.68072800	-4.82693800
H	-1.28034800	2.37960100	-4.28401200
H	-0.61982300	1.33801500	-2.14367500
H	-4.11294600	1.27230400	2.25420300
H	-6.06411000	0.08749800	3.18020100
H	-6.66939600	-2.17115500	2.34689000
H	-5.30928500	-3.24205100	0.56471200
H	-3.32596600	-2.07715100	-0.35364200
C	2.06157300	-3.88612200	-1.67460100
O	2.87247600	-3.33134800	-2.38556600
O	1.67770000	-5.16466100	-1.81663800
C	2.29911400	-5.85927700	-2.90698300
H	1.89259600	-6.87001600	-2.87893100
H	2.05910100	-5.37054900	-3.85544100
H	3.38531800	-5.87441300	-2.78055300
O	-0.44382400	-1.23665200	-1.60704100
H	-1.28262900	-0.93187100	-1.98708000
C	1.33287100	-3.24584900	-0.51111300
H	0.26079600	-3.29578500	-0.73725800
H	1.48994800	-3.85965500	0.38559100

TS8-s

C	-3.33876200	-1.45589000	-1.97669400
C	-2.26305500	-1.33900000	-0.92932600
C	-2.91546600	-1.34619600	0.43234900
C	-4.34997000	-0.88837900	0.15125700
C	-4.66834300	-1.50128500	-1.21538200
C	-0.92171500	-1.30058500	-1.17750700
C	-0.43249100	-1.43935500	-2.58967300
H	0.59785100	-1.80744200	-2.64517300
H	-2.37886300	-0.69497600	1.13527200

H	-4.38655500	0.20731900	0.09229300
H	-5.04807500	-1.19460200	0.93919900
H	-5.47323800	-0.97620100	-1.74052400
H	-5.00087900	-2.54080400	-1.09742900
H	-3.18209700	-2.32175700	-2.63531300
H	-3.27027700	-0.57659300	-2.63836900
H	-1.04637000	-2.14524800	-3.16653800
H	-0.48123800	-0.48128200	-3.13093600
Co	0.10643900	-0.25352900	0.28761000
P	0.00065900	1.77325300	-0.62490300
P	2.08158800	-0.43825900	-0.65321100
C	-1.41561100	2.02378900	-1.72845200
C	-1.29468000	2.10815800	-3.11888700
C	-2.43000700	2.24405600	-3.91583200
C	-3.69243200	2.30124400	-3.33121800
C	-3.82266800	2.20914900	-1.94564700
C	-2.69332400	2.05958800	-1.14912900
C	-0.00814900	3.15951200	0.55605400
C	-0.85038700	4.27240500	0.45448500
C	-0.73724700	5.31430200	1.37212600
C	0.21182900	5.25733800	2.38938300
C	1.05653300	4.15374500	2.49382700
C	0.94659300	3.10779900	1.58593100
C	1.55762900	2.07266700	-1.58386100
C	2.18452500	0.76287200	-2.04127900
C	3.17740800	0.20030300	0.65184500
C	4.26214100	1.03879500	0.36264400
C	5.05169900	1.53757300	1.39370100
C	4.77006800	1.19776100	2.71562800
C	3.70064400	0.35348000	3.00773400

C	2.89998700	-0.14228500	1.98391700
C	2.79687000	-1.99574300	-1.23289200
C	3.32534500	-2.12247800	-2.52533500
C	3.89929800	-3.32413800	-2.92742600
C	3.94547900	-4.40364400	-2.04921200
C	3.41594000	-4.28335000	-0.76572900
C	2.84167400	-3.08682700	-0.35274100
H	-0.31330000	2.07002900	-3.59169900
H	-2.32417800	2.31429900	-4.99609000
H	-4.57612300	2.41660000	-3.95499000
H	-4.80794000	2.25308400	-1.48442400
H	-2.79791800	1.98493500	-0.06497800
H	-1.58654700	4.33463100	-0.34505900
H	-1.39300500	6.17799900	1.28609800
H	0.29409400	6.07505800	3.10209800
H	1.80013400	4.10021200	3.28637500
H	1.60354500	2.24053700	1.67470500
H	1.38817500	2.77114600	-2.41228800
H	2.22789800	2.58131500	-0.87825700
H	3.21612000	0.91135400	-2.38489800
H	1.62760100	0.32766700	-2.88060500
H	4.50001200	1.30326700	-0.66749700
H	5.89101800	2.18997400	1.16247100
H	5.38946100	1.59048400	3.51950000
H	3.48034800	0.08498600	4.03854000
H	2.04242400	-0.77430500	2.21374400
H	3.29905700	-1.28598900	-3.22256400
H	4.31361600	-3.41460600	-3.92919000
H	4.39412300	-5.34243000	-2.36710900
H	3.44796000	-5.12780700	-0.08073800

H	2.40801200	-2.99659800	0.64079400
C	-2.78535800	-2.76716700	2.49194900
O	-1.74993400	-2.50383900	3.07789000
O	-3.94818900	-3.01700400	3.10141200
C	-3.90372300	-2.96668700	4.53736000
H	-4.91569300	-3.19821800	4.86767800
H	-3.19189600	-3.70306400	4.91888100
H	-3.60064000	-1.97070700	4.87120300
C	-2.89919800	-2.79931500	0.99450700
H	-2.02370400	-3.34411400	0.62390200
H	-3.79630800	-3.34164400	0.67610800
O	-0.40140300	0.30427800	1.94251500
H	-0.59886800	1.24957200	2.05015300
O	0.25004100	-2.07392900	0.99519400
H	-0.39399200	-1.98967200	-0.18858500
H	-0.37190400	-2.08535300	1.76354300

TS8-t

C	-2.98963400	-3.24381300	-0.06978700
C	-2.03588000	-2.29810900	0.61023100
C	-2.81715100	-1.39358900	1.54210900
C	-4.21631100	-2.02426000	1.59454600
C	-4.38634600	-2.69859700	0.24036500
C	-0.69598900	-2.25995700	0.38565300
C	-0.03388200	-3.17881500	-0.60878500
H	1.06424500	-3.09843400	-0.58006300
H	-2.89711800	-0.41998400	1.02265500
H	-4.99541800	-1.29159000	1.82970800
H	-4.23558500	-2.78127700	2.39286800
H	-4.66655800	-1.95705500	-0.51880400
H	-5.15890800	-3.47406500	0.23495700

H	-2.86155800	-4.25426400	0.35290400
H	-2.76937200	-3.33824100	-1.14347900
H	-0.26765200	-4.23324500	-0.39886300
H	-0.35369800	-2.98522200	-1.64285500
Co	0.35349500	-0.47641700	0.57959800
P	-0.45056900	0.50606200	-1.30256100
P	2.45352500	-0.14250600	-0.35735600
C	-1.65764600	-0.41892900	-2.31131100
C	-1.23277600	-1.30751000	-3.30771600
C	-2.16090200	-1.99852600	-4.08063400
C	-3.52417100	-1.80692400	-3.86922700
C	-3.95480900	-0.92269300	-2.88299100
C	-3.02989900	-0.23631900	-2.10216700
C	-1.19992500	2.15001600	-1.12046300
C	-0.99916400	3.12532800	-2.10763200
C	-1.63474800	4.35860500	-2.01412200
C	-2.48392800	4.62583000	-0.94344000
C	-2.69667400	3.65656100	0.03293400
C	-2.05793800	2.42240400	-0.04589700
C	0.99620500	0.74962100	-2.44559000
C	2.11940100	-0.24559600	-2.16442500
C	3.13606300	1.51717600	-0.10785800
C	4.45833700	1.80089700	-0.48222000
C	4.94431300	3.09780500	-0.36970700
C	4.11902600	4.11430000	0.11137700
C	2.80854800	3.83438400	0.48815100
C	2.31125500	2.53726600	0.38555900
C	3.74507900	-1.32869500	0.07051200
C	4.15361500	-2.35538400	-0.79095700
C	5.10583100	-3.27927500	-0.36918000

C	5.64797800	-3.19293300	0.91089400
C	5.23979900	-2.17878700	1.77651200
C	4.29067100	-1.25155500	1.36335300
H	-0.16919800	-1.45735000	-3.49463800
H	-1.81660600	-2.67876200	-4.85670600
H	-4.25044800	-2.33837500	-4.48021800
H	-5.01876200	-0.75687300	-2.72488400
H	-3.37778400	0.46943200	-1.34719300
H	-0.35771600	2.92603600	-2.96451000
H	-1.47029400	5.10865800	-2.78452300
H	-2.98138500	5.59075500	-0.87124500
H	-3.36112700	3.86523100	0.86864700
H	-2.20187100	1.68277100	0.73891100
H	0.66755400	0.70710300	-3.49091600
H	1.36781000	1.76653800	-2.26111000
H	3.01286200	-0.01418900	-2.75925800
H	1.81345500	-1.27458300	-2.40350800
H	5.10669600	1.00675400	-0.85374100
H	5.97095000	3.31629900	-0.65549500
H	4.50589200	5.12737200	0.20038500
H	2.16977300	4.62460400	0.87695300
H	1.29662900	2.30554400	0.71573400
H	3.74132500	-2.43006300	-1.79673500
H	5.42860200	-4.06685500	-1.04666800
H	6.39133100	-3.91746000	1.23566100
H	5.66453700	-2.10746000	2.77524600
H	3.98177900	-0.45075200	2.03673300
C	-2.32057000	0.21901900	3.51091700
O	-1.58726300	0.65962600	4.36880300
O	-3.36893200	0.90477800	3.00264800

C	-3.51799700	2.22023100	3.54919000
H	-4.41152300	2.63556000	3.08087300
H	-3.63825300	2.17291900	4.63453200
H	-2.63417800	2.82459700	3.31882100
C	-2.18586800	-1.15695800	2.91859100
H	-1.11702700	-1.37970200	2.94002500
H	-2.63148700	-1.85737100	3.64117200
O	-0.22321100	0.89138200	1.66190900
H	0.08185900	0.66578600	2.56192100
O	0.97866300	-1.53359600	2.16654700
H	1.82607900	-1.97706400	1.97976400
H	0.10590700	-2.13665400	1.50659800

MECP1

C	10.19151512	1.24523873	-3.84179511
C	11.06127687	1.79546667	-4.89818079
C	10.92145305	4.32344581	-3.63587228
C	10.40387334	3.40115192	-2.56942353
C	9.44626603	2.32507307	-3.06411255
C	11.67167438	1.70659218	-6.02987280
C	11.91526216	0.92678962	-7.25407885
H	12.35396007	1.51813055	-8.06615570
H	10.17539168	4.99498990	-4.07411027
H	9.88881182	4.02457311	-1.82341091
H	11.24695348	2.92811091	-2.04259371
H	8.67617157	2.78368984	-3.70437958
H	8.91358164	1.87051056	-2.22044880
H	10.80857823	0.65742073	-3.14536416
H	9.48982211	0.52889710	-4.29596732
H	12.60152179	0.09386789	-7.05432991
H	10.96791564	0.49788783	-7.60859545

Co	11.87756503	3.46387885	-5.30298286
P	10.66333124	4.49368485	-6.98079199
P	13.74677899	4.17005489	-6.46656718
C	9.50787348	3.34076023	-7.80239535
C	9.40400351	3.28014119	-9.19751173
C	8.51062992	2.39842181	-9.80050505
C	7.70152091	1.57701740	-9.02135795
C	7.78388801	1.64126421	-7.63212057
C	8.68328721	2.51249174	-7.02863684
C	9.62956465	5.95478359	-6.65541658
C	10.02612946	7.24748171	-7.01753414
C	9.21093249	8.33576669	-6.72895761
C	8.00295101	8.15103850	-6.06062293
C	7.60777094	6.87017411	-5.68155651
C	8.41509289	5.77796407	-5.97727086
C	11.86469273	4.95333368	-8.30694941
C	13.19233019	5.40603957	-7.71047451
C	15.10161495	4.97529141	-5.54780351
C	15.65558343	6.19566015	-5.94952060
C	16.67644127	6.78086419	-5.20650044
C	17.16821476	6.14898564	-4.06833120
C	16.63064106	4.92844215	-3.66555162
C	15.59344945	4.35270158	-4.39150166
C	14.58656710	2.85372554	-7.41607146
C	14.71641058	2.87957731	-8.80937250
C	15.32251044	1.82008523	-9.48174233
C	15.81501282	0.72961836	-8.77277075
C	15.69658407	0.69646783	-7.38428479
C	15.07745109	1.74392766	-6.71333889
H	10.01148100	3.92635822	-9.82830606

H	8.44428876	2.36090128	-10.88589093
H	7.00339387	0.89058574	-9.49511778
H	7.14842079	1.00758079	-7.01600623
H	8.75784467	2.54148347	-5.94158824
H	10.98284110	7.41291692	-7.50882118
H	9.52626428	9.33495862	-7.02167486
H	7.36865936	9.00589883	-5.83653311
H	6.66357092	6.71944563	-5.16267198
H	8.08549409	4.77737968	-5.69659672
H	12.01072834	4.04093420	-8.90441535
H	11.44159487	5.71121678	-8.97915965
H	13.07038535	6.34911306	-7.16627906
H	13.94644923	5.56401269	-8.49140355
H	15.29236020	6.70422438	-6.84045419
H	17.09186624	7.73519078	-5.52326821
H	17.97092810	6.60657474	-3.49399303
H	17.01336336	4.42955296	-2.77658516
H	15.15821799	3.40956042	-4.05478885
H	14.34935779	3.72617935	-9.38758156
H	15.41377167	1.85295107	-10.56558488
H	16.29366944	-0.09284944	-9.29962836
H	16.08098413	-0.15356498	-6.82405380
H	14.96341429	1.69191556	-5.62979267
C	12.25835893	4.75494453	-3.67282351
C	12.60345353	6.12076828	-4.12704657
H	12.95410169	4.26558685	-2.98579301
O	12.18266705	6.61135104	-5.16258245
O	13.41436845	6.87062005	-3.36017651
C	13.78968042	6.43705091	-2.05258024
H	14.50743556	5.60994309	-2.10340211

H	14.27022337	7.30022734	-1.59043403
H	12.91455495	6.14376705	-1.46229254

MECP2

C	6.52775462	-0.59103271	-0.23585924
C	5.15885515	-0.47221167	-0.85987100
C	4.27119758	-1.59224823	-0.33316306
C	5.08067494	-2.15107203	0.86701932
C	6.52727721	-1.97111464	0.42369358
C	4.75830722	0.45458078	-1.73995179
C	5.59125927	1.55650734	-2.29546264
H	5.13332968	2.54177499	-2.12581532
C	2.91295652	-1.14208546	0.03257377
H	4.20493370	-2.39445011	-1.08429277
H	4.79919318	-3.18270360	1.10373970
H	4.87726926	-1.53885609	1.75838689
H	6.78428294	-2.74002472	-0.31753734
H	7.24434288	-2.06164962	1.24651443
H	6.66530634	0.19431455	0.52547292
H	7.34009710	-0.45674561	-0.96039790
H	6.59351815	1.58079994	-1.85470893
H	5.70915232	1.46288983	-3.38349143
Co	-0.61258909	-0.06199161	-0.66353509
P	-0.43666705	1.56752428	0.87039566
P	-2.33190112	-0.77362680	0.47622363
C	-1.66851152	1.23947448	2.21601533
C	-2.02833576	-0.24556746	2.21388418
H	-1.29566129	1.57144489	3.19278925
H	-2.55345473	1.84653918	1.98326989
H	-2.88364681	-0.45506657	2.86896399
H	-1.18227143	-0.84606842	2.57353787

C	1.72023650	-1.90060874	-0.08535989
O	0.59683635	-1.50393737	0.31947123
O	1.85262064	-3.10046580	-0.67340974
C	0.62637803	-3.81974107	-0.87872891
H	0.89432722	-4.66759815	-1.50902798
H	0.22554286	-4.16866080	0.07894636
H	-0.11702174	-3.17671095	-1.36592880
H	2.82124433	-0.22391800	0.61407659
O	-1.22819627	-0.97538341	-2.14530331
H	-0.51017564	-0.89716905	-2.79605100
H	3.73593841	0.38391273	-2.12812992
O	1.03531546	0.56767144	-1.79838870
H	1.07047949	1.52687381	-1.94118520
H	1.85525613	0.34239589	-1.30792345
C	-3.90258425	-0.01393025	-0.01882627
C	-5.01461449	-0.02113903	0.83357672
C	-4.00219533	0.56977498	-1.28786906
C	-6.20861053	0.56008114	0.42278006
H	-4.95419123	-0.49168759	1.81546576
C	-5.20257097	1.14602800	-1.69426154
H	-3.14115117	0.53420104	-1.95684172
C	-6.30200614	1.14440665	-0.84043091
H	-7.07082331	0.55174257	1.08632308
H	-5.28056074	1.59105670	-2.68416168
H	-7.24028556	1.59214920	-1.16159964
C	-2.57621183	-2.56353625	0.48201739
C	-2.28927754	-3.35635956	1.59925560
C	-3.01515411	-3.16721676	-0.70559175
C	-2.45044431	-4.73757937	1.53202560
H	-1.94149963	-2.90523366	2.52720713

C	-3.16848296	-4.54713142	-0.76500207
H	-3.21151442	-2.55355469	-1.58257323
C	-2.88678145	-5.33381189	0.35132996
H	-2.23510108	-5.34786254	2.40638302
H	-3.51150235	-5.01089706	-1.68724825
H	-3.01076964	-6.41378697	0.30167774
C	-0.75954193	3.23287941	0.22386298
C	-0.51547450	4.38951796	0.97770381
C	-1.27887375	3.35130877	-1.07222466
C	-0.79137360	5.64117474	0.43820603
H	-0.10182159	4.30961448	1.98346373
C	-1.55333587	4.60681527	-1.60852852
H	-1.47671522	2.44738192	-1.65532949
C	-1.30954778	5.75035454	-0.85281833
H	-0.59781363	6.53692143	1.02521616
H	-1.95647977	4.69064048	-2.61582453
H	-1.52074245	6.73239826	-1.27139599
C	1.20827022	1.65690480	1.63833679
C	2.22998199	2.37399676	0.99513352
C	1.52870492	0.86689242	2.74961230
C	3.54037135	2.30429941	1.45917818
H	1.99489131	3.00689473	0.13753419
C	2.83897748	0.80673675	3.21531291
H	0.75563244	0.28724517	3.25379065
C	3.84722383	1.51982837	2.56955312
H	4.32522027	2.86497644	0.95482663
H	3.07444401	0.19386613	4.08298216
H	4.87137599	1.46711088	2.93389497

3.7 NMR Spectra

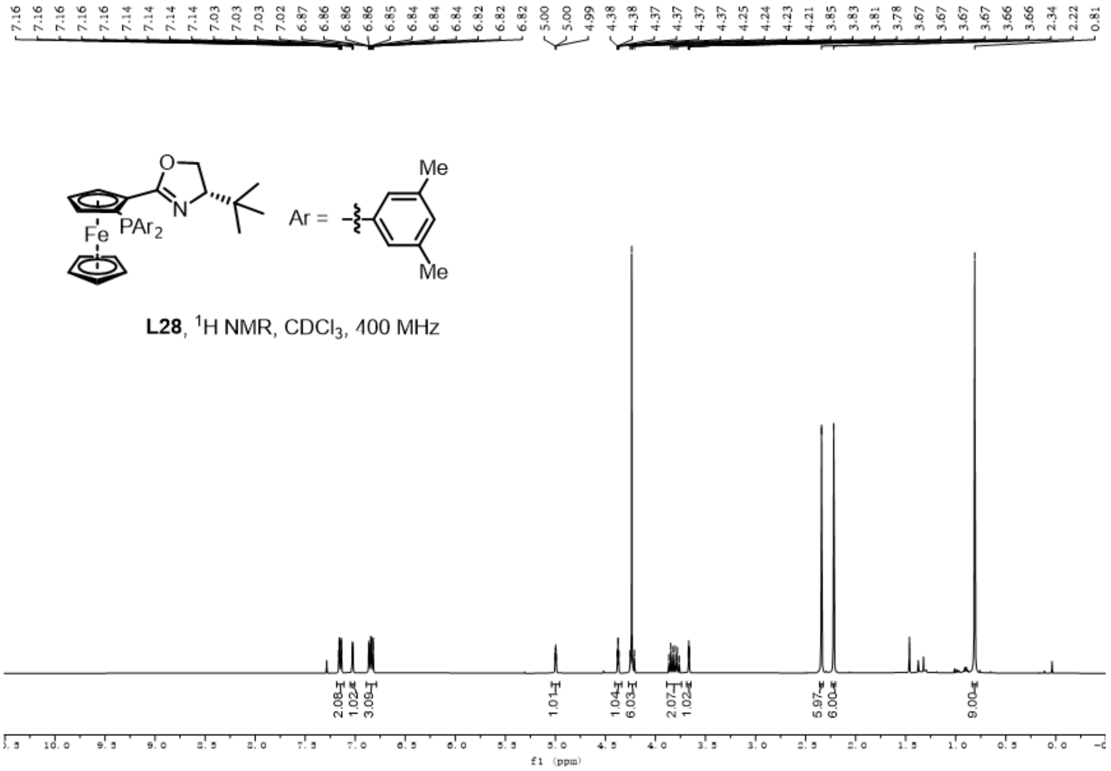

Supplementary Figure 6. ^{1}H NMR (400 MHz, $CDCl_3$, 25 °C) spectra for **L28**

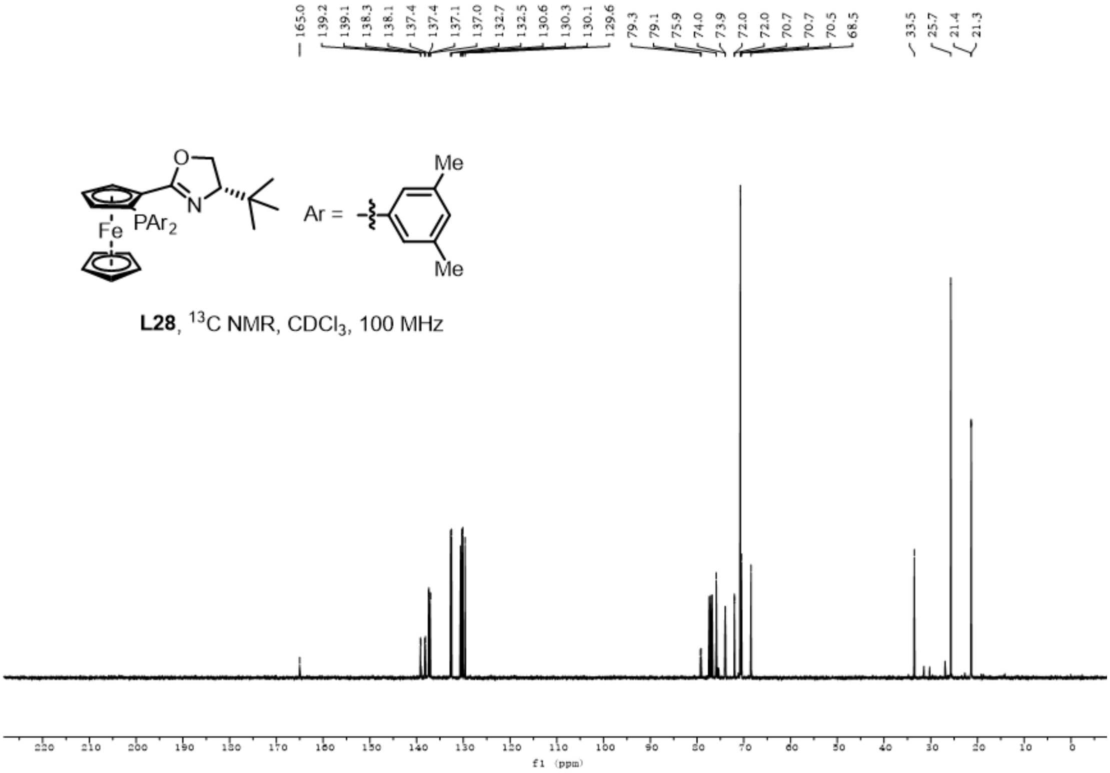

Supplementary Figure 7. ^{13}C NMR (400 MHz, $CDCl_3$, 25 °C) spectra for **L28**

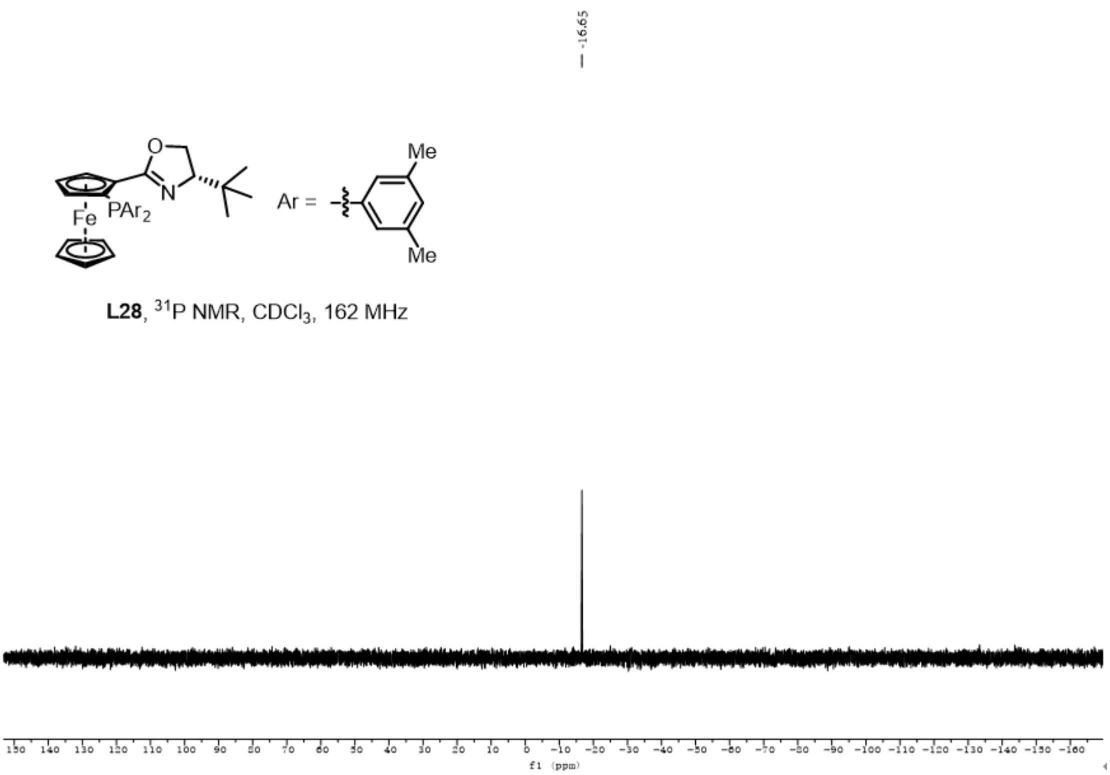

Supplementary Figure 8. ^{31}P NMR (400 MHz, $CDCl_3$, 25 °C) spectra for **L28**

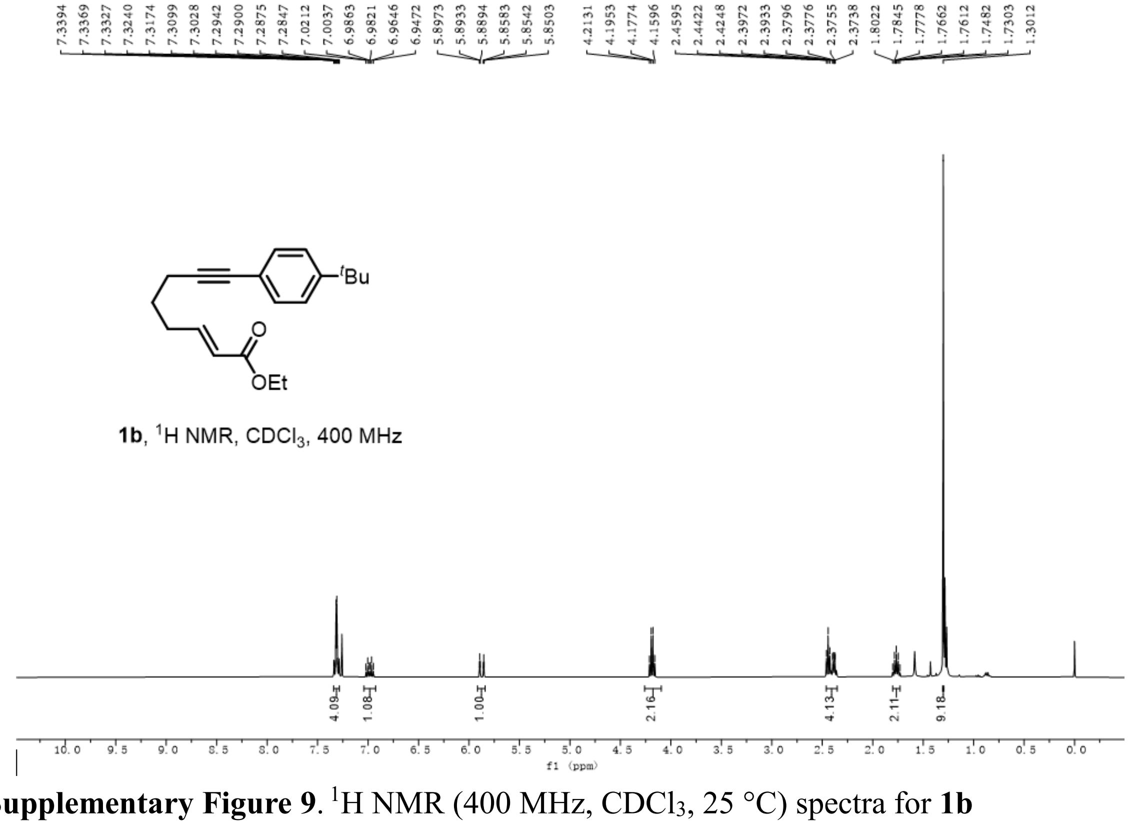

Supplementary Figure 9. ^{1}H NMR (400 MHz, $CDCl_3$, 25 °C) spectra for **1b**

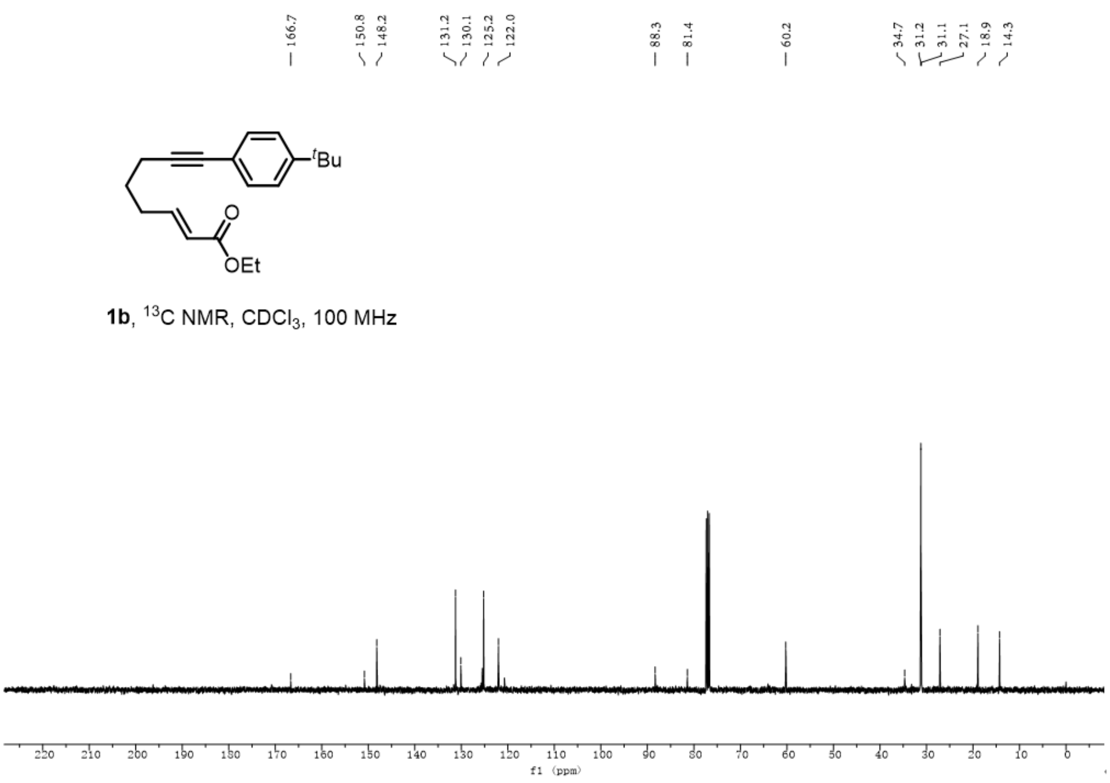

Supplementary Figure 10. ^{13}C NMR (400 MHz, $CDCl_3$, 25 °C) spectra for **1b**

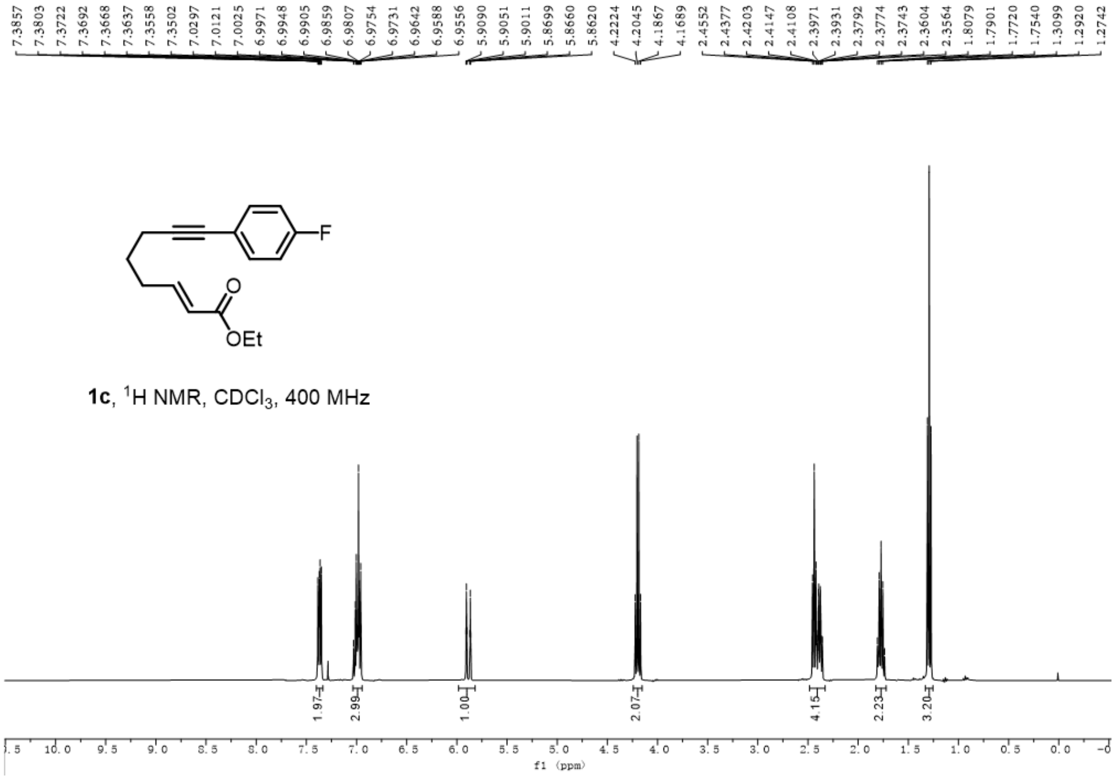

Supplementary Figure 11. ^{1}H NMR (400 MHz, $CDCl_3$, 25 °C) spectra for **1c**

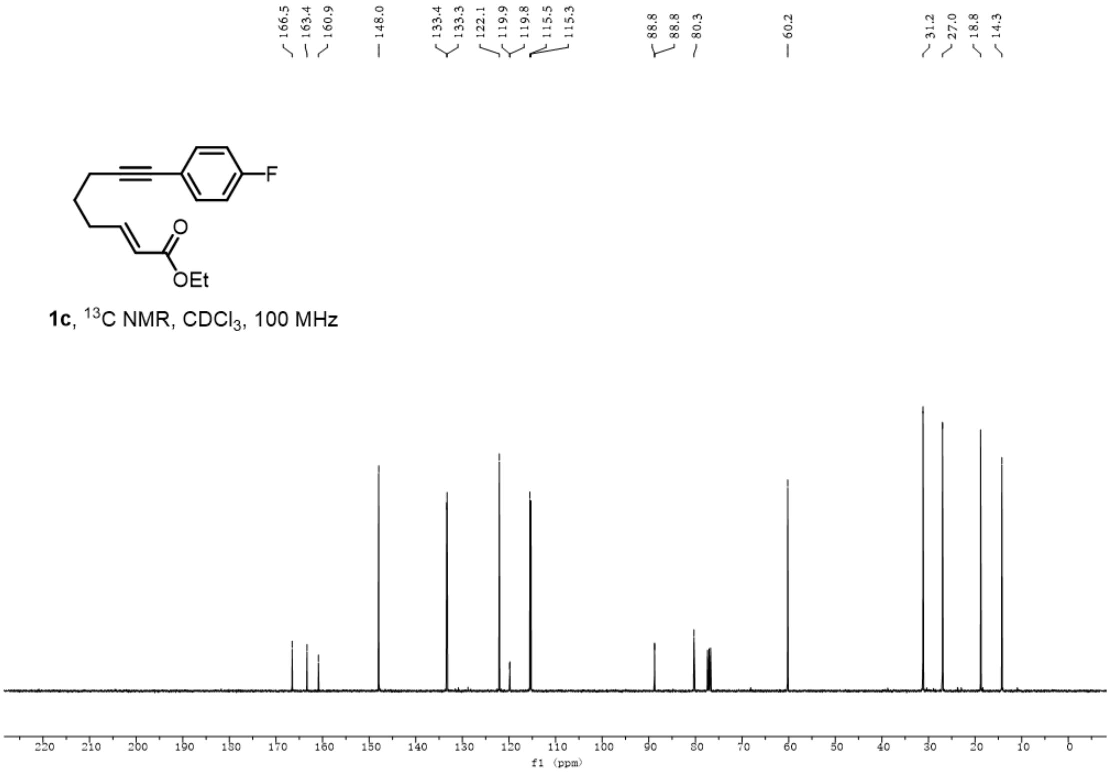

Supplementary Figure 12. ^{13}C NMR (400 MHz, $CDCl_3$, 25 °C) spectra for **1c**

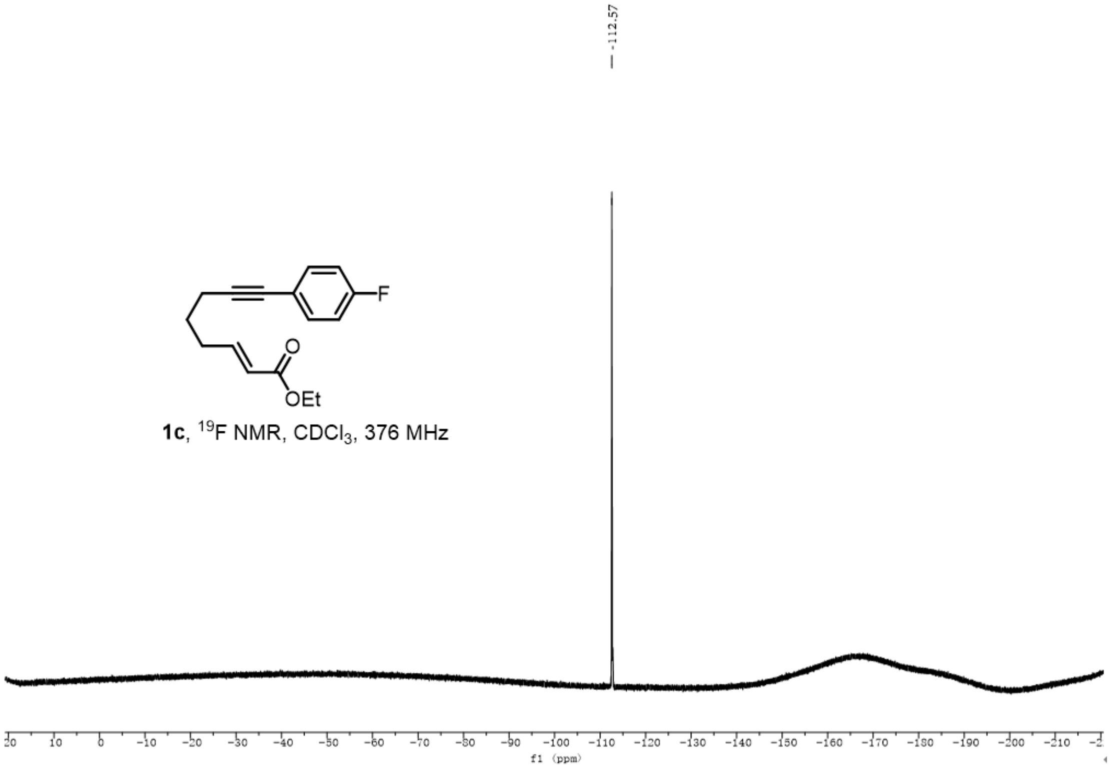

Supplementary Figure 13. ^{19}F NMR (400 MHz, $CDCl_3$, 25 °C) spectra for **1c**

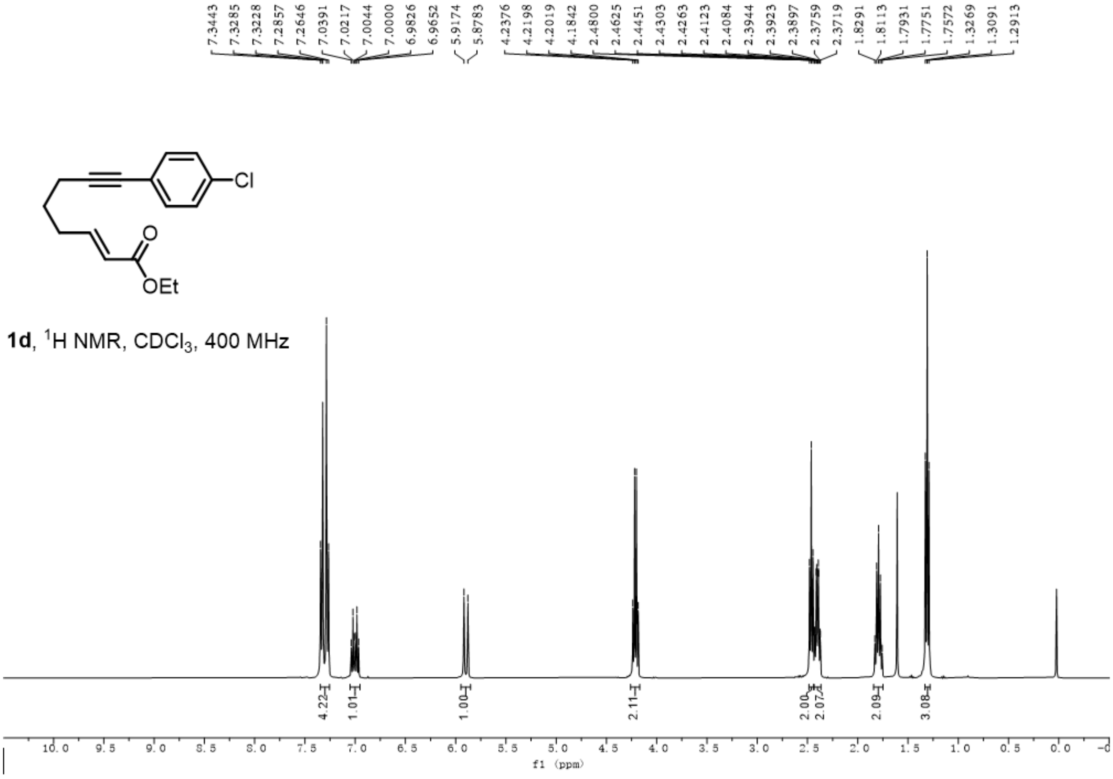

Supplementary Figure 14. ^{1}H NMR (400 MHz, CDCl$_3$, 25 °C) spectra for **1d**

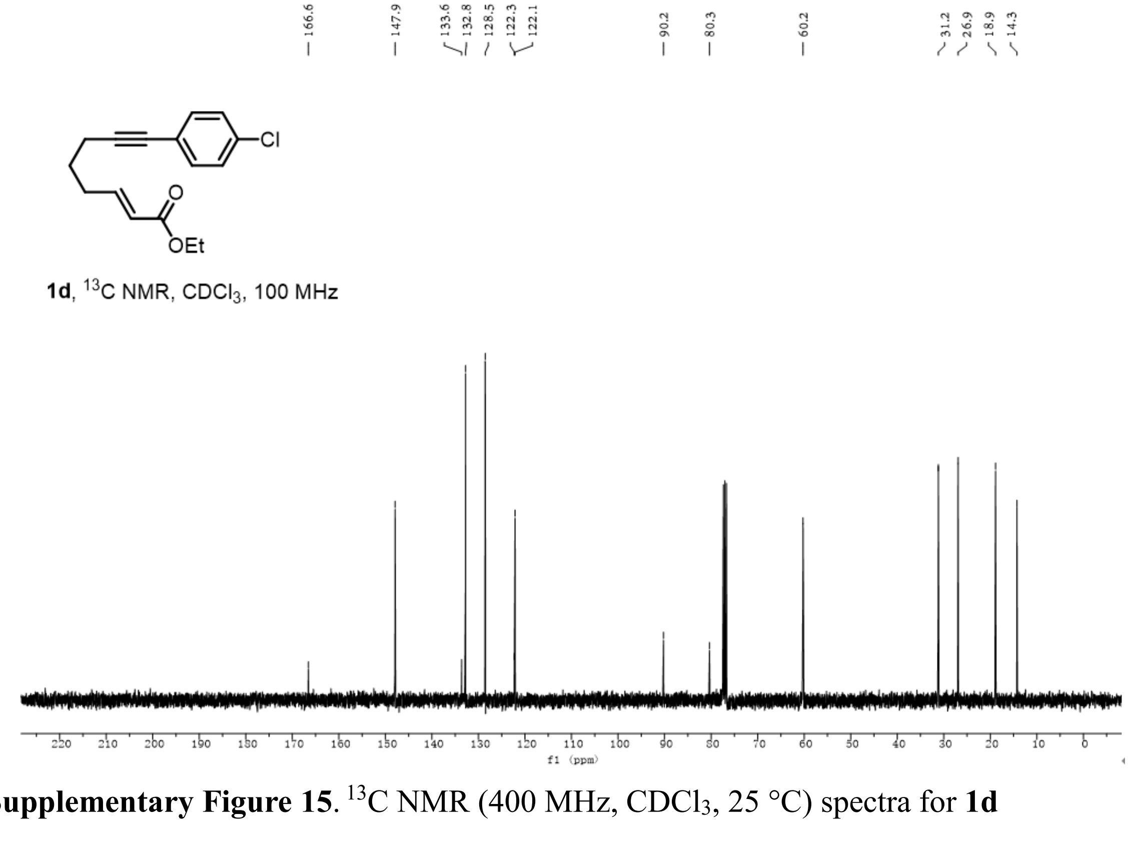

Supplementary Figure 15. ^{13}C NMR (400 MHz, CDCl$_3$, 25 °C) spectra for **1d**

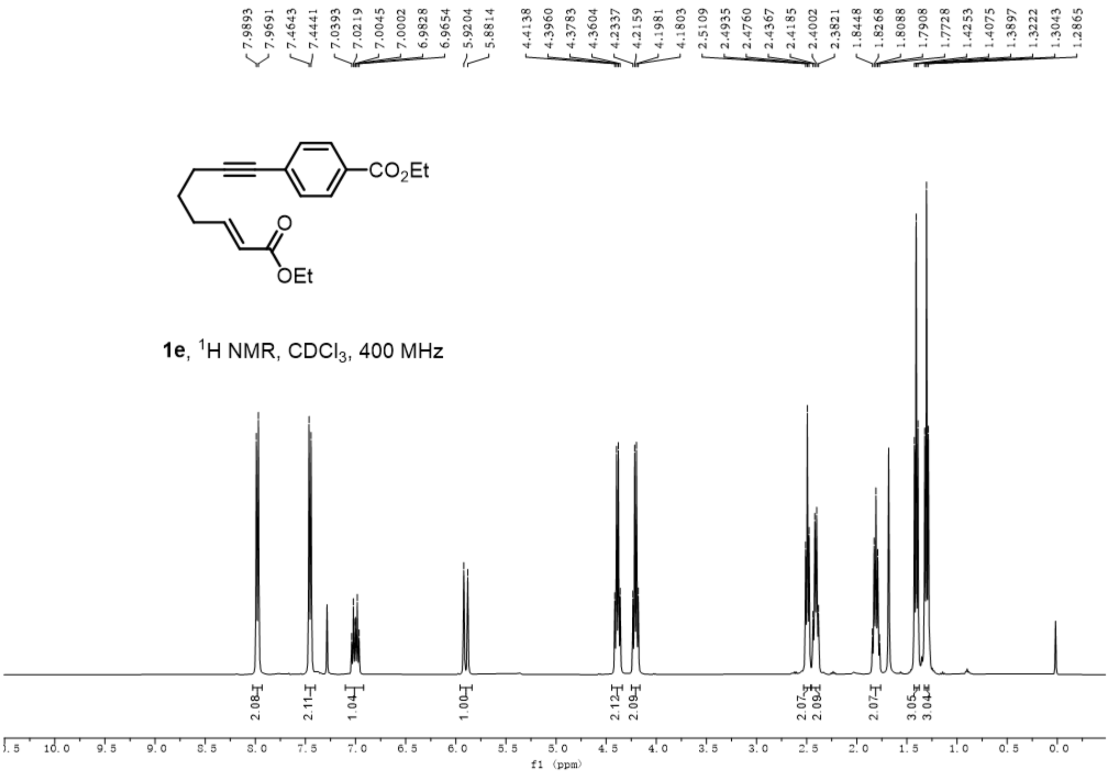

Supplementary Figure 16. ^{1}H NMR (400 MHz, $CDCl_3$, 25 °C) spectra for **1e**

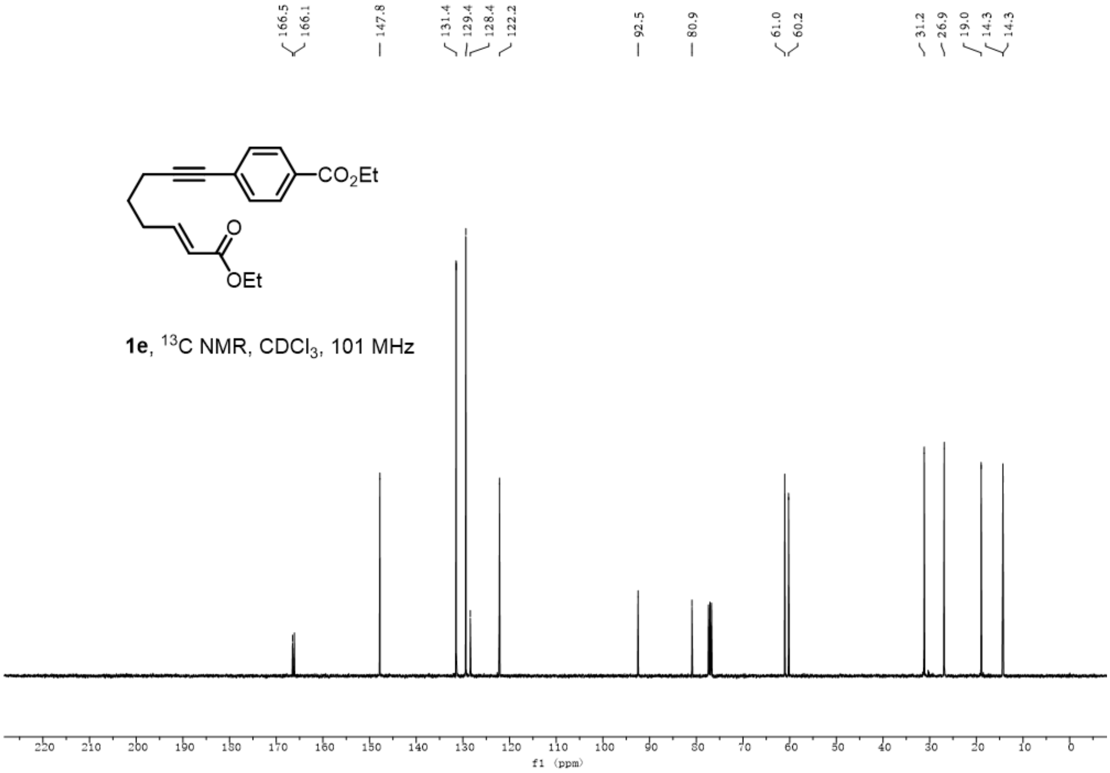

Supplementary Figure 17. ^{13}C NMR (400 MHz, $CDCl_3$, 25 °C) spectra for **1e**

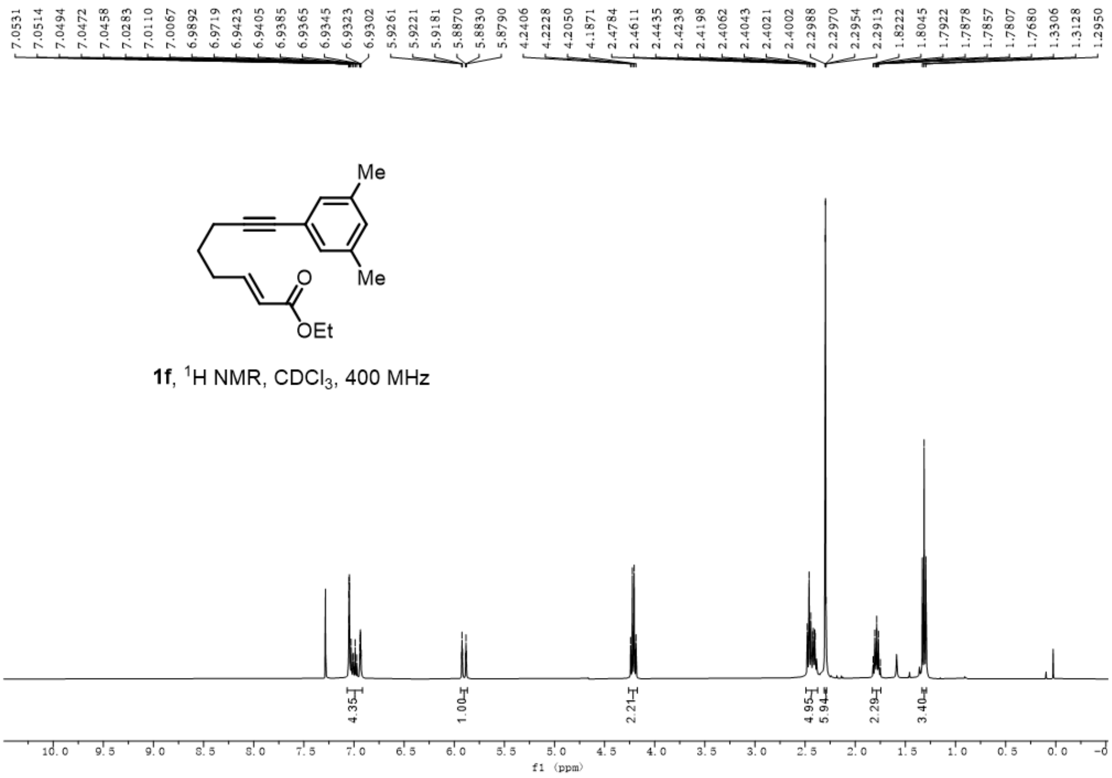

Supplementary Figure 18. ^{1}H NMR (400 MHz, $CDCl_3$, 25 °C) spectra for **1f**

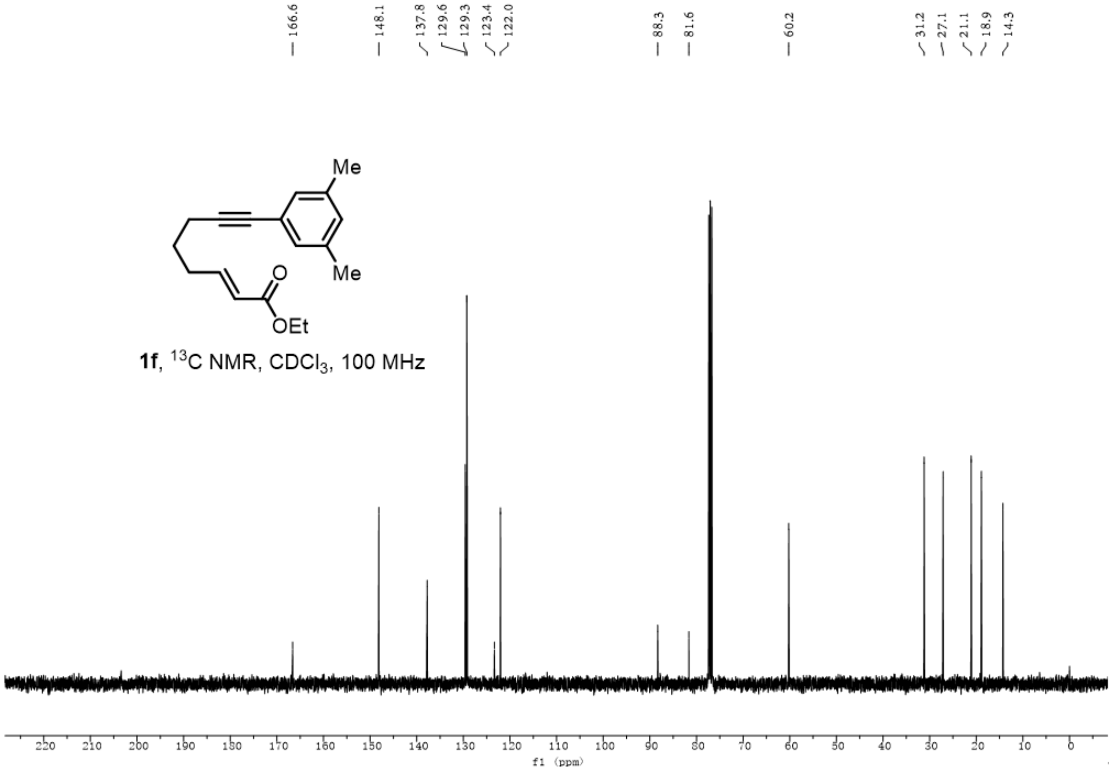

Supplementary Figure 19. ^{13}C NMR (400 MHz, $CDCl_3$, 25 °C) spectra for **1f**

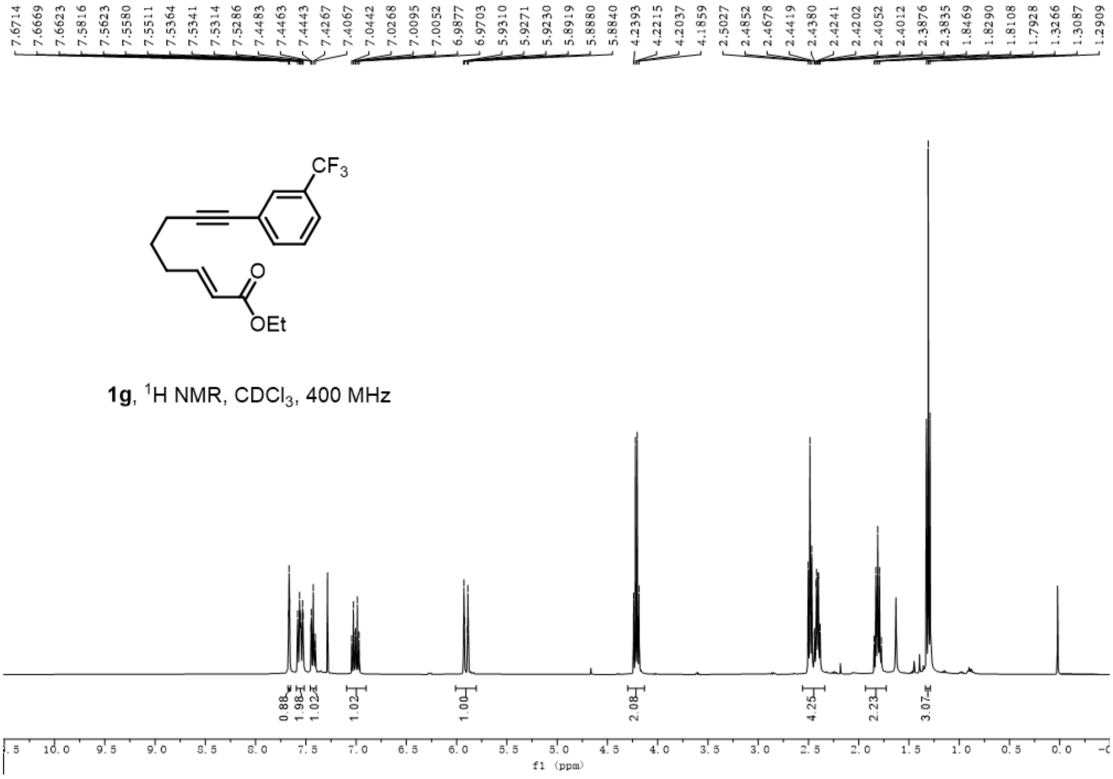

Supplementary Figure 20. ^{1}H NMR (400 MHz, $CDCl_3$, 25 °C) spectra for **1g**

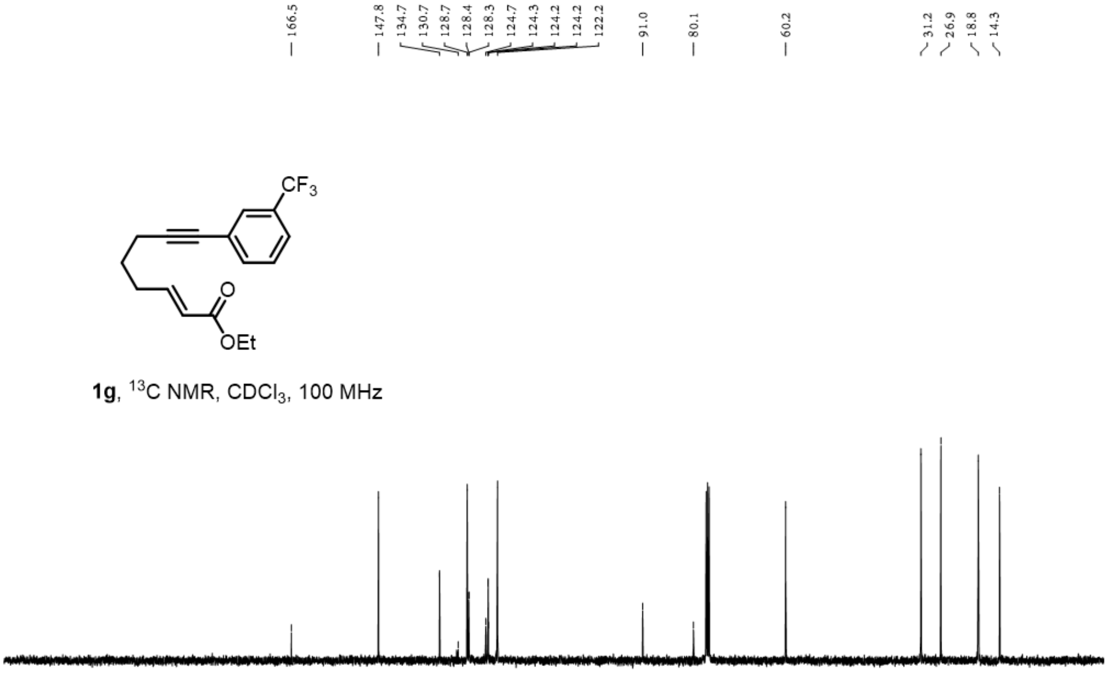

Supplementary Figure 21. ^{13}C NMR (400 MHz, $CDCl_3$, 25 °C) spectra for **1g**

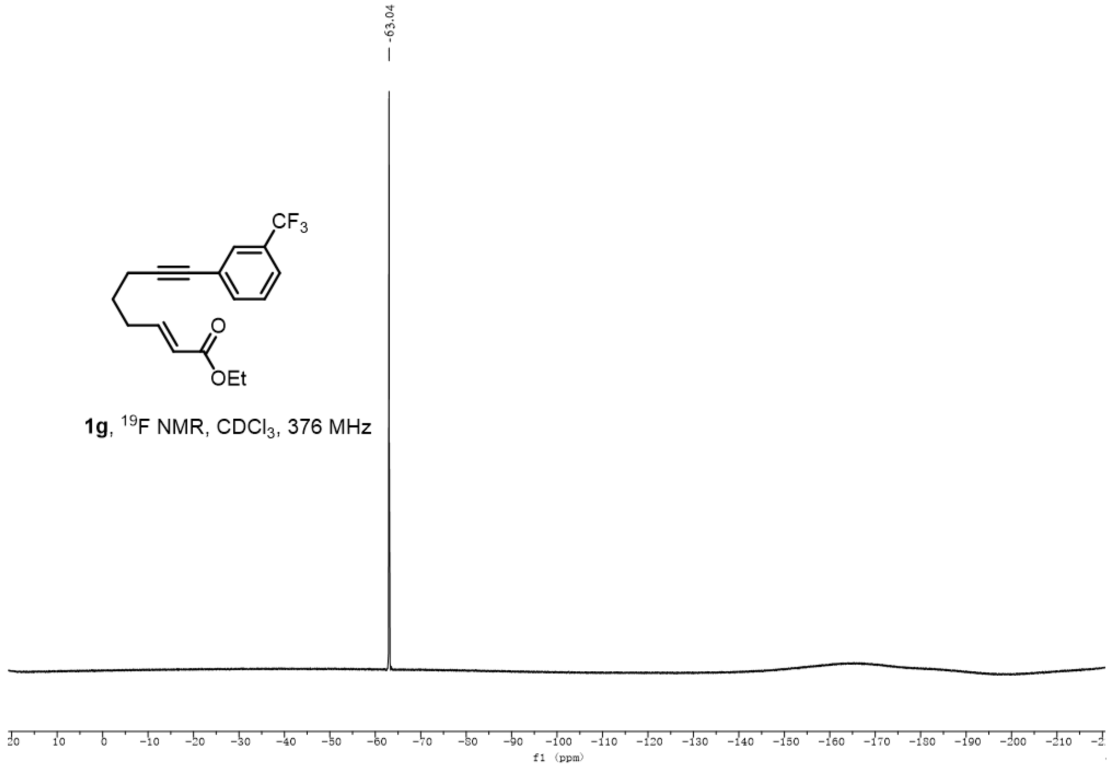

Supplementary Figure 22. ^{19}F NMR (400 MHz, $CDCl_3$, 25 °C) spectra for **1g**

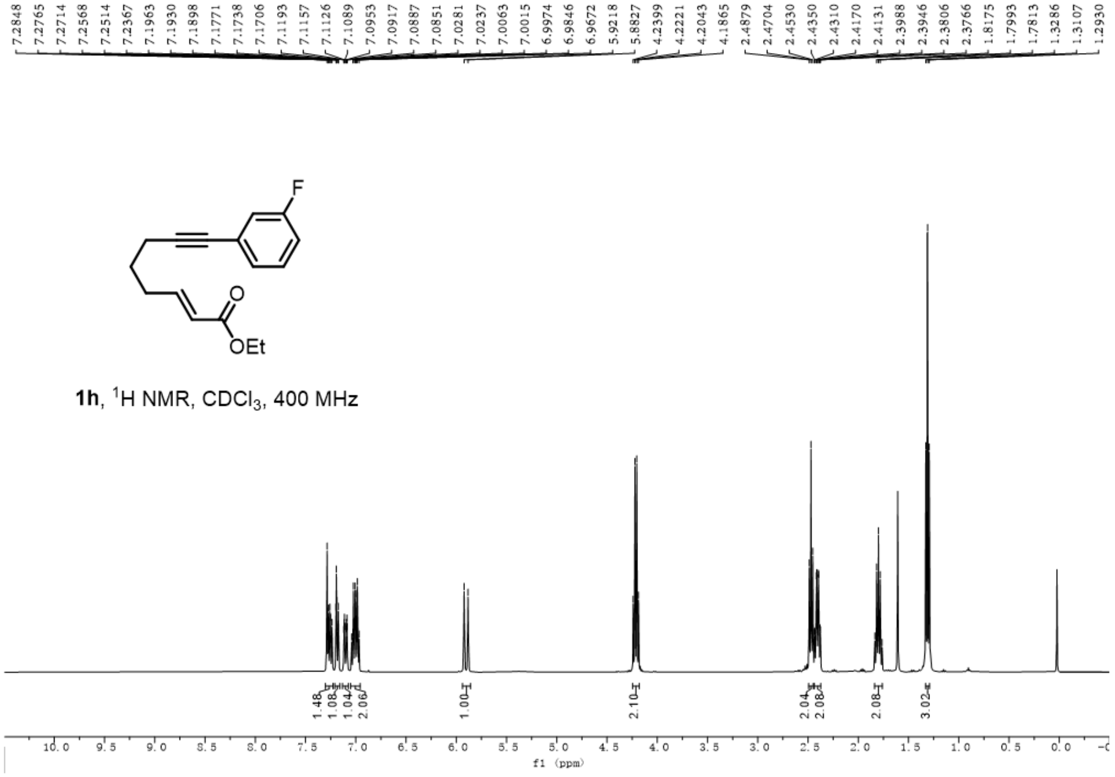

Supplementary Figure 23. ^{1}H NMR (400 MHz, $CDCl_3$, 25 °C) spectra for **1h**

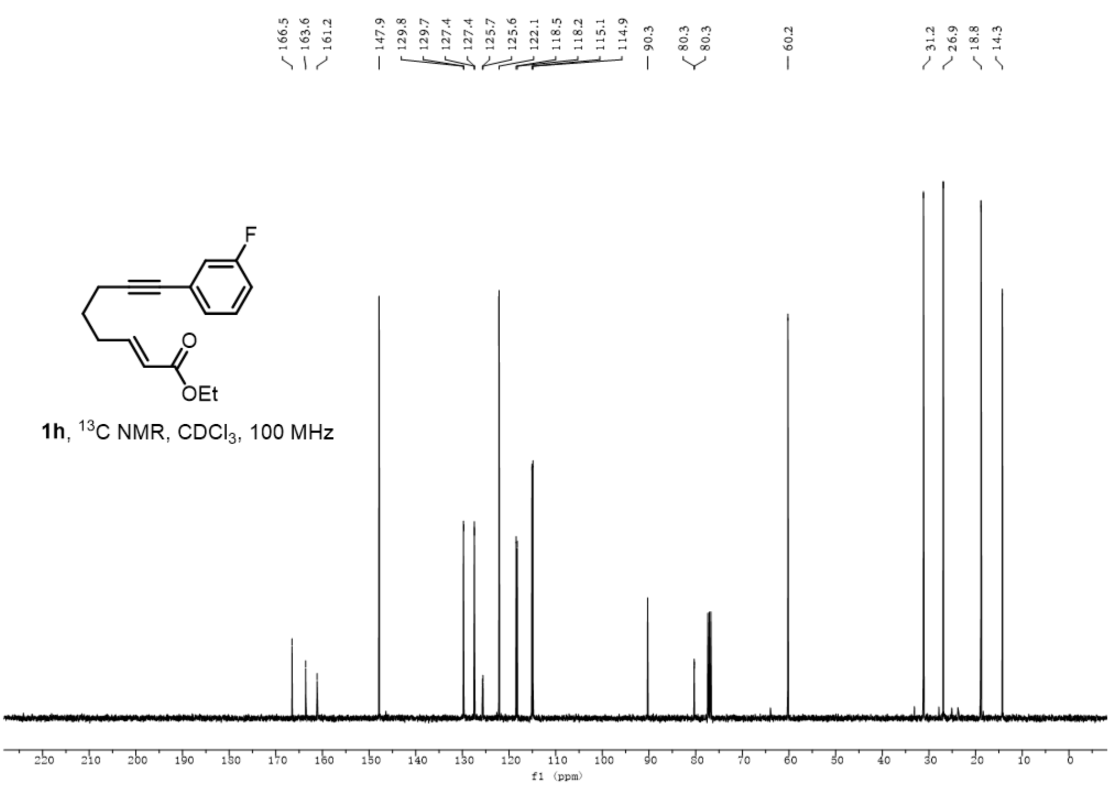

Supplementary Figure 24. ^{13}C NMR (400 MHz, $CDCl_3$, 25 °C) spectra for **1h**

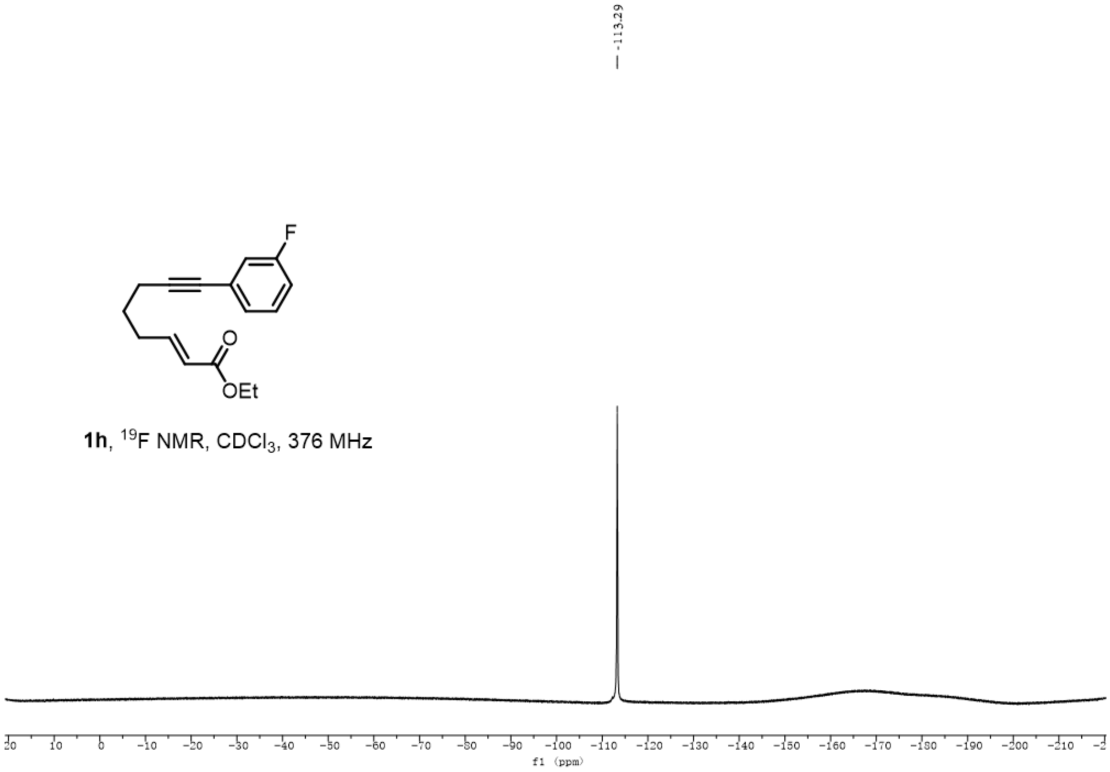

Supplementary Figure 25. ^{19}F NMR (400 MHz, $CDCl_3$, 25 °C) spectra for **1h**

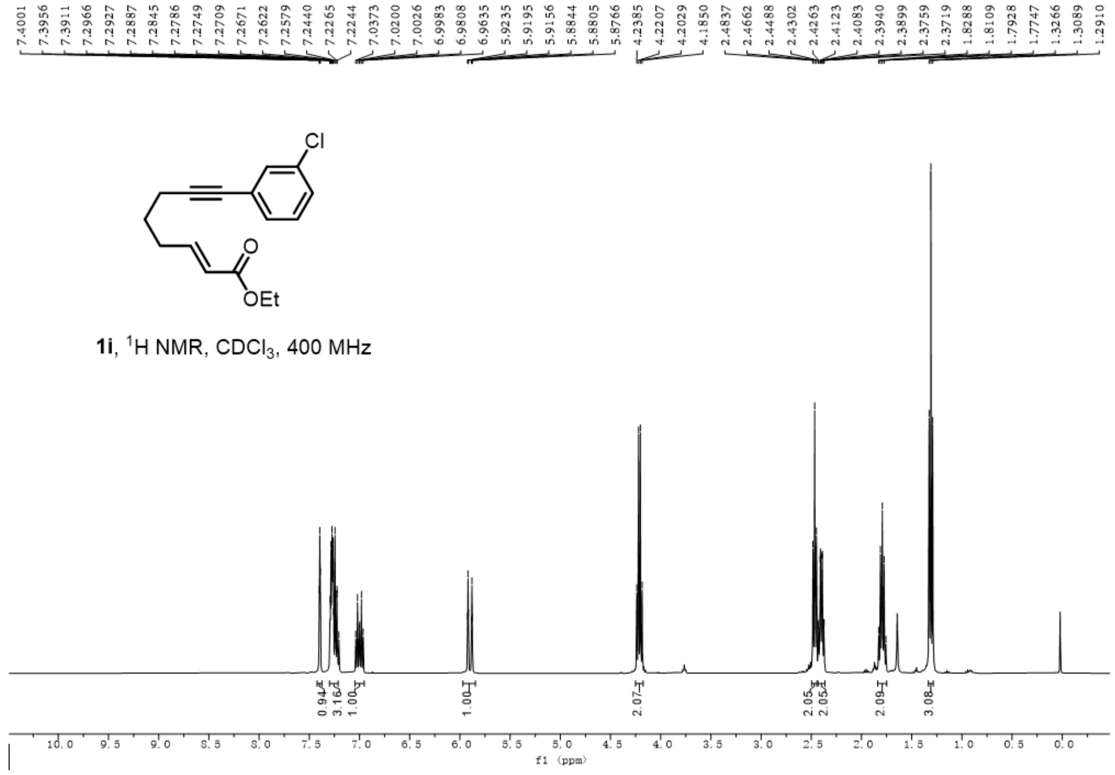

Supplementary Figure 26. ^{1}H NMR (400 MHz, $CDCl_3$, 25 °C) spectra for **1i**

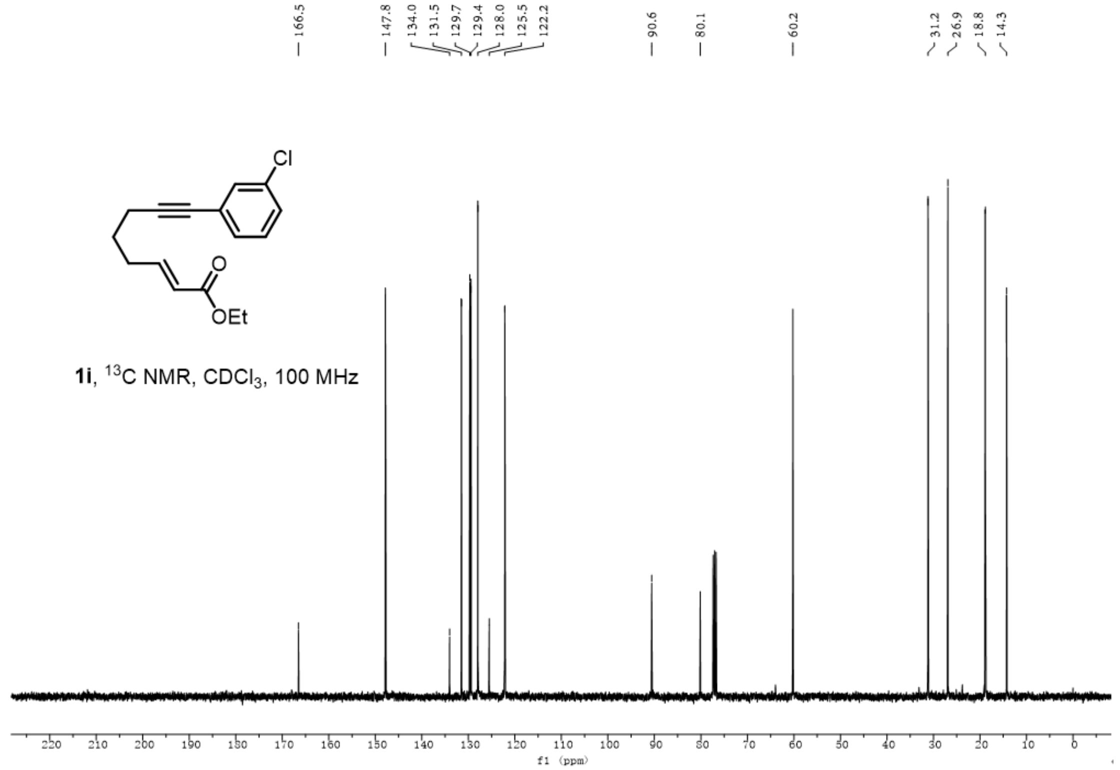

Supplementary Figure 27. ^{13}C NMR (400 MHz, $CDCl_3$, 25 °C) spectra for **1i**

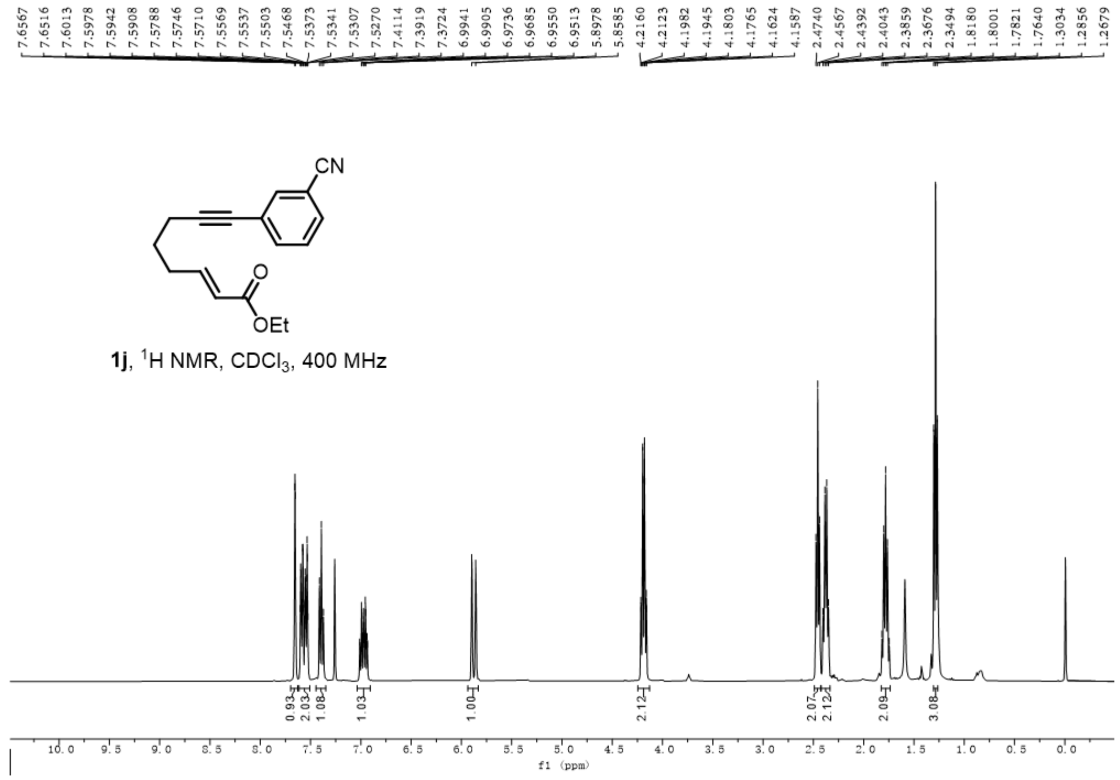

Supplementary Figure 28. ^{1}H NMR (400 MHz, $CDCl_3$, 25 °C) spectra for **1i**

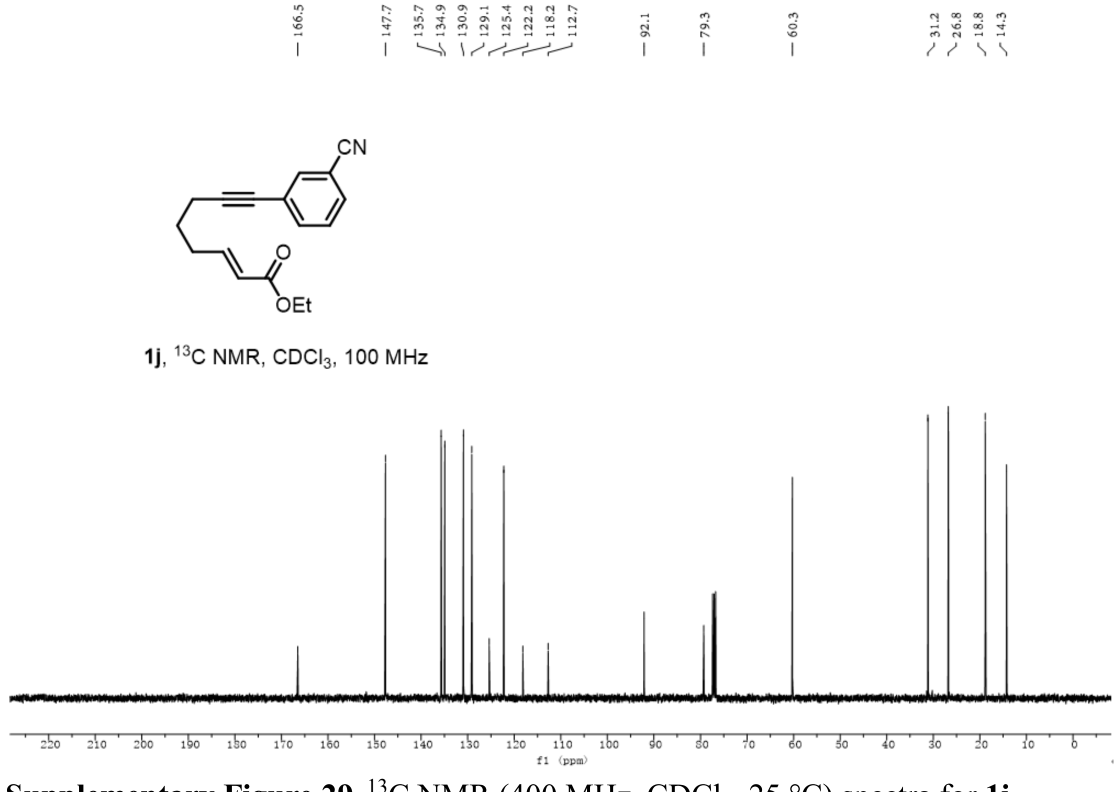

Supplementary Figure 29. ^{13}C NMR (400 MHz, $CDCl_3$, 25 °C) spectra for **1i**

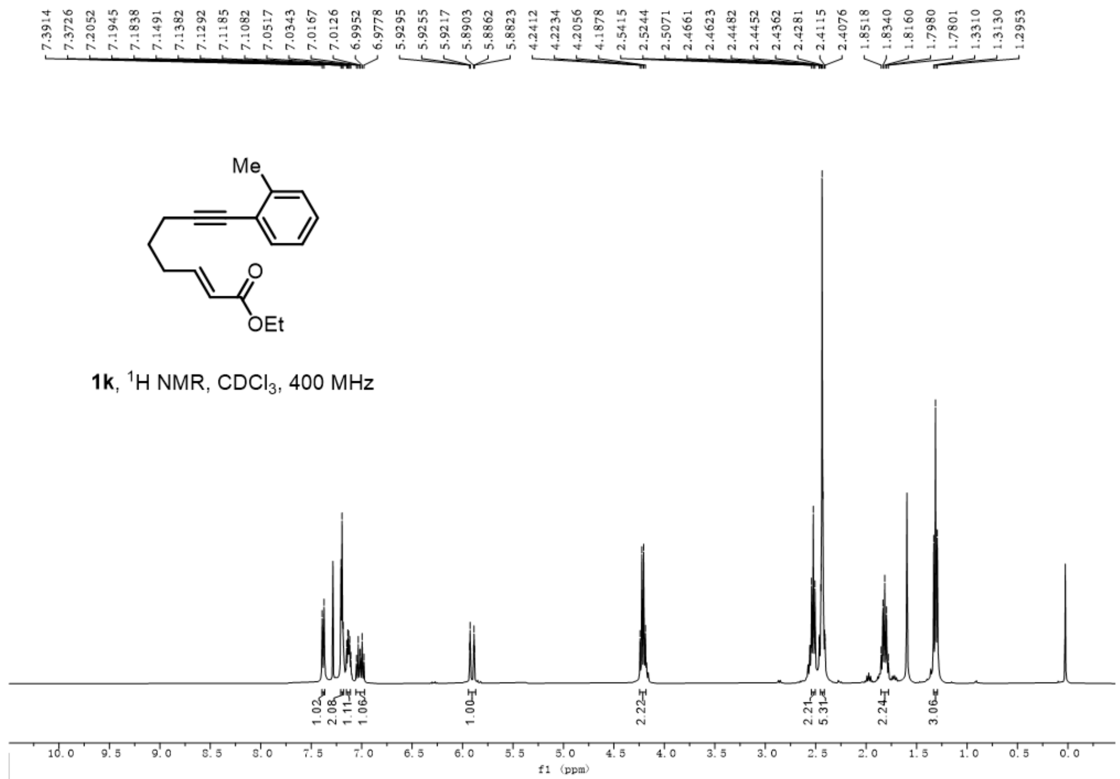

Supplementary Figure 30. ^{1}H NMR (400 MHz, $CDCl_3$, 25 °C) spectra for **1k**

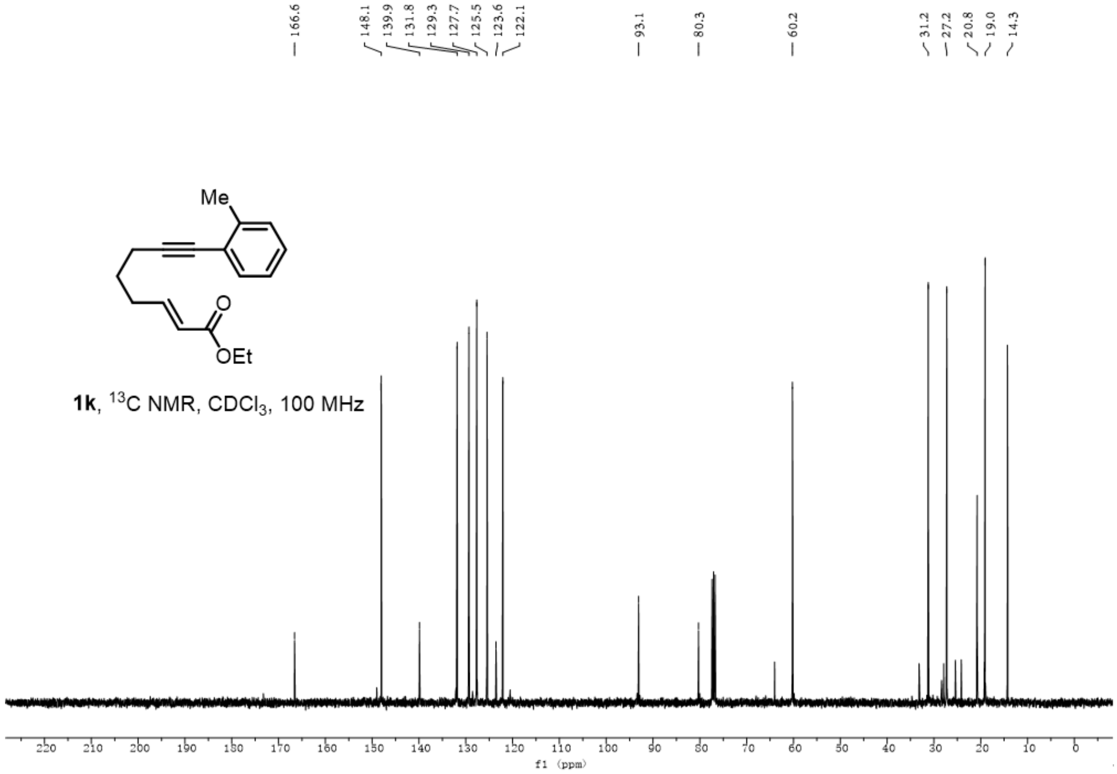

Supplementary Figure 31. ^{13}C NMR (400 MHz, $CDCl_3$, 25 °C) spectra for **1k**

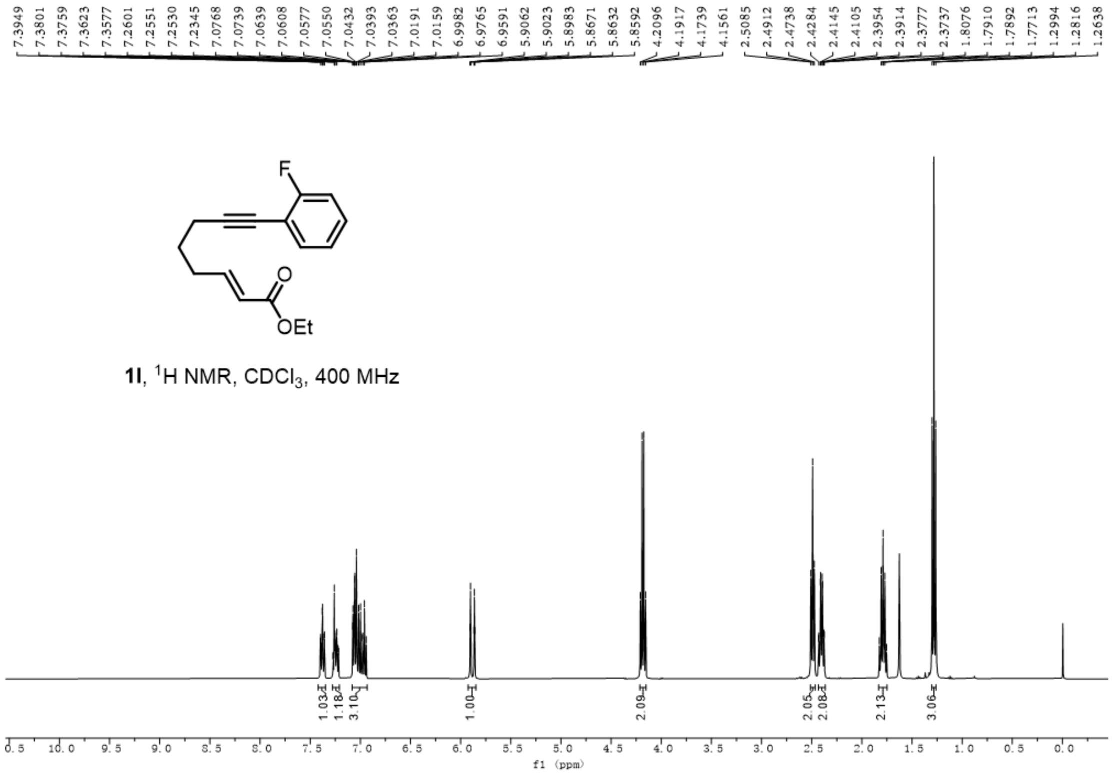

Supplementary Figure 32. ^{1}H NMR (400 MHz, $CDCl_3$, 25 °C) spectra for **1l**

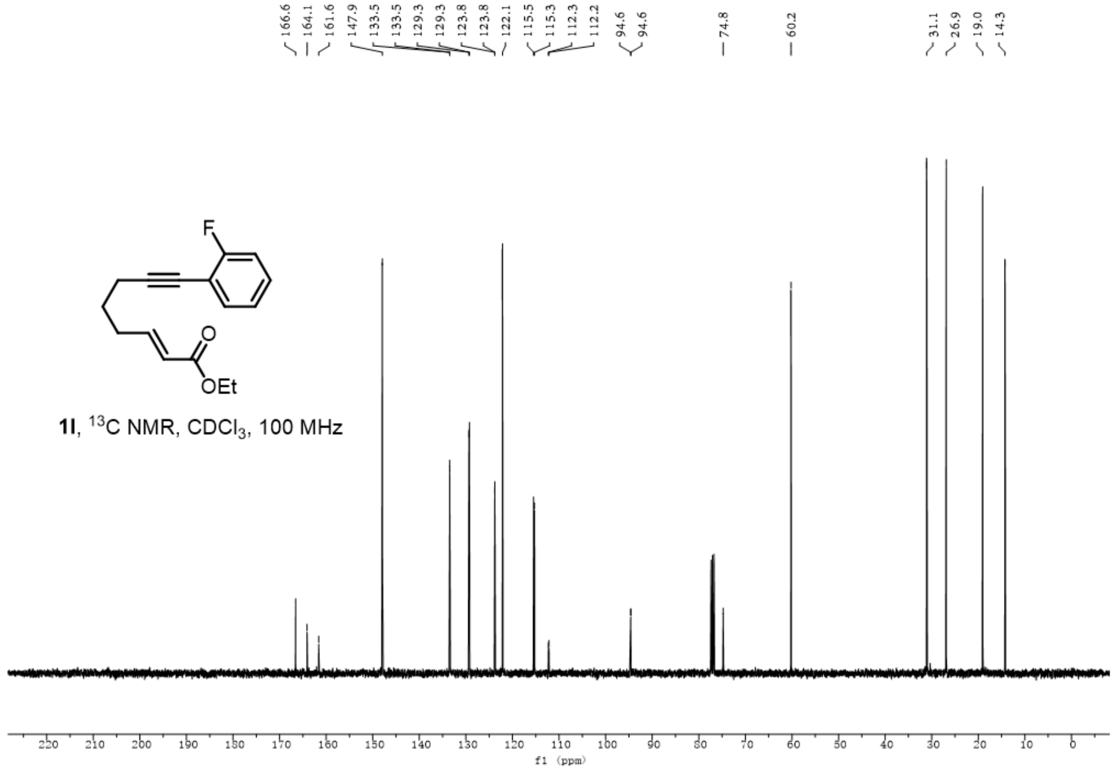

Supplementary Figure 33. ^{13}C NMR (400 MHz, $CDCl_3$, 25 °C) spectra for **1l**

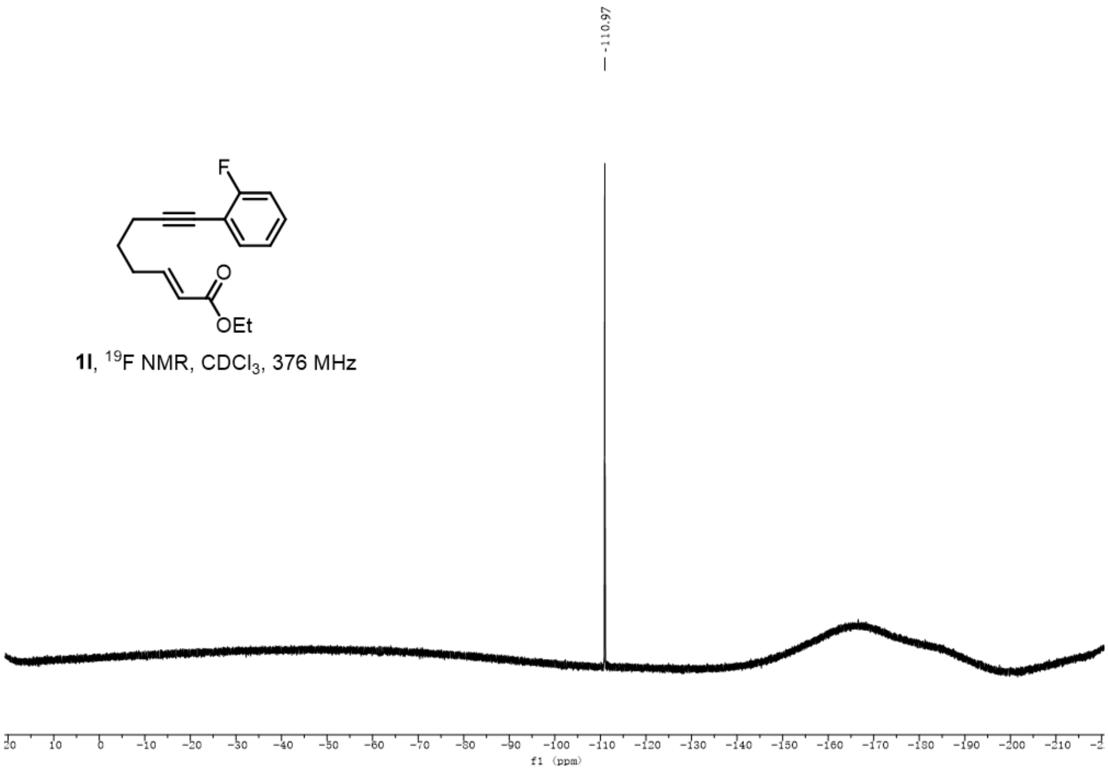

Supplementary Figure 34. ^{19}F NMR (400 MHz, $CDCl_3$, 25 °C) spectra for **1l**

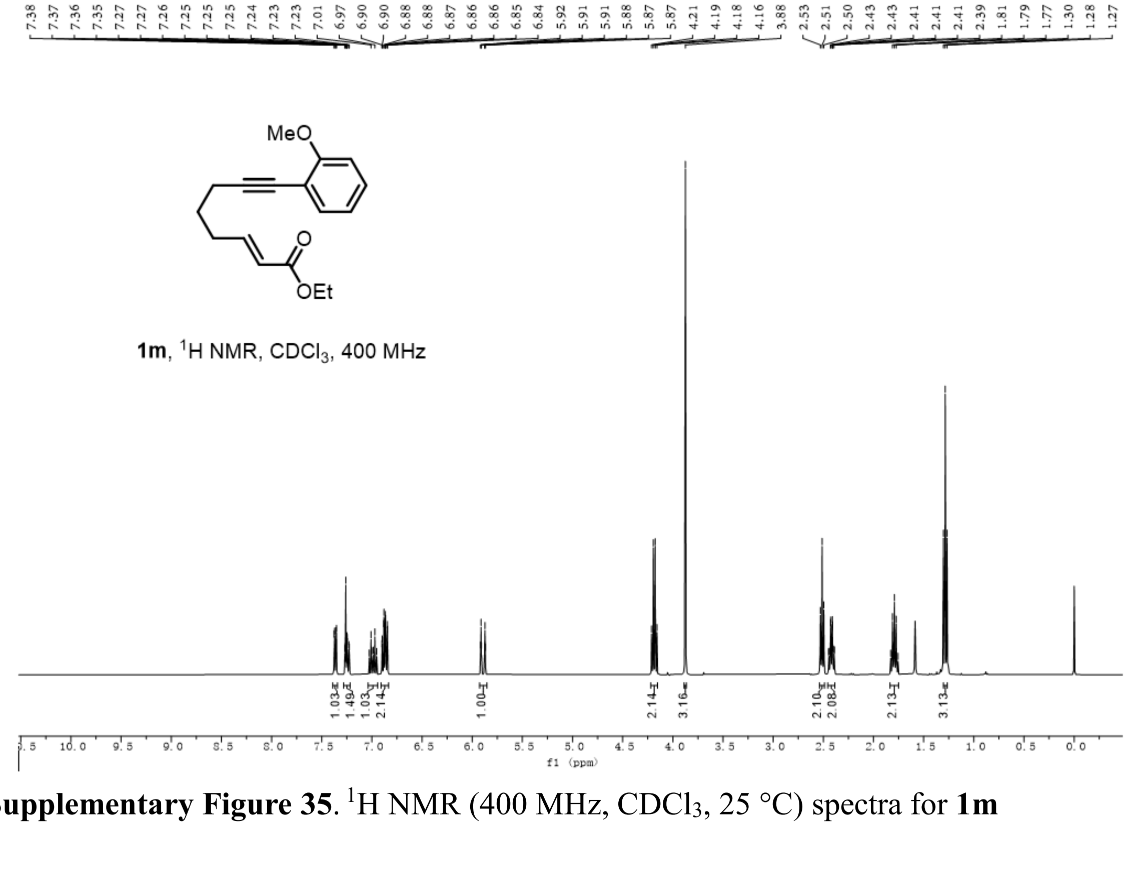

Supplementary Figure 35. ^{1}H NMR (400 MHz, $CDCl_3$, 25 °C) spectra for **1m**

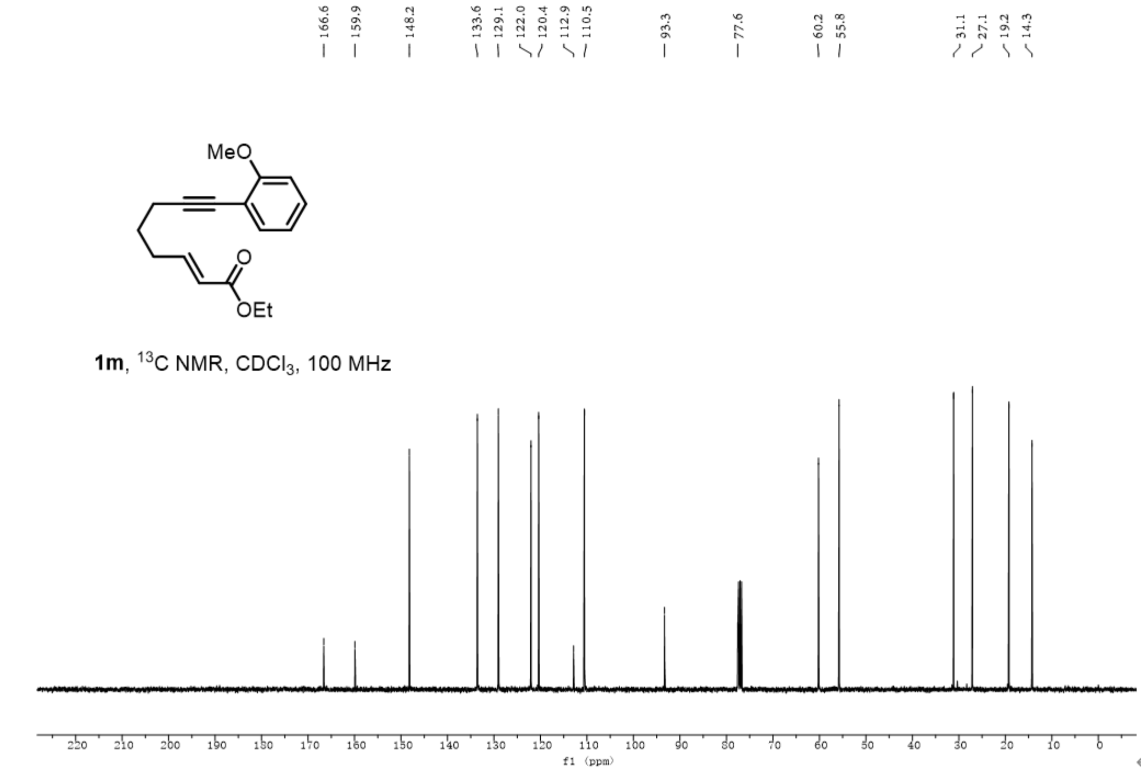

Supplementary Figure 36. ^{13}C NMR (400 MHz, CDCl$_3$, 25 °C) spectra for **1m**

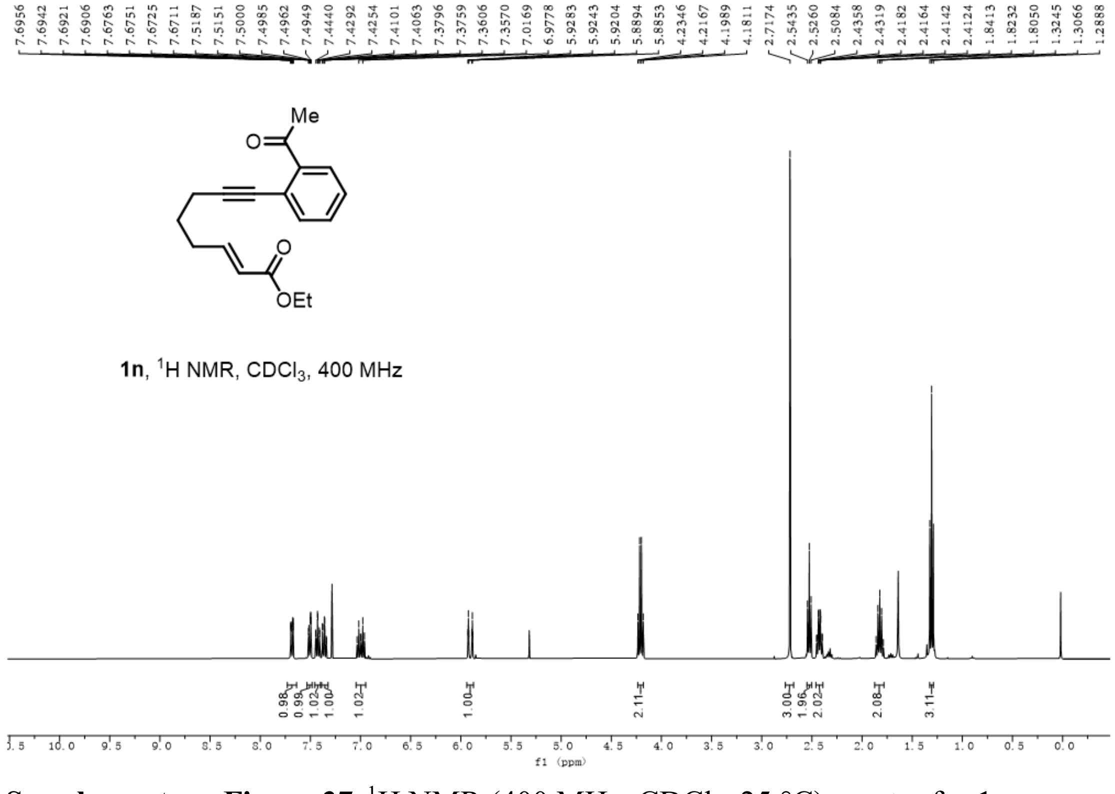

Supplementary Figure 37. ^{1}H NMR (400 MHz, CDCl$_3$, 25 °C) spectra for **1n**

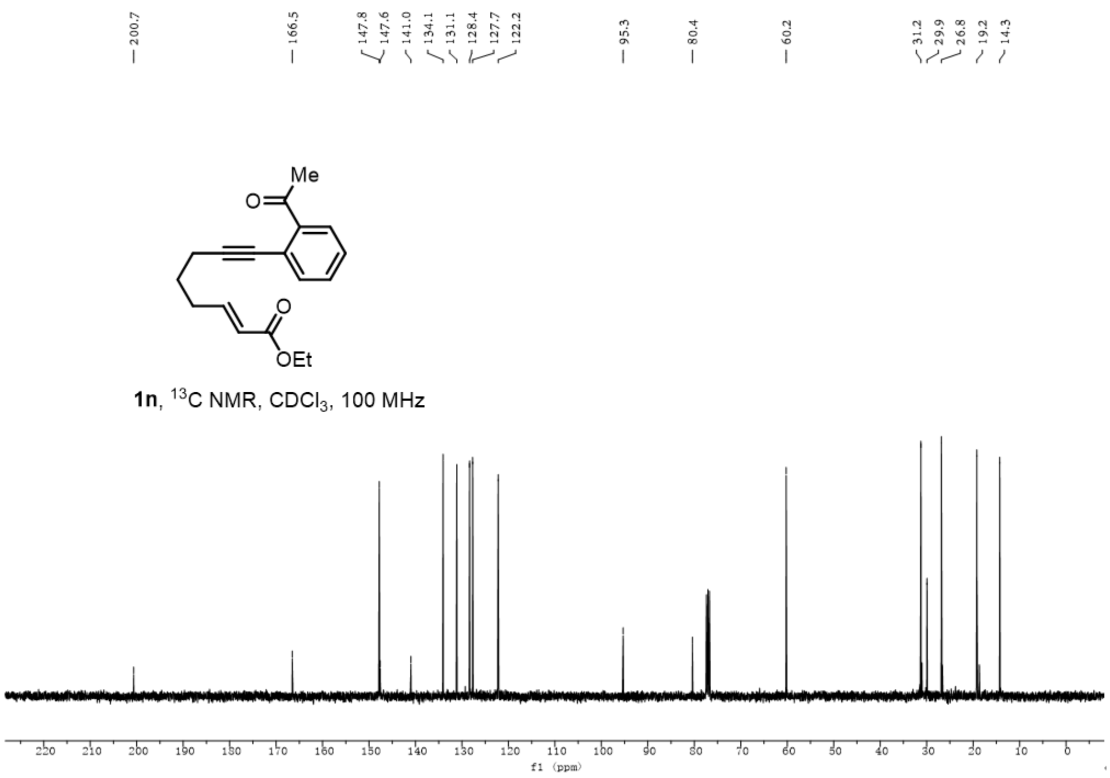

Supplementary Figure 38. ^{13}C NMR (400 MHz, $CDCl_3$, 25 °C) spectra for **1n**

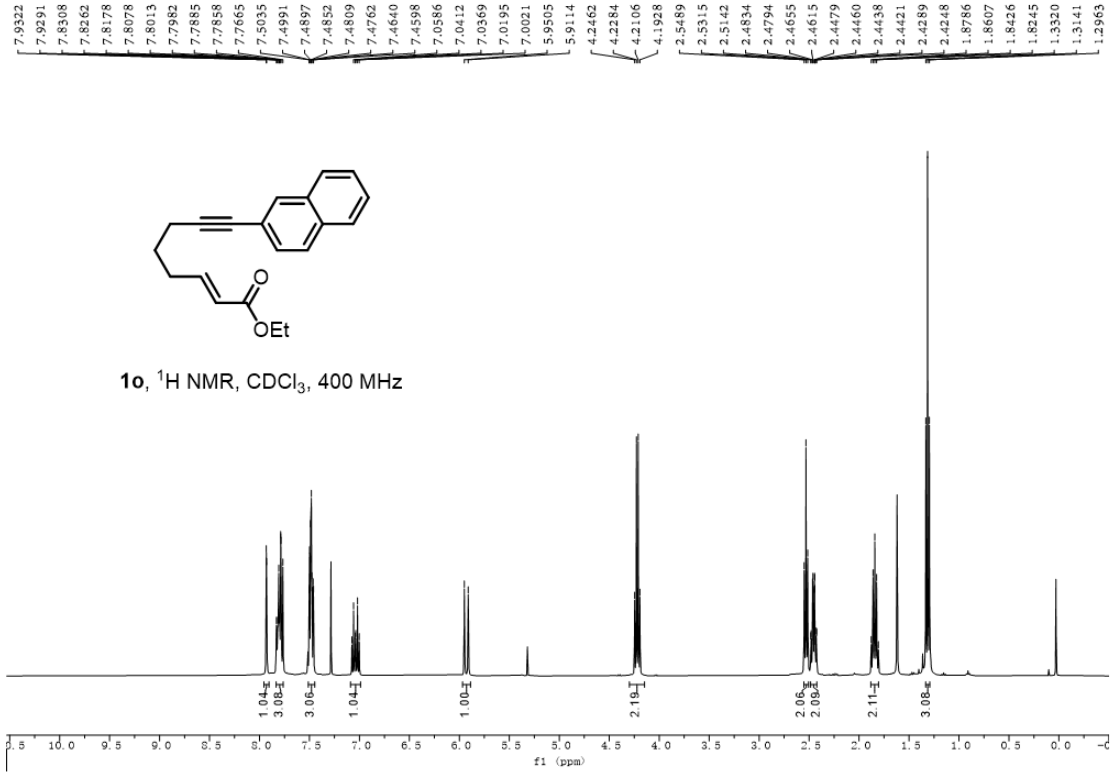

Supplementary Figure 39. ^{1}H NMR (400 MHz, $CDCl_3$, 25 °C) spectra for **1o**

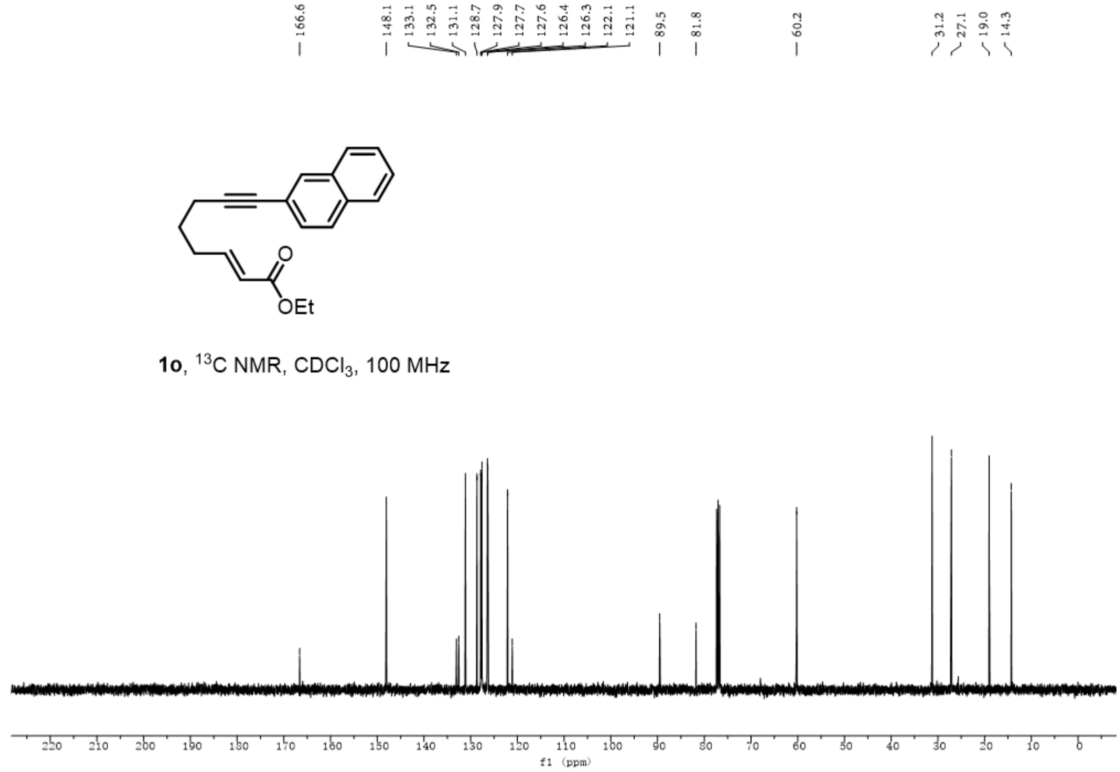

Supplementary Figure 40. ^{13}C NMR (400 MHz, $CDCl_3$, 25 °C) spectra for **1o**

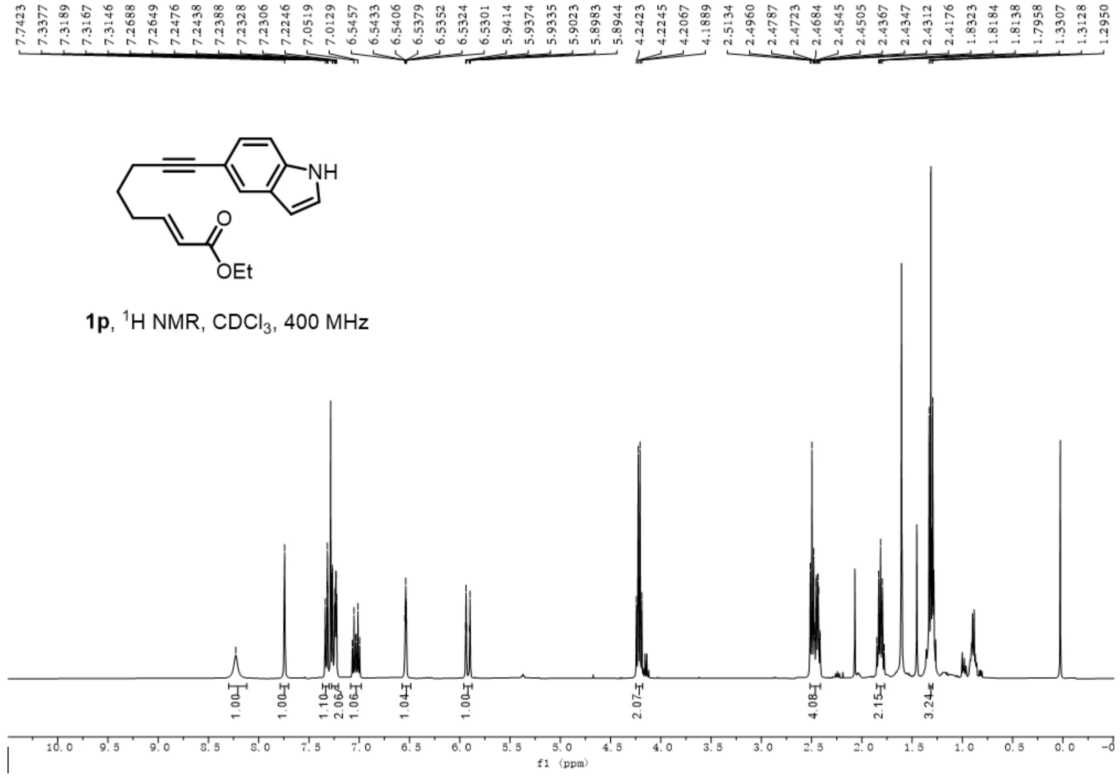

Supplementary Figure 41. ^{1}H NMR (400 MHz, $CDCl_3$, 25 °C) spectra for **1p**

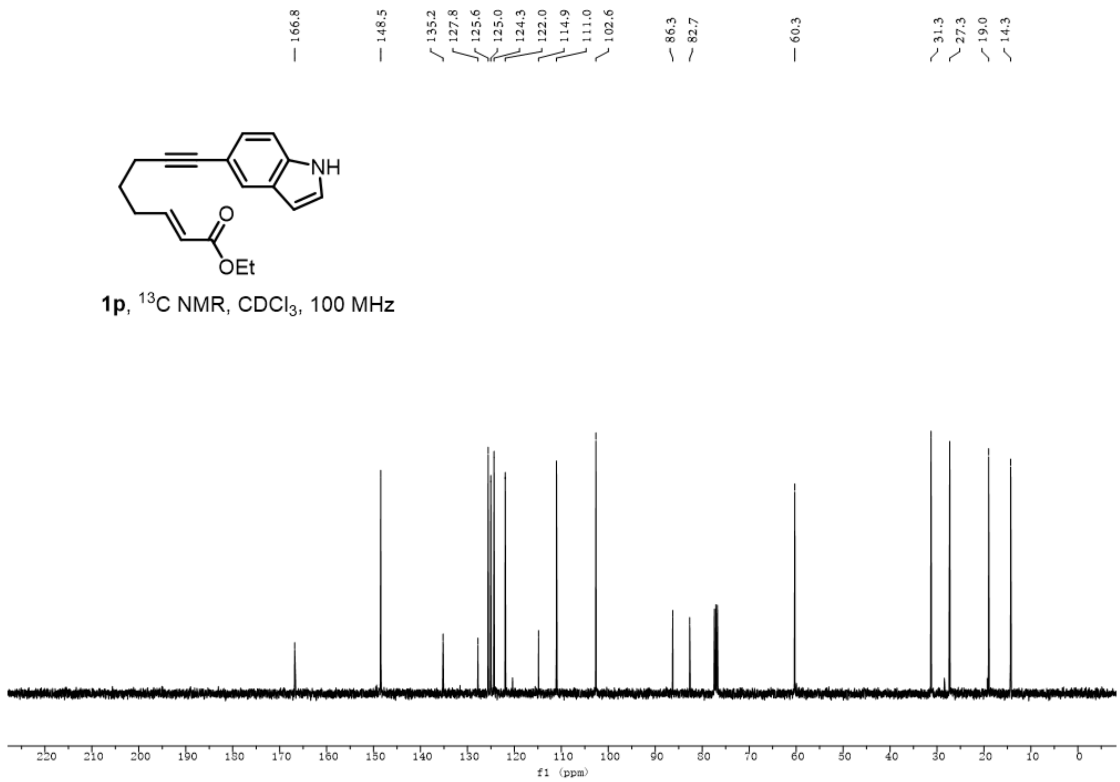

Supplementary Figure 42. ^{13}C NMR (400 MHz, $CDCl_3$, 25 °C) spectra for **1p**

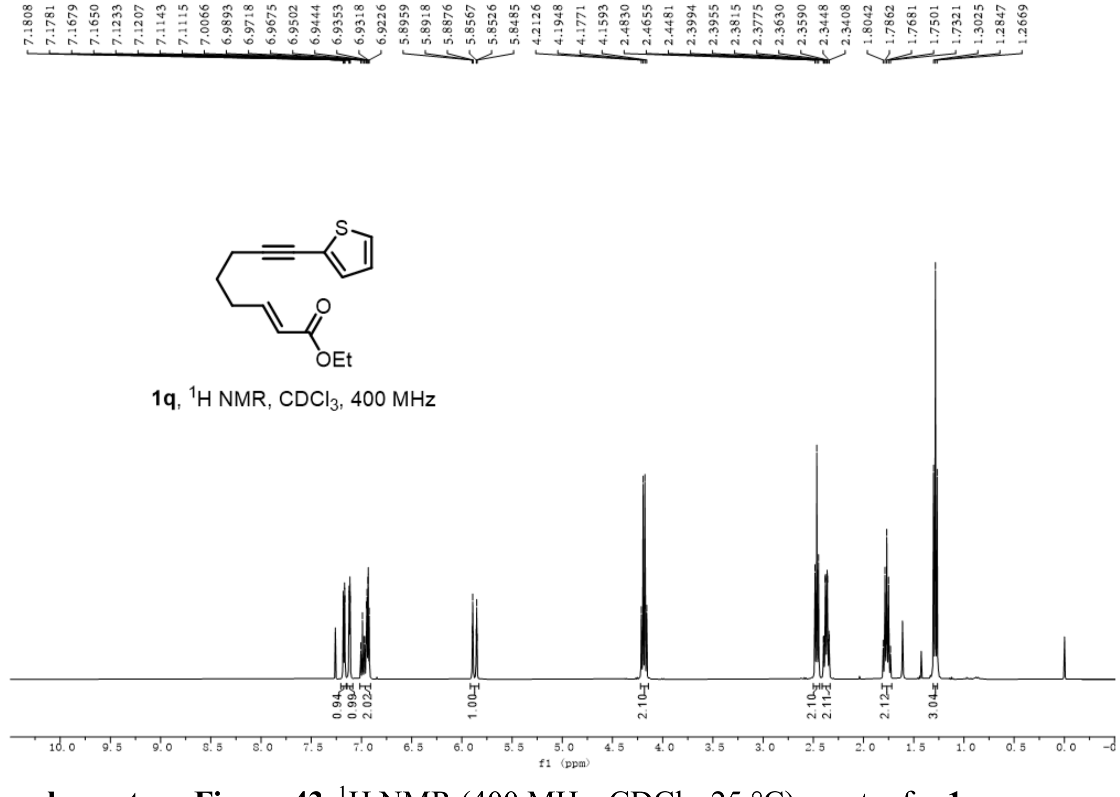

Supplementary Figure 43. ^{1}H NMR (400 MHz, $CDCl_3$, 25 °C) spectra for **1q**

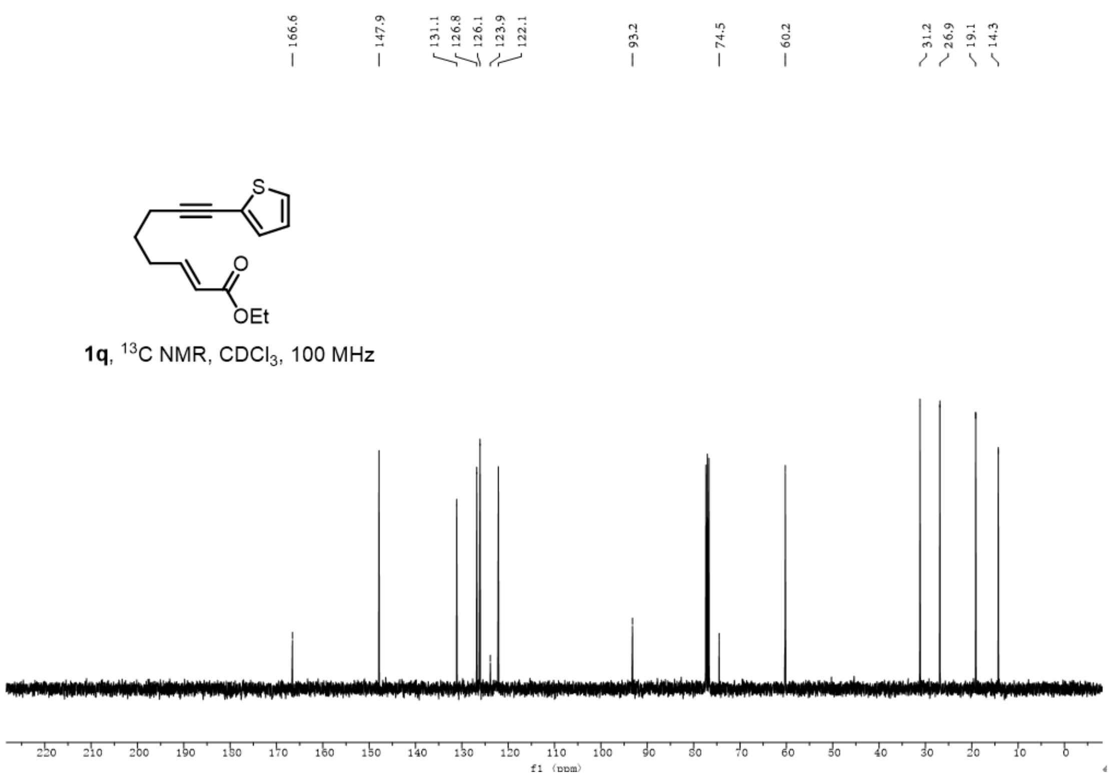

Supplementary Figure 44. ^{13}C NMR (400 MHz, CDCl$_3$, 25 °C) spectra for **1q**

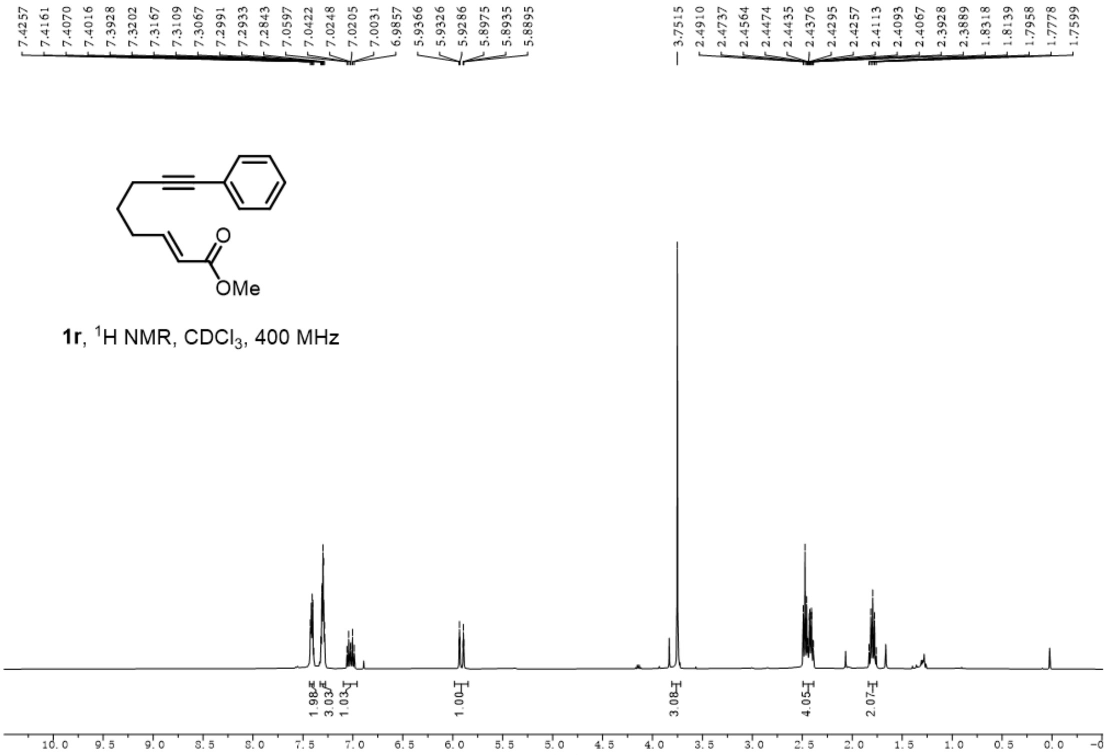

Supplementary Figure 45. ^{1}H NMR (400 MHz, CDCl$_3$, 25 °C) spectra for **1r**

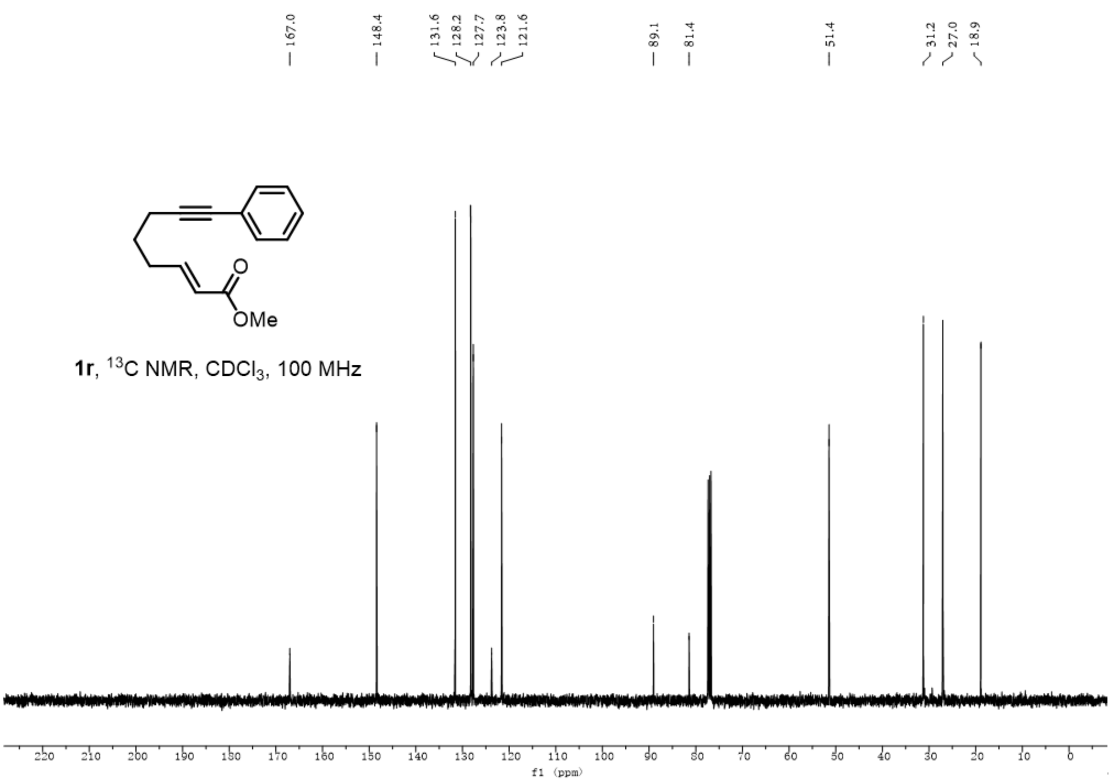

Supplementary Figure 46. ^{13}C NMR (400 MHz, $CDCl_3$, 25 °C) spectra for **1r**

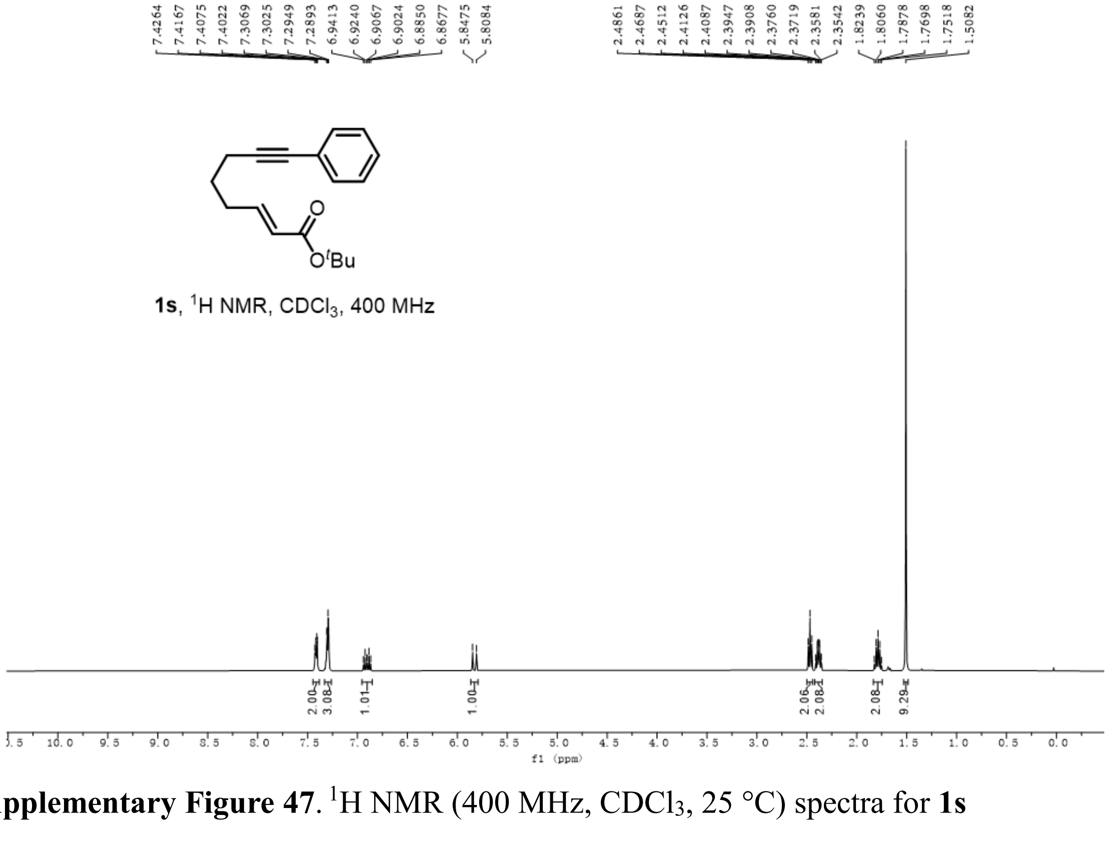

Supplementary Figure 47. ^{1}H NMR (400 MHz, $CDCl_3$, 25 °C) spectra for **1s**

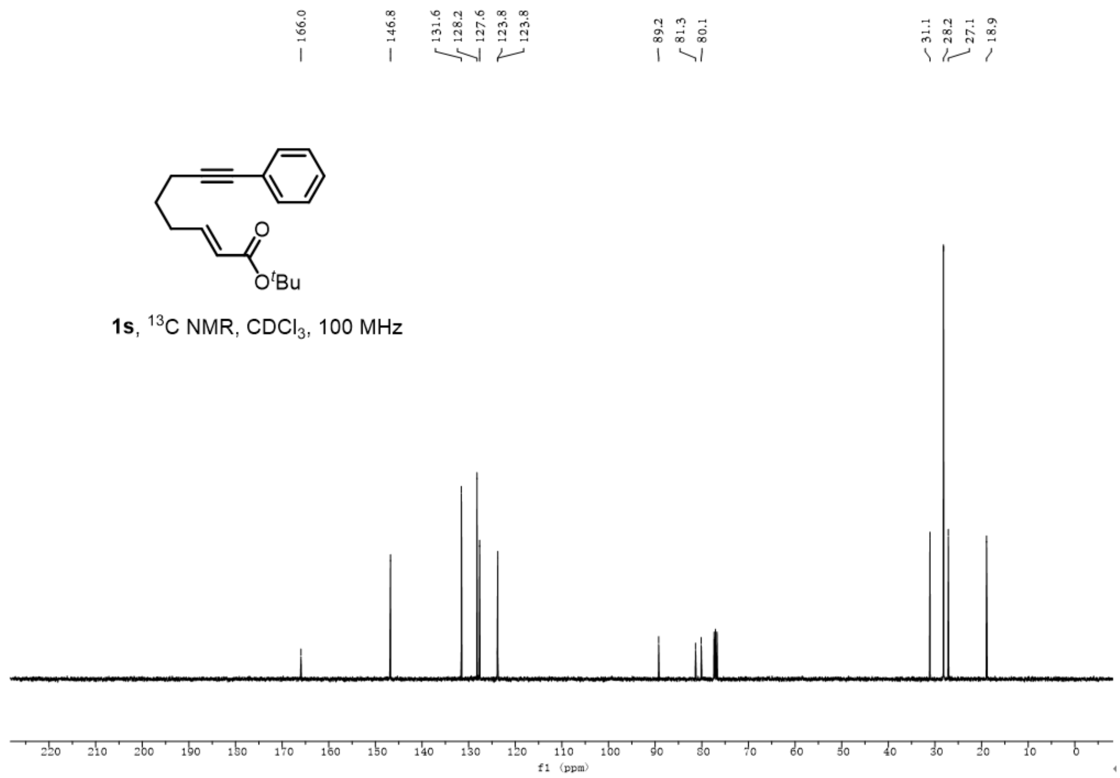

Supplementary Figure 48. ^{13}C NMR (400 MHz, $CDCl_3$, 25 °C) spectra for **1s**

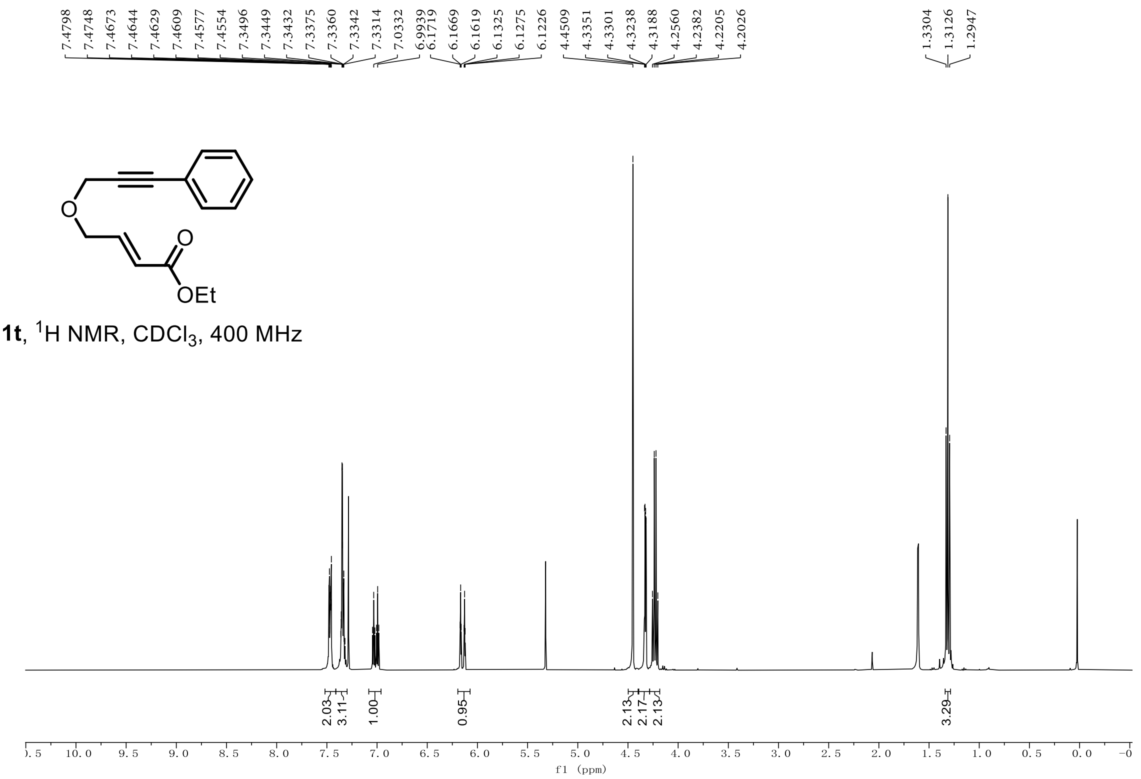

Supplementary Figure 49. ^{1}H NMR (400 MHz, $CDCl_3$, 25 °C) spectra for **1t**

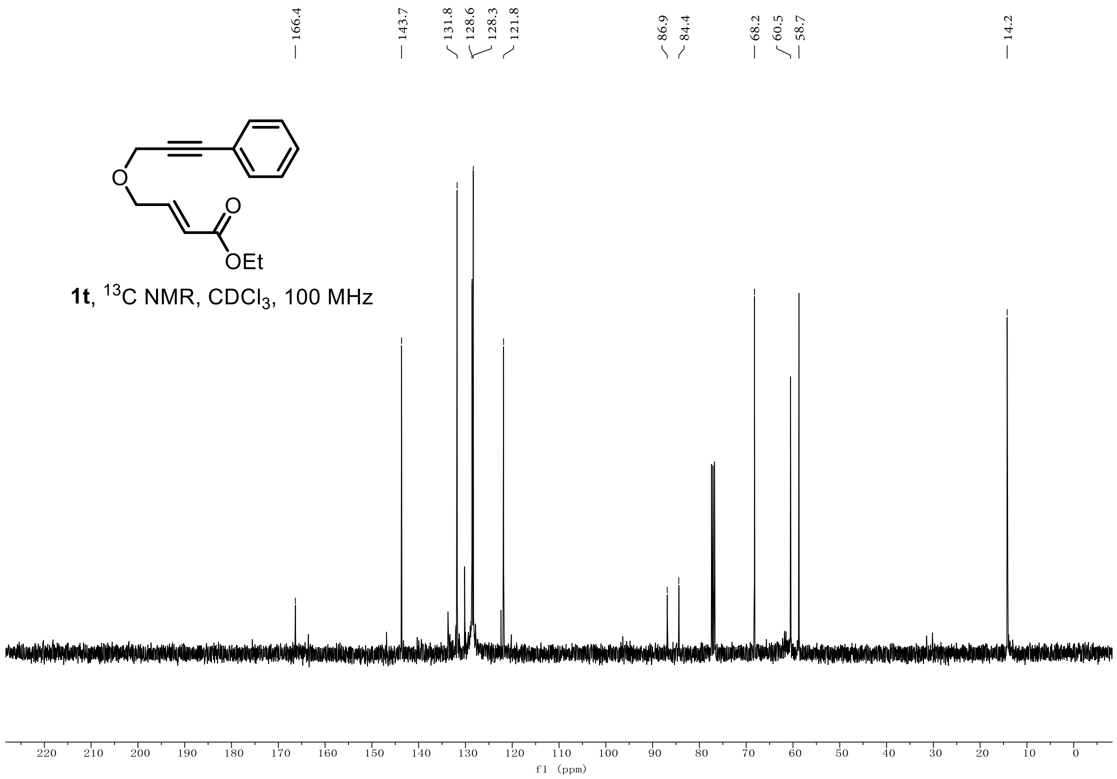

Supplementary Figure 50. ^{13}C NMR (400 MHz, $CDCl_3$, 25 °C) spectra for **1t**

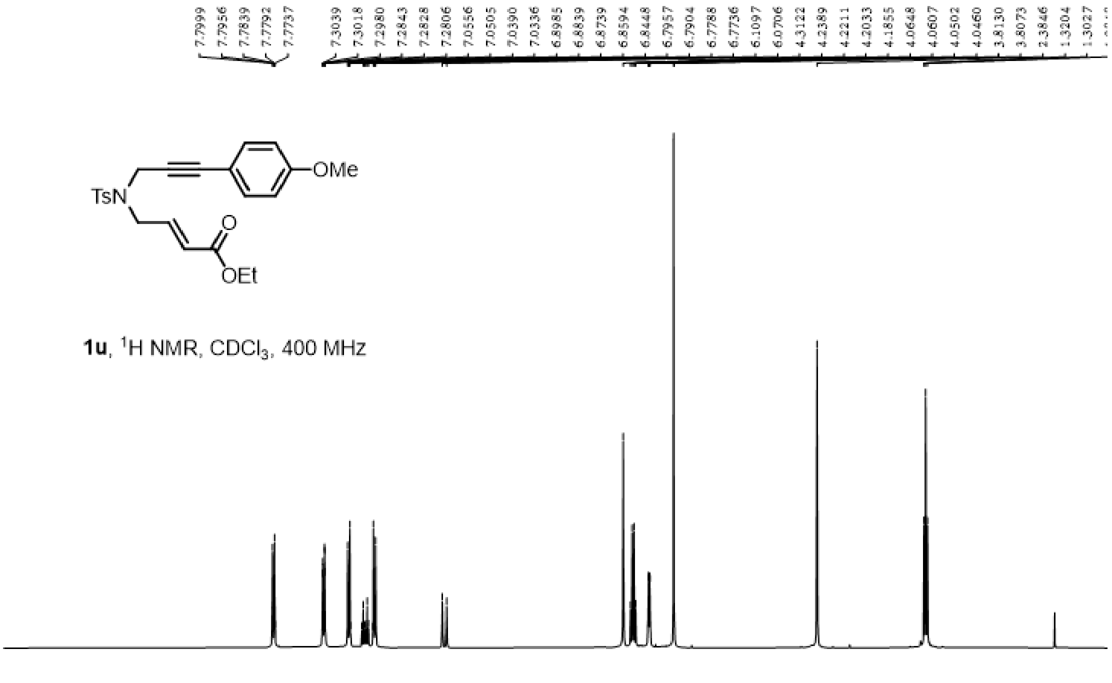

Supplementary Figure 51. ^{1}H NMR (400 MHz, $CDCl_3$, 25 °C) spectra for **1u**

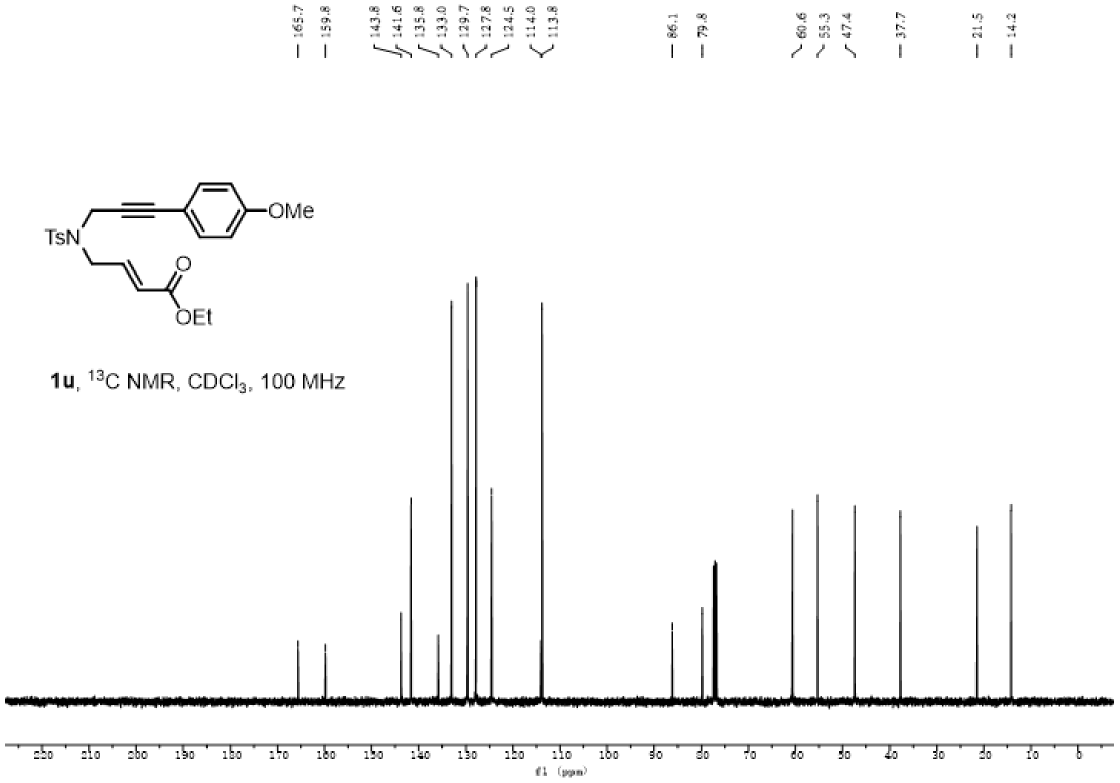

Supplementary Figure 52. ^{13}C NMR (400 MHz, $CDCl_3$, 25 °C) spectra for **1u**

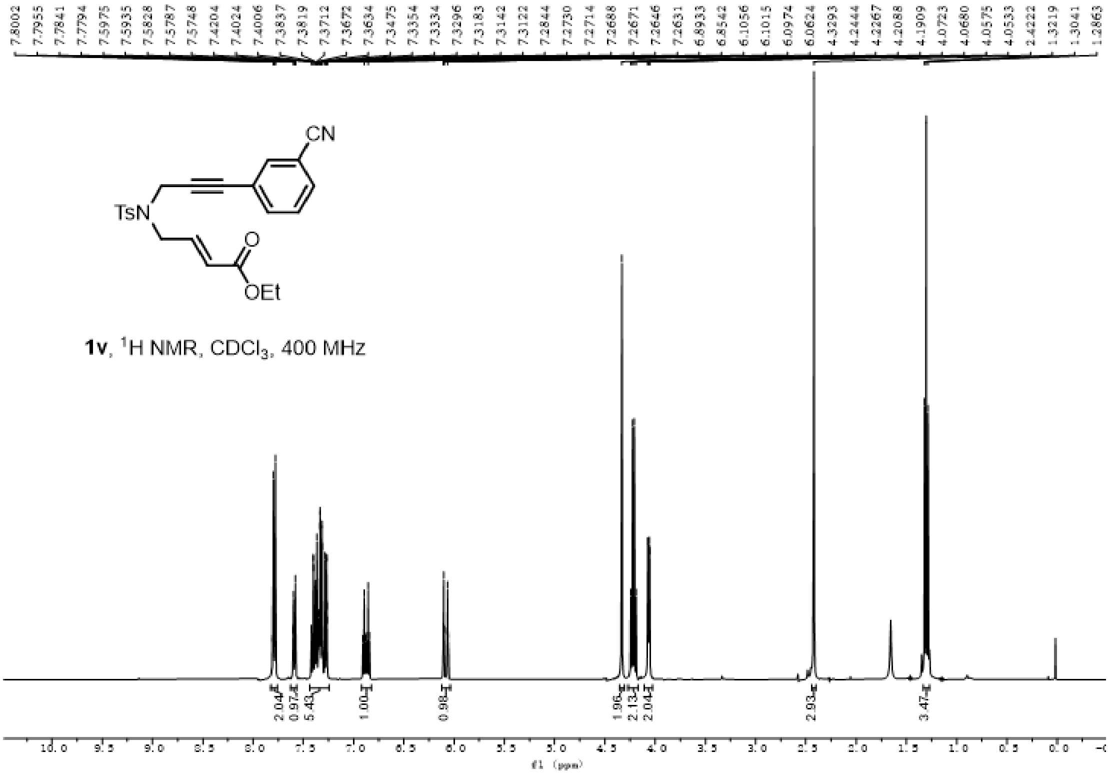

Supplementary Figure 53. ^{1}H NMR (400 MHz, $CDCl_3$, 25 °C) spectra for **1v**

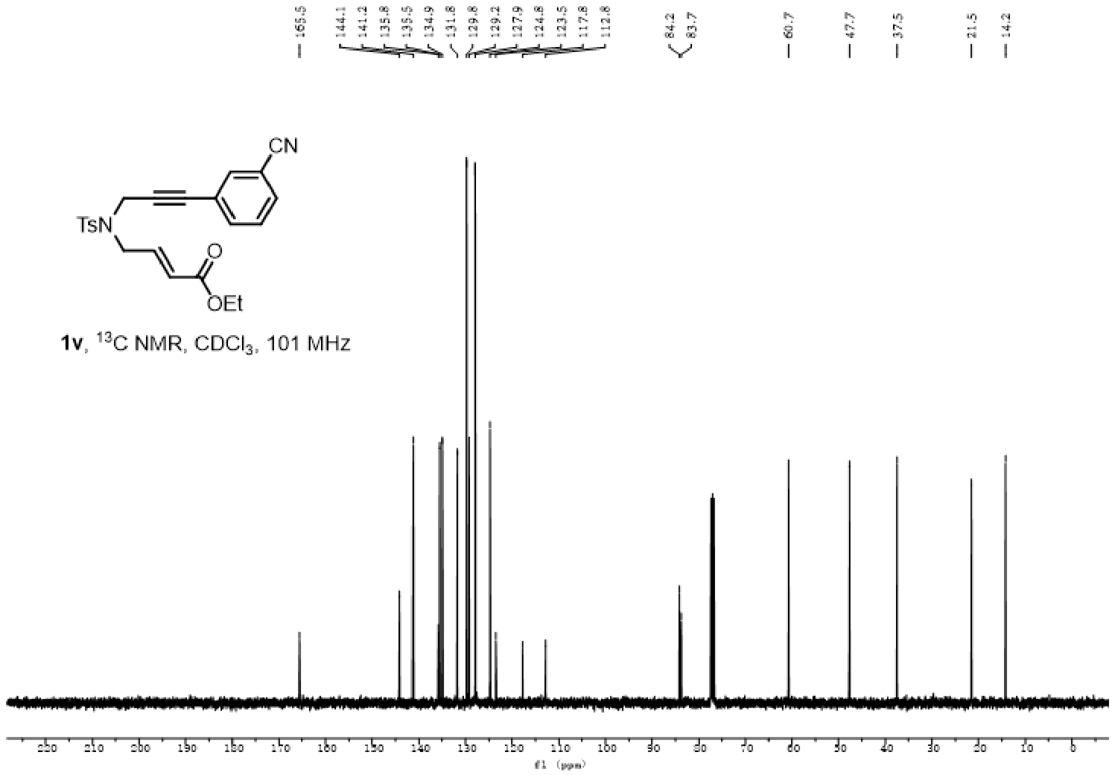

Supplementary Figure 54. ^{13}C NMR (400 MHz, $CDCl_3$, 25 °C) spectra for **1v**

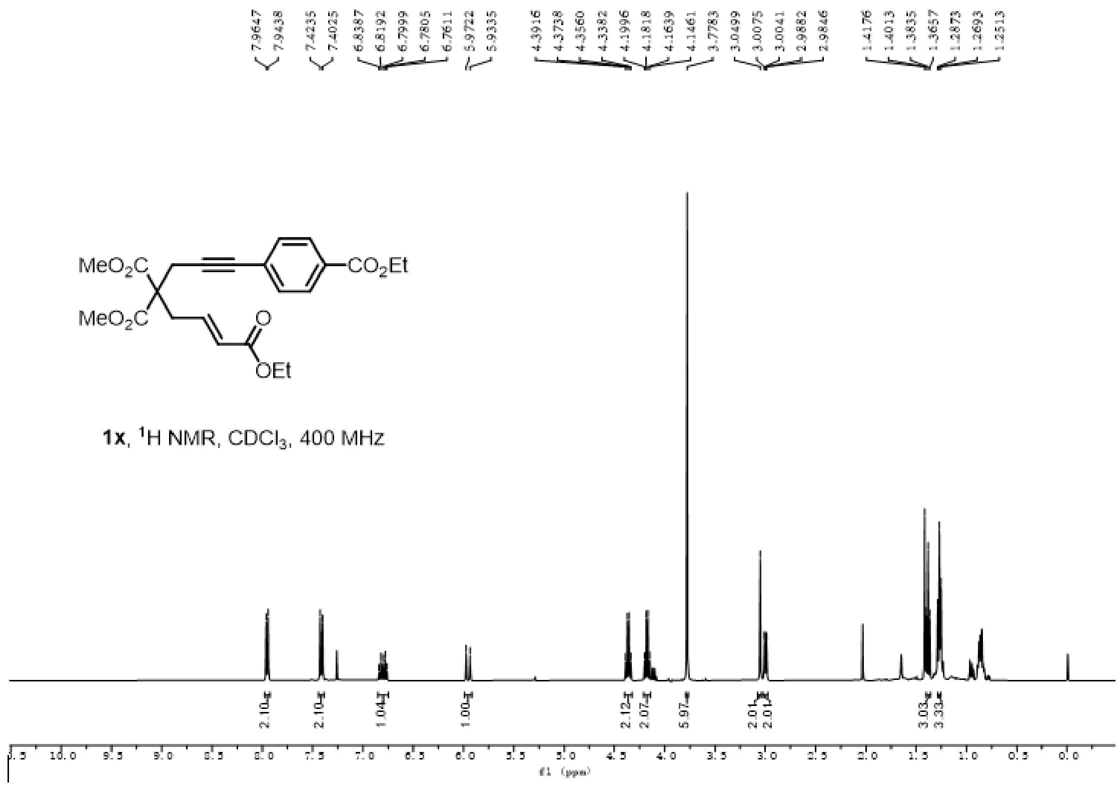

Supplementary Figure 55. ^{1}H NMR (400 MHz, $CDCl_3$, 25 °C) spectra for **1x**

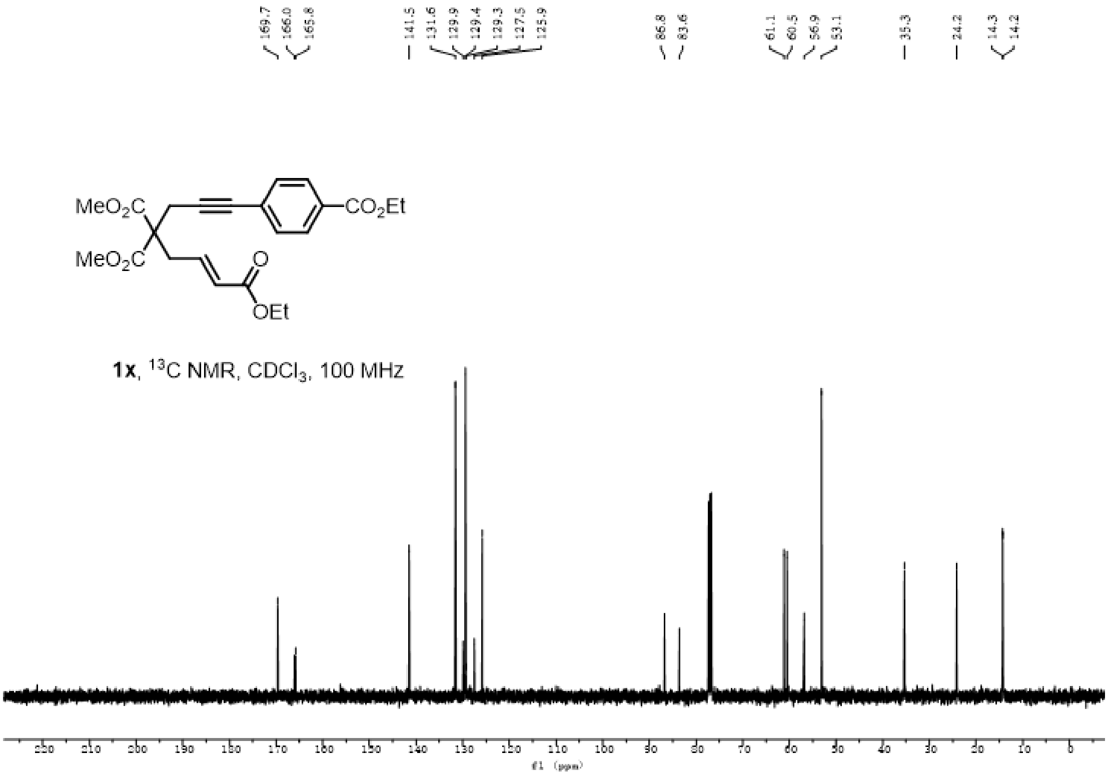

Supplementary Figure 56. ^{13}C NMR (400 MHz, $CDCl_3$, 25 °C) spectra for **1x**

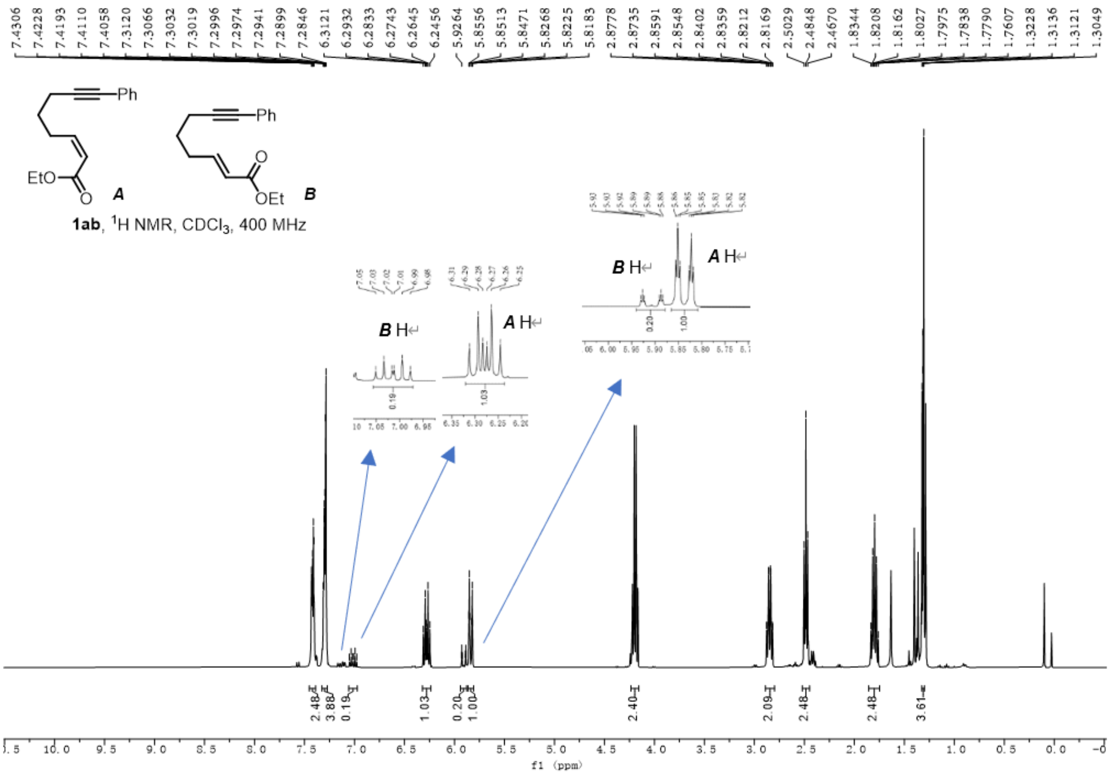

Supplementary Figure 57. ^{1}H NMR (400 MHz, $CDCl_3$, 25 °C) spectra for **1ab**

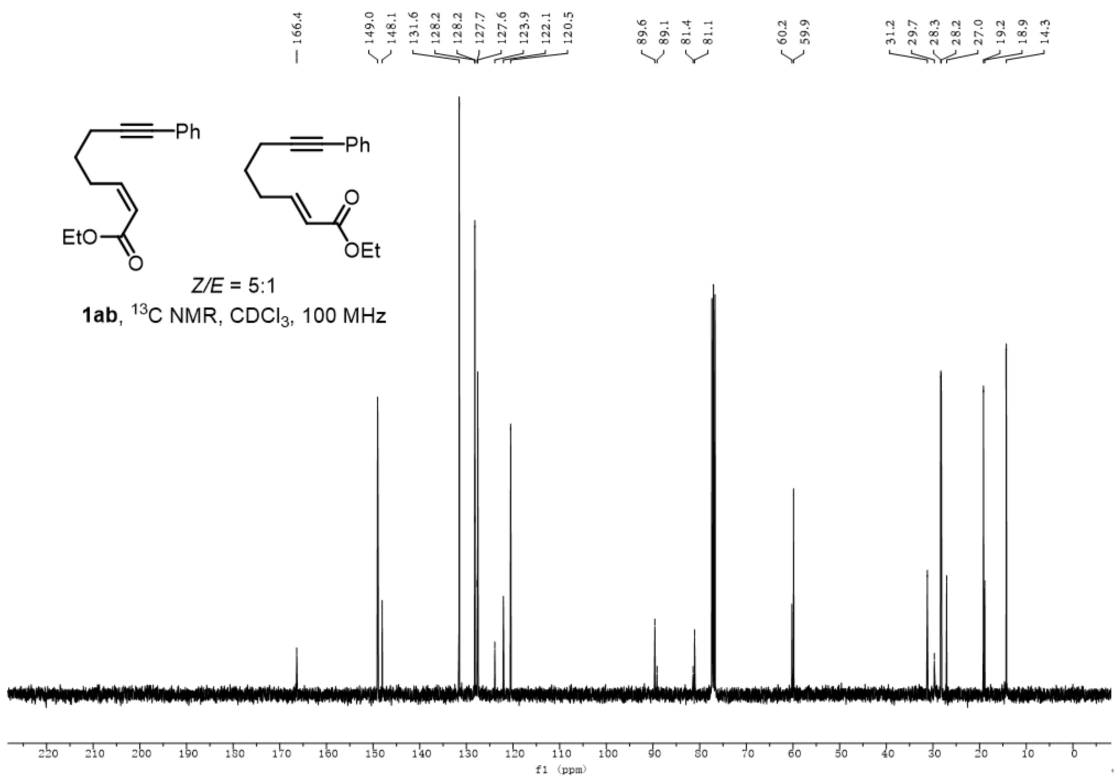

Supplementary Figure 58. ^{13}C NMR (400 MHz, $CDCl_3$, 25 °C) spectra for **1ab**

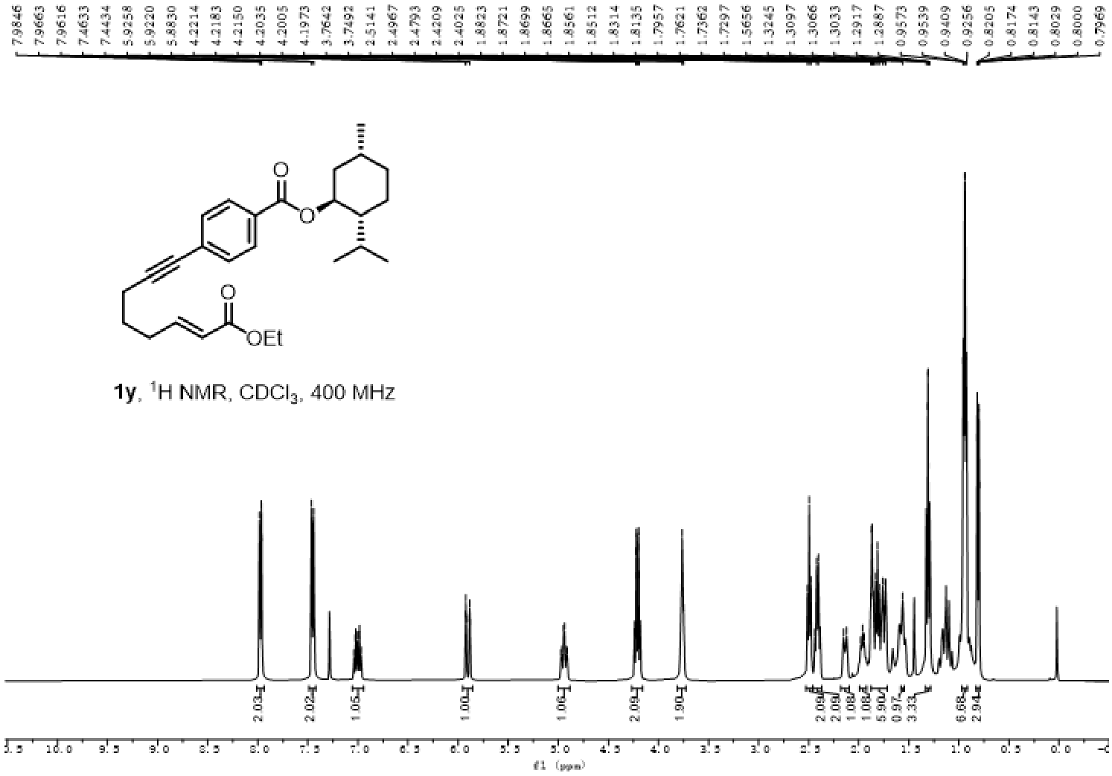

Supplementary Figure 59. ^{1}H NMR (400 MHz, $CDCl_3$, 25 °C) spectra for **1y**

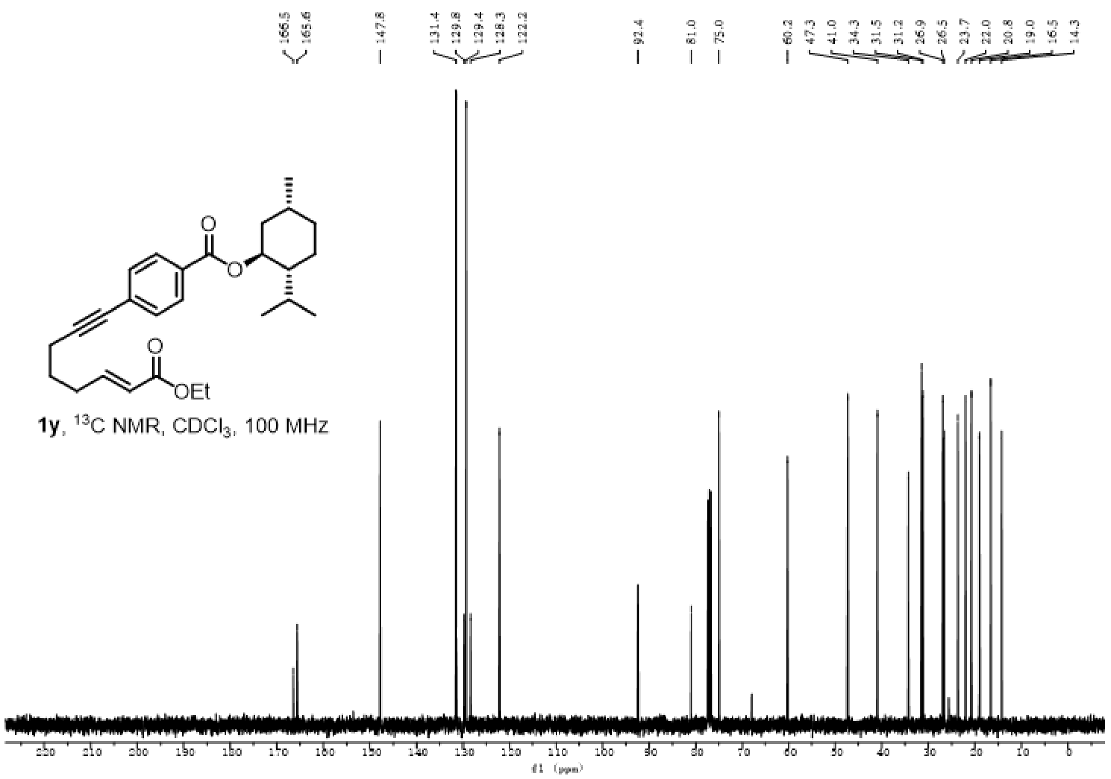

Supplementary Figure 60. ^{13}C NMR (400 MHz, $CDCl_3$, 25 °C) spectra for **1y**

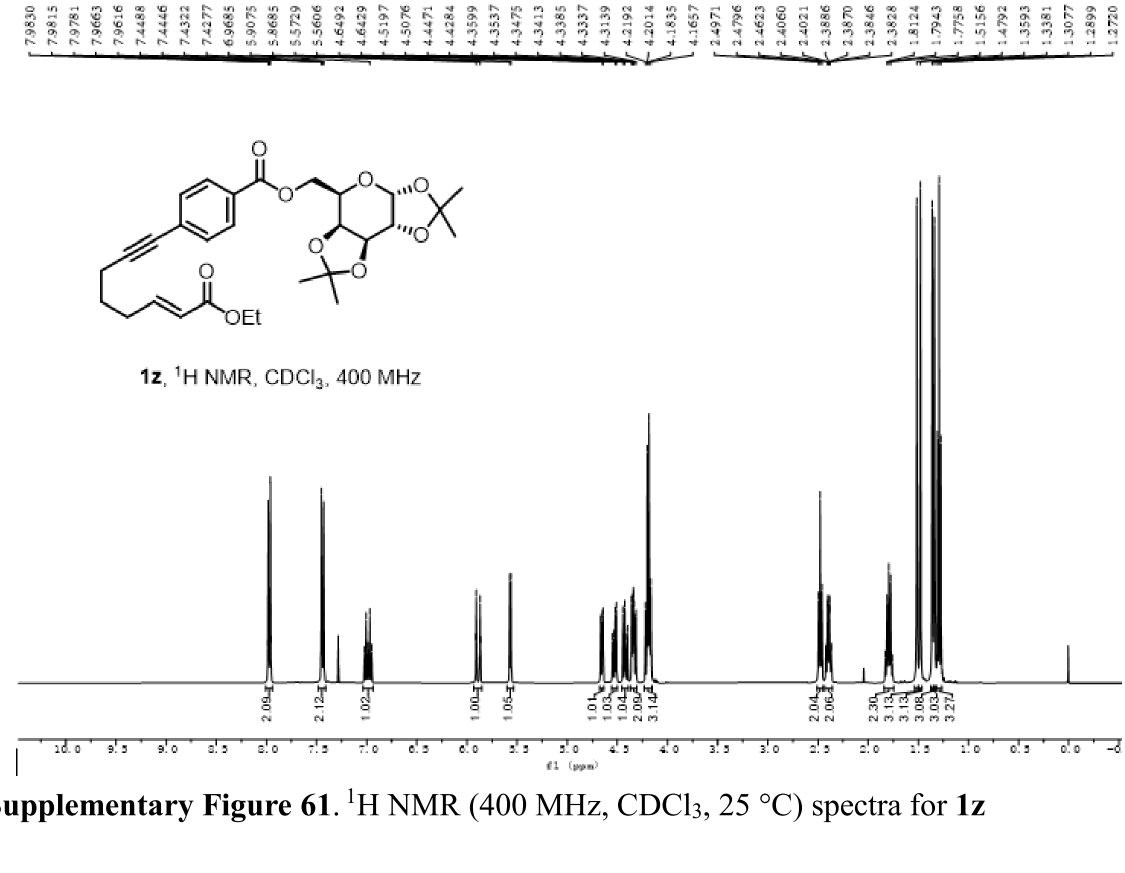

Supplementary Figure 61. ^{1}H NMR (400 MHz, $CDCl_3$, 25 °C) spectra for **1z**

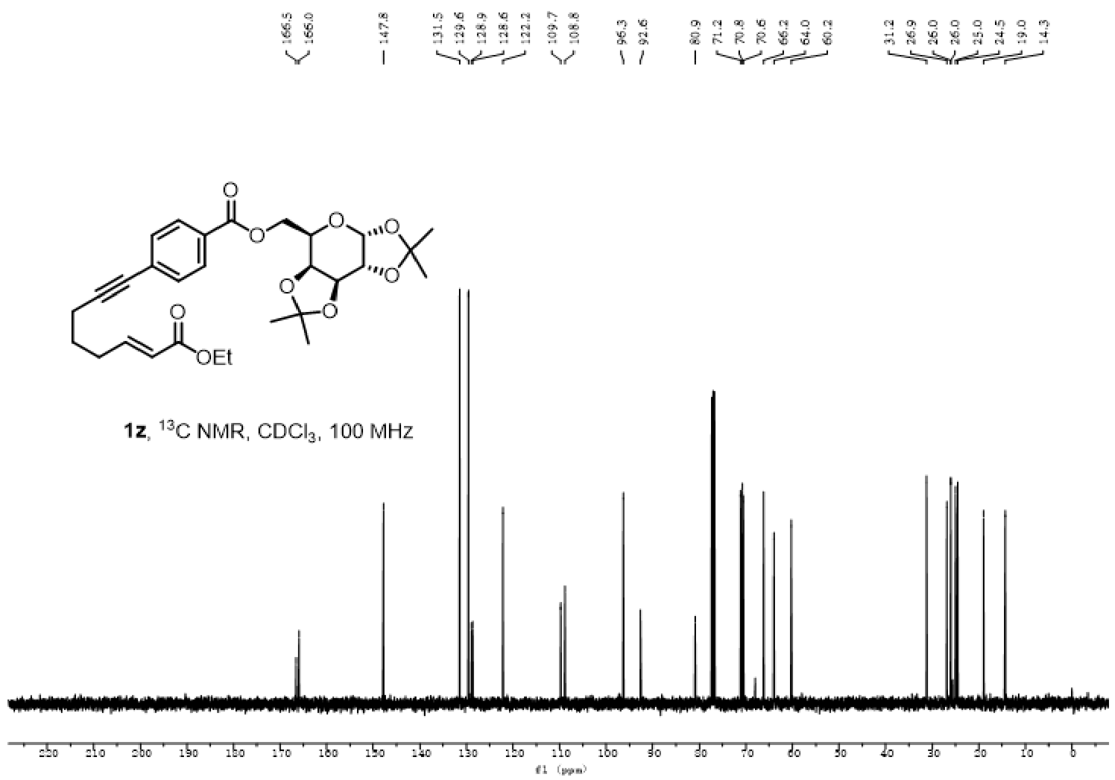

Supplementary Figure 62. ^{13}C NMR (400 MHz, $CDCl_3$, 25 °C) spectra for **1z**

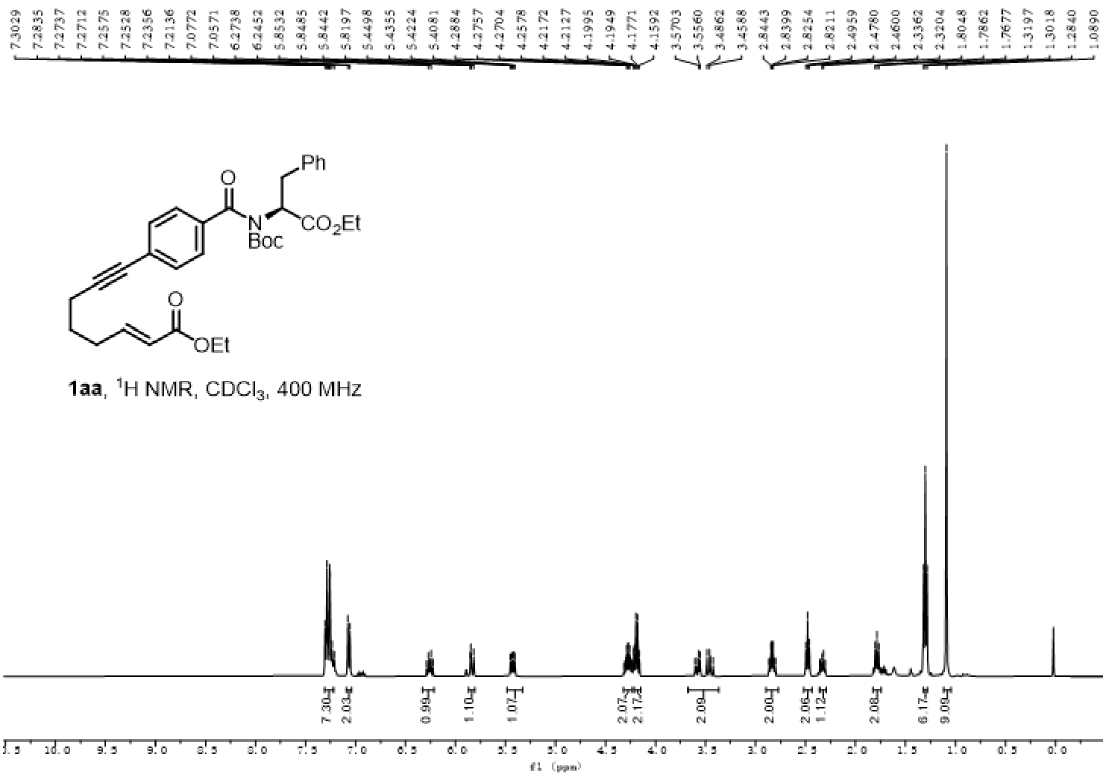

Supplementary Figure 63. ^{1}H NMR (400 MHz, $CDCl_3$, 25 °C) spectra for **1aa**

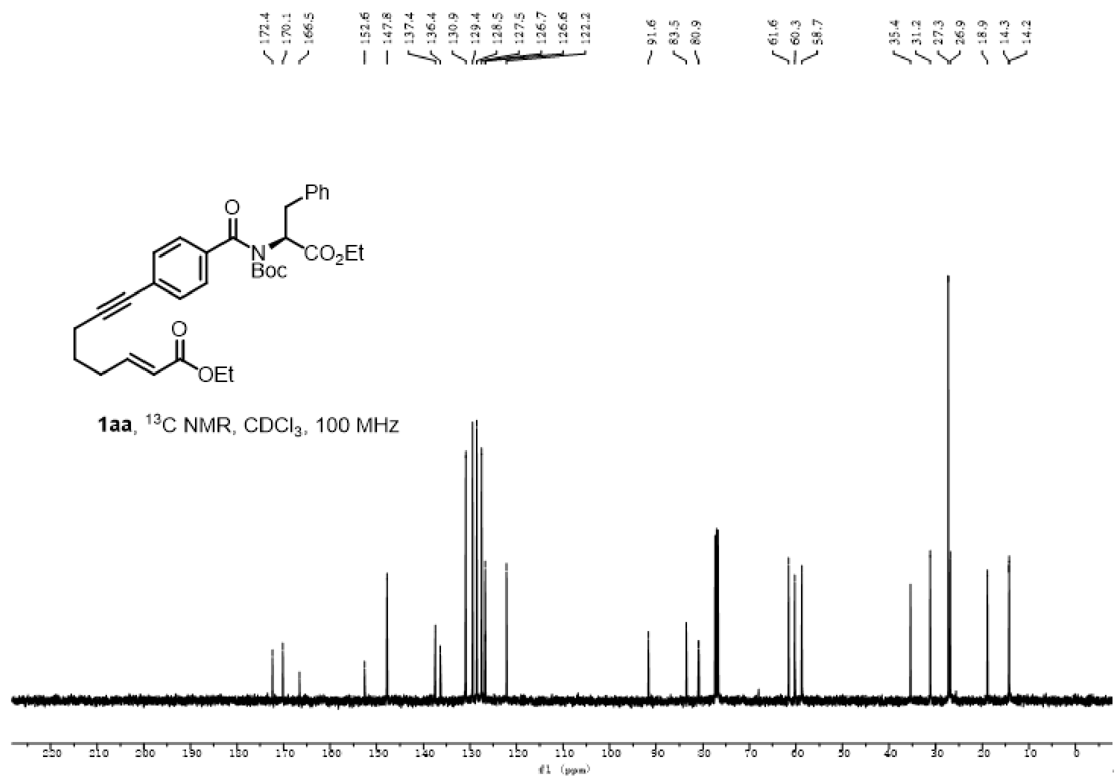

Supplementary Figure 64. ^{13}C NMR (400 MHz, $CDCl_3$, 25 °C) spectra for **1aa**

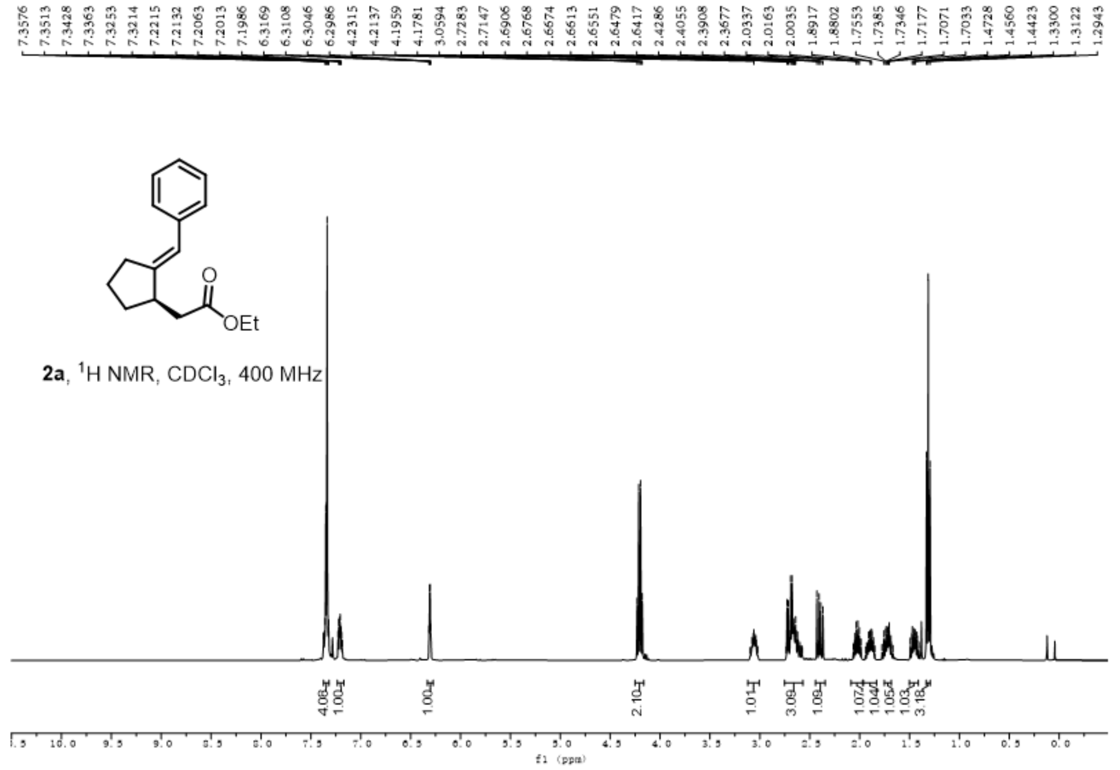

Supplementary Figure 65. ^{1}H NMR (400 MHz, $CDCl_3$, 25 °C) spectra for **2a**

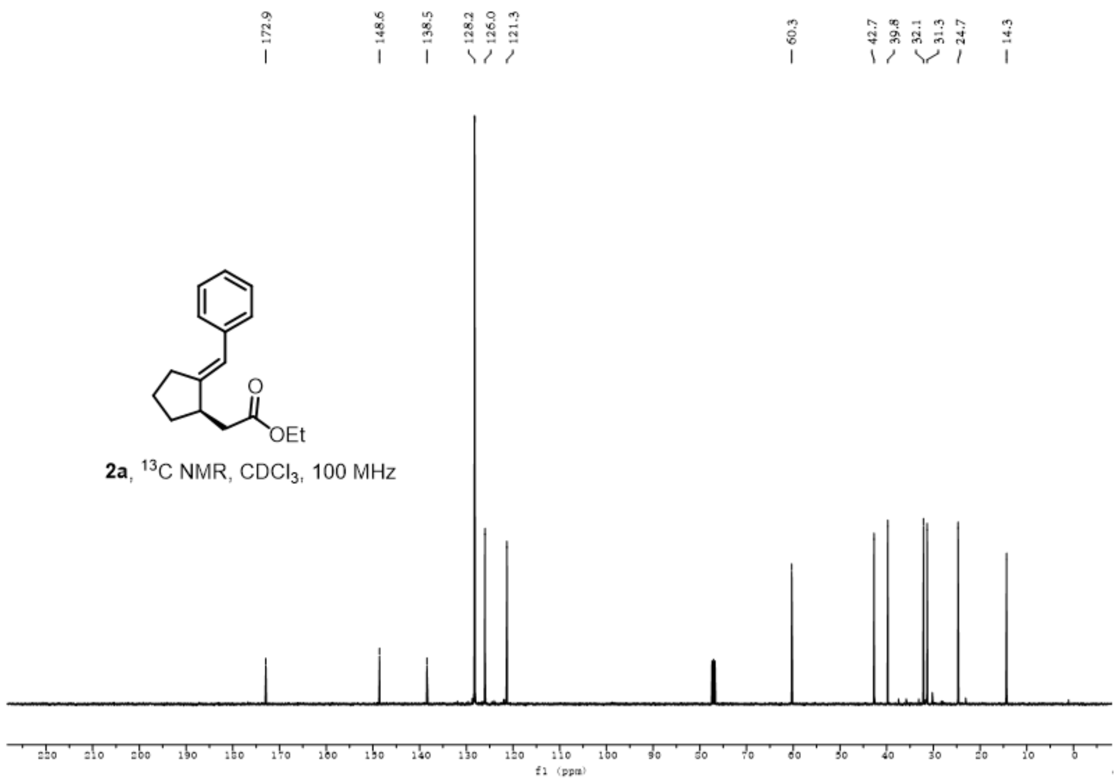

Supplementary Figure 66. ^{13}C NMR (400 MHz, $CDCl_3$, 25 °C) spectra for **2a**

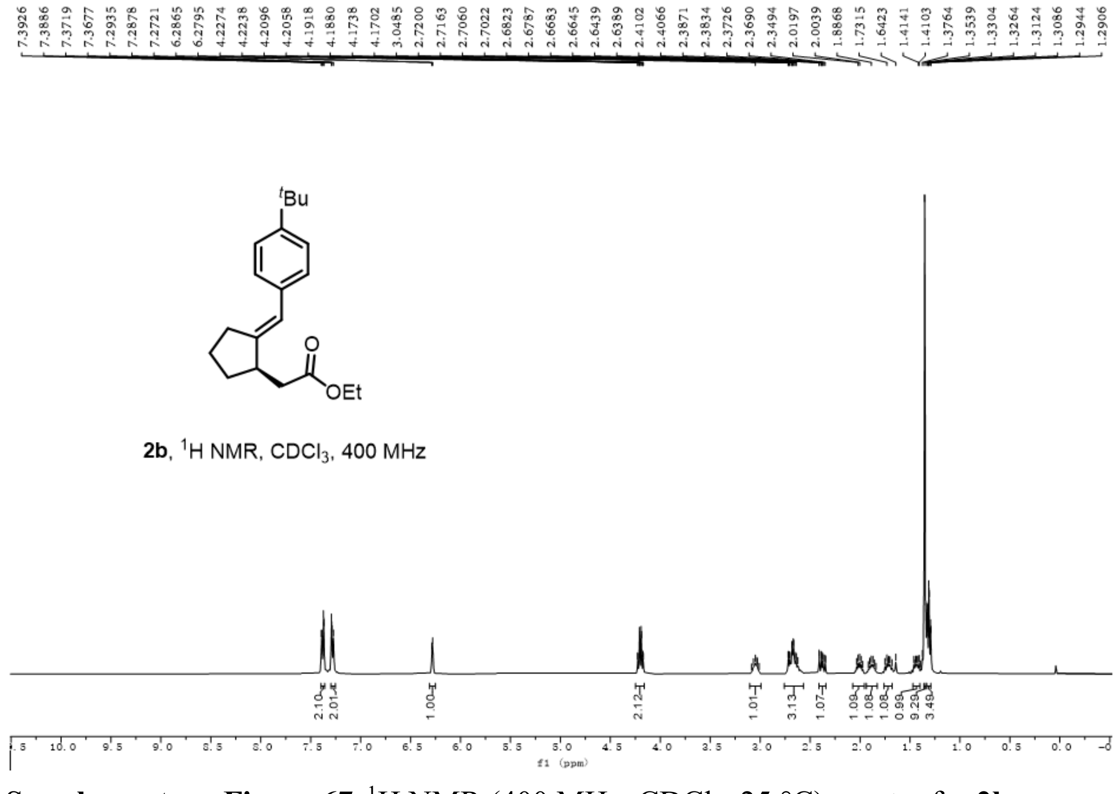

Supplementary Figure 67. ^{1}H NMR (400 MHz, $CDCl_3$, 25 °C) spectra for **2b**

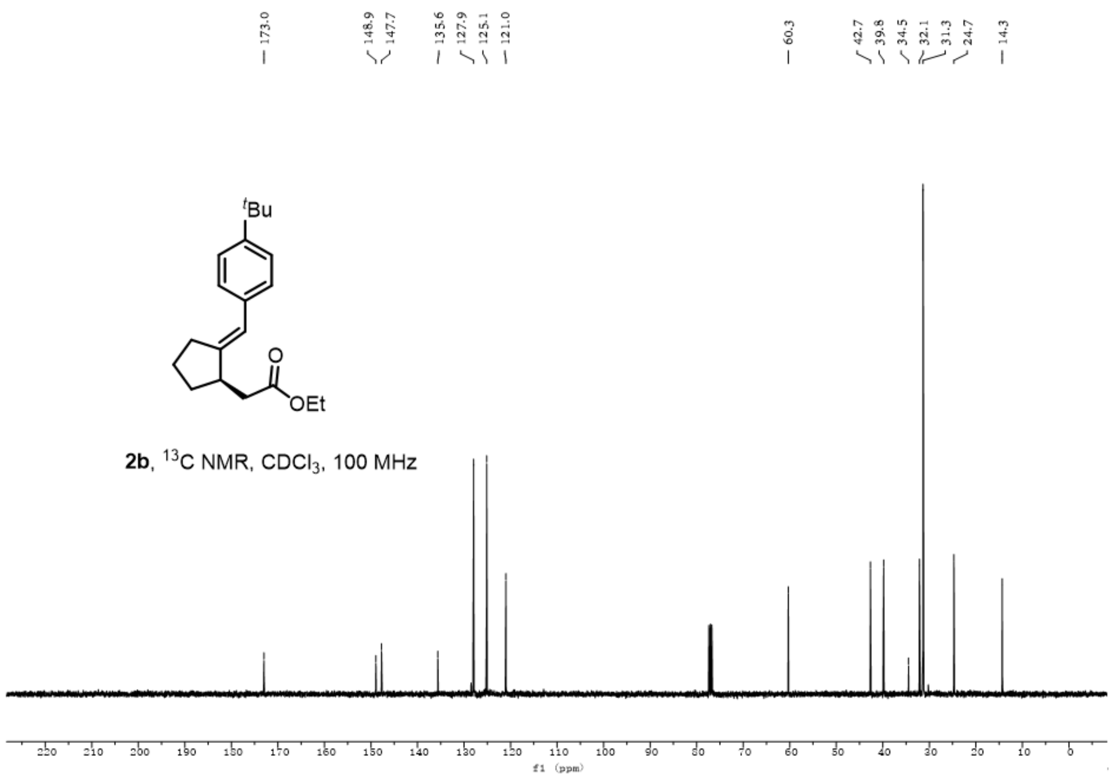

Supplementary Figure 68. ^{13}C NMR (400 MHz, $CDCl_3$, 25 °C) spectra for **2b**

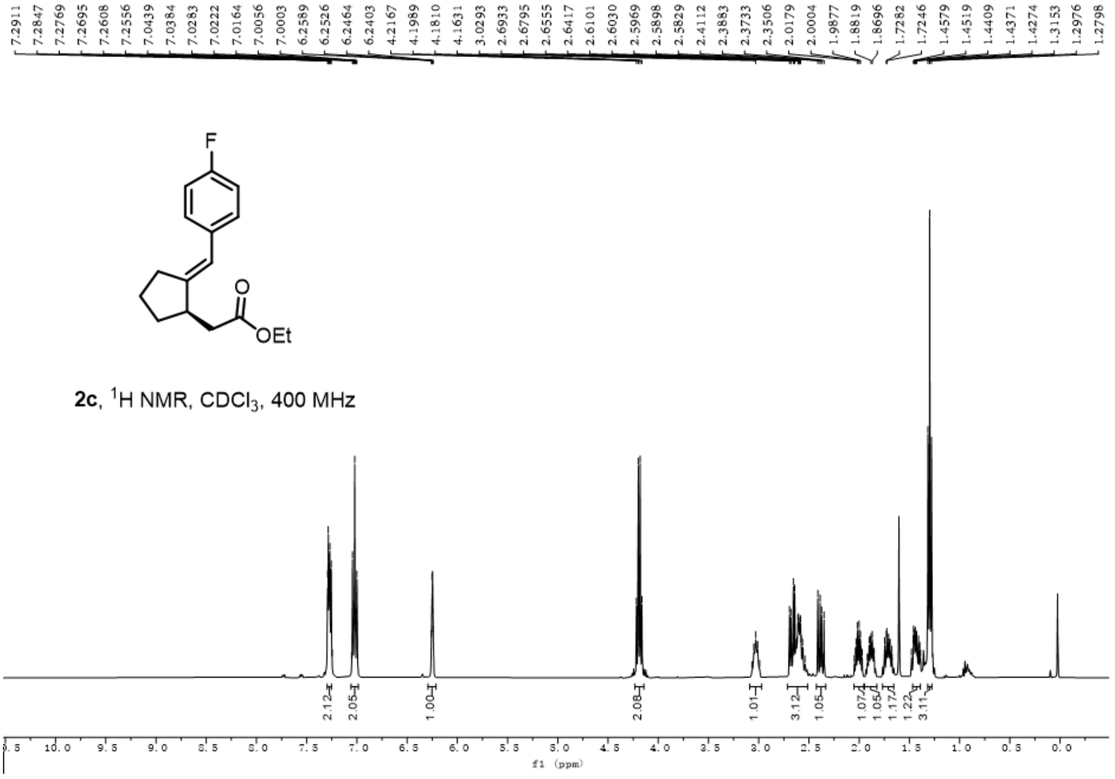

Supplementary Figure 69. ^{1}H NMR (400 MHz, $CDCl_3$, 25 °C) spectra for **2c**

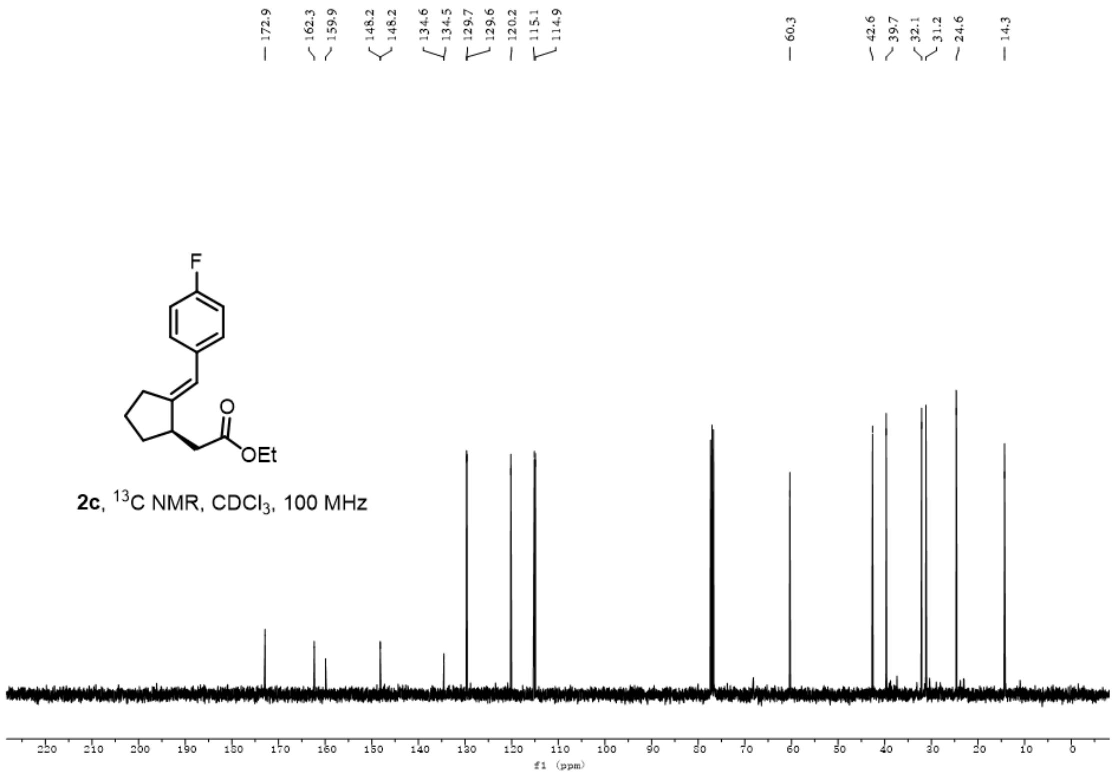

Supplementary Figure 70. ^{13}C NMR (400 MHz, $CDCl_3$, 25 °C) spectra for **2c**

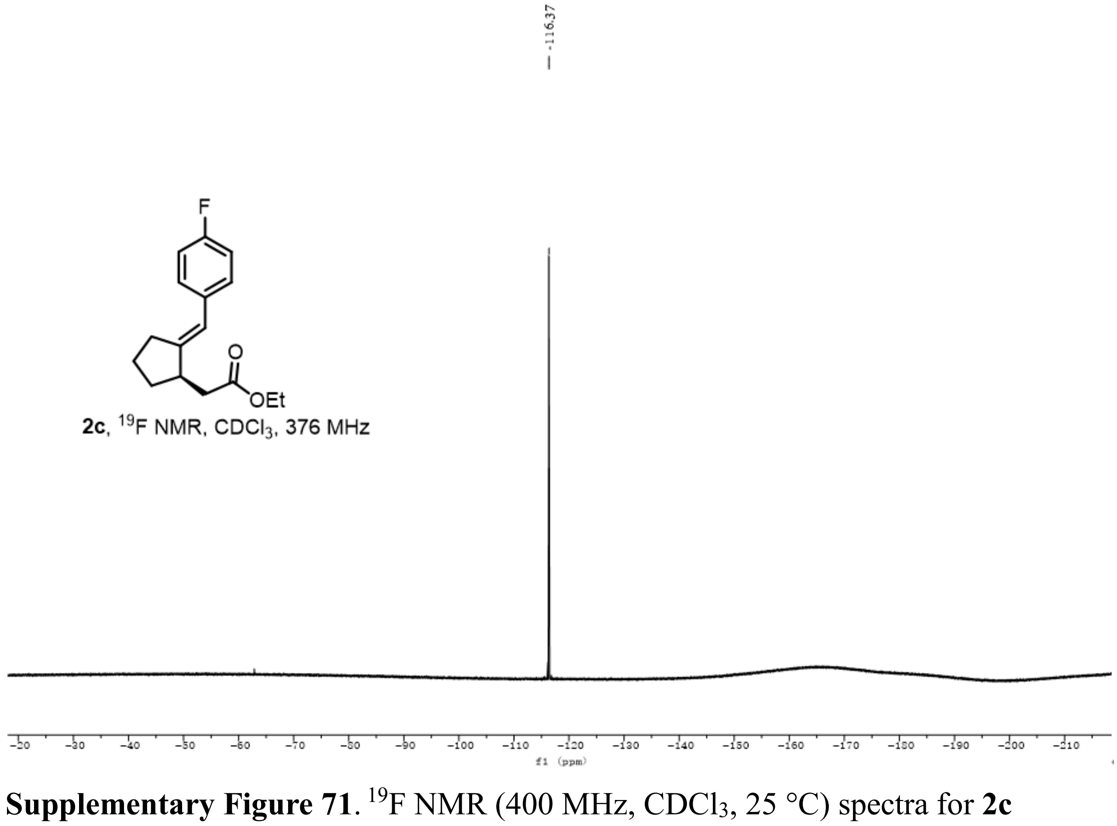

Supplementary Figure 71. ^{19}F NMR (400 MHz, $CDCl_3$, 25 °C) spectra for **2c**

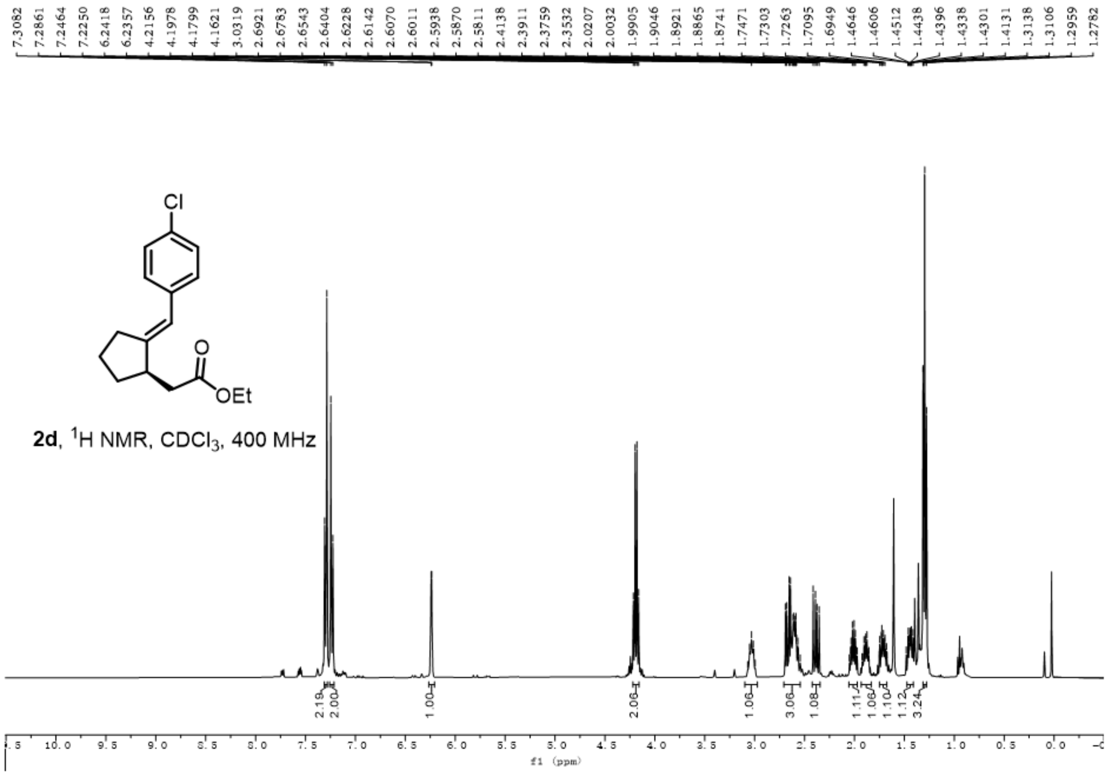

Supplementary Figure 72. ^{1}H NMR (400 MHz, $CDCl_3$, 25 °C) spectra for **2d**

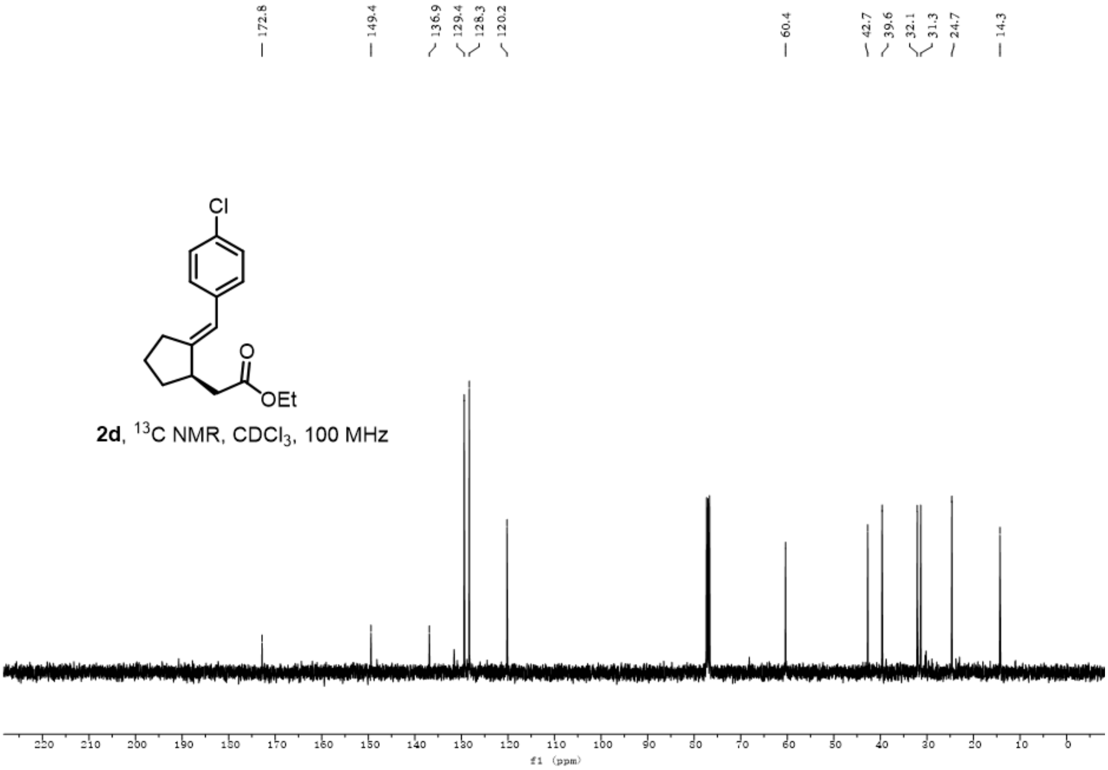

Supplementary Figure 73. ^{13}C NMR (400 MHz, $CDCl_3$, 25 °C) spectra for **2d**

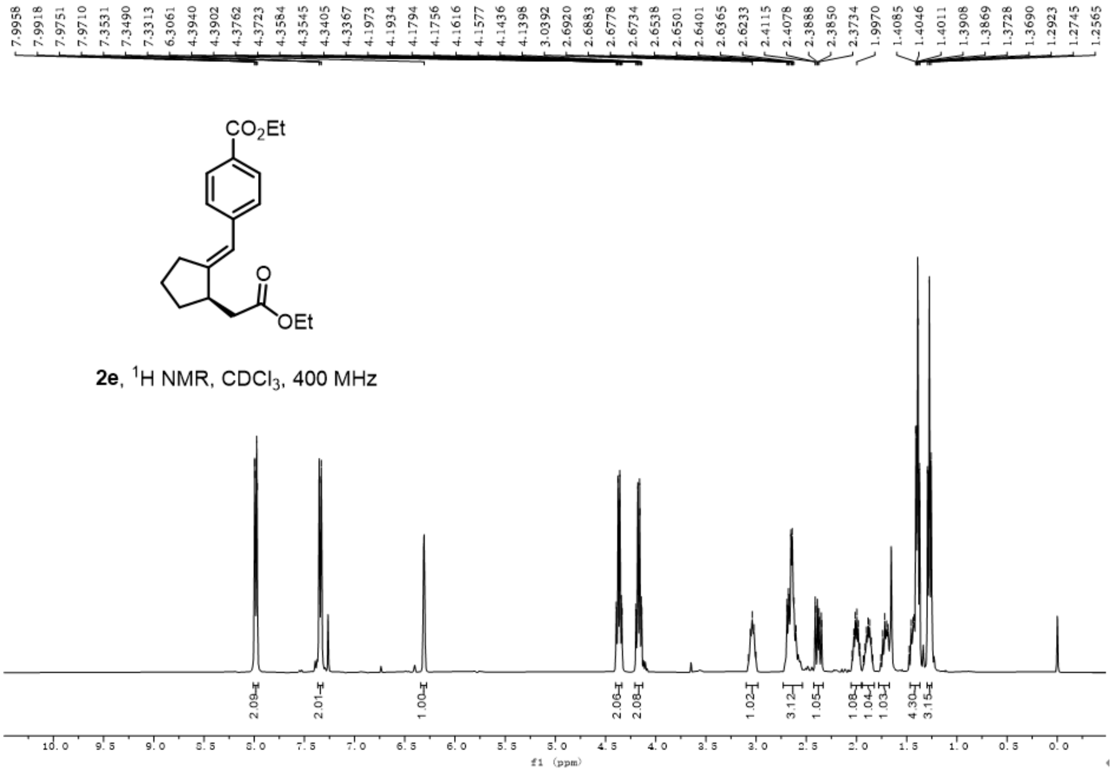

Supplementary Figure 74. ^{1}H NMR (400 MHz, $CDCl_3$, 25 °C) spectra for **2e**

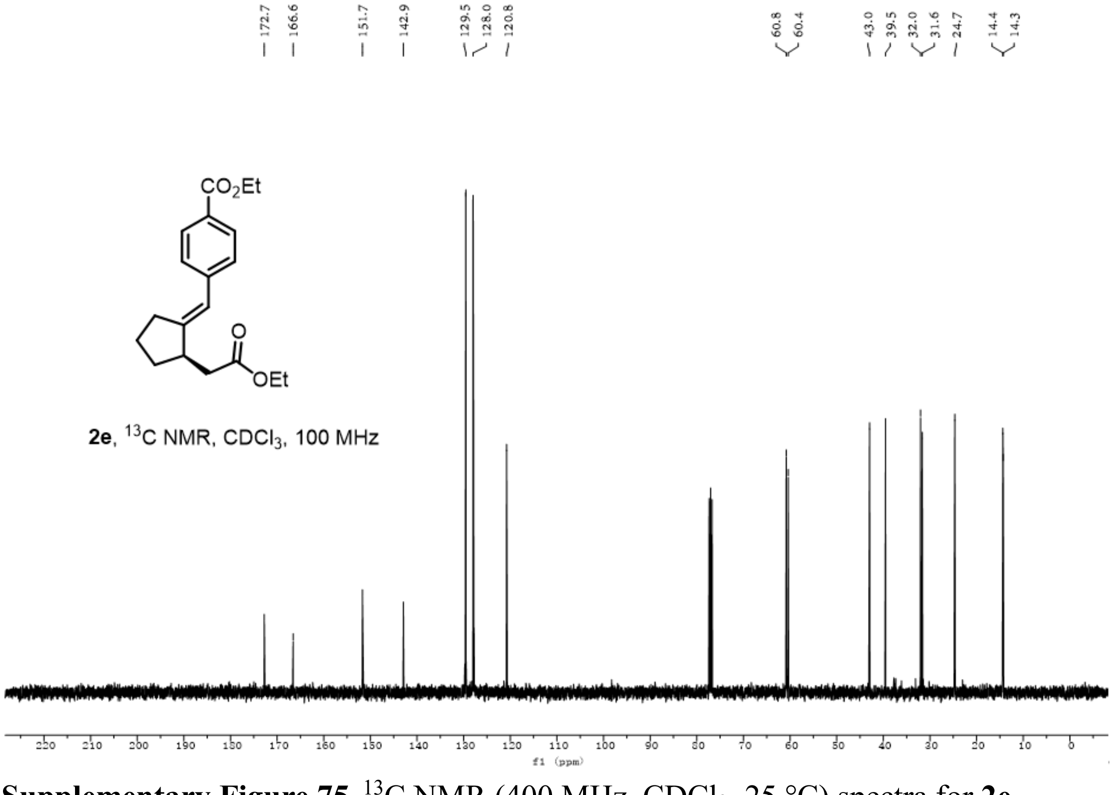

Supplementary Figure 75. ^{13}C NMR (400 MHz, $CDCl_3$, 25 °C) spectra for **2e**

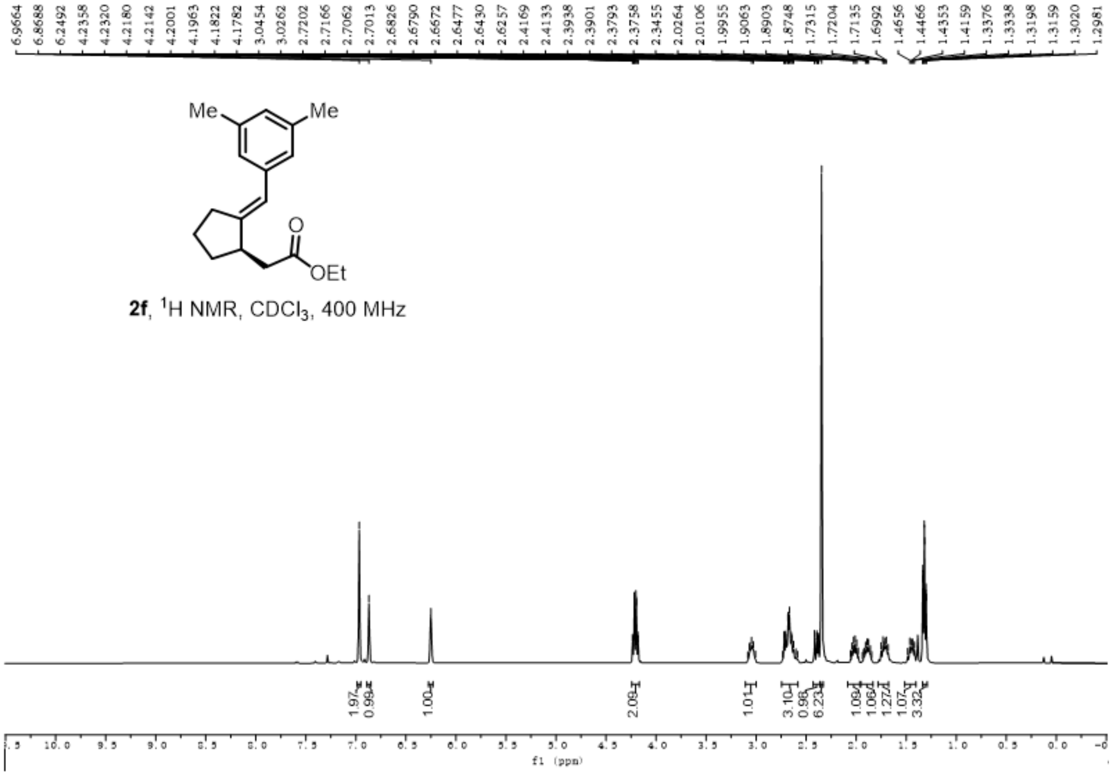

Supplementary Figure 76. ^{1}H NMR (400 MHz, $CDCl_3$, 25 °C) spectra for **2f**

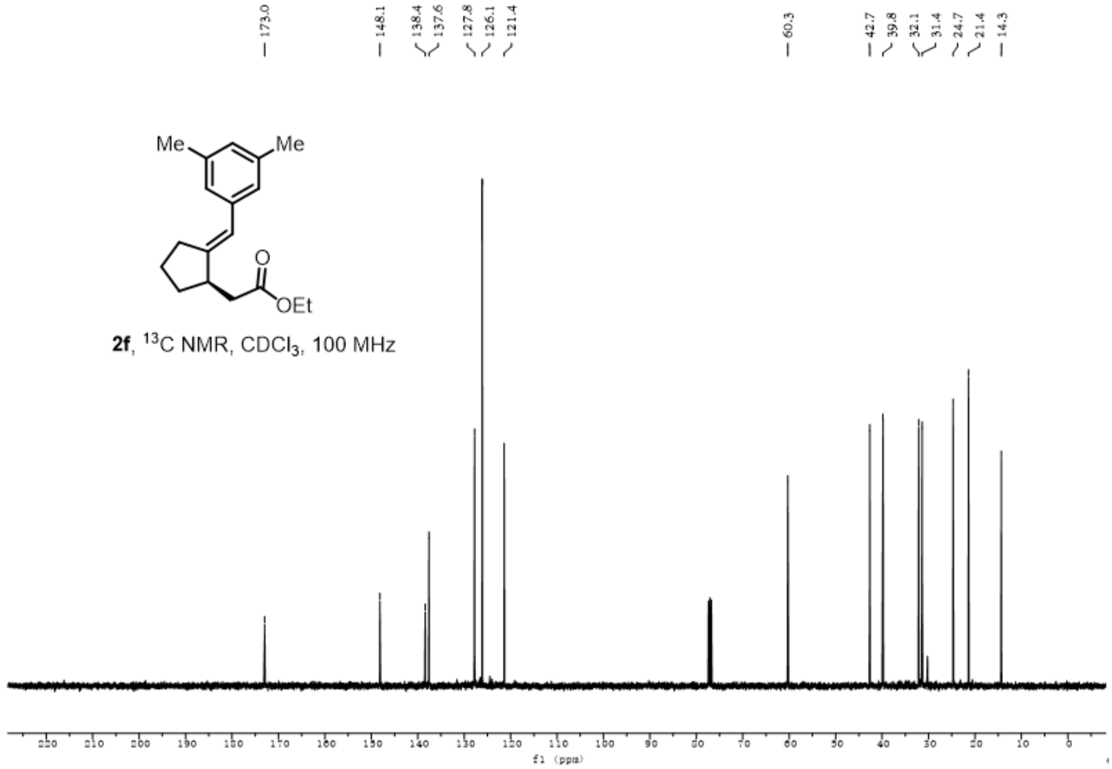

Supplementary Figure 77. ^{13}C NMR (400 MHz, $CDCl_3$, 25 °C) spectra for **2f**

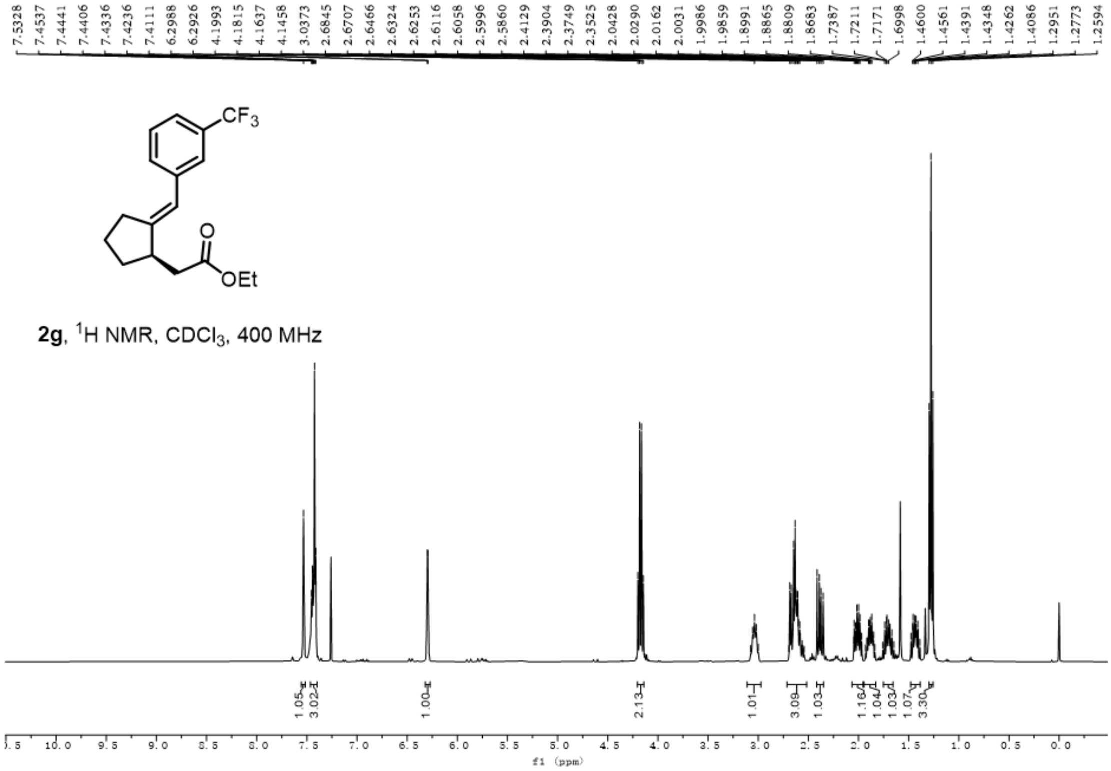

Supplementary Figure 78. ^{1}H NMR (400 MHz, $CDCl_3$, 25 °C) spectra for **2g**

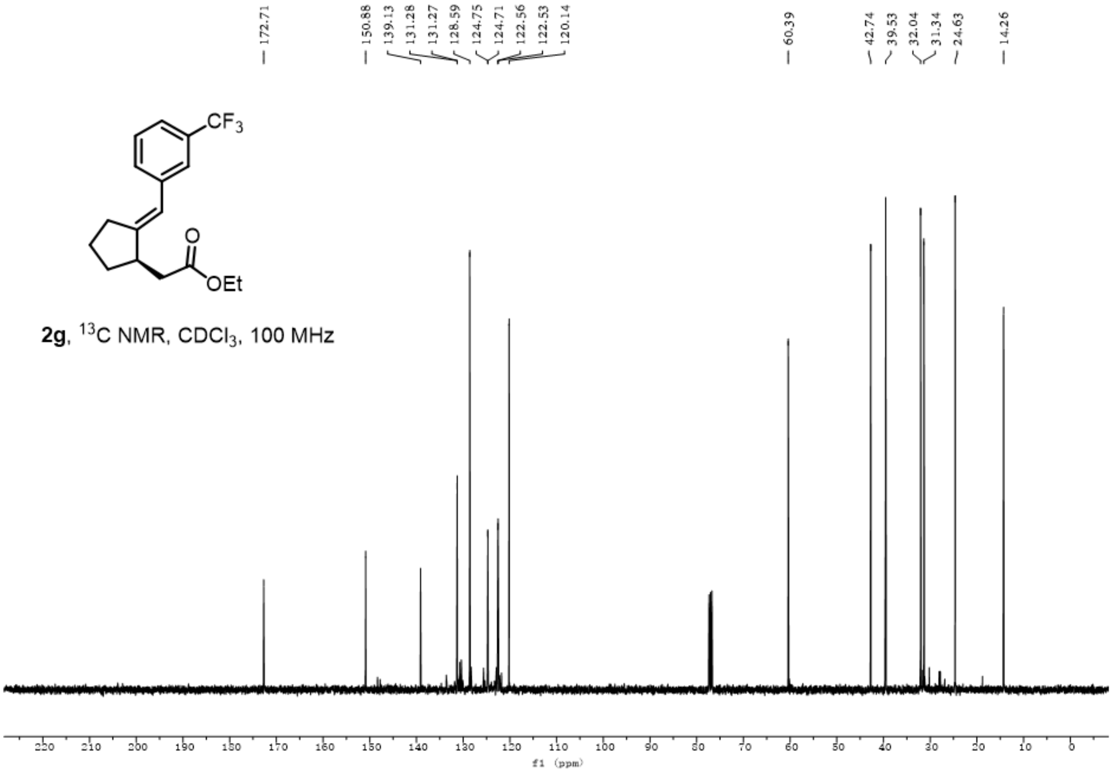

Supplementary Figure 79. ^{13}C NMR (400 MHz, $CDCl_3$, 25 °C) spectra for **2g**

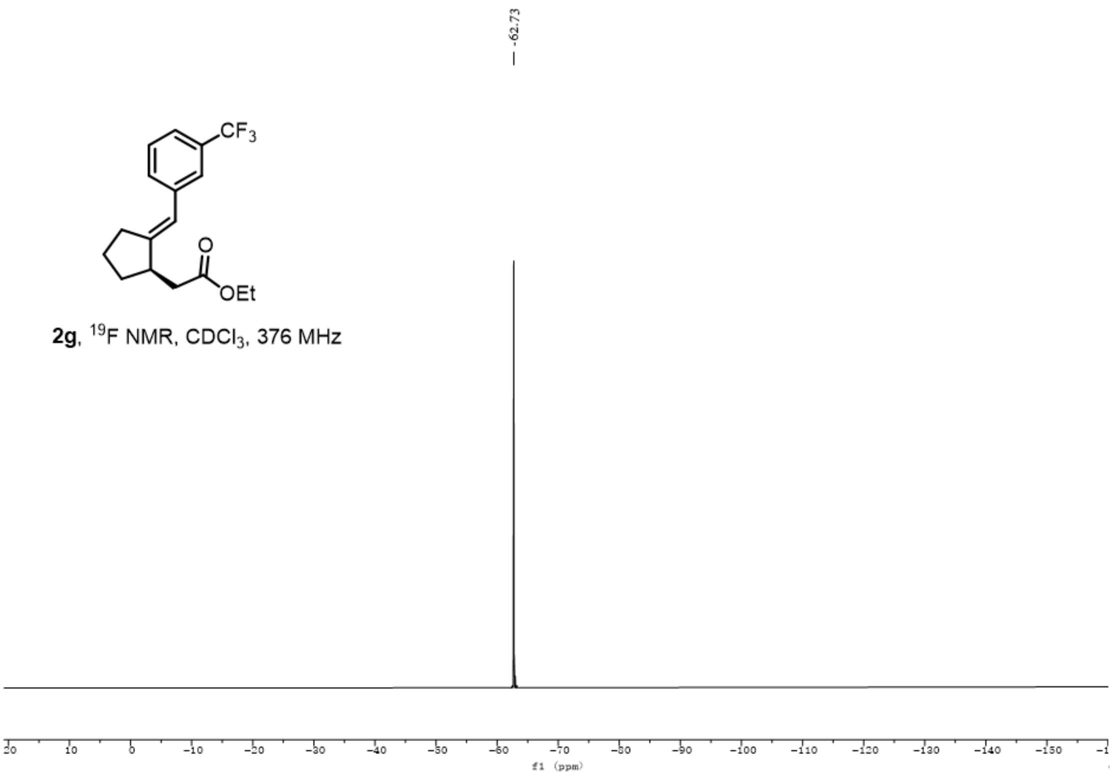

Supplementary Figure 80. ^{19}F NMR (400 MHz, $CDCl_3$, 25 °C) spectra for **2g**

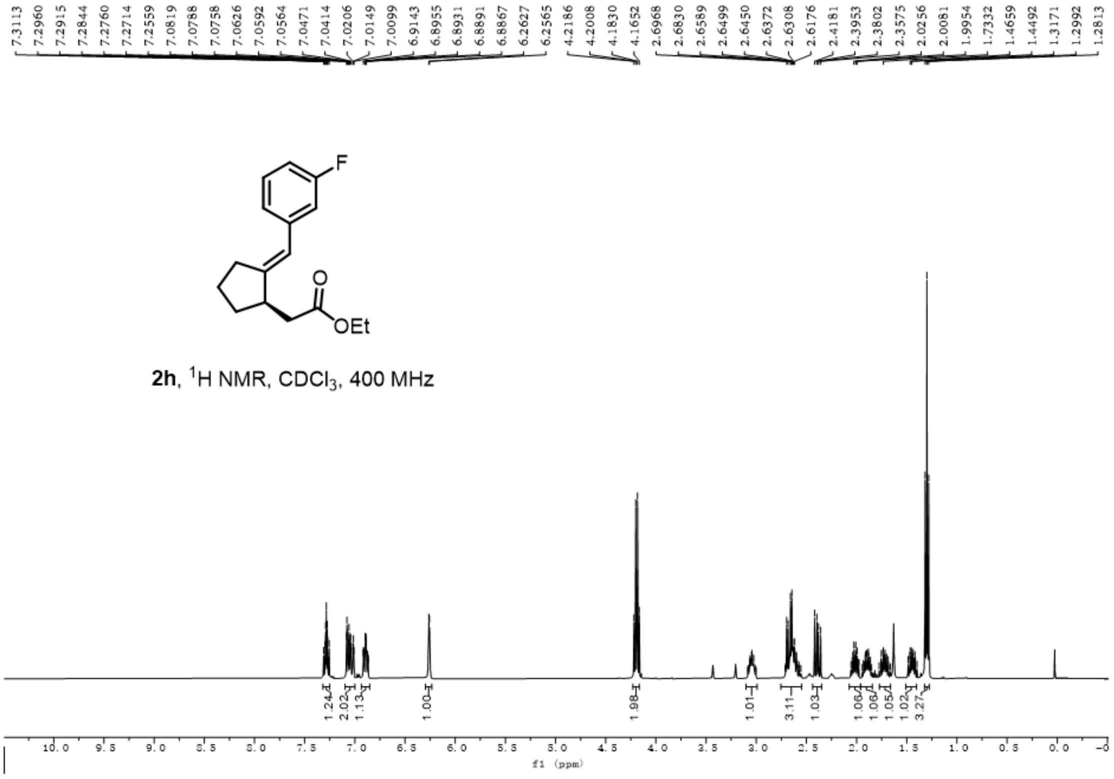

Supplementary Figure 81. ^{1}H NMR (400 MHz, $CDCl_3$, 25 °C) spectra for **2h**

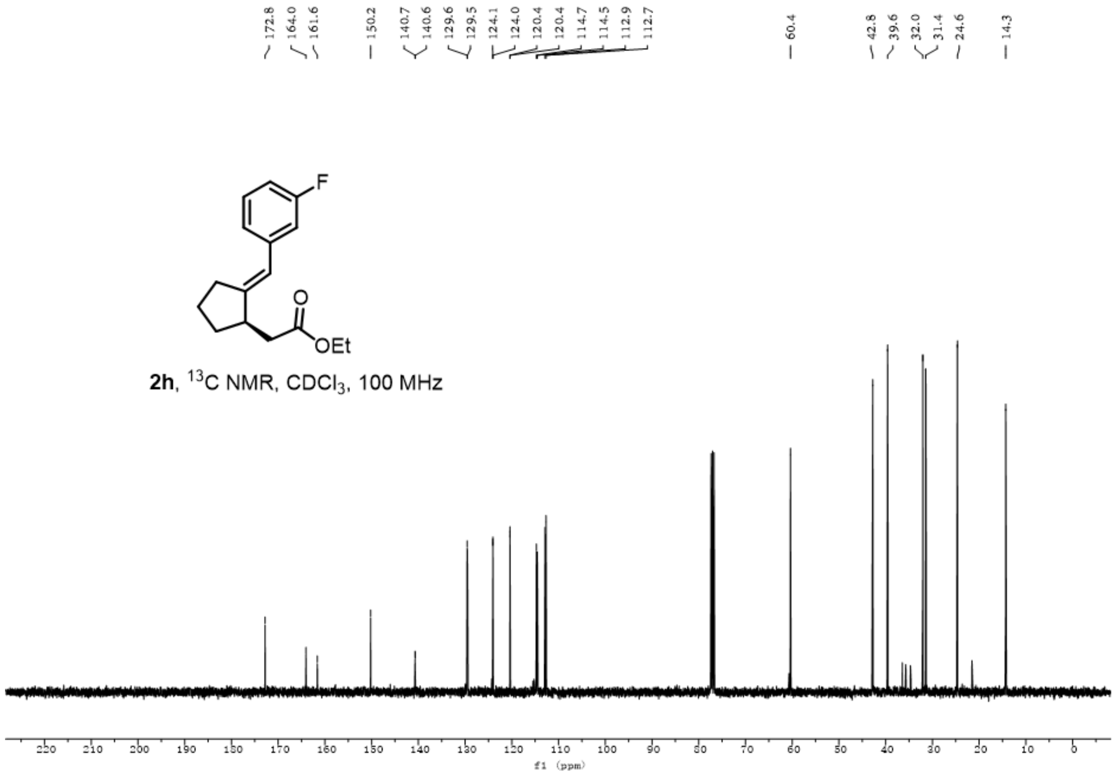

Supplementary Figure 82. ^{13}C NMR (400 MHz, $CDCl_3$, 25 °C) spectra for **2h**

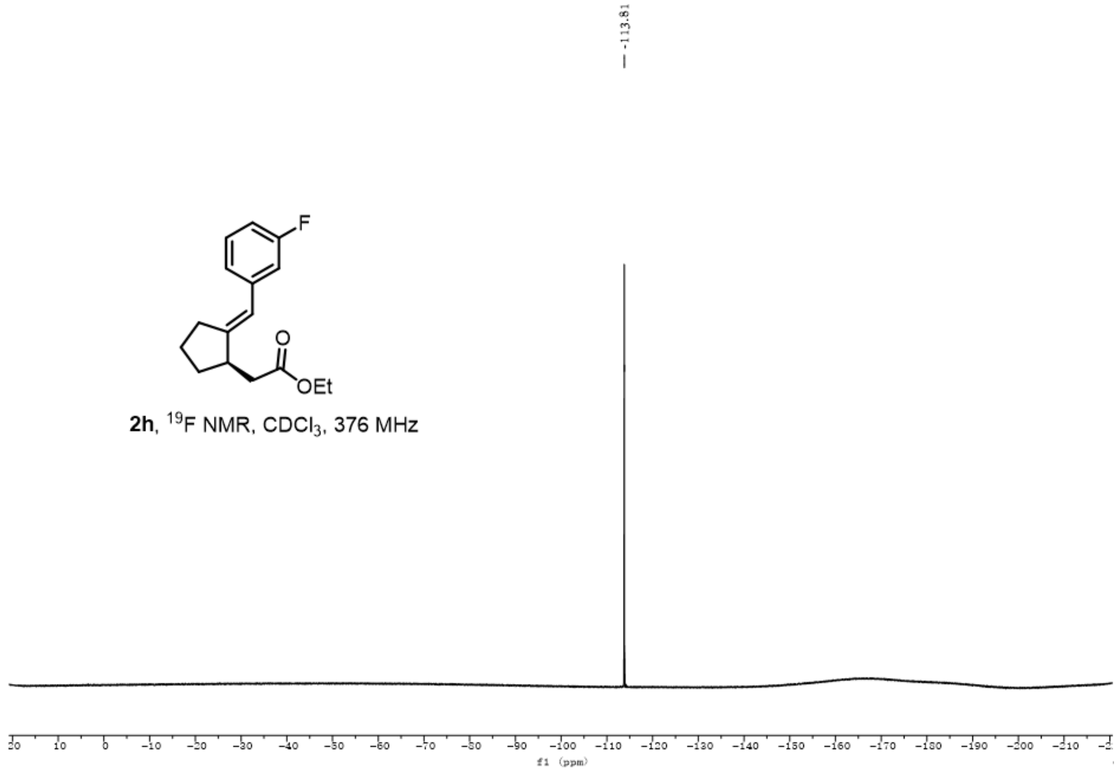

Supplementary Figure 83. ^{19}F NMR (400 MHz, $CDCl_3$, 25 °C) spectra for **2h**

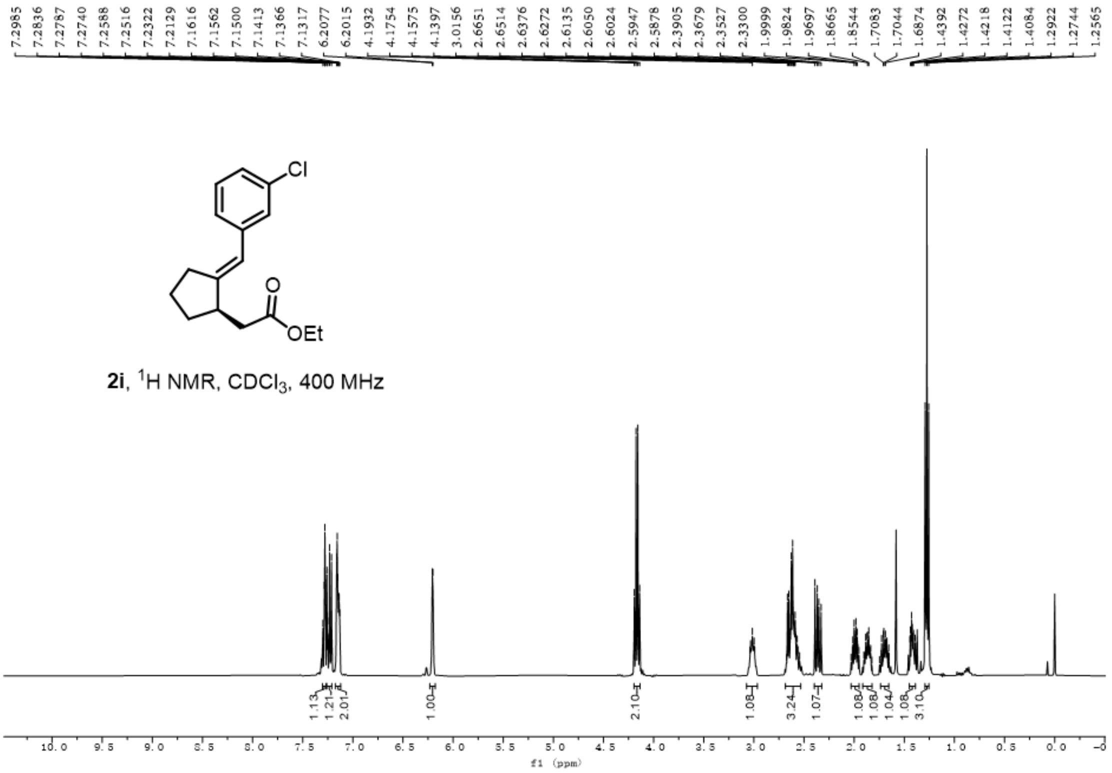

Supplementary Figure 84. ^{1}H NMR (400 MHz, $CDCl_3$, 25 °C) spectra for **2i**

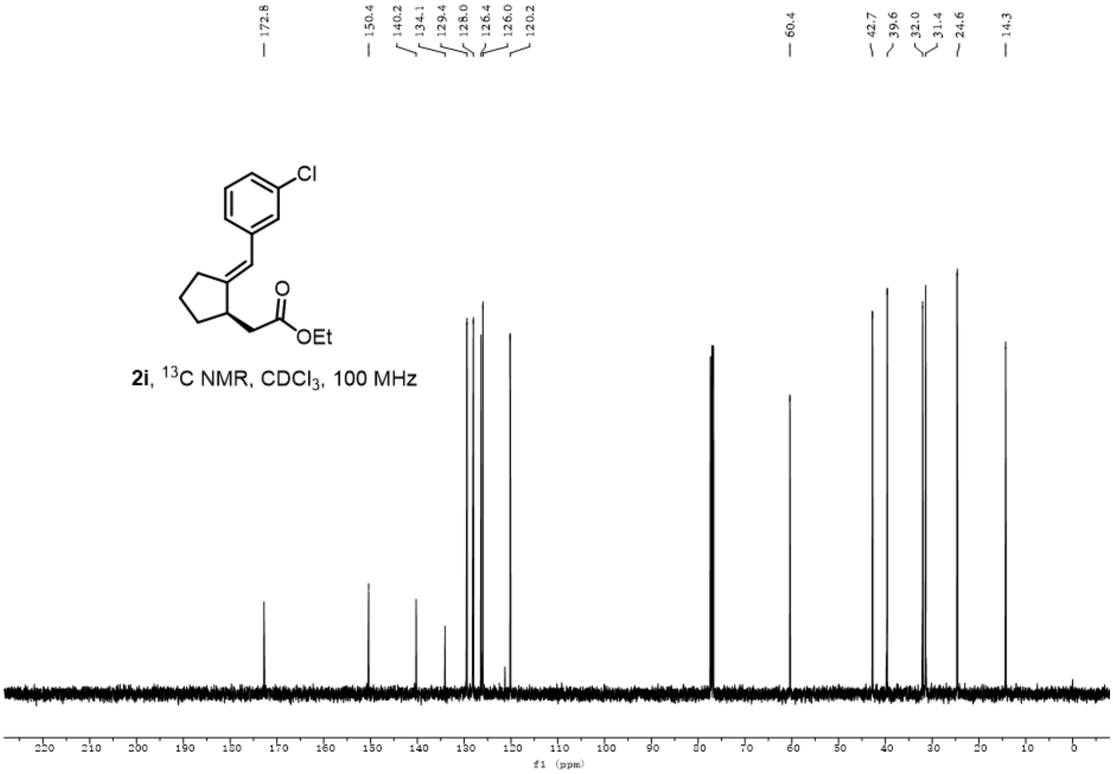

Supplementary Figure 85. ^{13}C NMR (400 MHz, $CDCl_3$, 25 °C) spectra for **2i**

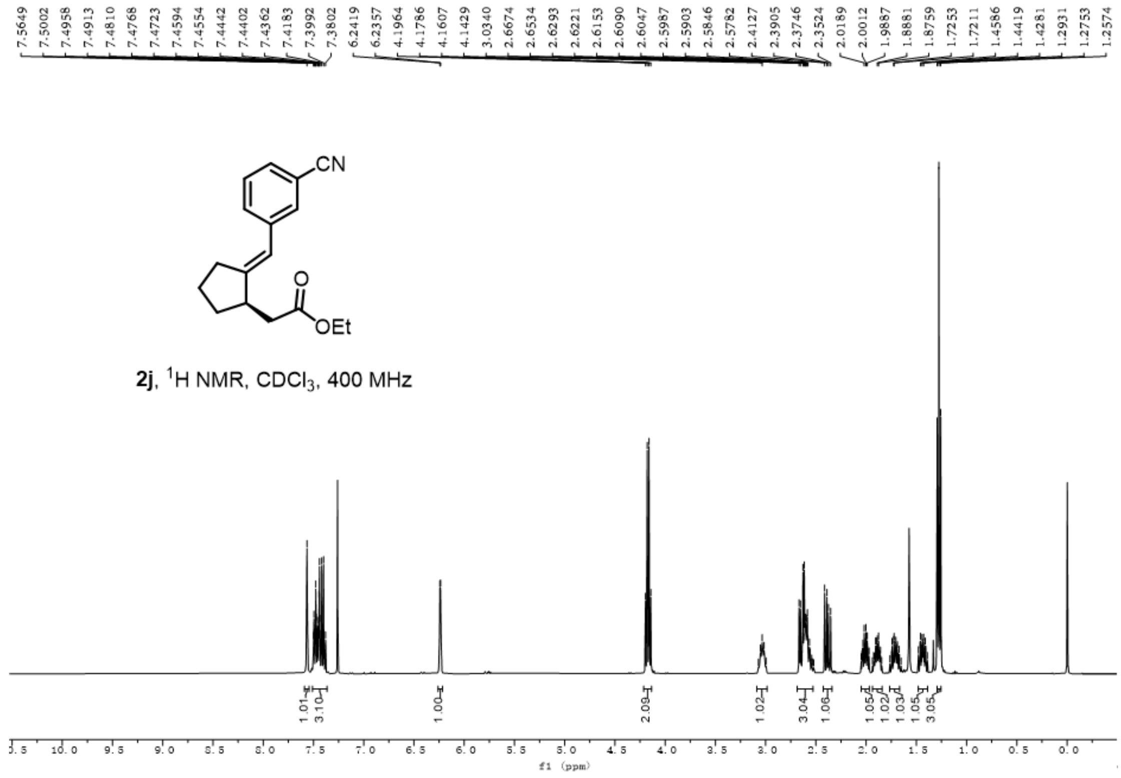

Supplementary Figure 86. ^{1}H NMR (400 MHz, $CDCl_3$, 25 °C) spectra for **2j**

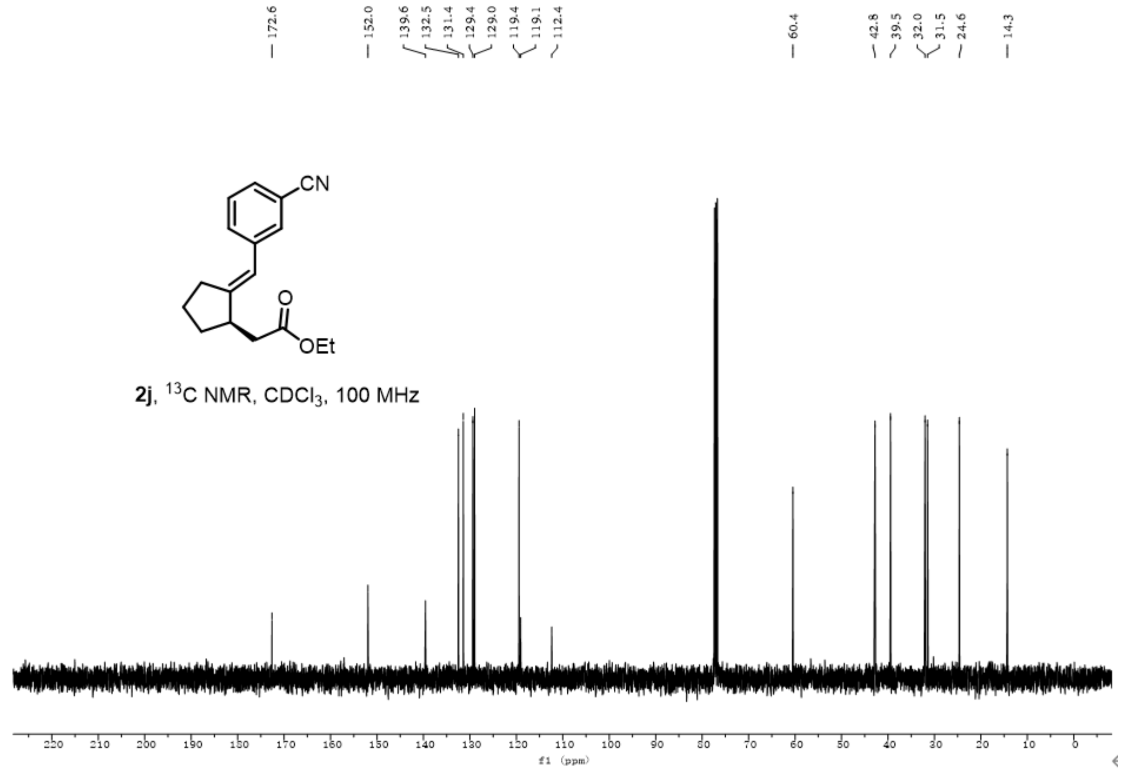

Supplementary Figure 87. ^{13}C NMR (400 MHz, $CDCl_3$, 25 °C) spectra for **2j**

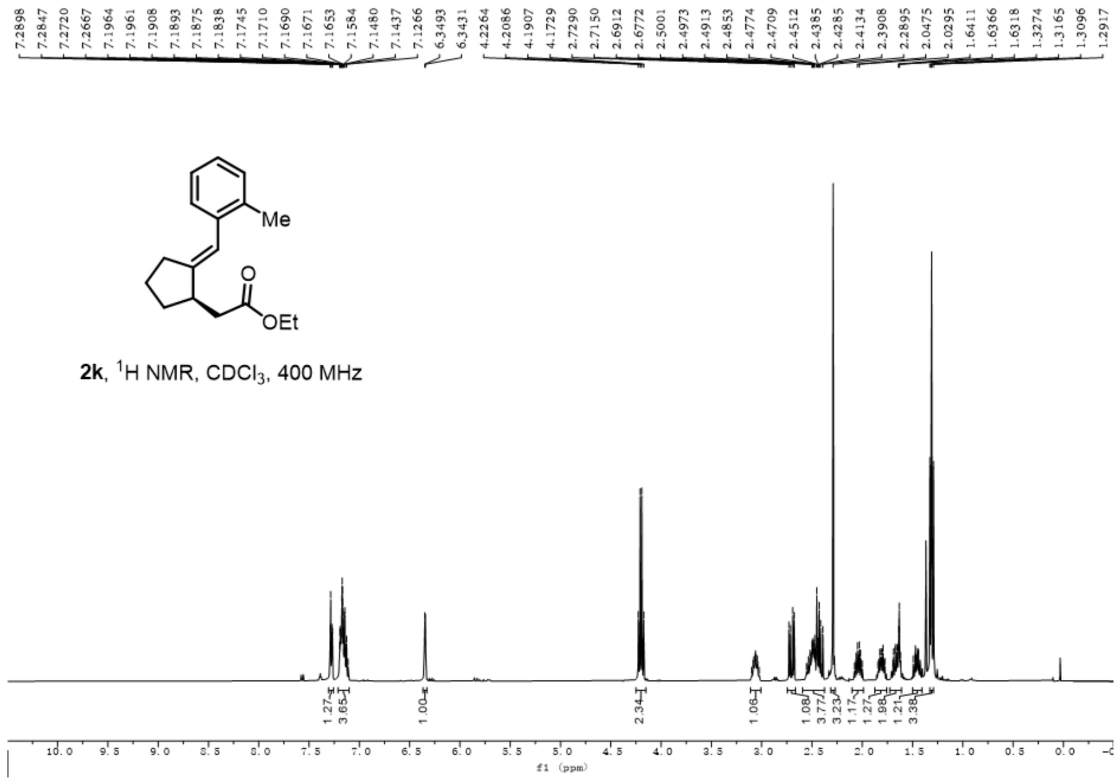

Supplementary Figure 88. ^{1}H NMR (400 MHz, $CDCl_3$, 25 °C) spectra for **2k**

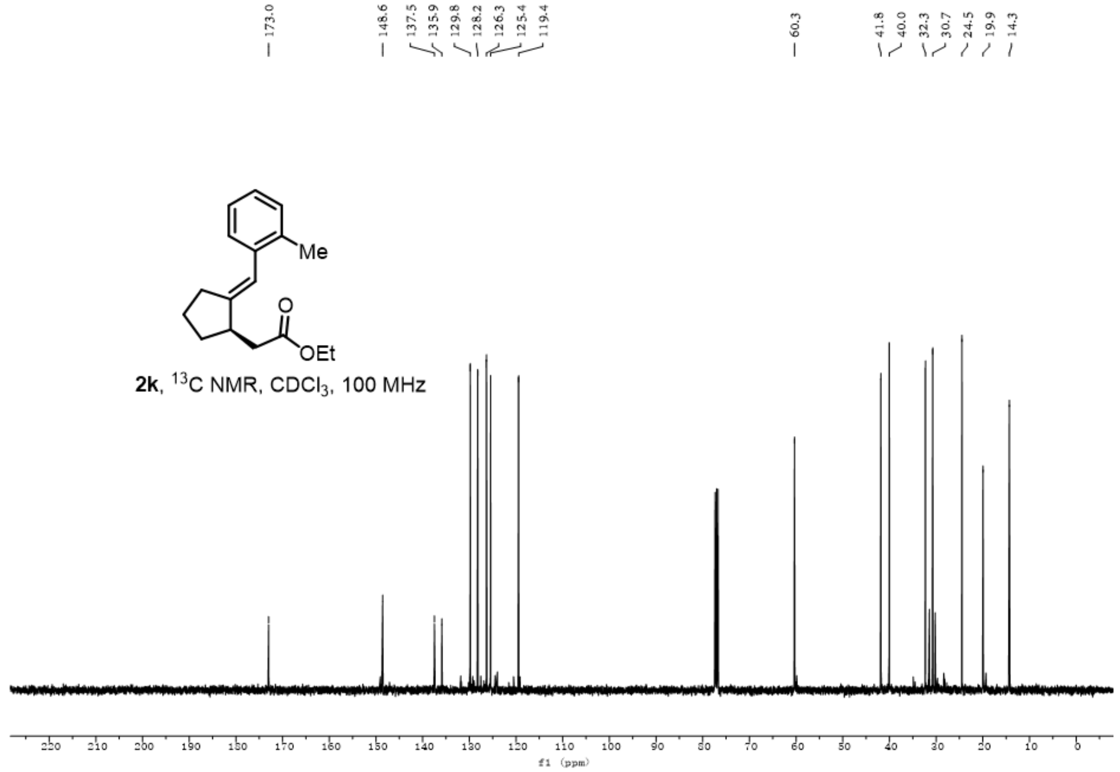

Supplementary Figure 89. ^{13}C NMR (400 MHz, $CDCl_3$, 25 °C) spectra for **2k**

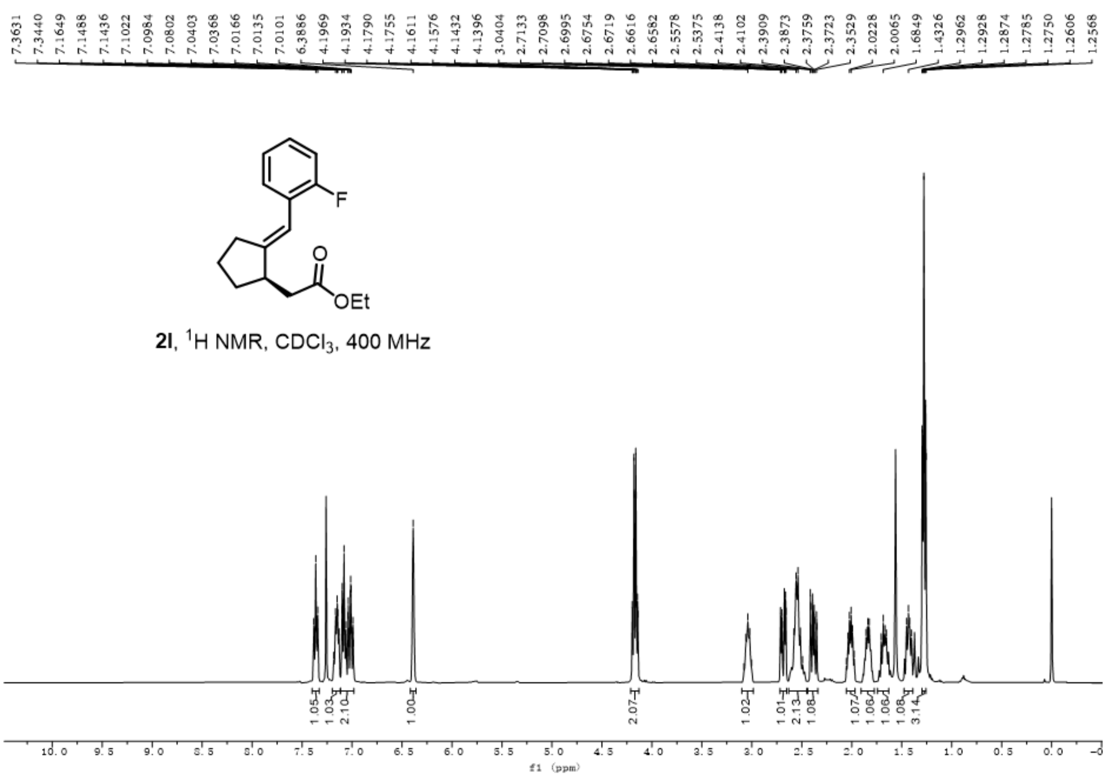

Supplementary Figure 90. ^{1}H NMR (400 MHz, $CDCl_3$, 25 °C) spectra for **2l**

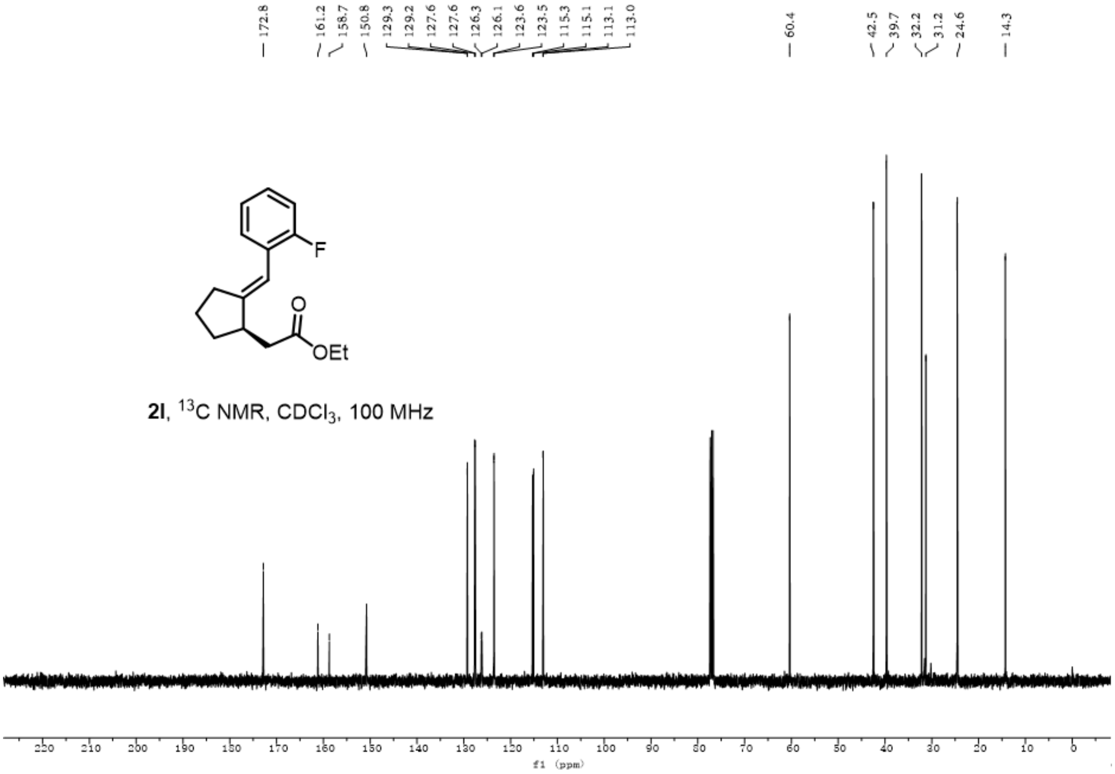

Supplementary Figure 91. ^{13}C NMR (400 MHz, $CDCl_3$, 25 °C) spectra for **2l**

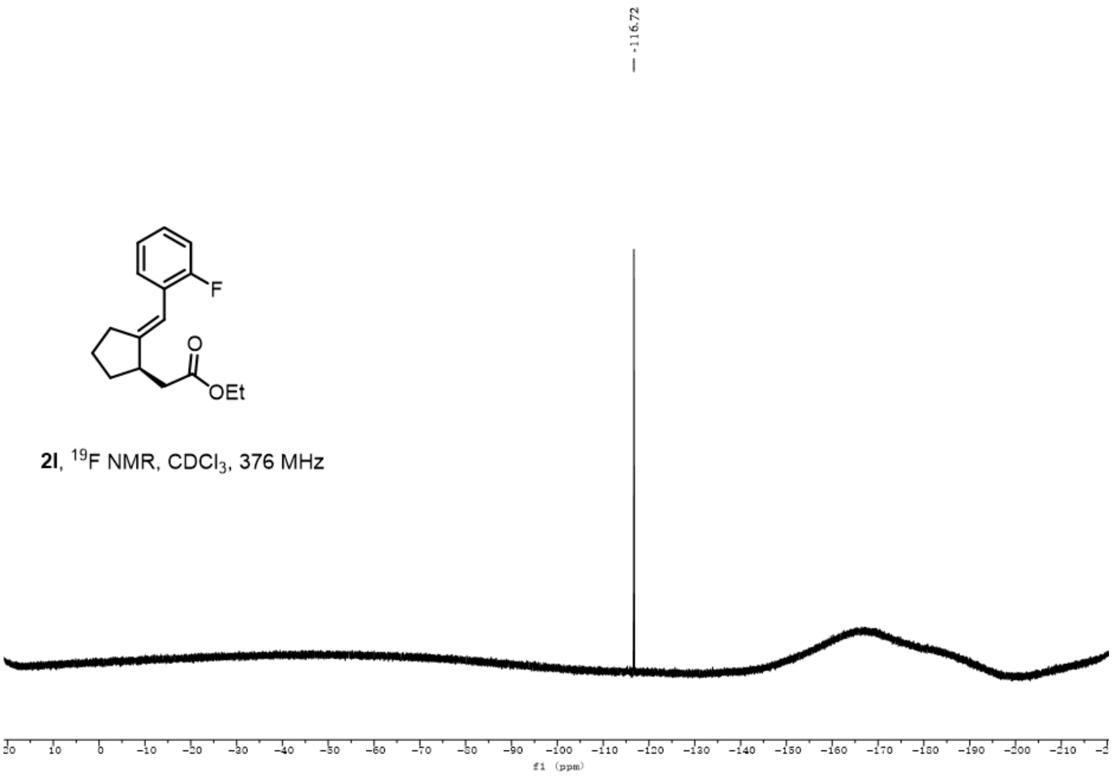

Supplementary Figure 92. ^{19}F NMR (400 MHz, $CDCl_3$, 25 °C) spectra for **2l**

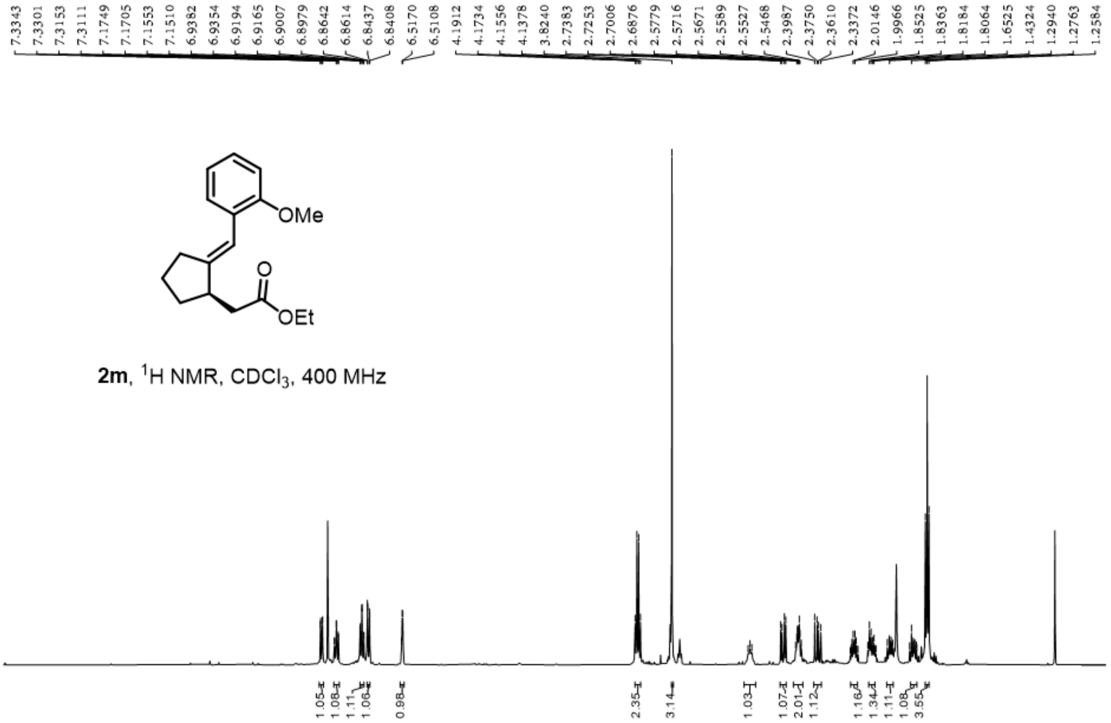

Supplementary Figure 93. ^{1}H NMR (400 MHz, $CDCl_3$, 25 °C) spectra for **2m**

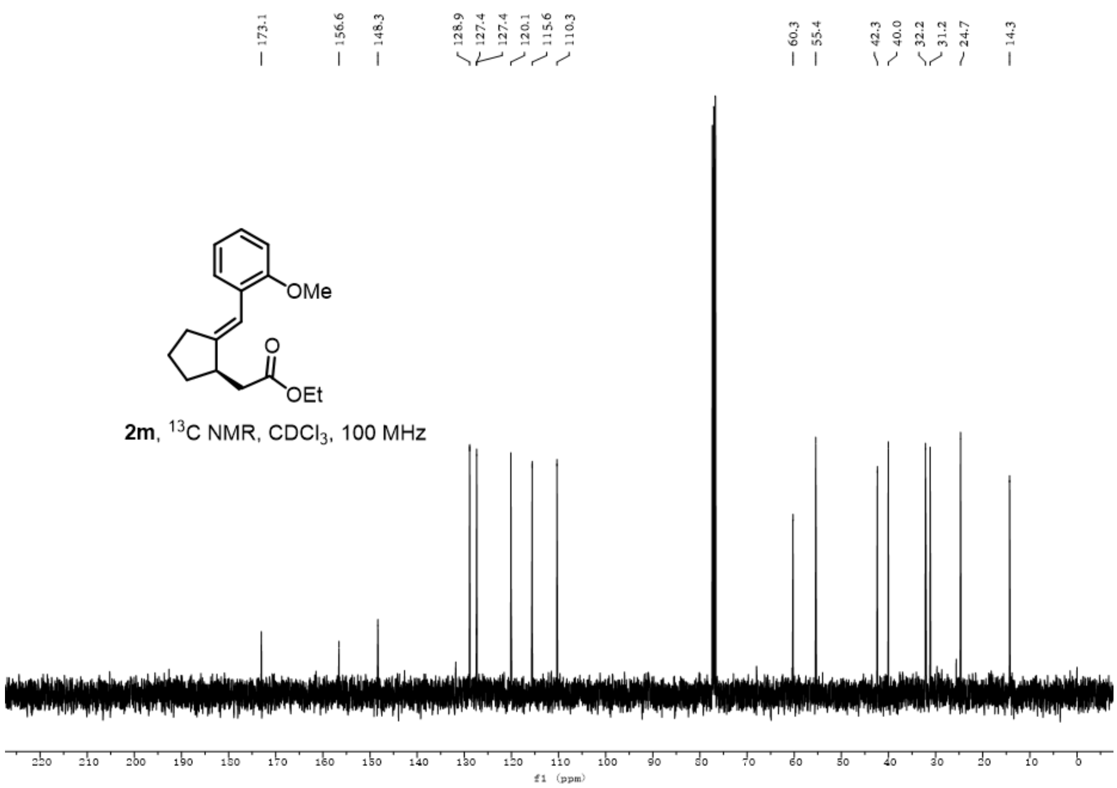

Supplementary Figure 94. ^{13}C NMR (400 MHz, $CDCl_3$, 25 °C) spectra for **2m**

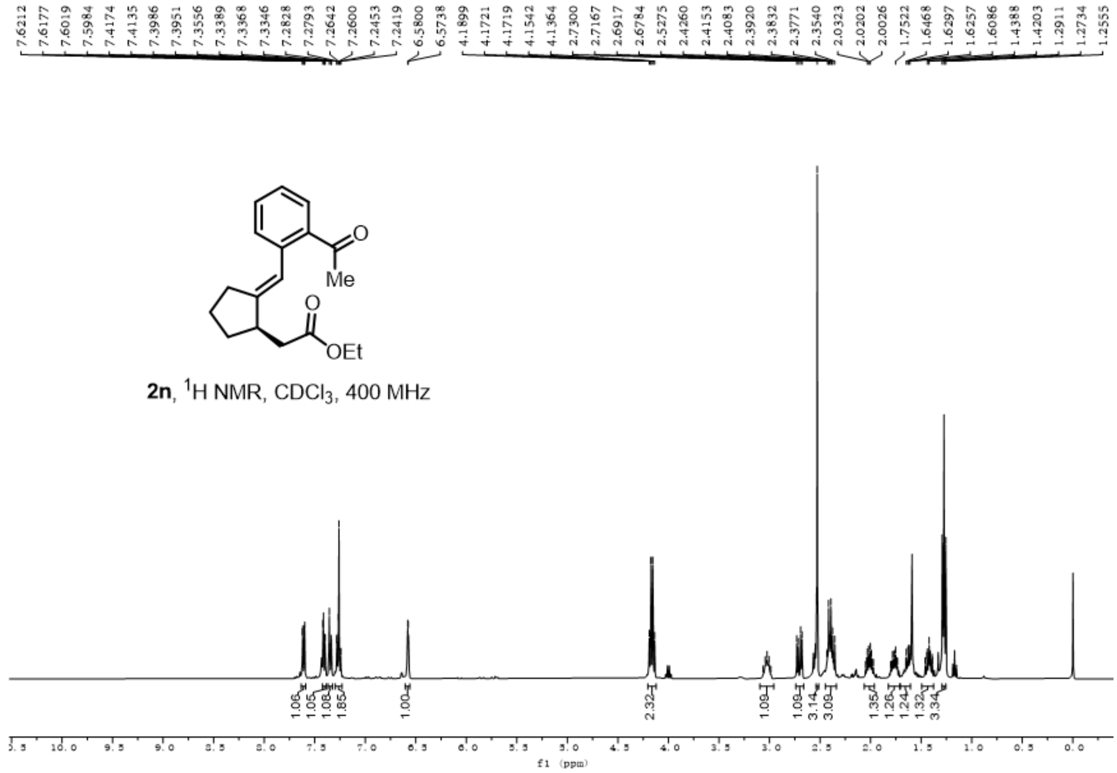

Supplementary Figure 95. ^{1}H NMR (400 MHz, $CDCl_3$, 25 °C) spectra for **2n**

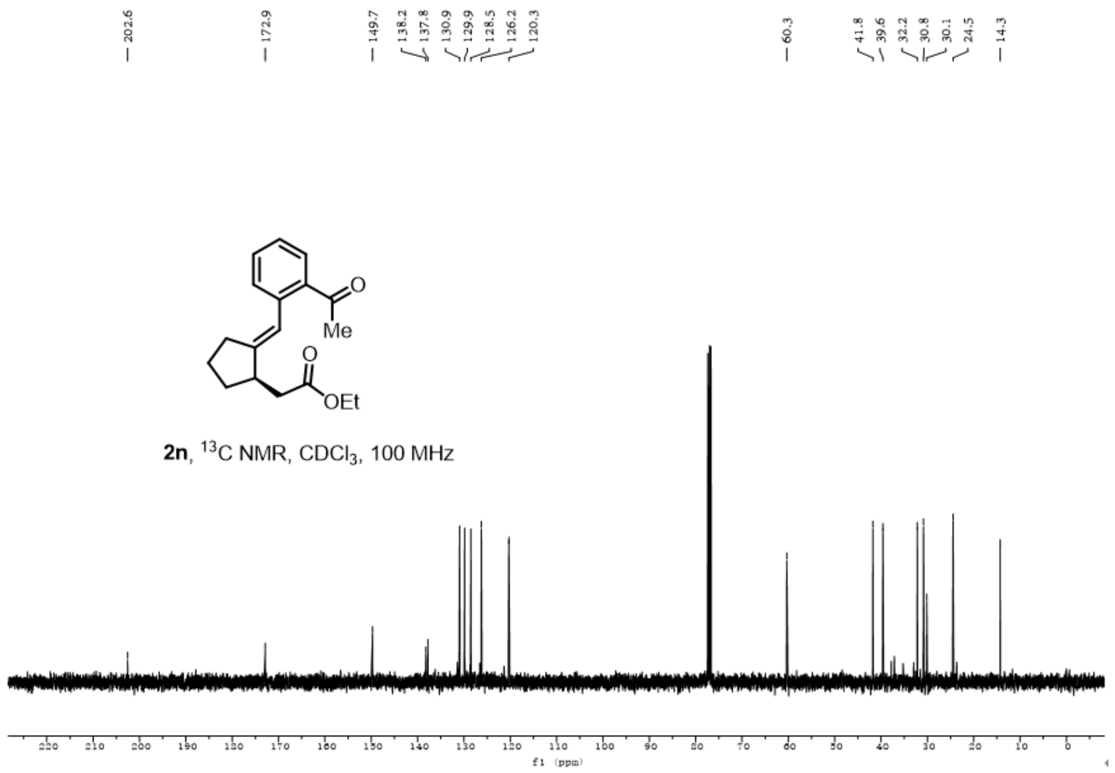

Supplementary Figure 96. ^{13}C NMR (400 MHz, $CDCl_3$, 25 °C) spectra for **2n**

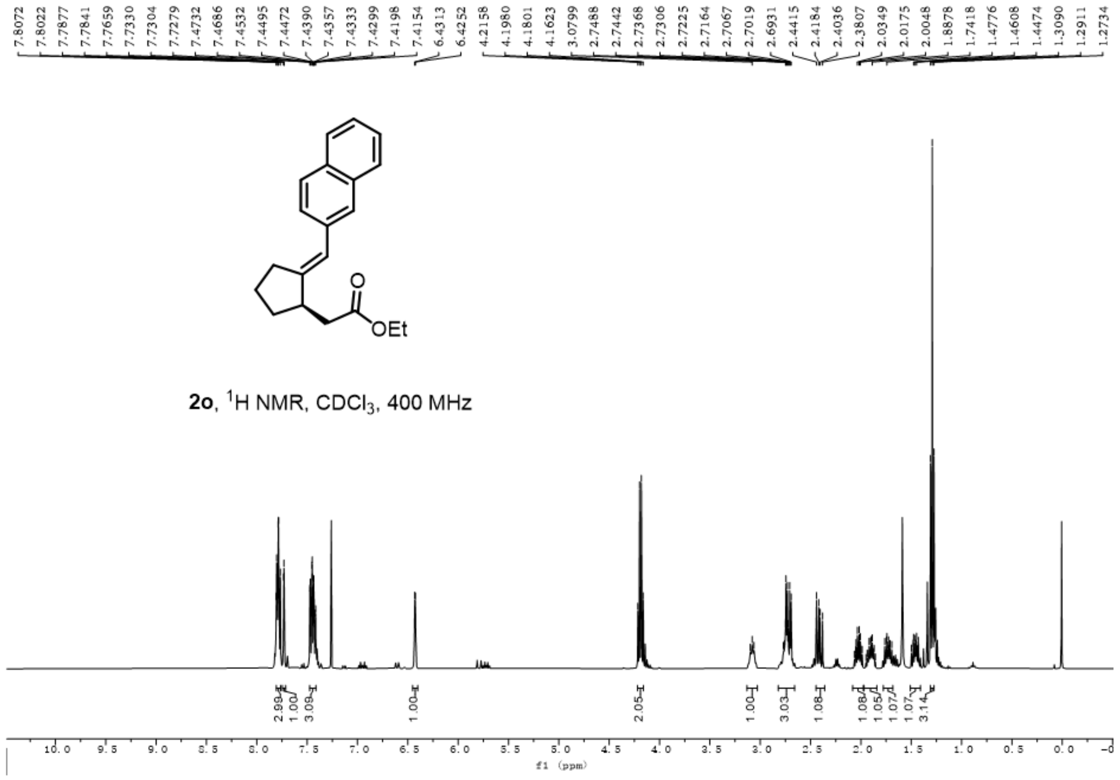

Supplementary Figure 97. ^{1}H NMR (400 MHz, $CDCl_3$, 25 °C) spectra for **2o**

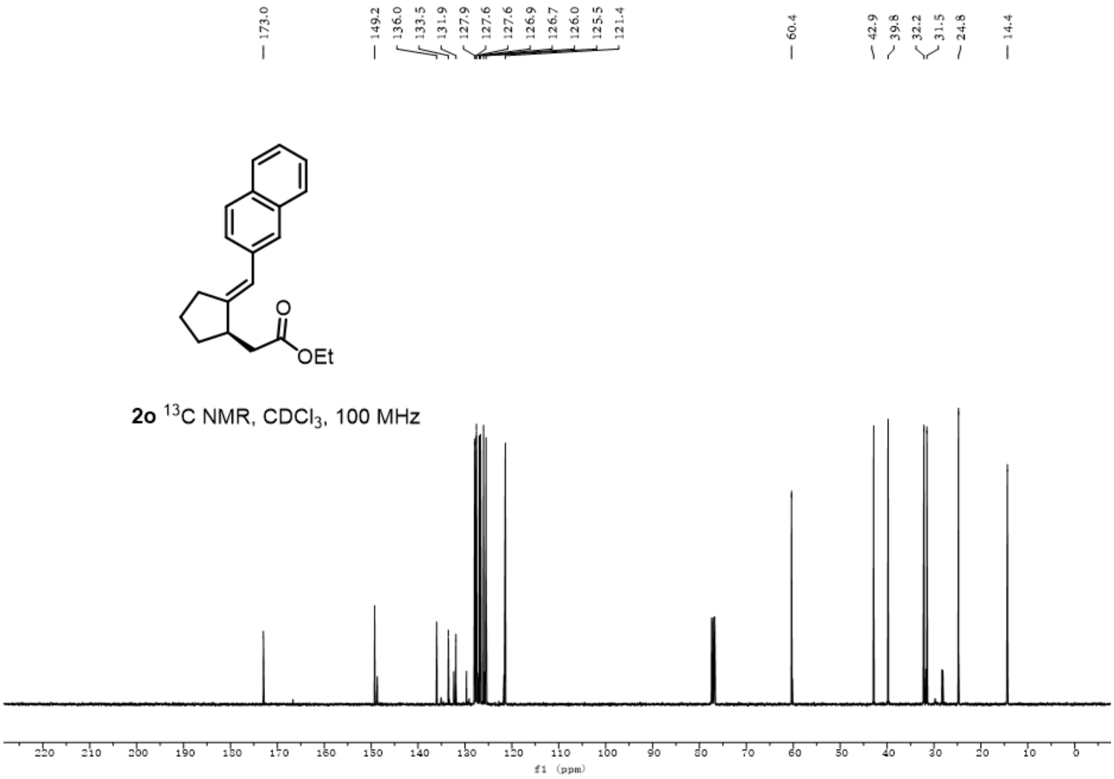

Supplementary Figure 98. ^{13}C NMR (400 MHz, $CDCl_3$, 25 °C) spectra for **2o**

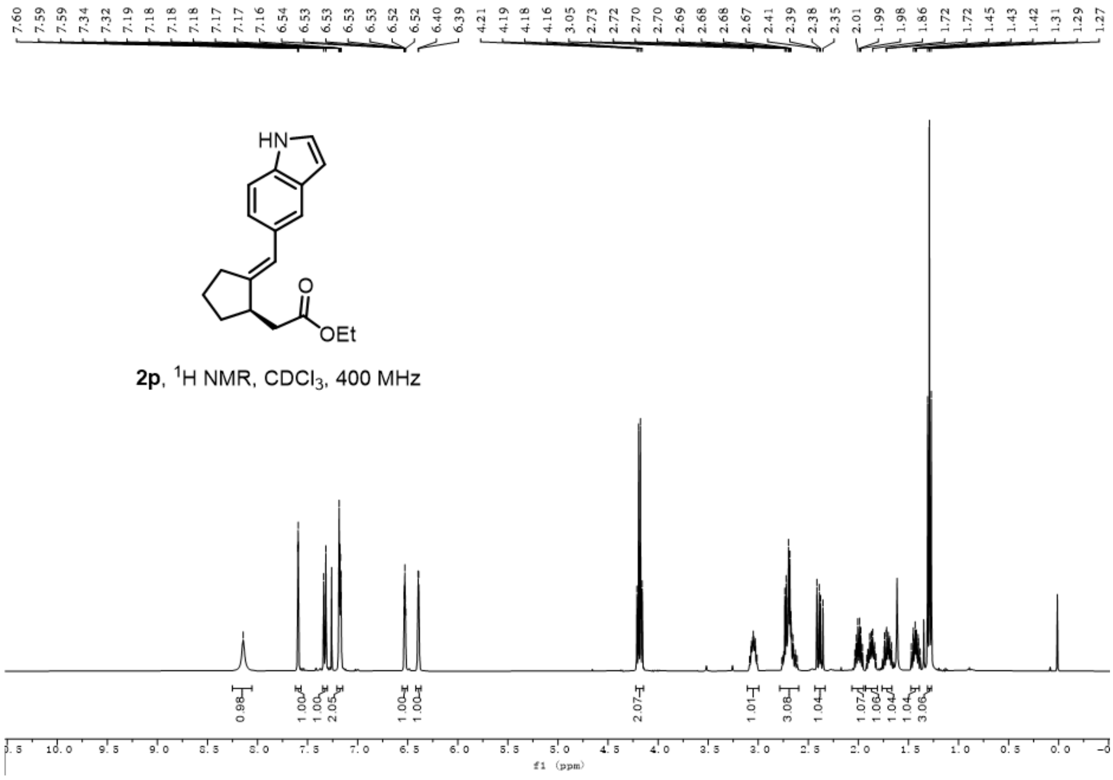

Supplementary Figure 99. ^{1}H NMR (400 MHz, $CDCl_3$, 25 °C) spectra for **2p**

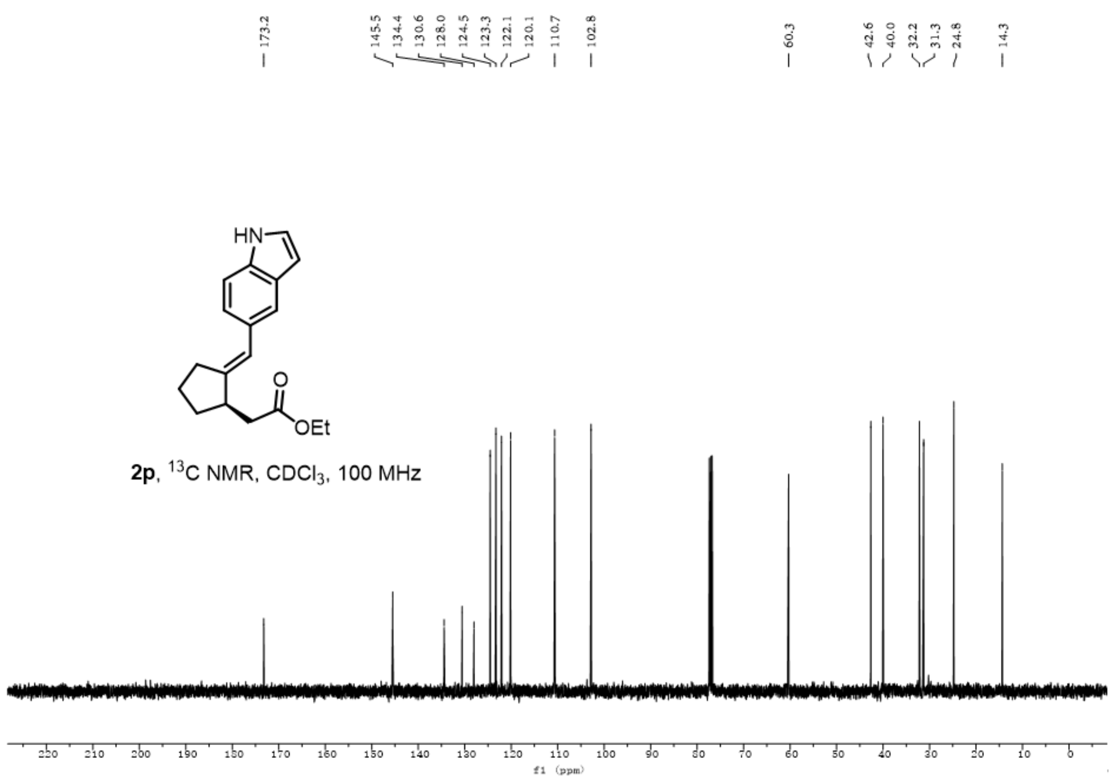

Supplementary Figure 100. ^{13}C NMR (400 MHz, $CDCl_3$, 25 °C) spectra for **2p**

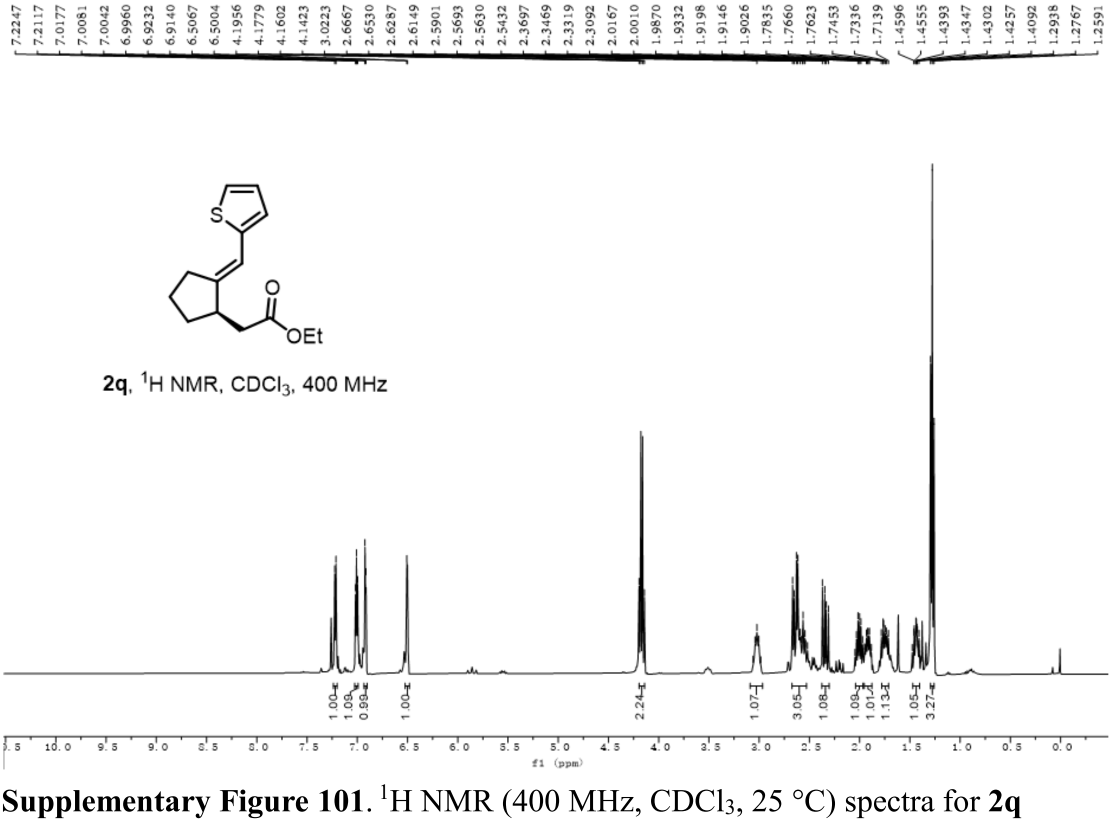

Supplementary Figure 101. ^{1}H NMR (400 MHz, $CDCl_3$, 25 °C) spectra for **2q**

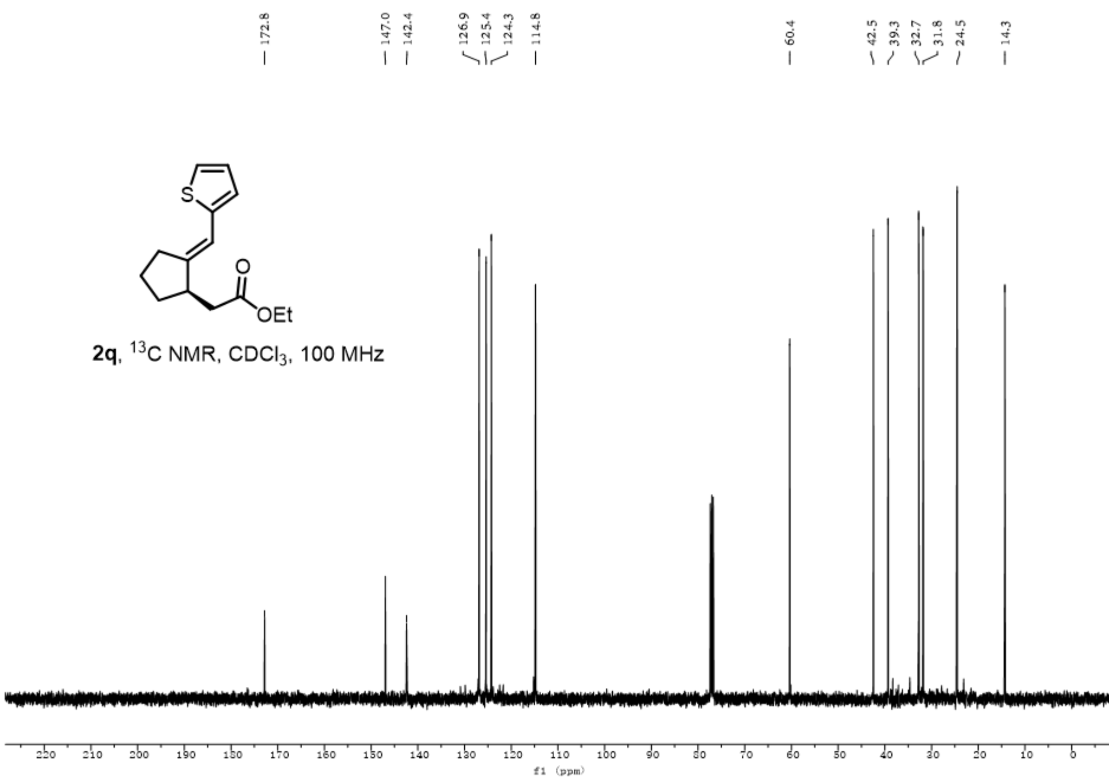

Supplementary Figure 102. ^{13}C NMR (400 MHz, $CDCl_3$, 25 °C) spectra for **2q**

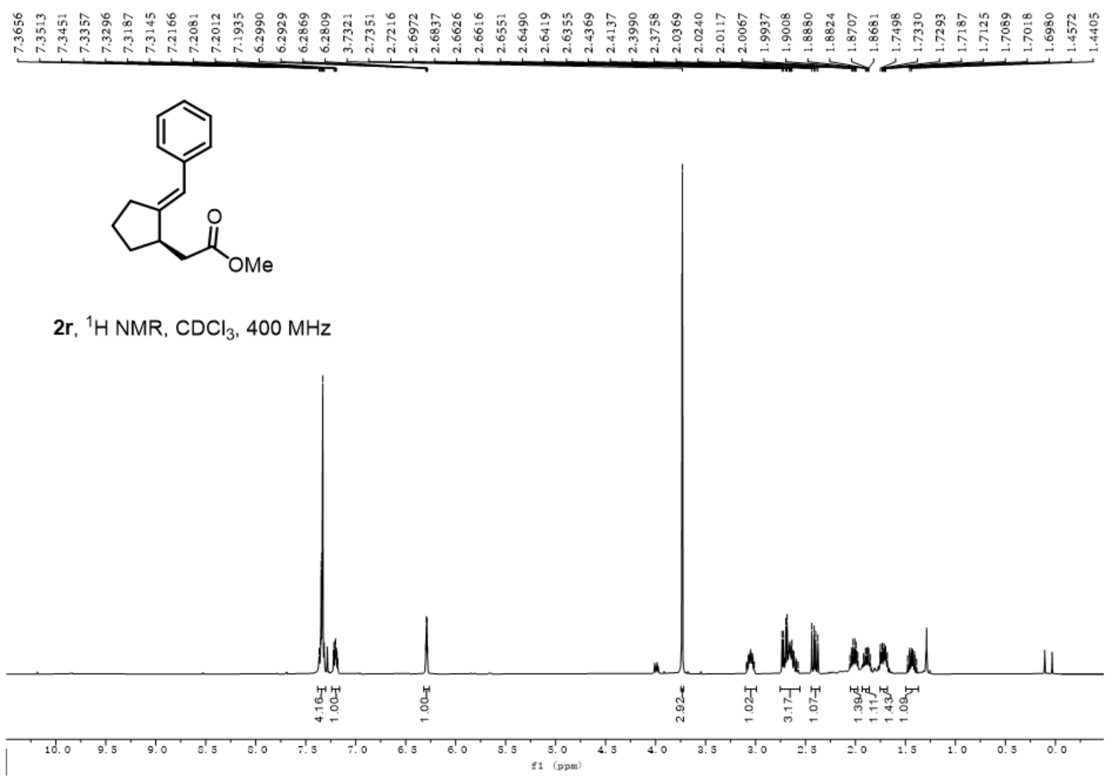

Supplementary Figure 103. ^{1}H NMR (400 MHz, $CDCl_3$, 25 °C) spectra for **2r**

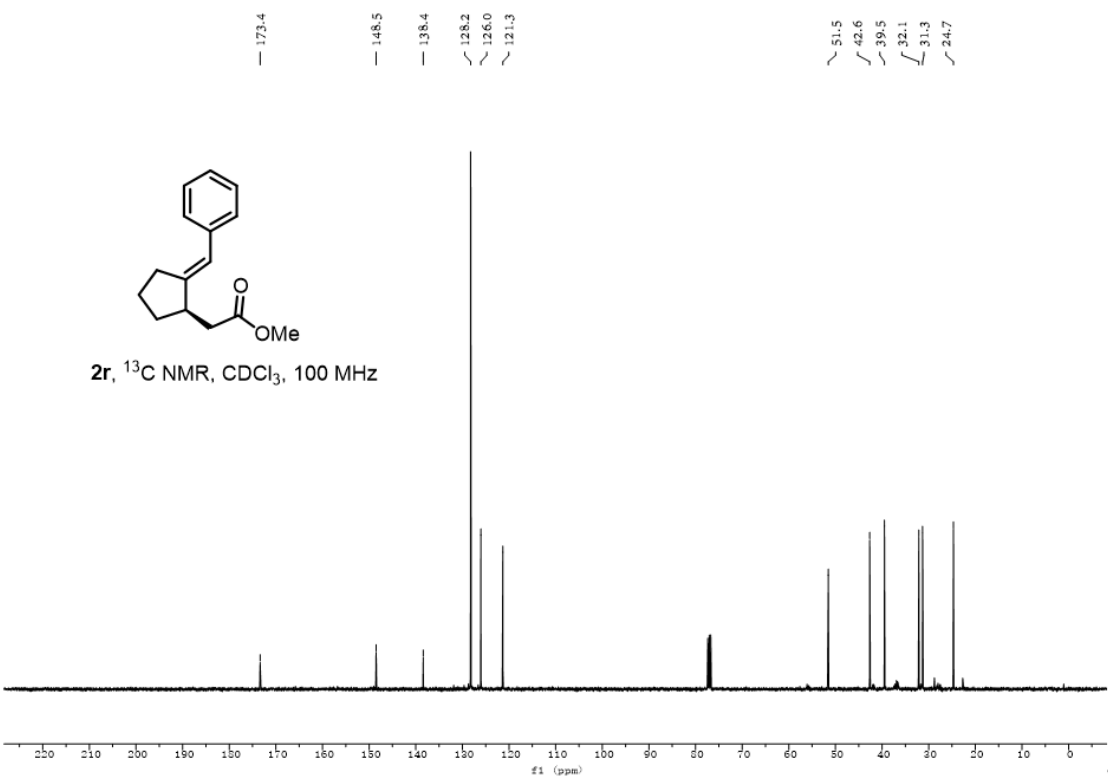

Supplementary Figure 104. ^{13}C NMR (400 MHz, $CDCl_3$, 25 °C) spectra for **2r**

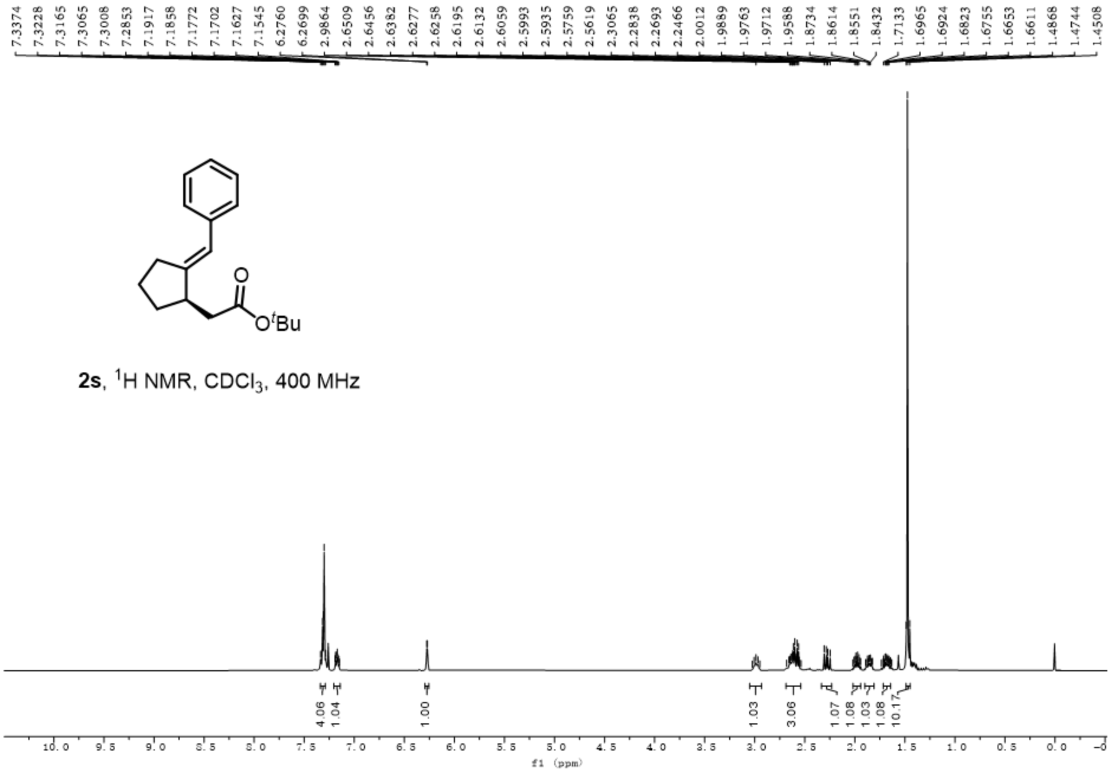

Supplementary Figure 105. ^{1}H NMR (400 MHz, $CDCl_3$, 25 °C) spectra for **2s**

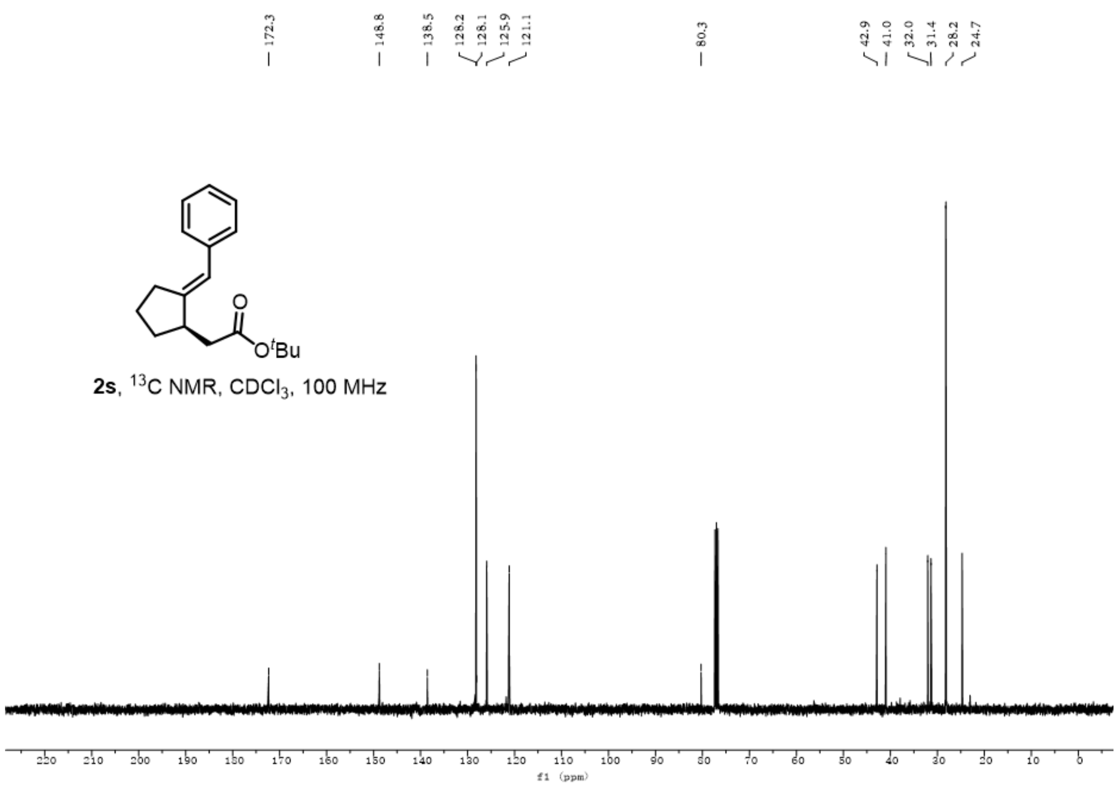

Supplementary Figure 106. ^{13}C NMR (400 MHz, $CDCl_3$, 25 °C) spectra for **2s**

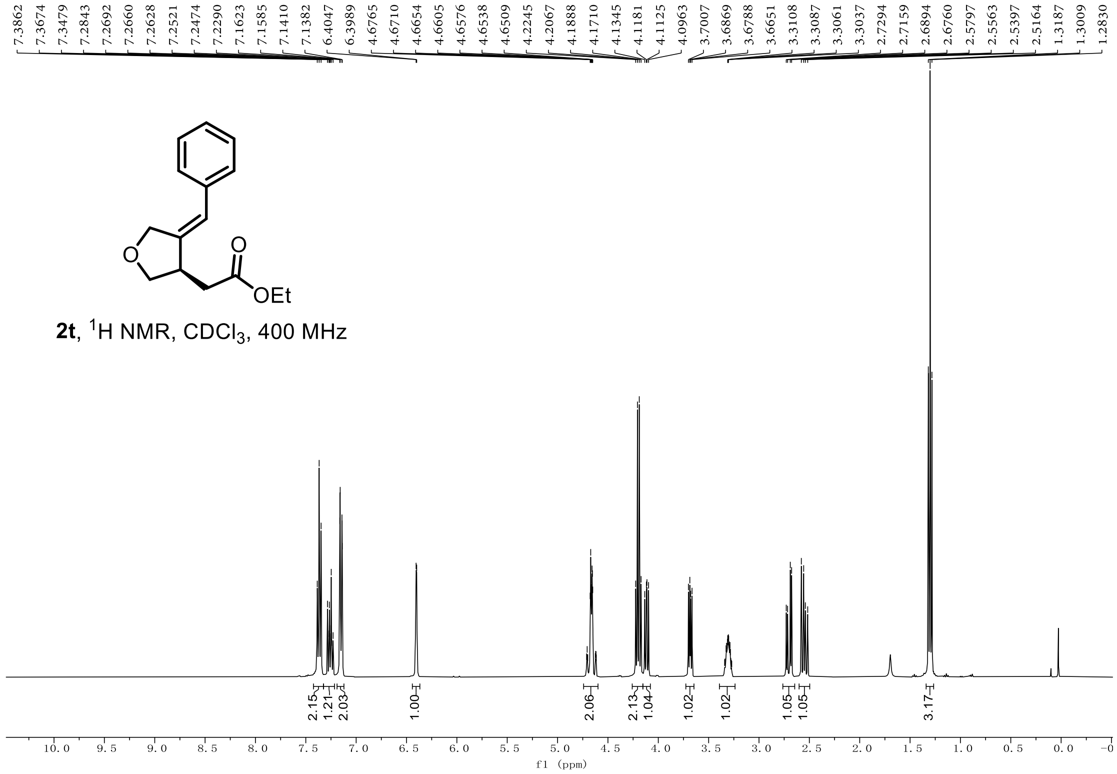

Supplementary Figure 107. ^{1}H NMR (400 MHz, $CDCl_3$, 25 °C) spectra for **2t**

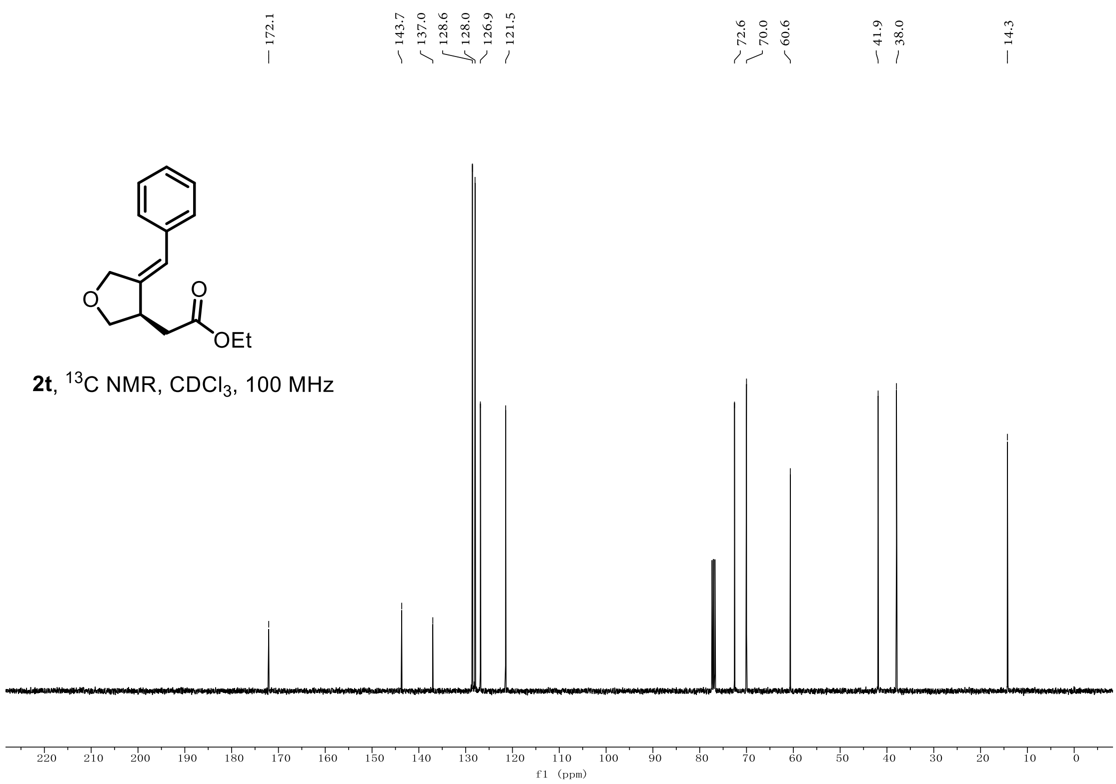

Supplementary Figure 108. ^{13}C NMR (400 MHz, $CDCl_3$, 25 °C) spectra for **2t**

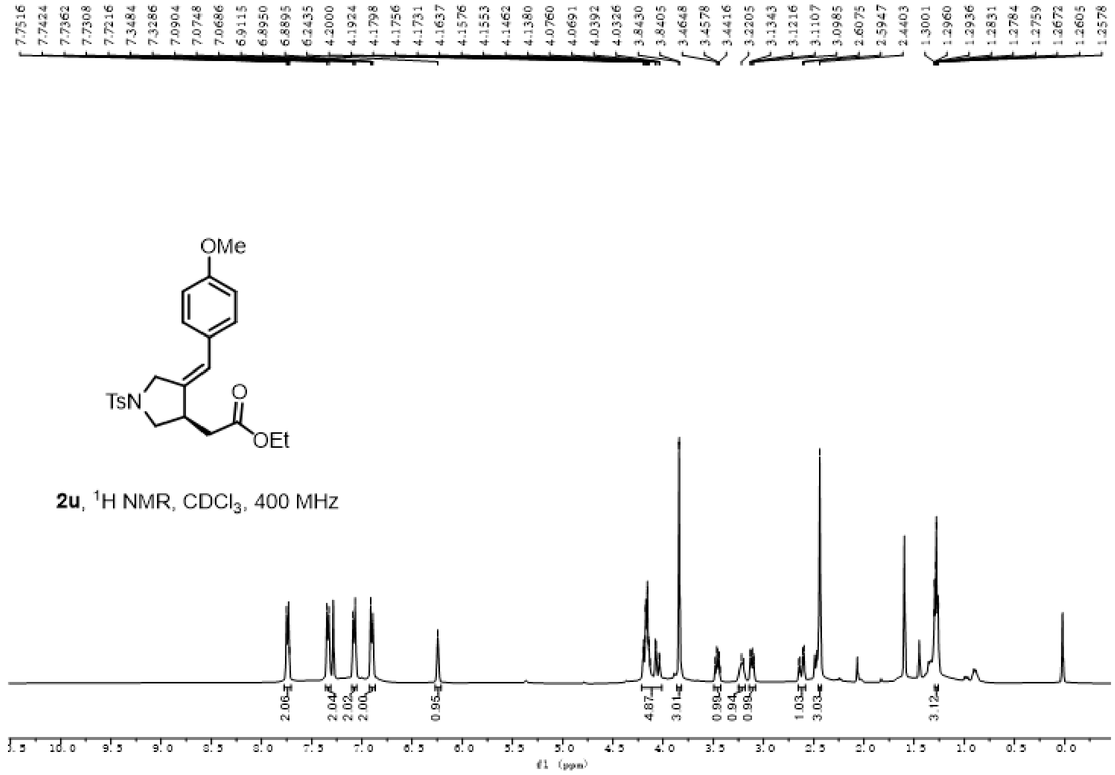

Supplementary Figure 109. ^{1}H NMR (400 MHz, $CDCl_3$) spectra for **2u**

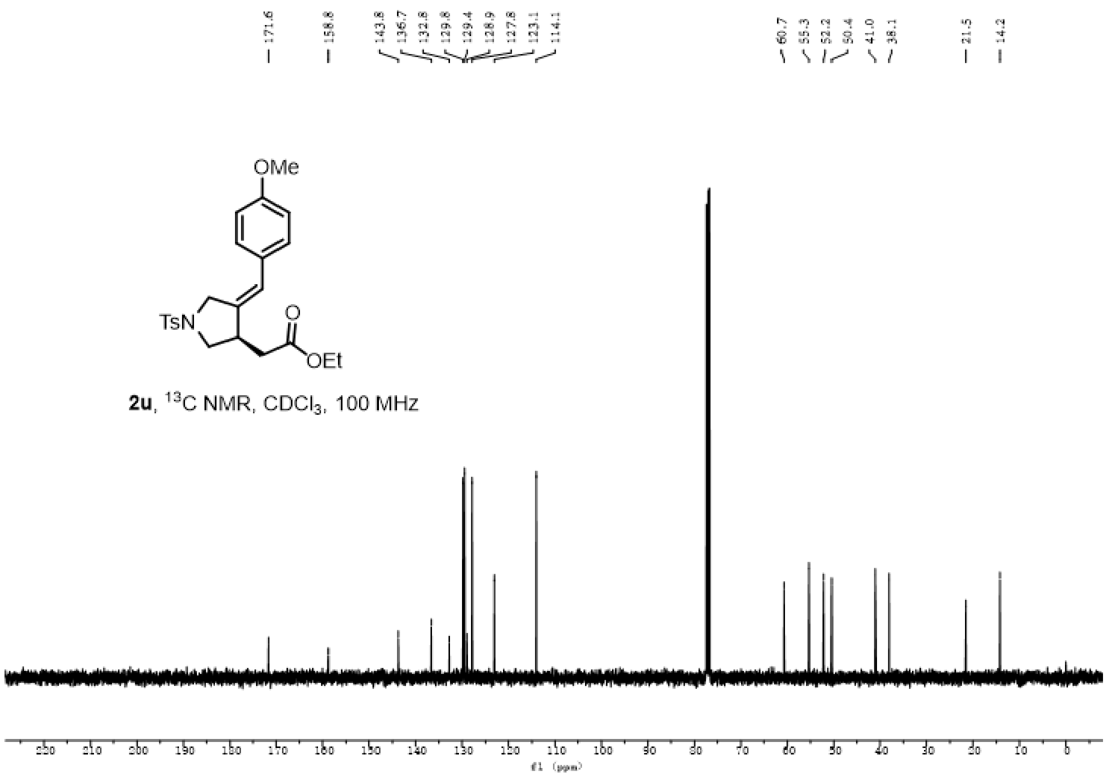

Supplementary Figure 110. ^{13}C NMR (400 MHz, $CDCl_3$, 25 °C) spectra for **2u**

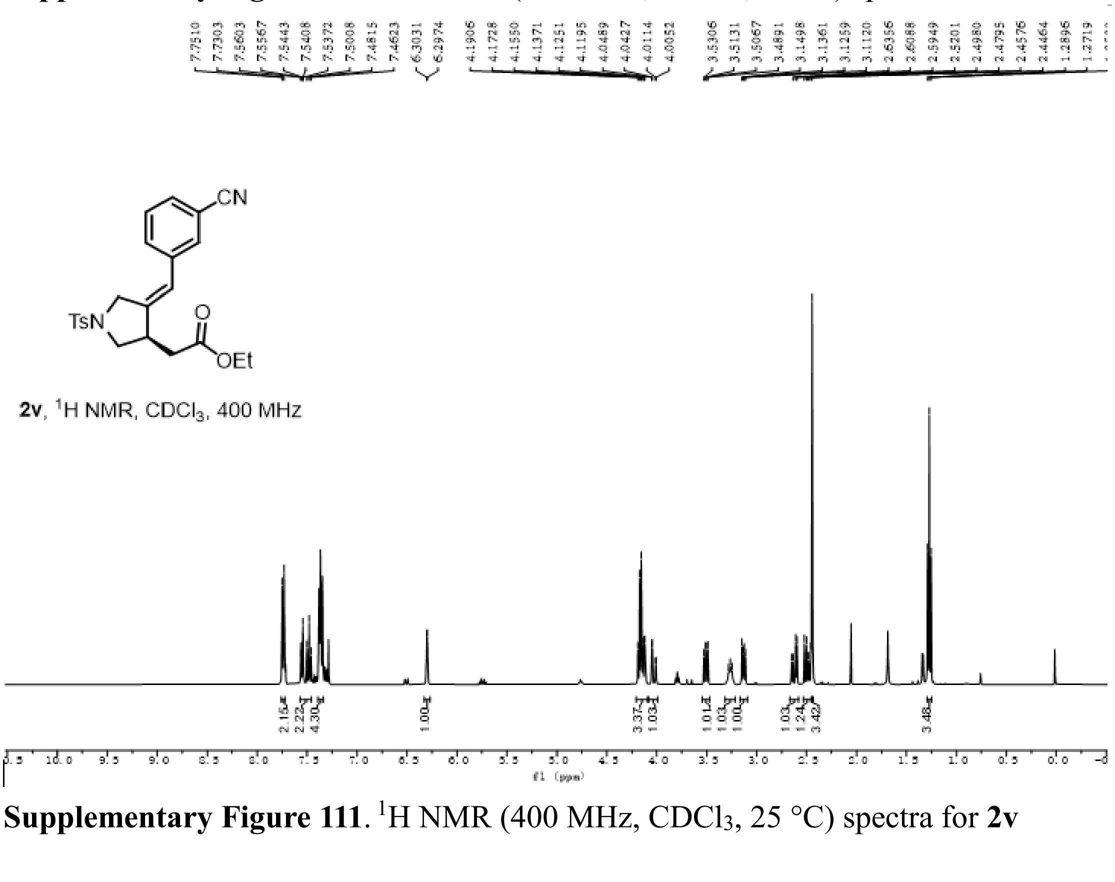

Supplementary Figure 111. ^{1}H NMR (400 MHz, $CDCl_3$, 25 °C) spectra for **2v**

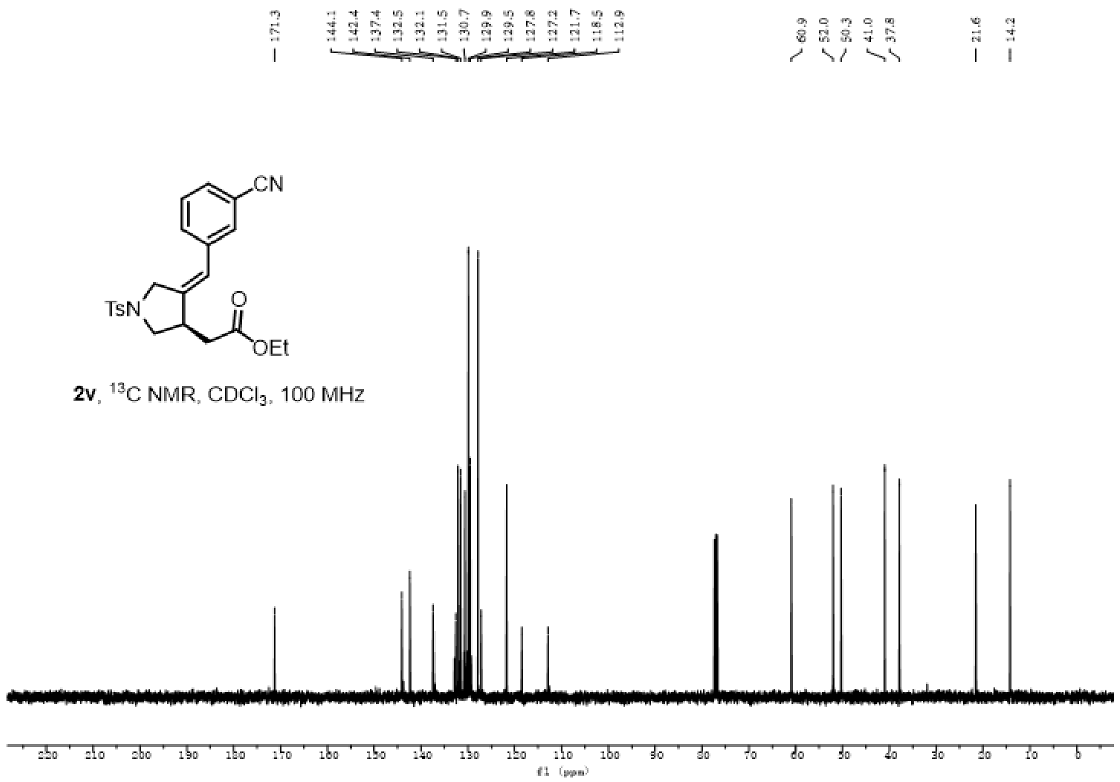

Supplementary Figure 112. ^{13}C NMR (400 MHz, $CDCl_3$, 25 °C) spectra for **2v**

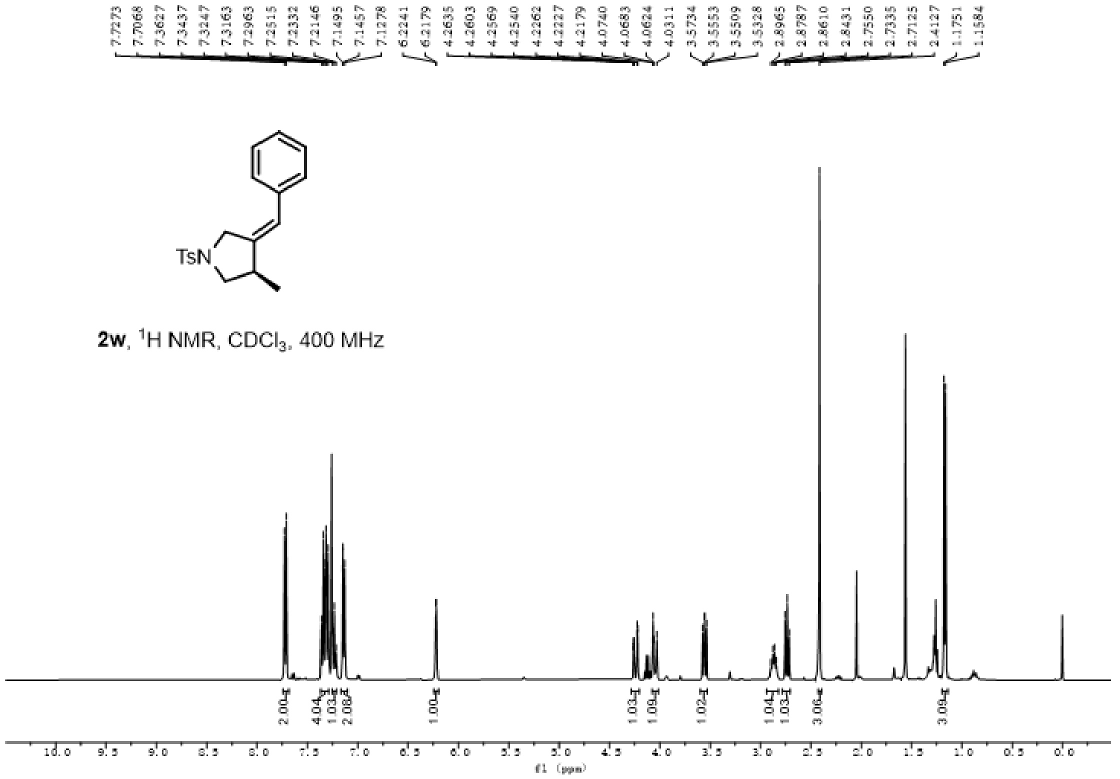

Supplementary Figure 113. ^{1}H NMR (400 MHz, $CDCl_3$, 25 °C) spectra for **2w**

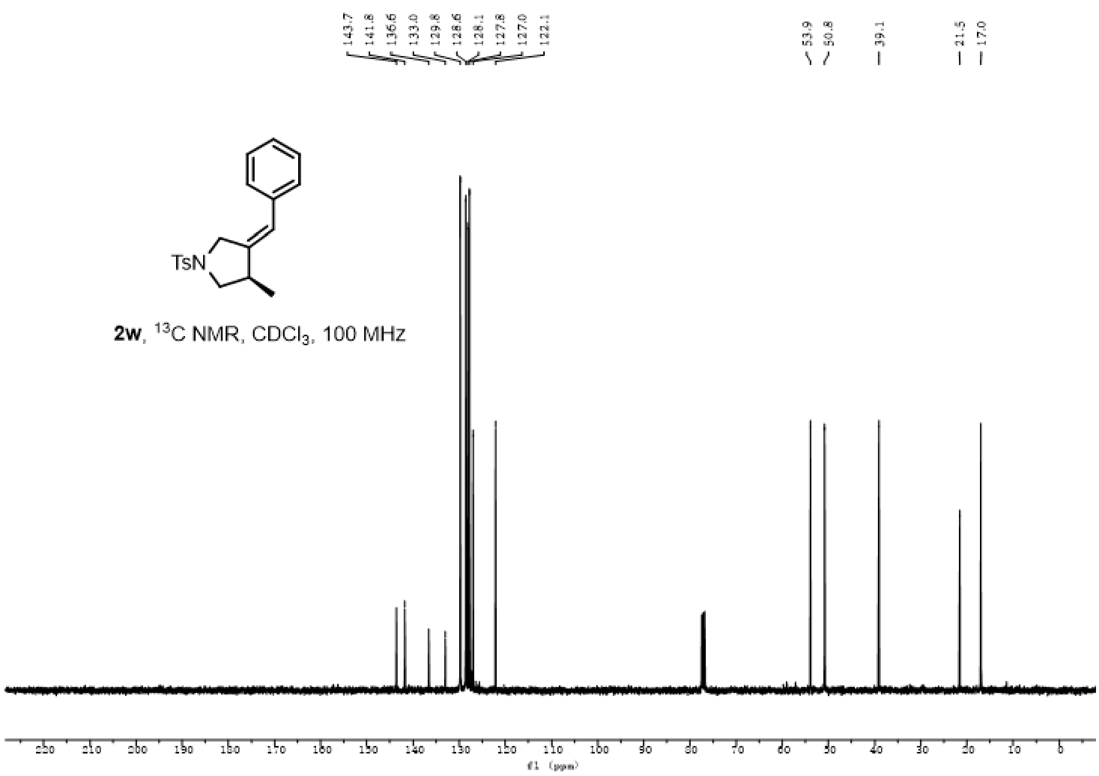

Supplementary Figure 114. ^{13}C NMR (400 MHz, $CDCl_3$, 25 °C) spectra for **2w**

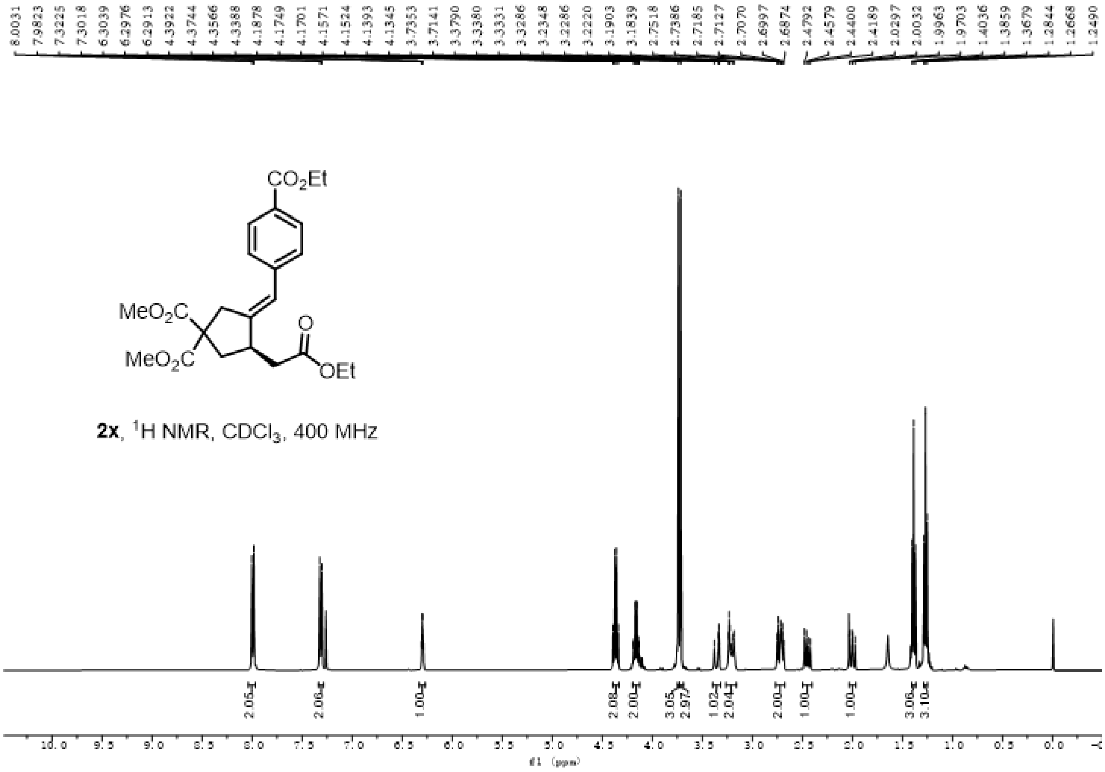

Supplementary Figure 115. ^{1}H NMR (400 MHz, $CDCl_3$, 25 °C) spectra for **2x**

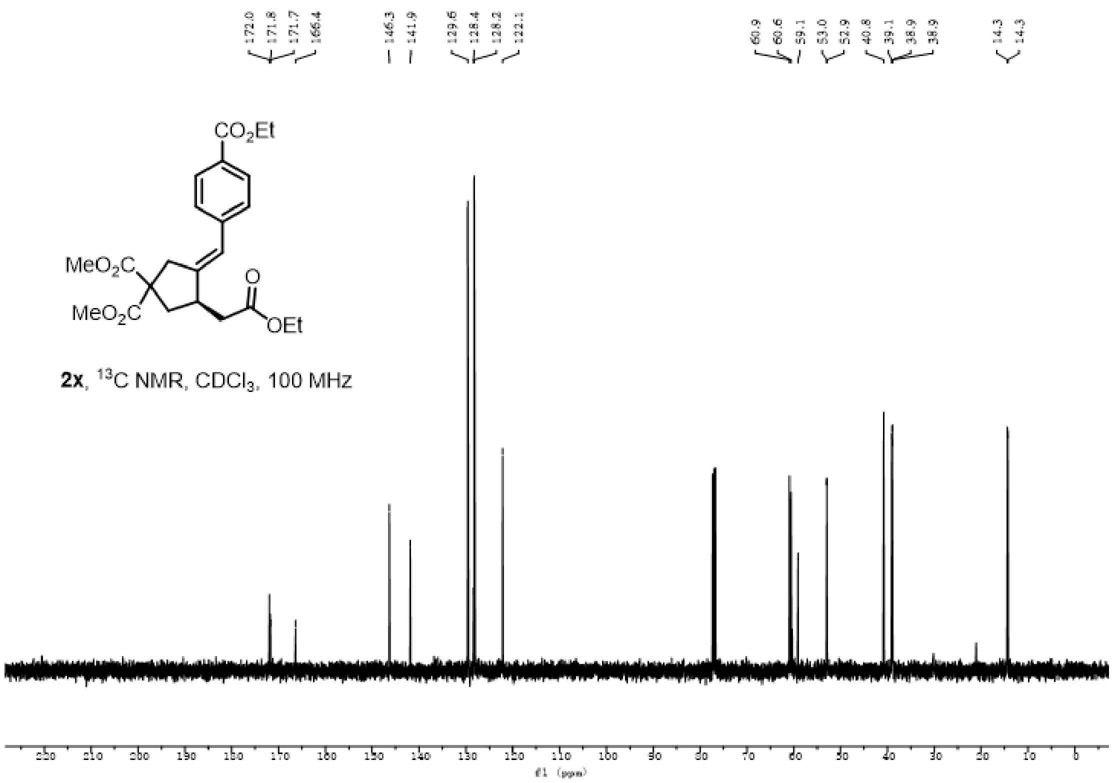

Supplementary Figure 116. ^{13}C NMR (400 MHz, $CDCl_3$, 25 °C) spectra for **2x**

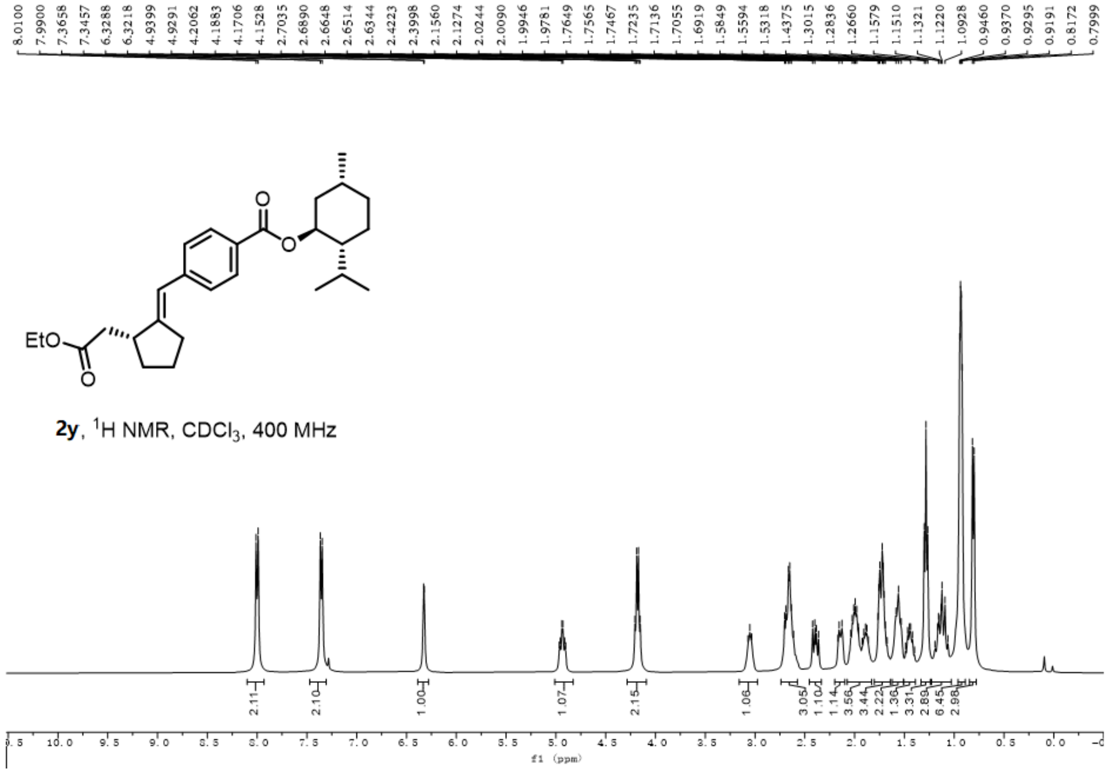

Supplementary Figure 117. ^{1}H NMR (400 MHz, $CDCl_3$, 25 °C) spectra for **2y**

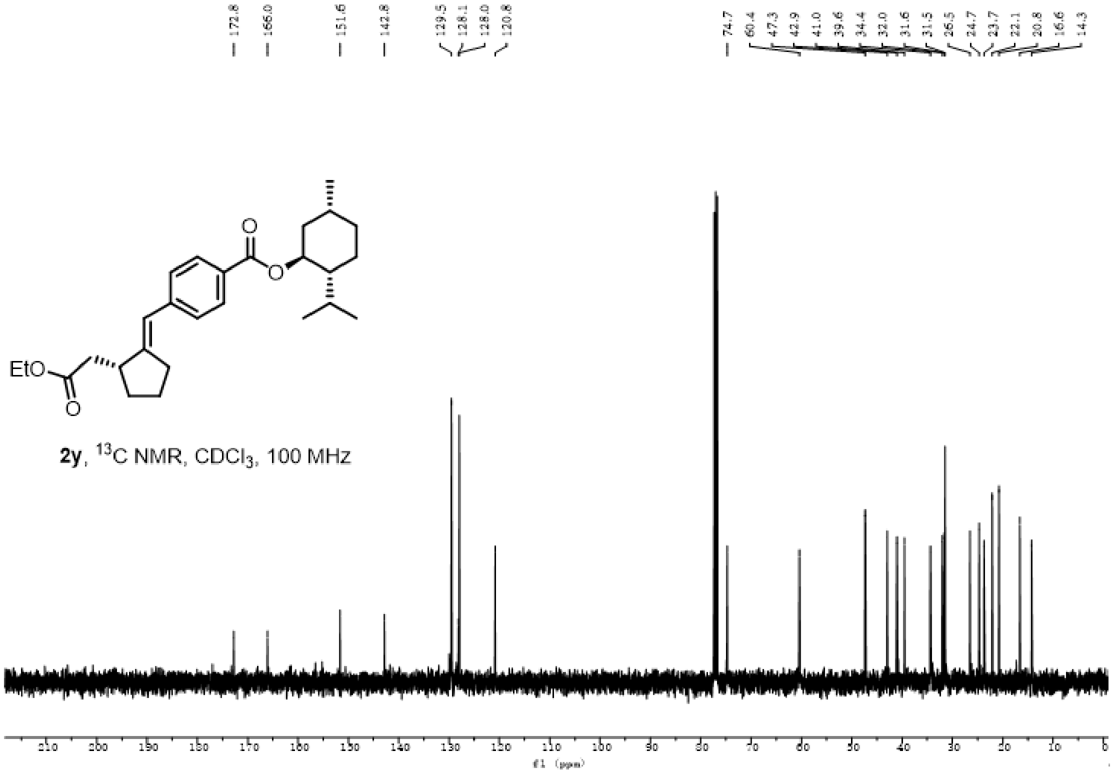

Supplementary Figure 118. ^{13}C NMR (400 MHz, $CDCl_3$, 25 °C) spectra for **2y**

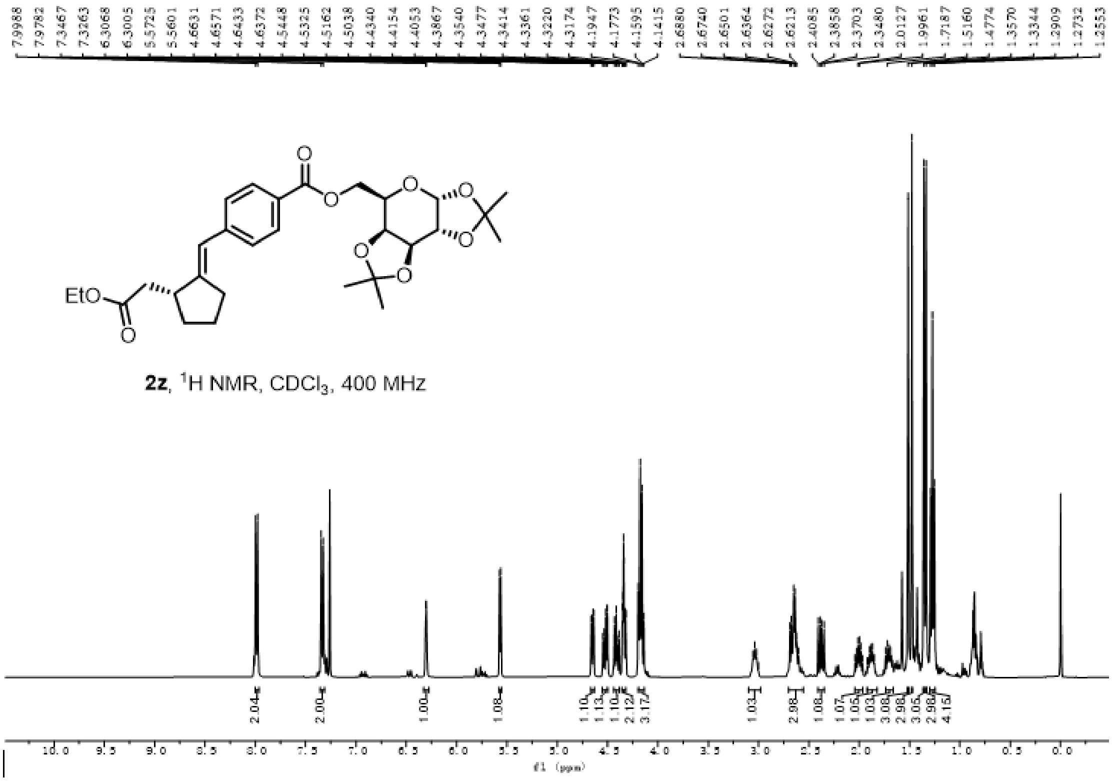

Supplementary Figure 119. ^{1}H NMR (400 MHz, $CDCl_3$, 25 °C) spectra for **2z**

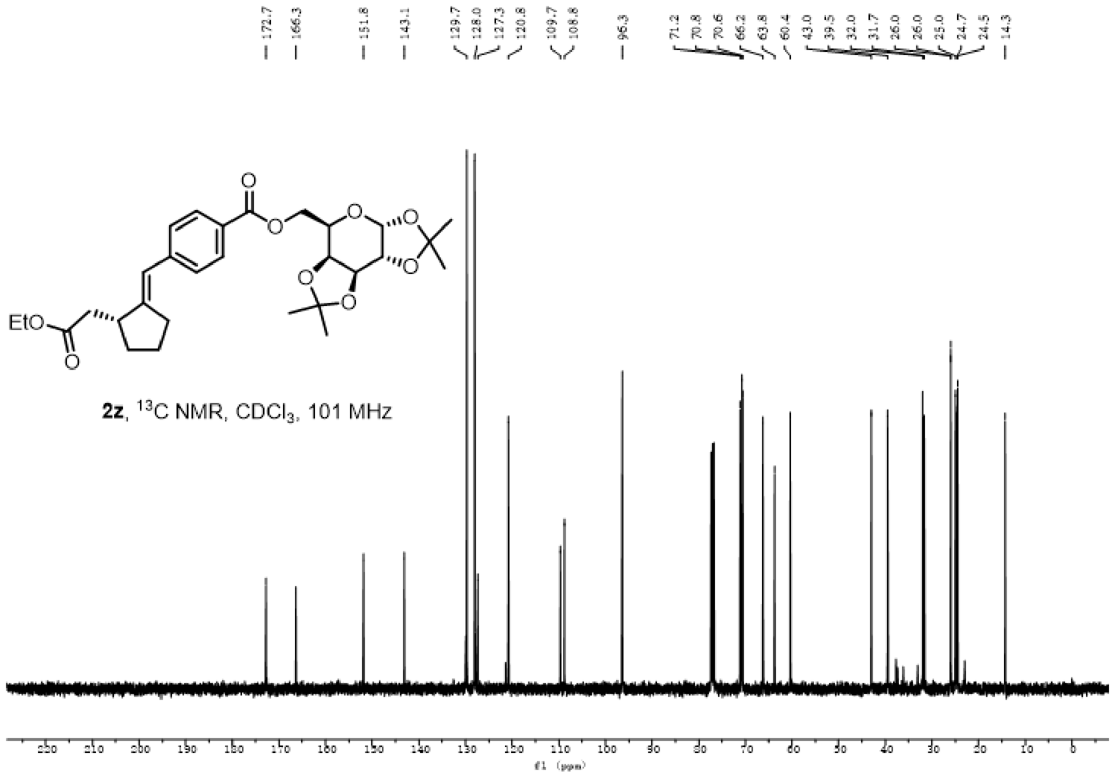

Supplementary Figure 120. ^{13}C NMR (400 MHz, $CDCl_3$, 25 °C) spectra for **2z**

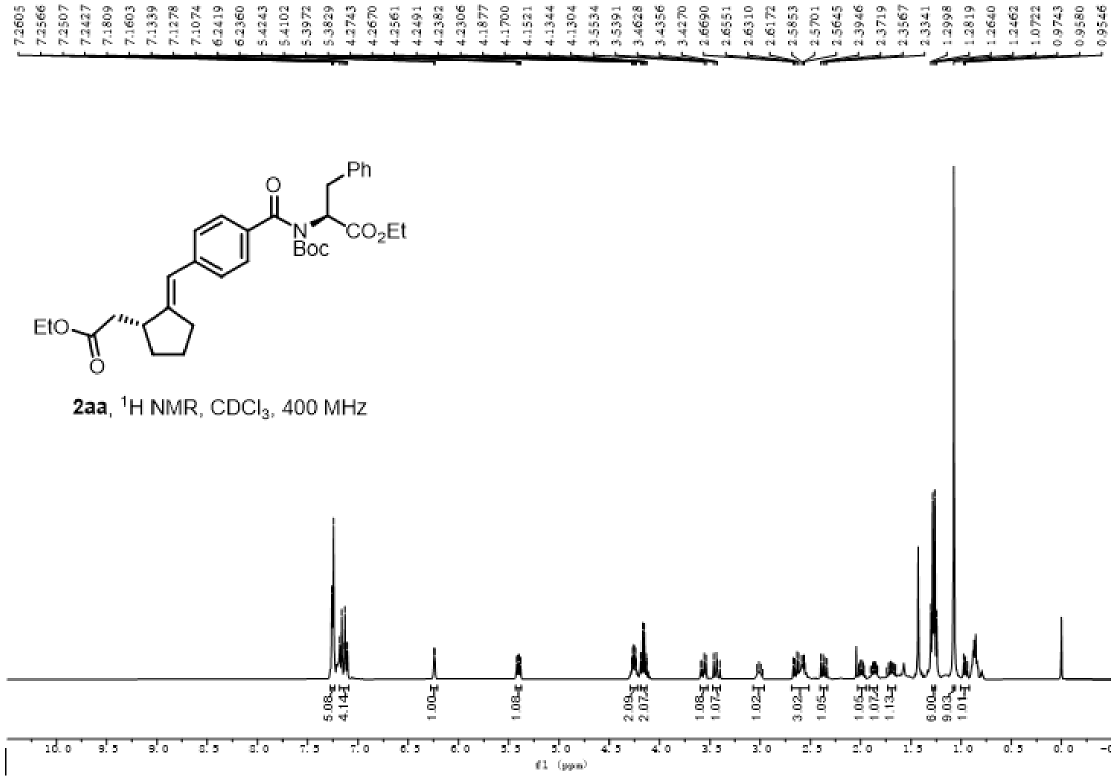

Supplementary Figure 121. ^{1}H NMR (400 MHz, $CDCl_3$, 25 °C) spectra for **2aa**

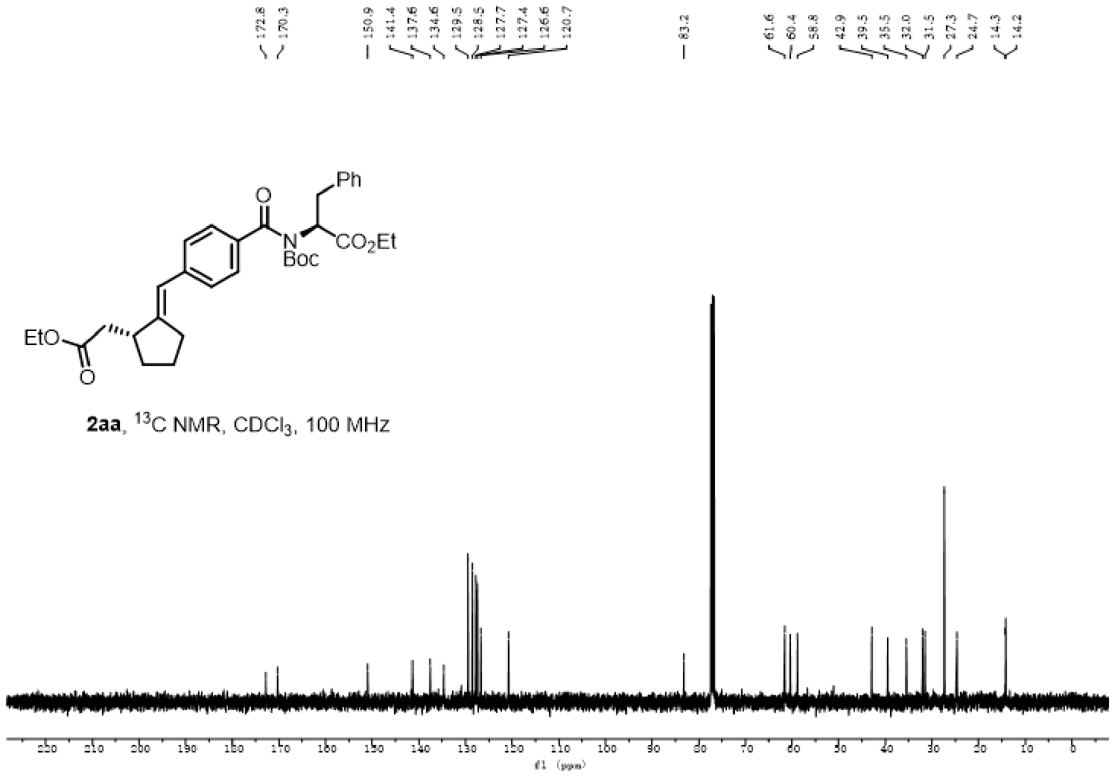

Supplementary Figure 122. ^{13}C NMR (400 MHz, $CDCl_3$, 25 °C) spectra for **2aa**

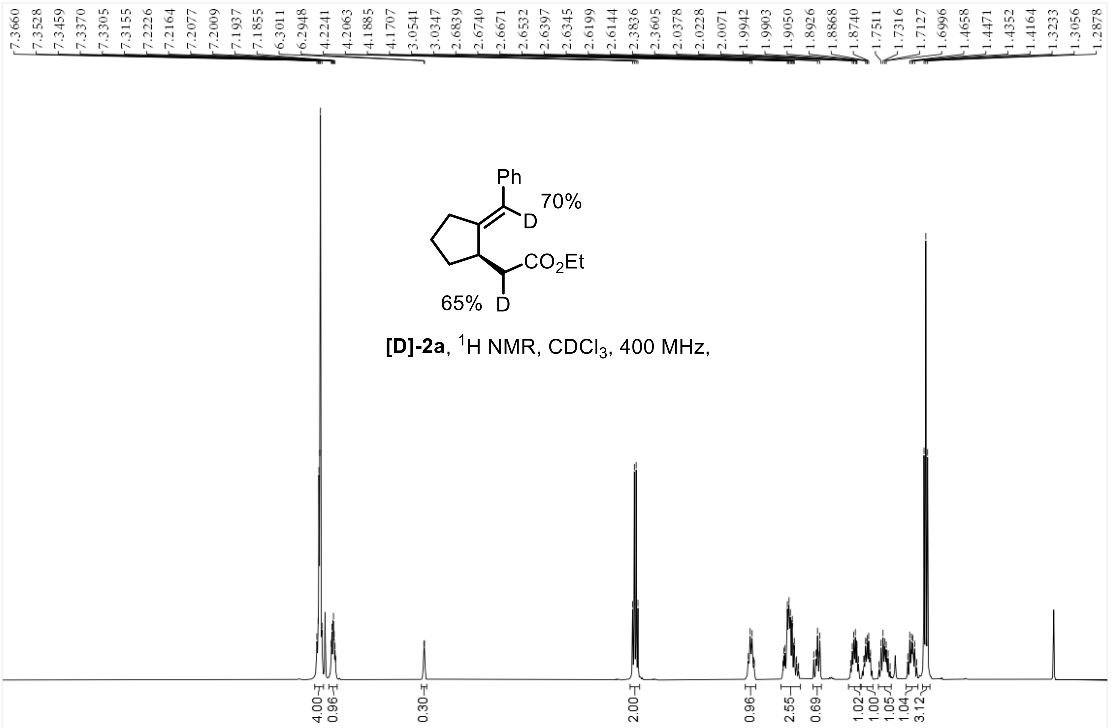

Supplementary Figure 123. ^{1}H NMR (400 MHz, $CDCl_3$, 25 °C) spectra for **[D]-2a**

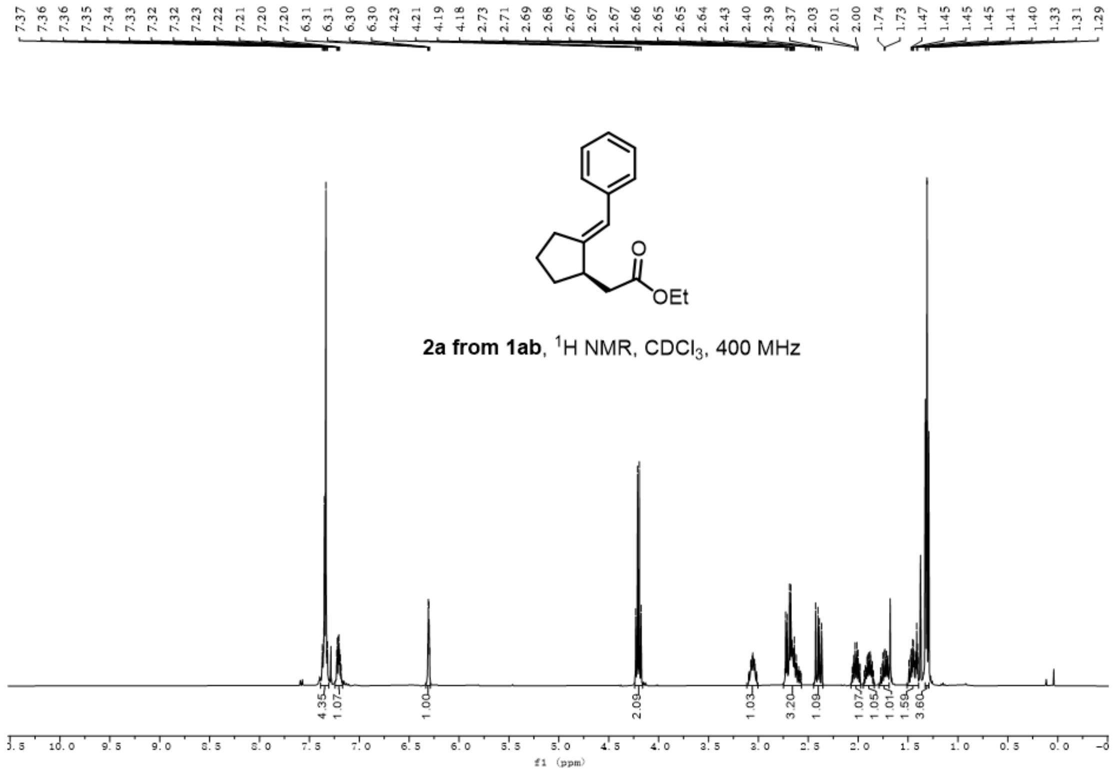

Supplementary Figure 124. ^{1}H NMR (400 MHz, CDCl$_3$, 25 °C) spectra for **2a** from **1ab**

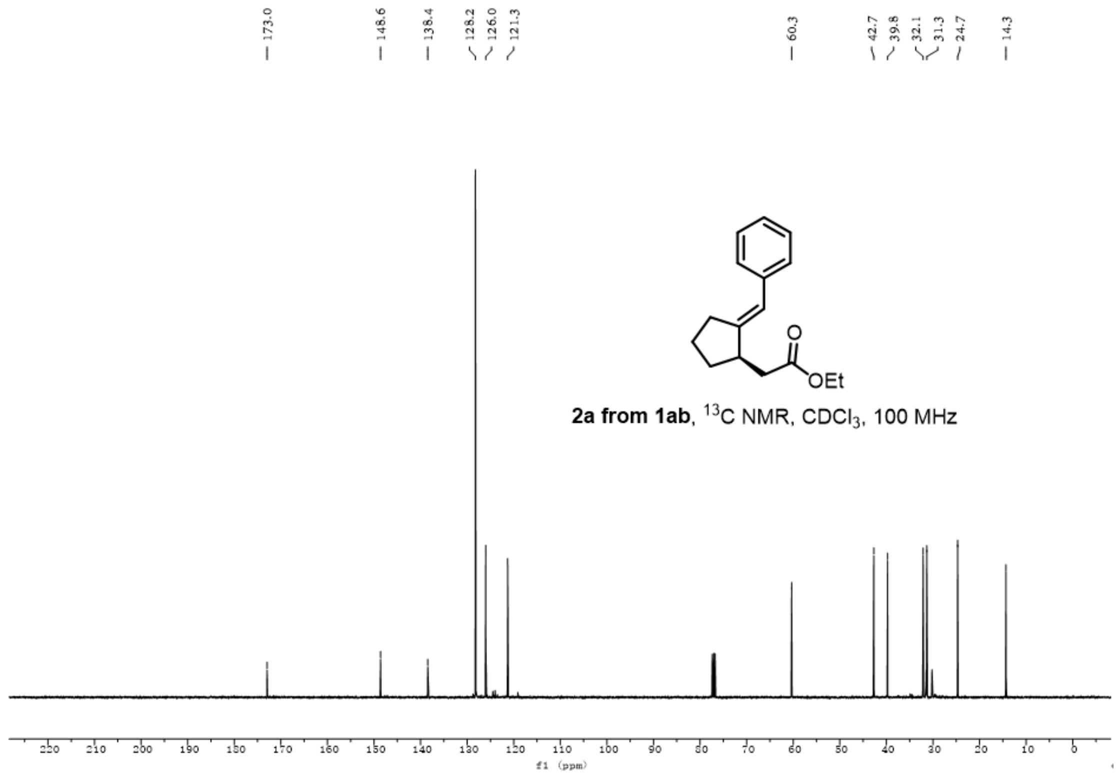

Supplementary Figure 125. ^{13}C NMR (400 MHz, CDCl$_3$, 25 °C) spectra for **2a** from **1ab**

4. Supplementary reference

1. Wang, D.-S.; Zhou, J.; Wang, D.-W.; Guo, Y.-L.; Zhou, Y.-G., *Tetrahedron Lett.* **2010**, *51*, 525-528.
2. Fernández, D. F.; Rodrigues, C. A. B.; Calvelo, M.; Gulías, M.; Mascareñas, J. L.; López, F., *ACS Catal.* **2018**, *8*, 7397-7402.
3. Mutra, M. R.; Dhandabani, G. K.; Li, J.; Wang, J.-J., *Chem. Commun.* **2020**, *56*, 2051-2054.
4. Martínez, C.; Aurrecoechea, J. M.; Madich, Y.; Denis, J. G.; de Lera, A. R.; Álvarez, R., *Eur. J. Org. Chem.* **2012**, *2012*, 99-106.
5. Grafton, M. W.; Farrugia, L. J.; Sutherland, A., *J. Org. Chem.* **2013**, *78*, 7199-7207.
6. Takahashi, K.; Honda, T., *Org. Lett.* **2010**, *12*, 3026-3029.
7. Spandl, R. J.; Rudyk, H.; Spring, D. R., *Chem. Commun.* **2008**, 3001-3003.
8. CCDC 2184064 (**2w**) contains the supplementary crystallographic data for this paper. These data can be obtained free of charge from The Cambridge Crystallographic Data Centre.
9. Frisch, M. J.; Trucks, G. W.; Schlegel, H. B.; Scuseria, G. E.; Robb, M. A.; Cheeseman, J.; R. Cooke, S.; Scalmani, G.; Barone, V.; Mennucci, B.; Petersson, G. A.; Nakatsuji, H.; aricato, M.; Li, X.; Hratchian, H. P.; Izmaylov, A. F.; Bloino, J.; Zheng, G.; Sonnenberg, J. L.; Hada, M.; Ehara, M.; Toyota, K.; Fukuda, R.; Hasegawa, J.; Ishida, M.; Nakajima, T.; Honda, Y.; Kitao, O.; Nakai, H.; Vreven, T.; Montgomery, J.; J. A.; Peralta, J. E.; Ogliaro, F.; Bearpark, M.; Heyd, J. J.; Brothers, E.; Kudin, K. N.; Staroverov, V. N.; Kobayashi, R.; Normand, J.; Raghavachari, K.; Rendell, A.; Burant, J. C.; Iyengar, S. S.; Tomasi, J.; Cossi, M.; Rega, N.; Millam, N. J.; Klene, M.; Knox, J. E.; Cross, J. B.; Bakken, V.; Adamo, C.; Jaramillo, J.; Gomperts, R.; Stratmann, R. E.; Yazyev, O.; Austin, A. J.; Cammi, R.; Pomelli, C.; Ochterski, J. W.; Martin, R. L.; Morokuma, K.; Zakrzewski, V. G.; Voth, G. A.; Salvador, P.; Dannenberg, J. J.; Dapprich, S.; Daniels, A. D.; Farkas, Ö.; Foresman, J. B.; Ortiz, J. V.; Cioslowski, J.; Fox, D. J. G., Revision D.01, Gaussian, Inc., Wallingford CT **2010.**
10. (a) Zhao, Y.; Truhlar, D. G. *J. Phys. Chem. A.* **2006**, *110*, 13126-13130. (b) Zhao,

Y.; Truhlar, D. G. *Acc. Chem. Res.* **2008**, *41*, 157-167. (c) Zhao, Y.; Truhlar, D. G. *Chem. Phys. Lett.* **2011**, *502*, 1-13.

11. Dolg, M.; Wedig, U.; Stoll, H.; Preuss, H. *J. Chem. Phys.* **1987**, *86*, 866-872.
12. Hehre, W.; Radom, L., P. v. R. Schleyer and JA Pople, Wiley, New York: 1986.
13. Zhao, Y.; Truhlar, D. G. *Theor. Chem. Acc.* **2008**, *119*, 525-525.
14. Tomasi, J.; Mennucci, B.; Cammi, R. *Chem. Rev.* **2005**, *105*, 2999-3094.
15. Lu, T., sobMECP program, http://sobereva.com/286.
16. Jiang, Y.-Y.; Jiang, J.-L.; Fu, Y. *Organometallics* **2016**, *35*, 3388-3396.
17. Leung, B. O.; Reid, D. L.; Armstrong, D. A.; Rauk, A. *J. Phys. Chem. A.* **2004**, *108*, 2720-2725.
18. Martin, R. L.; Hay, P. J.; Pratt, L. R. *J. Phys. Chem. A.* **1998**, *102*, 3565-3573.
19. (a) Li, H.; Jiang, J.; Lu, G.; Huang, F.; Wang, Z.-X. *Organometallics* **2011**, *30*, 3131-3141. (b) Li, H.; Wen, M.; Wang, Z.-X. *Inorg. Chem.* **2012**, *51*, 5716-5727. (c) Qu, S.; Dang, Y.; Song, C.; Wen, M.; Huang, K.-W.; Wang, Z.-X. *J. Am. Chem. Soc.* **2014**, *136*, 4974-4991. (d) Yu, J.-L.; Zhang, S.-Q.; Hong, X. *J. Am. Chem. Soc.* **2017**, *139*, 7224-7243.
