# Supplementary material for: Cobalt-catalyzed enantioselective intramolecular reductive cyclization via electrochemistry
Source: Nat Commun. 2023 Mar 9;14:1301. doi: 10.1038/s41467-023-36704-9 (PMC9998880; doi:10.1038/s41467-023-36704-9)
Supplement: Supplementary file 2 — Description of Additional Supplementary Files [file 41467_2023_36704_MOESM2_ESM.docx]

**Description of Additional Supplementary Files**

**File Name: Supplementary Data 1
Description:** Crystallographic data for compound **2w**, CCDC reference 2184064.

**File Name: Supplementary Data 2
Description:** Energy data (hartrees) for the calculated structures.
