# Supplementary material for: Cobalt-catalyzed enantioselective intramolecular reductive cyclization via electrochemistry
Source: Nat Commun. 2023 Mar 9;14:1301. doi: 10.1038/s41467-023-36704-9 (PMC9998880; doi:10.1038/s41467-023-36704-9)
Supplement: Supplementary file 3 — Supplementary Data 1 [file 41467_2023_36704_MOESM3_ESM.pdf]

Crystal data and structure refinement for **2w**.

cu_GSQ_3_354_a

Submitted by: **None**

None

Solved by: **None**

Sample ID: **cu_GSQ_3_354_a**

R_1=4.89%

Crystal Data and Experimental

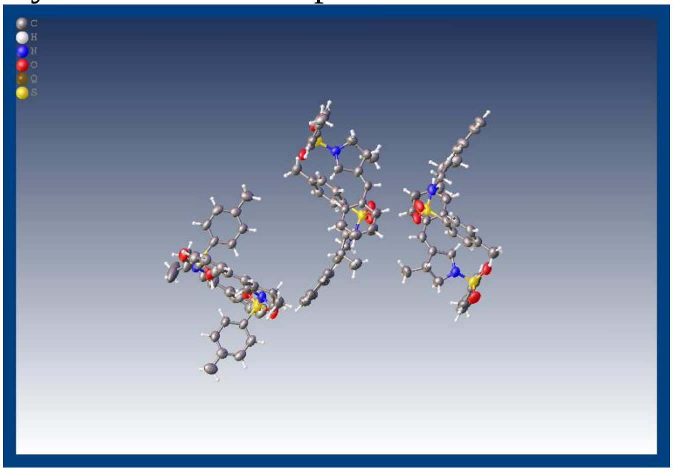

Experimental. Single colourless block-shaped crystals of **cu_GSQ_3_354_a** were obtained by recrystallisation from .... A suitable crystal 0.08×0.06×0.05 mm^3 was selected and mounted on a suitable support on an Bruker APEX-II CCD diffractometer. The crystal was kept at a steady T = 200.00 K during data collection. The structure was solved with the ShelXT 2018/2 (Sheldrick, 2018) structure solution program using the Intrinsic Phasing solution method and by using **Olex2** (Dolomanov et al., 2009) as the graphical interface. The model was refined with version 2018/3 of ShelXL 2018/3 (Sheldrick, 2015) using Least Squares minimisation.

Crystal Data. $C_{19}H_{21}NO_2S$, M_r = 327.43, triclinic, P1 (No. 1), a = 13.8015(5) Å, b = 14.1636(5) Å, c = 14.6393(5) Å, α = 96.515(2)°, β = 94.936(2)°, γ = 111.946(2)°, V = 2611.42(16) Å^3, T = 200.00 K, Z = 6, Z' = 6, μ(CuK_α) = 1.717, 195320 reflections measured, 18164 unique (R_{int} = 0.0688) which were used in all calculations. The final wR_2 was 0.1362 (all data) and R_1 was 0.0489 (I > 2(I)).

**Compound**	**cu_GSQ_3_354_a**
Formula	$C_{19}H_{21}NO_2S$
$D_{calc.}$/ g cm^{-3}	1.249
μ/mm^{-1}	1.717
Formula Weight	327.43
Colour	colourless
Shape	block
Size/mm^3	0.08×0.06×0.05
T/K	200.00
Crystal System	triclinic
Flack Parameter	0.069(6)
Hooft Parameter	0.069(6)
Space Group	P1
a/Å	13.8015(5)
b/Å	14.1636(5)
c/Å	14.6393(5)
α/°	96.515(2)
β/°	94.936(2)
γ/°	111.946(2)
V/Å^3	2611.42(16)
Z	6
Z'	6
Wavelength/Å	1.54178
Radiation type	CuK_α
Θ_{min}/°	3.068
Θ_{max}/°	67.093
Measured Refl.	195320
Independent Refl.	18164
Reflections with I > 2(I)	16326
R_{int}	0.0688
Parameters	1255
Restraints	22
Largest Peak	0.608
Deepest Hole	-0.603
GooF	1.024
wR_2 (all data)	0.1362
wR_2	0.1299
R_1 (all data)	0.0550
R_1	0.0489

Structure Quality Indicators

Reflections:

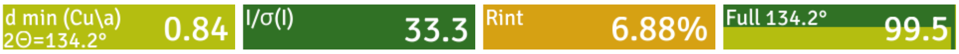

Refinement:

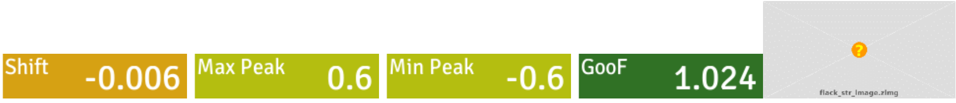

Experimental Extended. A colourless block-shaped crystal with dimensions 0.08×0.06×0.05 mm^3 was mounted on a suitable support. Data were collected using an Bruker APEX-II CCD diffractometer operating at T = 200.00 K.

Data were measured using ϕ and ω scans using CuK$_\alpha$ radiation. The maximum resolution that was achieved was Θ = 67.093° (0.84 Å).

The diffraction pattern was indexed and the unit cell was refined using SAINT V8.38A (Bruker, 2018) on 9347 reflections, 5% of the observed reflections.

Data reduction, scaling and absorption corrections were performed using SAINT V8.38A (Bruker, 2018). The final completeness is 99.50 % out to 67.093° in Θ. No absorption correction was performed. The absorption coefficient μ of this material is 1.717 mm^{-1} at this wavelength (λ = 1.542Å) and the minimum and maximum transmissions are 0 and 0.

The structure was solved and the space group P1 (# 1) determined by the ShelXT 2018/2 (Sheldrick, 2018) structure solution program using Intrinsic Phasing and refined by Least Squares using version 2018/3 of ShelXL 2018/3 (Sheldrick, 2015). All non-hydrogen atoms were refined anisotropically. Hydrogen atom positions were calculated geometrically and refined using the riding model. Hydrogen atom positions were calculated geometrically and refined using the riding model.

Table: Fractional Atomic Coordinates (×10^4) and Equivalent Isotropic Displacement Parameters (Å^2×10^3) for **cu_GSQ_3_354_a**. U_{eq} is defined as 1/3 of the trace of the orthogonalised U_{ij}.

Atom	x	y	z	U_{eq}
S001	6596.7(9)	4978.2(9)	2203.6(9)	53.2(3)
S002	5248.7(10)	11422.2(10)	4559.6(10)	62.5(3)
S003	9186.8(10)	2541.2(9)	8033.6(10)	61.3(3)
S004	8650.7(10)	-1396.7(9)	6573.5(9)	56.5(3)
S005	995.6(9)	6169.0(10)	2477.8(10)	60.1(3)
S006	2520.7(10)	-145.1(11)	-18.2(10)	65.6(4)
O007	9448(3)	1860(3)	7394(3)	69.5(11)
O008	8244(3)	-2474(3)	6631(3)	74.6(11)
N009	7912(3)	2157(3)	7859(3)	57.5(10)
O00A	3048(3)	-360(3)	771(3)	77.5(12)
O00B	5580(3)	4957(3)	1875(3)	68.1(10)
O00C	50(3)	5398(3)	2683(3)	71.2(11)
O00D	2006(3)	6205(3)	2824(3)	74.9(12)
O00E	7533(3)	5766(3)	2012(3)	62.6(9)
N00F	900(3)	6001(3)	1354(3)	59.0(11)
O00G	2738(4)	-391(4)	-946(3)	89.2(14)
C00H	8125(4)	12514(4)	3750(4)	56.3(12)
O00I	4781(3)	11672(3)	3758(3)	75.3(11)
O00J	9606(4)	3620(3)	8013(4)	82.8(13)
C00K	7285(4)	1044(4)	7773(4)	55.1(12)
N00L	4834(3)	10186(3)	4413(3)	55.5(10)
C00M	9996(4)	2046(5)	10971(4)	62.8(14)
C00N	7039(4)	12138(4)	3745(4)	56.0(12)
C00O	6203(4)	2067(4)	7968(3)	58.3(12)
O00P	8452(3)	-691(3)	7241(3)	63.6(9)

Atom	x	y	z	U_{eq}
O00Q	5063(4)	11730(4)	5469(3)	86.3(13)
C00R	6246(4)	4615(4)	4765(4)	60.4(13)
C00S	9694(4)	1244(4)	10231(4)	60.8(14)
C00T	7534(4)	-1947(4)	3867(5)	67.6(16)
C00U	7555(4)	3668(4)	1672(4)	52.1(11)
C00V	9545(4)	2356(4)	9166(4)	52.1(12)
C00W	-1015(5)	-1582(4)	44(4)	65.2(14)
C00X	4633(5)	8180(5)	588(5)	69.4(15)
C00Y	148(4)	5995(4)	-147(4)	56.1(12)
C00Z	5667(4)	2925(4)	1524(4)	53.8(12)
C010	5825(4)	4324(4)	3730(4)	59.3(12)
C011	2754(4)	1581(4)	1080(4)	52.8(11)
N012	2811(3)	1097(3)	152(3)	56.0(10)
C013	-115(4)	5830(4)	810(4)	54.1(12)
C014	4490(4)	8306(4)	1504(4)	57.0(12)
C015	9482(4)	1390(4)	9334(4)	53.6(12)
N016	6689(3)	5061(3)	3323(3)	49.7(9)
C017	1161(4)	-744(4)	-9(4)	53.2(12)
C018	6612(4)	3779(4)	1759(3)	48.4(10)
C019	4607(4)	7634(4)	2089(4)	54.4(12)
C01A	8210(4)	-300(4)	5291(4)	54.2(12)
C01B	8376(5)	12474(5)	5367(4)	67.4(15)
C01C	7363(5)	2720(4)	8361(4)	63.9(13)
C01D	4812(4)	9622(4)	3488(4)	52.0(11)
C01E	9867(4)	3175(4)	9892(5)	62.6(14)
C01F	13626(4)	2832(5)	6689(4)	66.6(15)
C01G	12625(4)	2083(5)	6722(4)	64.4(14)
C01H	982(4)	7386(4)	2857(4)	52.4(12)
C01I	1926(4)	8246(4)	3074(4)	58.3(13)
C01J	5670(4)	1981(4)	1181(4)	58.3(13)
C01K	452(4)	-975(4)	-821(4)	61.2(13)
C01L	7839(4)	-2082(4)	4744(5)	62.3(14)
C01M	-490(4)	5957(4)	-880(4)	60.1(13)
C01N	2944(4)	3514(4)	2556(4)	51.5(11)
C01O	4072(5)	-2733(5)	7748(5)	74.8(16)
C01P	4575(4)	8533(4)	3682(4)	53.1(11)
C01Q	6628(4)	11934(4)	4550(4)	51.9(11)
C01R	63(4)	8466(5)	3268(4)	63.6(14)
C01S	2929(4)	3427(4)	1546(4)	57.2(12)
C01T	7708(4)	5225(4)	3844(3)	50.9(11)
C01U	2840(4)	2637(4)	921(4)	58.4(12)
N01V	9921(3)	-1016(3)	6648(3)	58.5(11)
C01W	-2122(5)	5854(5)	-1814(4)	70.3(15)
C01X	4498(4)	7724(4)	3072(4)	56.0(12)
C01Y	7548(4)	2728(4)	1316(4)	58.5(13)
C01Z	-1625(4)	5716(4)	-994(4)	55.9(12)
C020	-2297(4)	5332(5)	-327(4)	61.8(13)
C021	4866(5)	-3043(5)	7565(5)	75.7(16)
C022	5841(5)	-2326(5)	7524(5)	78.9(18)
C023	5904(5)	5453(5)	5192(4)	77.5(16)
C024	2870(5)	3801(6)	4457(5)	79.4(18)
C025	-2202(5)	-1992(6)	56(6)	92(2)
C026	8182(4)	-1252(4)	5466(4)	54.5(12)
C027	6618(4)	1866(4)	1067(4)	55.5(12)
C028	756(5)	-967(4)	821(4)	60.2(13)
C029	10985(5)	5966(5)	5160(5)	72.0(16)
C02A	7368(5)	-822(6)	2680(5)	84.1(19)
C02B	4236(4)	-1701(5)	7889(4)	64.2(14)
C02C	2763(5)	4355(5)	2993(5)	69.3(15)
C02D	991(4)	9328(5)	3499(4)	61.0(13)
C02E	4849(5)	6823(5)	1707(5)	69.0(15)
C02F	9915(5)	5708(5)	4988(4)	65.6(14)
C02G	2407(5)	1555(5)	-565(4)	68.5(15)
C02H	2764(5)	2685(5)	-123(4)	75.8(15)
C02I	1921(4)	9193(5)	3386(4)	60.5(13)

Atom	x	y	z	U_{eq}
C02J	6017(4)	-1287(5)	7625(5)	77.2(18)
C02K	32(4)	7496(5)	2956(4)	61.0(14)
C02L	7289(5)	12098(4)	5377(4)	63.3(14)
C02M	7921(4)	-177(4)	4398(4)	58.5(13)
C02N	5348(4)	127(5)	7925(4)	62.1(13)
C02O	11593(5)	135(5)	6472(6)	83(2)
C02P	-3362(5)	5122(5)	-473(4)	71.0(16)
C02Q	11438(6)	5800(6)	5969(5)	83.8(19)
C02R	7438(4)	4968(4)	4782(4)	54.2(12)
C02S	5084(5)	9627(4)	5133(4)	63.4(13)
C02T	4440(5)	8511(4)	4685(4)	64.5(13)
C02U	12461(5)	1013(5)	6474(5)	81.8(19)
C02V	5218(4)	-940(4)	7818(4)	59.1(12)
C02W	3119(4)	2843(4)	3110(4)	57.0(12)
C02X	1761(4)	6648(4)	884(5)	64.8(14)
C02Y	9265(5)	5296(4)	5634(4)	60.6(13)
C02Z	2711(6)	4474(6)	3937(5)	85(2)
C030	6624(6)	839(5)	671(5)	81.5(18)
C031	8806(4)	12678(4)	4572(4)	60.9(13)
C032	9717(5)	5140(5)	6454(4)	70.8(15)
C033	5424(5)	2263(5)	8588(4)	68.8(14)
C034	6179(4)	976(4)	7888(4)	57.4(12)
C035	10799(6)	5379(6)	6617(5)	85(2)
C036	10384(5)	-1598(5)	6017(6)	88(2)
C037	10099(4)	3021(4)	10784(5)	67.4(16)
C038	4905(6)	7392(6)	231(5)	81.7(18)
C039	-3809(5)	5258(6)	-1299(5)	75.4(16)
C03A	990(6)	10361(6)	3857(5)	83.0(18)
C03B	-315(5)	-1376(4)	821(4)	65.0(14)
C03C	-3176(5)	5623(6)	-1967(5)	78.3(17)
C03D	8112(5)	5000(4)	5513(4)	62.0(13)
C03E	7599(4)	-984(4)	3655(4)	61.2(14)
C03F	4999(6)	6706(6)	804(5)	88(2)
C03G	10182(5)	1860(6)	11940(5)	86(2)
C03H	1315(4)	6227(5)	-135(5)	69.2(15)
C03I	11867(5)	2436(5)	6972(6)	81.0(19)
C03J	-600(5)	-1380(5)	-784(4)	66.6(15)
C03K	3795(6)	3346(6)	-386(5)	101(2)
C03L	9974(5)	13063(6)	4562(5)	88(2)
C03M	3094(4)	2983(5)	4050(4)	67.2(14)
C03N	13078(5)	4177(5)	7180(7)	97(2)
C03O	10541(4)	90(4)	6707(5)	61.6(13)
C03P	13829(5)	3854(5)	6911(5)	81.0(18)
C03Q	1846(5)	6900(5)	-824(5)	89.0(19)
C03R	4772(6)	7732(5)	5146(4)	86.5(18)
C03S	12079(5)	3454(5)	7204(7)	96(2)
C03T	11513(6)	-922(6)	6136(8)	120(3)
C03U	12208(9)	-1256(8)	6622(13)	226(7)

Table: Anisotropic Displacement Parameters ($\times 10^4$) **cu_GSQ_3_354_a**. The anisotropic displacement factor exponent takes the form: $-2\pi^2[h^2a^{*2} \times U_{11} + ... + 2hka^* \times b^* \times U_{12}]$

Atom	U_{11}	U_{22}	U_{33}	U_{23}	U_{13}	U_{12}
S001	50.0(6)	45.5(6)	64.9(7)	17.0(5)	2.9(5)	18.2(5)
S002	62.9(7)	56.2(7)	78.8(9)	2.5(6)	18.9(6)	35.1(6)
S003	66.4(7)	47.6(7)	82.0(9)	21.5(6)	35.2(7)	27.2(6)
S004	62.1(7)	45.0(6)	67.6(8)	13.3(5)	24.9(6)	21.9(5)
S005	45.8(6)	58.2(7)	79.3(9)	27.9(6)	3.7(6)	19.5(5)
S006	58.9(7)	65.3(8)	75.9(9)	-10.3(7)	4.8(6)	35.0(6)
O007	73(2)	66(2)	84(3)	19.2(19)	43(2)	34.2(19)
O008	86(3)	48(2)	93(3)	26(2)	34(2)	20.9(19)
N009	62(2)	53(2)	73(3)	19(2)	25(2)	35(2)
O00A	73(2)	66(2)	100(3)	-4(2)	-13(2)	45(2)
O00B	60(2)	63(2)	89(3)	18(2)	-4.1(19)	33.8(18)

Atom	U_{11}	U_{22}	U_{33}	U_{23}	U_{13}	U_{12}
O00C	56(2)	67(2)	82(3)	34(2)	6.4(18)	9.6(18)
O00D	53(2)	69(2)	110(3)	32(2)	-6(2)	28.8(18)
O00E	60(2)	48.7(19)	71(2)	24.6(17)	2.4(17)	9.4(16)
N00F	45(2)	54(2)	81(3)	20(2)	11(2)	18.9(18)
O00G	79(3)	96(3)	93(3)	-22(2)	25(2)	43(2)
C00H	60(3)	59(3)	53(3)	5(2)	10(2)	27(2)
O00I	63(2)	64(2)	112(3)	13(2)	8(2)	40.2(19)
O00J	94(3)	48(2)	115(4)	32(2)	48(3)	26(2)
C00K	55(3)	55(3)	67(3)	11(2)	15(2)	32(2)
N00L	55(2)	53(2)	64(3)	6.2(19)	16.1(19)	26.5(19)
C00M	38(2)	79(4)	73(4)	6(3)	8(2)	27(2)
C00N	69(3)	56(3)	49(3)	5(2)	2(2)	33(3)
C00O	68(3)	74(3)	51(2)	13(2)	11(2)	45(3)
O00P	70(2)	59(2)	69(2)	7.7(17)	30.3(18)	30.3(17)
O00Q	100(3)	84(3)	88(3)	-5(2)	44(2)	49(3)
C00R	63(3)	54(3)	67(3)	15(2)	20(2)	20(2)
C00S	45(3)	52(3)	87(4)	9(3)	7(2)	22(2)
C00T	42(3)	58(3)	88(4)	-14(3)	14(3)	10(2)
C00U	40(2)	57(3)	58(3)	18(2)	9(2)	15(2)
C00V	45(2)	39(2)	70(3)	4(2)	17(2)	13.6(19)
C00W	64(3)	48(3)	75(4)	-2(3)	9(3)	15(2)
C00X	61(3)	65(3)	79(4)	8(3)	-5(3)	24(3)
C00Y	51(3)	44(2)	72(3)	3(2)	17(2)	16(2)
C00Z	38(2)	59(3)	64(3)	10(2)	4(2)	20(2)
C010	53(3)	56(3)	65(3)	13(2)	14(2)	15(2)
C011	54(3)	53(3)	55(3)	1(2)	7(2)	26(2)
N012	52(2)	59(2)	60(2)	-1.0(19)	9.7(19)	27.4(19)
C013	47(3)	50(3)	66(3)	11(2)	13(2)	19(2)
C014	54(3)	53(3)	64(3)	3(2)	-1(2)	25(2)
C015	47(2)	45(3)	71(3)	5(2)	10(2)	21(2)
N016	45(2)	43(2)	60(2)	12.4(17)	8.9(17)	14.3(16)
C017	64(3)	43(3)	52(3)	-6(2)	0(2)	25(2)
C018	43(2)	54(3)	47(3)	14(2)	3.4(19)	17(2)
C019	41(2)	44(2)	77(3)	4(2)	1(2)	19(2)
C01A	52(3)	45(3)	67(3)	2(2)	13(2)	22(2)
C01B	71(3)	66(3)	57(3)	-12(3)	-14(3)	27(3)
C01C	80(3)	52(3)	76(3)	13(2)	26(3)	39(3)
C01D	46(2)	47(3)	65(3)	7(2)	11(2)	21(2)
C01E	52(3)	45(3)	87(4)	4(3)	26(3)	14(2)
C01F	47(3)	86(4)	61(3)	11(3)	12(2)	19(3)
C01G	49(3)	67(3)	76(4)	6(3)	9(2)	23(2)
C01H	41(2)	57(3)	62(3)	24(2)	7(2)	19(2)
C01I	38(2)	71(3)	67(3)	20(3)	3(2)	21(2)
C01J	43(2)	58(3)	66(3)	2(3)	3(2)	14(2)
C01K	69(3)	60(3)	46(3)	-7(2)	-1(2)	22(3)
C01L	55(3)	42(3)	87(4)	1(3)	21(3)	15(2)
C01M	63(3)	55(3)	55(3)	0(2)	20(3)	15(2)
C01N	40(2)	47(3)	69(3)	6(2)	6(2)	20(2)
C01O	59(3)	78(4)	81(4)	15(3)	14(3)	17(3)
C01P	44(2)	49(2)	68(3)	10(2)	9(2)	20(2)
C01Q	55(3)	46(3)	58(3)	0(2)	8(2)	25(2)
C01R	48(3)	81(4)	79(4)	33(3)	24(3)	35(3)
C01S	60(3)	48(3)	68(3)	10(2)	7(2)	26(2)
C01T	50(3)	46(2)	54(3)	5(2)	4(2)	18(2)
C01U	58(3)	51(3)	67(3)	10(2)	15(2)	21(2)
N01V	59(2)	51(2)	74(3)	9(2)	19(2)	29(2)
C01W	89(4)	63(3)	55(3)	13(3)	17(3)	23(3)
C01X	46(2)	48(3)	79(3)	19(2)	8(2)	22(2)
C01Y	48(3)	74(4)	61(3)	20(3)	11(2)	29(2)
C01Z	66(3)	46(3)	47(3)	-1(2)	6(2)	14(2)
C020	55(3)	74(3)	48(3)	10(2)	6(2)	17(2)
C021	57(3)	63(3)	100(4)	8(3)	10(3)	17(3)
C022	60(3)	56(3)	116(5)	7(3)	22(3)	17(3)
C023	64(3)	73(3)	88(4)	-8(3)	23(3)	22(3)
C024	72(4)	94(5)	72(4)	-5(3)	12(3)	36(3)

Atom	U_{11}	U_{22}	U_{33}	U_{23}	U_{13}	U_{12}
C025	64(4)	82(5)	111(6)	-9(4)	6(4)	14(3)
C026	43(2)	42(2)	79(4)	2(2)	20(2)	16(2)
C027	58(3)	63(3)	53(3)	10(2)	7(2)	31(2)
C028	74(3)	54(3)	55(3)	4(2)	-5(2)	31(3)
C029	68(3)	75(4)	66(4)	-4(3)	-5(3)	27(3)
C02A	58(3)	112(6)	80(4)	0(4)	1(3)	37(3)
C02B	46(3)	81(4)	66(3)	10(3)	11(2)	24(3)
C02C	70(3)	68(4)	81(4)	-1(3)	9(3)	43(3)
C02D	59(3)	72(4)	59(3)	21(3)	16(2)	30(3)
C02E	80(4)	58(3)	78(4)	6(3)	6(3)	39(3)
C02F	65(3)	67(3)	57(3)	3(3)	-3(3)	22(3)
C02G	71(4)	80(4)	57(3)	10(3)	15(3)	32(3)
C02H	90(4)	68(3)	69(3)	11(3)	17(3)	30(3)
C02I	48(3)	65(3)	66(3)	16(3)	3(2)	18(2)
C02J	48(3)	69(4)	117(5)	11(3)	25(3)	24(3)
C02K	39(2)	73(4)	74(3)	35(3)	13(2)	18(2)
C02L	78(4)	59(3)	50(3)	-3(2)	11(3)	27(3)
C02M	50(3)	54(3)	73(4)	2(2)	9(2)	24(2)
C02N	47(3)	82(4)	65(3)	5(3)	11(2)	34(3)
C02O	54(3)	69(3)	134(6)	6(3)	18(3)	35(2)
C02P	58(3)	80(4)	60(3)	15(3)	5(2)	10(3)
C02Q	76(4)	90(5)	82(5)	-7(4)	-13(3)	40(4)
C02R	68(3)	43(2)	50(3)	4(2)	11(2)	20(2)
C02S	66(3)	64(3)	62(3)	11(3)	15(2)	25(3)
C02T	69(3)	60(3)	68(3)	22(2)	19(3)	25(3)
C02U	50(3)	74(4)	126(6)	6(4)	22(3)	30(3)
C02V	51(3)	67(3)	57(3)	3(2)	1(2)	24(2)
C02W	52(3)	47(3)	71(4)	3(2)	5(2)	21(2)
C02X	48(3)	60(3)	91(4)	17(3)	22(3)	23(2)
C02Y	77(4)	52(3)	53(3)	-3(2)	-1(3)	31(3)
C02Z	88(4)	85(4)	93(5)	-11(4)	14(4)	53(4)
C030	92(5)	72(4)	91(5)	10(3)	23(4)	41(4)
C031	59(3)	50(3)	64(3)	-12(2)	-3(2)	19(2)
C032	85(4)	69(3)	60(3)	-2(3)	-4(3)	38(3)
C033	75(3)	78(4)	74(3)	10(3)	20(3)	52(3)
C034	61(3)	64(3)	59(3)	4(2)	8(2)	38(3)
C035	102(5)	89(5)	64(4)	-5(3)	-13(4)	47(4)
C036	81(3)	61(3)	136(6)	-1(3)	37(3)	43(2)
C037	41(3)	60(3)	92(4)	-13(3)	13(3)	15(2)
C038	88(4)	92(5)	72(4)	6(4)	16(3)	43(4)
C039	67(3)	88(4)	60(3)	13(3)	-2(3)	19(3)
C03A	91(5)	84(4)	84(4)	12(4)	21(4)	45(4)
C03B	78(4)	53(3)	63(3)	9(2)	10(3)	24(3)
C03C	81(4)	97(5)	56(3)	20(3)	0(3)	32(4)
C03D	78(4)	53(3)	58(3)	6(2)	14(3)	28(3)
C03E	37(2)	69(3)	74(4)	-2(3)	4(2)	20(2)
C03F	109(5)	89(5)	83(5)	-3(4)	15(4)	61(4)
C03G	61(3)	117(6)	79(4)	-2(4)	1(3)	40(4)
C03H	57(3)	61(3)	93(4)	9(3)	28(3)	24(2)
C03I	47(3)	56(3)	135(6)	7(3)	25(3)	15(2)
C03J	70(4)	67(3)	52(3)	-9(3)	-8(3)	24(3)
C03K	88(5)	113(6)	93(5)	20(4)	26(4)	26(4)
C03L	60(4)	85(5)	98(5)	-18(4)	1(3)	18(3)
C03M	57(3)	73(4)	67(4)	8(3)	7(3)	21(3)
C03N	67(4)	58(4)	151(7)	9(4)	31(4)	7(3)
C03O	54(3)	49(3)	87(4)	11(3)	17(3)	24(2)
C03P	59(3)	66(4)	102(5)	6(3)	20(3)	7(3)
C03Q	72(4)	86(4)	94(4)	2(3)	34(3)	13(3)
C03R	116(5)	77(4)	73(4)	29(3)	9(3)	41(4)
C03S	58(3)	57(4)	171(8)	12(4)	35(4)	19(3)
C03T	75(3)	75(3)	220(9)	1(4)	30(4)	45(3)
C03U	151(8)	91(6)	417(18)	-40(9)	-100(11)	78(6)

Table: Bond Lengths in Å for **cu_GSQ_3_354_a**.

Atom	Atom	Length/Å
S001	O00B	1.431(4)
S001	O00E	1.432(4)
S001	N016	1.619(4)
S001	C018	1.757(5)
S002	O00I	1.433(5)
S002	N00L	1.607(4)
S002	O00Q	1.427(4)
S002	C01Q	1.768(5)
S003	O007	1.432(4)
S003	N009	1.622(5)
S003	O00J	1.422(4)
S003	C00V	1.766(6)
S004	O008	1.431(4)
S004	O00P	1.435(4)
S004	N01V	1.620(4)
S004	C026	1.756(6)
S005	O00C	1.436(4)
S005	O00D	1.422(4)
S005	N00F	1.622(5)
S005	C01H	1.757(5)
S006	O00A	1.434(4)
S006	O00G	1.448(5)
S006	N012	1.635(5)
S006	C017	1.749(5)
N009	C00K	1.471(7)
N009	C01C	1.472(6)
N00F	C013	1.471(6)
N00F	C02X	1.483(7)
C00H	C00N	1.390(7)
C00H	C031	1.401(8)
C00K	C034	1.518(7)
N00L	C01D	1.483(7)
N00L	C02S	1.480(7)
C00M	C00S	1.388(8)
C00M	C037	1.395(9)
C00M	C03G	1.490(9)
C00N	C01Q	1.365(7)
C00O	C01C	1.538(8)
C00O	C033	1.545(7)
C00O	C034	1.524(7)
C00R	C010	1.527(8)
C00R	C023	1.524(7)
C00R	C02R	1.527(8)
C00S	C015	1.376(8)
C00T	C01L	1.372(9)
C00T	C03E	1.405(8)
C00U	C018	1.383(6)
C00U	C01Y	1.370(8)
C00V	C015	1.390(7)
C00V	C01E	1.387(8)
C00W	C025	1.523(9)
C00W	C03B	1.355(8)
C00W	C03J	1.398(9)
C00X	C014	1.373(9)
C00X	C038	1.369(9)
C00Y	C013	1.499(8)
C00Y	C01M	1.311(8)
C00Y	C03H	1.516(7)
C00Z	C018	1.390(7)
C00Z	C01J	1.375(7)
C010	N016	1.484(6)
C011	N012	1.473(6)
C011	C01U	1.501(7)
N012	C02G	1.469(7)

Atom	Atom	Length/Å
C014	C019	1.394(8)
N016	C01T	1.465(6)
C017	C01K	1.396(7)
C017	C028	1.401(8)
C019	C01X	1.456(8)
C019	C02E	1.384(7)
C01A	C026	1.388(7)
C01A	C02M	1.383(8)
C01B	C02L	1.394(8)
C01B	C031	1.362(9)
C01D	C01P	1.519(7)
C01E	C037	1.378(9)
C01F	C01G	1.402(8)
C01F	C03P	1.361(9)
C01G	C02U	1.444(9)
C01G	C03I	1.376(8)
C01H	C01I	1.392(7)
C01H	C02K	1.394(7)
C01I	C02I	1.370(8)
C01J	C027	1.399(7)
C01K	C03J	1.355(8)
C01L	C026	1.392(8)
C01M	C01Z	1.464(8)
C01N	C01S	1.468(8)
C01N	C02C	1.403(7)
C01N	C02W	1.389(8)
C01O	C021	1.361(9)
C01O	C02B	1.381(9)
C01P	C01X	1.335(7)
C01P	C02T	1.498(8)
C01Q	C02L	1.393(8)
C01R	C02D	1.382(8)
C01R	C02K	1.382(8)
C01S	C01U	1.322(7)
C01T	C02R	1.502(7)
C01U	C02H	1.535(8)
N01V	C036	1.504(7)
N01V	C03O	1.467(7)
C01W	C01Z	1.400(8)
C01W	C03C	1.359(9)
C01Y	C027	1.385(8)
C01Z	C020	1.418(7)
C020	C02P	1.378(8)
C021	C022	1.361(8)
C022	C02J	1.386(9)
C024	C02Z	1.352(10)
C024	C03M	1.392(9)
C027	C030	1.507(8)
C028	C03B	1.371(8)
C029	C02F	1.375(8)
C029	C02Q	1.377(9)
C02A	C03E	1.498(9)
C02B	C02V	1.403(8)
C02C	C02Z	1.385(10)
C02D	C02I	1.388(7)
C02D	C03A	1.497(9)
C02E	C03F	1.358(9)
C02F	C02Y	1.384(8)
C02G	C02H	1.531(9)
C02H	C03K	1.493(9)
C02J	C02V	1.401(7)
C02M	C03E	1.393(8)
C02N	C02V	1.442(8)
C02N	C034	1.326(8)

Atom	Atom	Length/Å
C02O	C02U	1.366(9)
C02O	C03O	1.501(7)
C02O	C03T	1.481(9)
C02P	C039	1.375(9)
C02Q	C035	1.382(11)
C02R	C03D	1.341(8)
C02S	C02T	1.527(8)
C02T	C03R	1.542(8)
C02W	C03M	1.372(8)
C02X	C03H	1.525(9)
C02Y	C032	1.383(8)

Atom	Atom	Length/Å
C02Y	C03D	1.475(8)
C031	C03L	1.499(8)
C032	C035	1.393(10)
C036	C03T	1.474(10)
C038	C03F	1.387(10)
C039	C03C	1.383(9)
C03H	C03Q	1.512(9)
C03I	C03S	1.356(9)
C03N	C03P	1.351(10)
C03N	C03S	1.382(9)
C03T	C03U	1.395(12)

Table: Bond Angles in ° for **cu_GSQ_3_354_a**.

Atom	Atom	Atom	Angle/°
O00B	S001	O00E	120.2(2)
O00B	S001	N016	107.4(2)
O00B	S001	C018	106.8(2)
O00E	S001	N016	106.4(2)
O00E	S001	C018	107.9(2)
N016	S001	C018	107.4(2)
O00I	S002	N00L	106.1(3)
O00I	S002	C01Q	107.1(3)
N00L	S002	C01Q	108.6(2)
O00Q	S002	O00I	120.9(3)
O00Q	S002	N00L	106.2(3)
O00Q	S002	C01Q	107.5(3)
O007	S003	N009	106.3(3)
O007	S003	C00V	107.7(2)
N009	S003	C00V	107.6(2)
O00J	S003	O007	120.3(2)
O00J	S003	N009	106.8(3)
O00J	S003	C00V	107.6(3)
O008	S004	O00P	120.0(2)
O008	S004	N01V	107.0(2)
O008	S004	C026	107.8(3)
O00P	S004	N01V	106.3(2)
O00P	S004	C026	107.6(2)
N01V	S004	C026	107.5(2)
O00C	S005	N00F	105.1(2)
O00C	S005	C01H	108.1(3)
O00D	S005	O00C	120.9(2)
O00D	S005	N00F	107.1(3)
O00D	S005	C01H	106.8(2)
N00F	S005	C01H	108.3(2)
O00A	S006	O00G	120.7(3)
O00A	S006	N012	106.5(2)
O00A	S006	C017	108.1(3)
O00G	S006	N012	106.1(3)
O00G	S006	C017	108.0(3)
N012	S006	C017	106.6(2)
C00K	N009	S003	117.7(3)
C00K	N009	C01C	108.7(4)
C01C	N009	S003	121.4(4)
C013	N00F	S005	118.6(3)
C013	N00F	C02X	108.4(4)
C02X	N00F	S005	120.5(4)
C00N	C00H	C031	120.8(5)
N009	C00K	C034	104.2(4)
C01D	N00L	S002	119.1(4)
C02S	N00L	S002	122.2(4)
C02S	N00L	C01D	108.5(4)

Atom	Atom	Atom	Angle/°
C00S	C00M	C037	118.3(6)
C00S	C00M	C03G	120.5(6)
C037	C00M	C03G	121.2(6)
C01Q	C00N	C00H	119.8(5)
C01C	C00O	C033	112.8(4)
C034	C00O	C01C	101.9(4)
C034	C00O	C033	114.1(5)
C010	C00R	C02R	103.3(4)
C023	C00R	C010	109.3(5)
C023	C00R	C02R	113.7(4)
C015	C00S	C00M	121.6(5)
C01L	C00T	C03E	122.4(5)
C01Y	C00U	C018	119.7(5)
C015	C00V	S003	120.2(4)
C01E	C00V	S003	119.7(4)
C01E	C00V	C015	120.1(5)
C03B	C00W	C025	122.5(6)
C03B	C00W	C03J	117.0(6)
C03J	C00W	C025	120.4(6)
C038	C00X	C014	120.4(6)
C013	C00Y	C03H	107.9(5)
C01M	C00Y	C013	127.7(5)
C01M	C00Y	C03H	124.4(5)
C01J	C00Z	C018	120.1(4)
N016	C010	C00R	101.9(4)
N012	C011	C01U	102.7(4)
C011	N012	S006	117.9(4)
C02G	N012	S006	119.0(4)
C02G	N012	C011	109.8(4)
N00F	C013	C00Y	104.3(4)
C00X	C014	C019	121.1(5)
C00S	C015	C00V	119.3(5)
C010	N016	S001	118.7(3)
C01T	N016	S001	118.3(3)
C01T	N016	C010	109.7(4)
C01K	C017	S006	120.9(4)
C01K	C017	C028	118.4(5)
C028	C017	S006	120.6(4)
C00U	C018	S001	120.8(4)
C00U	C018	C00Z	119.7(5)
C00Z	C018	S001	119.5(4)
C014	C019	C01X	124.9(5)
C02E	C019	C014	117.5(6)
C02E	C019	C01X	117.7(5)
C02M	C01A	C026	119.7(5)
C031	C01B	C02L	121.9(5)
N009	C01C	C00O	101.7(4)

Atom	Atom	Atom	Angle/°
N00L	C01D	C01P	102.7(4)
C037	C01E	C00V	119.8(5)
C03P	C01F	C01G	120.9(5)
C01F	C01G	C02U	118.1(5)
C03I	C01G	C01F	116.7(6)
C03I	C01G	C02U	125.2(5)
C01I	C01H	S005	119.9(4)
C01I	C01H	C02K	119.6(5)
C02K	C01H	S005	120.5(4)
C02I	C01I	C01H	120.1(5)
C00Z	C01J	C027	120.7(5)
C03J	C01K	C017	119.7(5)
C00T	C01L	C026	119.5(5)
C00Y	C01M	C01Z	130.1(5)
C02C	C01N	C01S	116.3(5)
C02W	C01N	C01S	126.0(5)
C02W	C01N	C02C	117.7(5)
C021	C01O	C02B	121.1(6)
C01X	C01P	C01D	126.5(5)
C01X	C01P	C02T	125.1(5)
C02T	C01P	C01D	108.4(4)
C00N	C01Q	S002	120.9(4)
C00N	C01Q	C02L	120.5(5)
C02L	C01Q	S002	118.6(4)
C02D	C01R	C02K	123.1(5)
C01U	C01S	C01N	130.5(5)
N016	C01T	C02R	103.6(4)
C011	C01U	C02H	109.7(4)
C01S	C01U	C011	128.1(5)
C01S	C01U	C02H	122.2(5)
C036	N01V	S004	119.1(4)
C03O	N01V	S004	117.8(3)
C03O	N01V	C036	108.5(4)
C03C	C01W	C01Z	122.5(6)
C01P	C01X	C019	130.9(5)
C00U	C01Y	C027	121.8(5)
C01W	C01Z	C01M	119.9(5)
C01W	C01Z	C020	115.3(5)
C020	C01Z	C01M	124.8(5)
C02P	C020	C01Z	122.1(5)
C022	C021	C01O	119.6(6)
C021	C022	C02J	120.5(6)
C02Z	C024	C03M	120.4(7)
C01A	C026	S004	120.4(4)
C01A	C026	C01L	119.7(6)
C01L	C026	S004	119.8(4)
C01J	C027	C030	120.9(5)
C01Y	C027	C01J	118.0(5)
C01Y	C027	C030	121.1(5)
C03B	C028	C017	119.6(5)
C02F	C029	C02Q	120.9(7)
C01O	C02B	C02V	121.0(5)
C02Z	C02C	C01N	120.4(6)
C01R	C02D	C02I	117.0(5)
C01R	C02D	C03A	121.3(5)
C02I	C02D	C03A	121.6(6)
C03F	C02E	C019	121.2(6)
C029	C02F	C02Y	121.0(6)
N012	C02G	C02H	103.3(5)
C02G	C02H	C01U	103.2(5)
C03K	C02H	C01U	111.8(5)
C03K	C02H	C02G	112.2(6)
C01I	C02I	C02D	121.8(5)
C022	C02J	C02V	121.3(5)
C01R	C02K	C01H	118.4(5)
C01B	C02L	C01Q	118.8(5)
C01A	C02M	C03E	122.1(5)
C034	C02N	C02V	131.2(5)
C02U	C02O	C03O	125.7(5)
C02U	C02O	C03T	124.8(6)
C03T	C02O	C03O	109.2(5)
C039	C02P	C020	119.9(6)
C029	C02Q	C035	118.8(7)
C01T	C02R	C00R	108.8(4)
C03D	C02R	C00R	124.6(5)
C03D	C02R	C01T	126.6(5)
N00L	C02S	C02T	100.8(4)
C01P	C02T	C02S	102.8(4)
C01P	C02T	C03R	115.8(5)
C02S	C02T	C03R	112.9(5)
C02O	C02U	C01G	130.6(5)
C02B	C02V	C02N	119.6(5)
C02J	C02V	C02B	116.3(5)
C02J	C02V	C02N	124.1(5)
C03M	C02W	C01N	121.4(5)
N00F	C02X	C03H	101.4(4)
C02F	C02Y	C03D	124.8(5)
C032	C02Y	C02F	118.4(6)
C032	C02Y	C03D	116.8(6)
C024	C02Z	C02C	120.5(6)
C00H	C031	C03L	120.1(6)
C01B	C031	C00H	118.3(5)
C01B	C031	C03L	121.6(6)
C02Y	C032	C035	120.6(7)
C00K	C034	C00O	107.3(4)
C02N	C034	C00K	126.5(5)
C02N	C034	C00O	126.2(5)
C02Q	C035	C032	120.3(7)
C03T	C036	N01V	103.7(6)
C01E	C037	C00M	120.9(5)
C00X	C038	C03F	118.8(7)
C02P	C039	C03C	119.3(6)
C00W	C03B	C028	122.8(6)
C01W	C03C	C039	120.8(6)
C02R	C03D	C02Y	131.2(5)
C00T	C03E	C02A	121.9(6)
C02M	C03E	C00T	116.5(6)
C02M	C03E	C02A	121.6(6)
C02E	C03F	C038	120.9(6)
C00Y	C03H	C02X	102.9(4)
C03Q	C03H	C00Y	115.5(6)
C03Q	C03H	C02X	115.7(5)
C03S	C03I	C01G	122.1(6)
C01K	C03J	C00W	122.5(6)
C02W	C03M	C024	119.5(6)
C03P	C03N	C03S	119.1(7)
N01V	C03O	C02O	103.6(4)
C03N	C03P	C01F	121.1(6)
C03I	C03S	C03N	120.1(6)
C036	C03T	C02O	106.8(5)
C03U	C03T	C02O	116.2(8)
C03U	C03T	C036	116.5(9)

Table: Torsion Angles in ° for **cu_GSQ_3_354_a**.

Atom	Atom	Atom	Atom	Angle/°
S001	N016	C01T	C02R	-164.8(3)
S002	N00L	C01D	C01P	170.1(3)
S002	N00L	C02S	C02T	175.5(3)
S002	C01Q	C02L	C01B	178.2(4)
S003	N009	C00K	C034	164.7(4)
S003	N009	C01C	C00O	-179.7(3)
S003	C00V	C015	C00S	-175.6(4)
S003	C00V	C01E	C037	177.0(4)
S004	N01V	C036	C03T	168.4(6)
S004	N01V	C03O	C02O	-163.2(5)
S005	N00F	C013	C00Y	165.1(3)
S005	N00F	C02X	C03H	-179.2(4)
S005	C01H	C01I	C02I	-178.8(4)
S005	C01H	C02K	C01R	179.2(4)
S006	N012	C02G	C02H	175.6(4)
S006	C017	C01K	C03J	-175.9(4)
S006	C017	C028	C03B	175.8(4)
O007	S003	N009	C00K	45.6(5)
O007	S003	N009	C01C	-176.4(4)
O007	S003	C00V	C015	-32.5(4)
O007	S003	C00V	C01E	148.9(4)
O008	S004	N01V	C036	51.6(5)
O008	S004	N01V	C03O	-173.6(4)
O008	S004	C026	C01A	162.9(4)
O008	S004	C026	C01L	-21.0(5)
N009	S003	C00V	C015	81.6(4)
N009	S003	C00V	C01E	-96.9(4)
N009	C00K	C034	C00O	4.1(6)
N009	C00K	C034	C02N	-175.2(5)
O00A	S006	N012	C011	-44.9(4)
O00A	S006	N012	C02G	177.9(4)
O00A	S006	C017	C01K	-158.1(4)
O00A	S006	C017	C028	24.4(5)
O00B	S001	N016	C010	50.5(4)
O00B	S001	N016	C01T	-172.5(3)
O00B	S001	C018	C00U	157.5(4)
O00B	S001	C018	C00Z	-24.8(5)
O00C	S005	N00F	C013	44.1(4)
O00C	S005	N00F	C02X	-178.1(4)
O00C	S005	C01H	C01I	155.6(4)
O00C	S005	C01H	C02K	-23.5(5)
O00D	S005	N00F	C013	173.9(4)
O00D	S005	N00F	C02X	-48.4(4)
O00D	S005	C01H	C01I	24.1(5)
O00D	S005	C01H	C02K	-155.0(4)
O00E	S001	N016	C010	-179.5(4)
O00E	S001	N016	C01T	-42.5(4)
O00E	S001	C018	C00U	26.9(5)
O00E	S001	C018	C00Z	-155.4(4)
N00F	S005	C01H	C01I	-90.9(5)
N00F	S005	C01H	C02K	90.0(5)
N00F	C02X	C03H	C00Y	36.6(5)
N00F	C02X	C03H	C03Q	163.5(5)
O00G	S006	N012	C011	-174.7(4)
O00G	S006	N012	C02G	48.2(4)
O00G	S006	C017	C01K	-25.9(5)
O00G	S006	C017	C028	156.6(4)
C00H	C00N	C01Q	S002	-178.2(4)
C00H	C00N	C01Q	C02L	-0.1(7)
O00I	S002	N00L	C01D	43.6(4)
O00I	S002	N00L	C02S	-175.3(4)
O00I	S002	C01Q	C00N	-22.2(5)

Atom	Atom	Atom	Atom	Angle/°
O00I	S002	C01Q	C02L	159.6(4)
O00J	S003	N009	C00K	175.2(4)
O00J	S003	N009	C01C	-46.8(5)
O00J	S003	C00V	C015	-163.6(4)
O00J	S003	C00V	C01E	17.9(5)
C00K	N009	C01C	C00O	-38.4(5)
N00L	S002	C01Q	C00N	91.9(5)
N00L	S002	C01Q	C02L	-86.2(4)
N00L	C01D	C01P	C01X	-178.2(5)
N00L	C01D	C01P	C02T	1.7(5)
N00L	C02S	C02T	C01P	38.6(5)
N00L	C02S	C02T	C03R	164.1(5)
C00M	C00S	C015	C00V	-1.6(7)
C00N	C00H	C031	C01B	-0.9(8)
C00N	C00H	C031	C03L	178.8(5)
C00N	C01Q	C02L	C01B	0.0(8)
O00P	S004	N01V	C036	-179.1(5)
O00P	S004	N01V	C03O	-44.3(5)
O00P	S004	C026	C01A	32.1(4)
O00P	S004	C026	C01L	-151.8(4)
O00Q	S002	N00L	C01D	173.4(4)
O00Q	S002	N00L	C02S	-45.5(5)
O00Q	S002	C01Q	C00N	-153.5(4)
O00Q	S002	C01Q	C02L	28.3(5)
C00R	C010	N016	S001	176.5(3)
C00R	C010	N016	C01T	36.2(5)
C00R	C02R	C03D	C02Y	178.5(5)
C00S	C00M	C037	C01E	2.6(7)
C00T	C01L	C026	S004	-176.1(4)
C00T	C01L	C026	C01A	0.1(7)
C00U	C01Y	C027	C01J	0.7(8)
C00U	C01Y	C027	C030	179.7(5)
C00V	S003	N009	C00K	-69.5(4)
C00V	S003	N009	C01C	68.6(4)
C00V	C01E	C037	C00M	-1.2(7)
C00X	C014	C019	C01X	178.4(5)
C00X	C014	C019	C02E	-1.3(8)
C00X	C038	C03F	C02E	-1.5(12)
C00Y	C01M	C01Z	C01W	171.6(6)
C00Y	C01M	C01Z	C020	-8.9(9)
C00Z	C01J	C027	C01Y	0.4(8)
C00Z	C01J	C027	C030	-178.6(6)
C010	C00R	C02R	C01T	18.9(5)
C010	C00R	C02R	C03D	-158.7(5)
C010	N016	C01T	C02R	-24.3(5)
C011	N012	C02G	C02H	35.3(6)
C011	C01U	C02H	C02G	11.1(6)
C011	C01U	C02H	C03K	-109.8(6)
N012	S006	C017	C01K	87.8(4)
N012	S006	C017	C028	-89.7(5)
N012	C011	C01U	C01S	-173.2(5)
N012	C011	C01U	C02H	9.2(5)
N012	C02G	C02H	C01U	-26.9(6)
N012	C02G	C02H	C03K	93.6(6)
C013	N00F	C02X	C03H	-37.7(5)
C013	C00Y	C01M	C01Z	-2.2(9)
C013	C00Y	C03H	C02X	-24.2(6)
C013	C00Y	C03H	C03Q	-151.2(5)
C014	C00X	C038	C03F	2.1(10)
C014	C019	C01X	C01P	-21.7(8)
C014	C019	C02E	C03F	2.0(9)
C015	C00V	C01E	C037	-1.6(7)
N016	S001	C018	C00U	-87.5(4)
N016	S001	C018	C00Z	90.2(4)
N016	C01T	C02R	C00R	2.4(5)

Atom	Atom	Atom	Atom	Angle/˚
N016	C01T	C02R	C03D	179.9(5)
C017	S006	N012	C011	70.3(4)
C017	S006	N012	C02G	-66.8(4)
C017	C01K	C03J	C00W	-0.1(9)
C017	C028	C03B	C00W	0.2(8)
C018	S001	N016	C010	-64.1(4)
C018	S001	N016	C01T	72.8(4)
C018	C00U	C01Y	C027	-2.4(8)
C018	C00Z	C01J	C027	0.2(8)
C019	C02E	C03F	C038	-0.6(11)
C01A	C02M	C03E	C00T	3.0(7)
C01A	C02M	C03E	C02A	-175.1(5)
C01C	N009	C00K	C034	21.7(6)
C01C	C00O	C034	C00K	-26.5(5)
C01C	C00O	C034	C02N	152.8(6)
C01D	N00L	C02S	C02T	-39.8(5)
C01D	C01P	C01X	C019	-0.2(8)
C01D	C01P	C02T	C02S	-25.4(5)
C01D	C01P	C02T	C03R	-149.0(5)
C01E	C00V	C015	C00S	3.0(7)
C01F	C01G	C02U	C02O	177.6(8)
C01F	C01G	C03I	C03S	-2.0(11)
C01G	C01F	C03P	C03N	0.8(11)
C01G	C03I	C03S	C03N	1.1(14)
C01H	S005	N00F	C013	-71.3(4)
C01H	S005	N00F	C02X	66.5(4)
C01H	C01I	C02I	C02D	0.2(8)
C01I	C01H	C02K	C01R	0.0(8)
C01J	C00Z	C018	S001	-179.6(4)
C01J	C00Z	C018	C00U	-1.9(8)
C01K	C017	C028	C03B	-1.8(7)
C01L	C00T	C03E	C02A	173.7(5)
C01L	C00T	C03E	C02M	-4.4(7)
C01M	C00Y	C013	N00F	-178.0(5)
C01M	C00Y	C03H	C02X	155.5(5)
C01M	C00Y	C03H	C03Q	28.5(8)
C01M	C01Z	C020	C02P	179.8(6)
C01N	C01S	C01U	C011	-1.7(9)
C01N	C01S	C01U	C02H	175.6(5)
C01N	C02C	C02Z	C024	2.2(11)
C01N	C02W	C03M	C024	1.1(8)
C01O	C021	C022	C02J	-3.0(12)
C01O	C02B	C02V	C02J	-1.8(9)
C01O	C02B	C02V	C02N	177.0(6)
C01Q	S002	N00L	C01D	-71.3(4)
C01Q	S002	N00L	C02S	69.9(4)
C01R	C02D	C02I	C01I	-1.0(8)
C01S	C01N	C02C	C02Z	176.9(6)
C01S	C01N	C02W	C03M	-178.5(5)
C01S	C01U	C02H	C02G	-166.7(5)
C01S	C01U	C02H	C03K	72.5(8)
C01T	C02R	C03D	C02Y	1.3(9)
C01U	C011	N012	S006	-168.6(3)
C01U	C011	N012	C02G	-27.8(5)
N01V	S004	C026	C01A	-82.1(4)
N01V	S004	C026	C01L	94.1(4)
N01V	C036	C03T	C02O	-23.0(10)
N01V	C036	C03T	C03U	108.7(11)
C01W	C01Z	C020	C02P	-0.8(9)
C01X	C019	C02E	C03F	-177.7(6)
C01X	C01P	C02T	C02S	154.5(5)
C01X	C01P	C02T	C03R	31.0(8)
C01Y	C00U	C018	S001	-179.4(4)
C01Y	C00U	C018	C00Z	2.9(7)
C01Z	C01W	C03C	C039	1.4(11)

Atom	Atom	Atom	Atom	Angle/˚
C01Z	C020	C02P	C039	1.8(10)
C020	C02P	C039	C03C	-1.1(10)
C021	C01O	C02B	C02V	2.1(10)
C021	C022	C02J	C02V	3.3(12)
C022	C02J	C02V	C02B	-0.9(10)
C022	C02J	C02V	C02N	-179.6(6)
C023	C00R	C010	N016	89.2(5)
C023	C00R	C02R	C01T	-99.4(5)
C023	C00R	C02R	C03D	83.0(7)
C025	C00W	C03B	C028	-177.3(6)
C025	C00W	C03J	C01K	177.2(6)
C026	S004	N01V	C036	-64.0(5)
C026	S004	N01V	C03O	70.8(5)
C026	C01A	C02M	C03E	-0.1(7)
C028	C017	C01K	C03J	1.7(8)
C029	C02F	C02Y	C032	-0.7(9)
C029	C02F	C02Y	C03D	179.8(5)
C029	C02Q	C035	C032	-0.7(10)
C02B	C01O	C021	C022	0.3(11)
C02C	C01N	C01S	C01U	-163.3(6)
C02C	C01N	C02W	C03M	1.4(8)
C02D	C01R	C02K	C01H	-1.0(9)
C02E	C019	C01X	C01P	158.0(6)
C02F	C029	C02Q	C035	-0.9(10)
C02F	C02Y	C032	C035	-0.9(9)
C02F	C02Y	C03D	C02R	6.1(9)
C02K	C01H	C01I	C02I	0.3(8)
C02K	C01R	C02D	C02I	1.4(9)
C02K	C01R	C02D	C03A	-178.5(6)
C02L	C01B	C031	C00H	0.9(8)
C02L	C01B	C031	C03L	-178.8(6)
C02M	C01A	C026	S004	174.7(4)
C02M	C01A	C026	C01L	-1.5(7)
C02P	C039	C03C	C01W	-0.4(11)
C02Q	C029	C02F	C02Y	1.6(9)
C02R	C00R	C010	N016	-32.1(5)
C02S	N00L	C01D	C01P	24.1(5)
C02T	C01P	C01X	C019	179.9(5)
C02U	C01G	C03I	C03S	179.4(8)
C02U	C02O	C03O	N01V	-177.0(7)
C02U	C02O	C03T	C036	-164.9(8)
C02U	C02O	C03T	C03U	63.2(15)
C02V	C02N	C034	C00K	-4.0(10)
C02V	C02N	C034	C00O	176.8(5)
C02W	C01N	C01S	C01U	16.6(9)
C02W	C01N	C02C	C02Z	-3.0(9)
C02X	N00F	C013	C00Y	22.7(5)
C02Y	C032	C035	C02Q	1.6(10)
C02Z	C024	C03M	C02W	-2.1(10)
C031	C00H	C00N	C01Q	0.6(8)
C031	C01B	C02L	C01Q	-0.4(8)
C032	C02Y	C03D	C02R	-173.4(6)
C033	C00O	C01C	N009	161.3(4)
C033	C00O	C034	C00K	-148.3(5)
C033	C00O	C034	C02N	31.0(8)
C034	C00O	C01C	N009	38.5(5)
C034	C02N	C02V	C02B	-180.0(6)
C034	C02N	C02V	C02J	-1.2(10)
C036	N01V	C03O	C02O	-23.9(7)
C037	C00M	C00S	C015	-1.1(7)
C038	C00X	C014	C019	-0.7(9)
C03A	C02D	C02I	C01I	178.9(6)
C03B	C00W	C03J	C01K	-1.4(9)
C03C	C01W	C01Z	C01M	178.7(6)
C03C	C01W	C01Z	C020	-0.8(9)

Atom	Atom	Atom	Atom	Angle/˚
C03D	C02Y	C032	C035	178.7(5)
C03E	C00T	C01L	C026	3.0(8)
C03G	C00M	C00S	C015	177.6(5)
C03G	C00M	C037	C01E	-176.2(5)
C03H	C00Y	C013	N00F	1.7(5)
C03H	C00Y	C01M	C01Z	178.1(5)
C03I	C01G	C02U	C02O	-3.8(13)
C03J	C00W	C03B	C028	1.4(8)
C03M	C024	C02Z	C02C	0.4(11)
C03O	N01V	C036	C03T	29.8(8)
C03O	C02O	C02U	C01G	2.7(14)
C03O	C02O	C03T	C036	9.0(10)
C03O	C02O	C03T	C03U	-122.9(11)
C03P	C01F	C01G	C02U	179.7(7)
C03P	C01F	C01G	C03I	1.1(10)
C03P	C03N	C03S	C03I	0.8(14)
C03S	C03N	C03P	C01F	-1.8(13)
C03T	C02O	C02U	C01G	175.6(8)
C03T	C02O	C03O	N01V	9.2(8)

Table: Hydrogen Fractional Atomic Coordinates (×10^4) and Equivalent Isotropic Displacement Parameters (Å^2×10^3) for **cu_GSQ_3_354_a**. U_{eq} is defined as 1/3 of the trace of the orthogonalised U_{ij}.

Atom	x	y	z	U_{eq}
H00H	8409.33	12660.86	3190.44	68
H00A	7557.7	742.75	8261.75	66
H00B	7295.61	680.42	7157.18	66
H00N	6583.68	12023.57	3183.1	67
H00O	6049.7	2219.29	7333.4	70
H00R	5974.12	3995.42	5081.43	72
H00S	9633.28	579.27	10346.93	73
H00T	7269.28	-2526.41	3386.39	81
H00U	8205.87	4241.35	1858.51	63
H00X	4541.39	8642.62	199.72	83
H00Z	5018.97	2993.13	1600.74	65
H01A	5711.95	3601.77	3498.05	71
H01B	5156.2	4422.17	3595.79	71
H01U	3342.95	1619.61	1538.85	63
H01V	2076.49	1199.21	1296.78	63
H01P	-408.88	6326.24	1067.17	65
H01Q	-632.75	5120.46	804.37	65
H014	4309.66	8861.54	1743.04	68
H015	9293.58	836.17	8834.8	64
H01L	8427.95	265.73	5783.37	65
H01E	8830.55	12591.34	5930	81
H01H	7578.73	3433.47	8224.45	77
H01I	7494.15	2740.66	9040.3	77
H01F	5500.15	9911.22	3257.69	62
H01G	4252.9	9643.51	3029.09	62
H01K	9926.61	3839.08	9775.03	75
H01M	14170.74	2624.08	6509.66	80
H01R	2575.61	8175.8	3005.16	70
H01J	5021.16	1400.69	1019.5	70
H01Z	705.36	-848.68	-1396.5	73
H01N	7816.33	-2736.45	4857.65	75
H01S	-164.28	6109.46	-1420.33	72
H01O	3392.62	-3235.29	7779.72	90
H01T	-584.69	8544.34	3325.72	76
H01	2994.22	4037.03	1300.38	69
H01C	8209.7	5948.54	3891.84	61
H01D	8020.47	4763.09	3547.28	61
H01W	-1705.93	6117.85	-2281.58	84

Atom	x	y	z	U_{eq}
H01X	4346.56	7101.8	3322.27	67
H01Y	8199.03	2665.78	1236.58	70
H020	-2003.98	5215.47	239.71	74
H021	4741.52	-3756.23	7466.56	91
H022	6405.23	-2537.83	7425.69	95
H02A	6054.26	5998.33	4805.23	116
H02B	6292.1	5743.08	5817.51	116
H02C	5145.56	5155.23	5226.95	116
H024	2828.38	3887.09	5103.3	95
H02X	-2395.88	-2504.41	475.93	138
H02Y	-2567.39	-2313.68	-571.84	138
H	-2407.18	-1421.85	272.15	138
H028	1220.19	-836.13	1380.95	72
H029	11418.32	6264.03	4714.43	86
H02I	7964.64	-785.67	2347.54	126
H02K	7257.49	-177.11	2689.53	126
H02M	6730.89	-1398.51	2364.76	126
H02H	3674.46	-1503.16	8036.85	77
H02Z	2674.3	4846.15	2638.11	83
H02E	4911.4	6339.89	2084.26	83
H02F	9616.89	5812.91	4418.28	79
H02	1628.64	1222.69	-704.5	82
HA	2714.83	1492.48	-1143.7	82
H5	2212.51	2953.21	-308.26	91
H02P	2572.25	9772.48	3529.42	73
H02J	6692.76	-800.41	7561.49	93
H02R	-620.44	6917.71	2811.57	73
H02L	7004.7	11956.01	5937.82	76
H02O	7941.75	477.92	4287.23	70
H02N	4731.1	241.27	8041.37	74
H02S	-3787.28	4883.79	-4.75	85
H02Q	12176.55	5971.37	6081.02	101
H02D	4849.87	9798.61	5727.78	76
H02G	5847.27	9771.4	5239.01	76
H02T	3681.02	8353.46	4743.52	77
H02U	13054.52	901.23	6280.35	98
H6	3259.68	2276.53	2833	68
H02V	2425.92	6553.61	1051.24	78
H02W	1887.52	7387.78	1033.4	78
H7	2563.32	5033.28	4221.87	102
H03A	6658.34	448.1	1175.98	122
H03B	7239.15	948.64	345.67	122
H03C	5978.03	451.67	235.18	122

Atom	x	y	z	U_{eq}
H032	9287.06	4868.2	6909.55	85
H03L	5569.57	2110.72	9209.43	103
H03M	4699.81	1816.77	8314.98	103
H03N	5509.9	2986.24	8632.07	103
H035	11097.77	5251.94	7175.43	102
H03R	10284.24	-2274.67	6204.48	106
H03S	10058.17	-1708.29	5364.36	106
H037	10332.58	3585.62	11277.73	81
H038	5026.59	7316.46	-396.5	98
H039	-4545.08	5103.57	-1408.41	91
H1	282.24	10355.43	3705.13	124
HB	1494.79	10890.94	3567.01	124
HC	1192.84	10512.44	4531.58	124
H8	-578.01	-1520.27	1390.1	78
H2	-3482.11	5714.41	-2540.28	94
H03D	7790.42	4795.46	6045.48	74
H03F	5171.48	6146.97	560.31	106
H03O	9515.71	1408.27	12116.58	129
H03P	10461.22	2518.13	12362.98	129
H03Q	10691.95	1530.35	11978.2	129
H3	1391.87	5554.09	-283.03	83
H03T	11173.08	1950.21	6981.69	97
H9	-1072.98	-1532.18	-1342.09	80
H10	4091.96	4002.2	39.84	151
HD	3686	3476.7	-1022.23	151
HE	4283.37	2992.49	-347.81	151
H03E	10331.68	13634.52	5073.63	131
H03G	10139.08	13302.93	3970.23	131
H03H	10214.88	12503.36	4634.81	131
H11	3227.56	2523.48	4419.38	81
H03U	13233.05	4890.87	7349.94	116
H03V	10610.17	460.93	7339.95	74
H03W	10213.75	393.22	6256.94	74
H03X	14511.27	4348.58	6875.93	97
H4	1514.96	6556.77	-1456.06	134
HF	2596.18	7018.18	-755.58	134
HG	1769.36	7562.59	-706.81	134
H03I	4347.02	7031.94	4823.49	130
H03J	4661.44	7782.87	5798.58	130
H03K	5520.11	7884.46	5107.36	130
H03Y	11540.32	3671.5	7384.34	115
H03Z	11708.07	-905.96	5494.57	144
H03	12849.92	-660.27	6894.39	339

**Atom**	**x**	**y**	**z**	***U_{eq}***
HH	12389.37	-1729.25	6195.22	339
HI	11871.56	-1612.16	7116.54	339
