# Supplementary material for: Cobalt-catalyzed enantioselective intramolecular reductive cyclization via electrochemistry
Source: Nat Commun. 2023 Mar 9;14:1301. doi: 10.1038/s41467-023-36704-9 (PMC9998880; doi:10.1038/s41467-023-36704-9)
Supplement: Supplementary file 4 — Supplementary Data 2 [file 41467_2023_36704_MOESM4_ESM.pdf]

**Energy data (hartrees) for the calculated structures.**

| Geometry | $E_{M06-L/6-31G(d)}$ | TCG | $E_{M06-L/6-311+G(d,p)}$ | TCG+$E_{M06-L/6-311+G(d,p)}$ | Imaginary Frequency |
|---|---|---|---|---|---|
| INT1A-s | -2373.11890329 | 0.579806 | -2373.83983062 | -2373.260025 | |
| INT1A-t | -2373.15590847 | 0.573631 | -2373.87321512 | -2373.299584 | |
| INT1B-s | -2373.25728908 | 0.574127 | -2373.84599350 | -2373.271867 | |
| INT1B-t | -2373.27471562 | 0.568769 | -2373.86369039 | -2373.294921 | |
| $H_2O$ | -76.3986473653 | 0.003919 | -76.4493343 | -76.44541529 | |
| INT1-s | -2373.01498778 | 0.583376 | -2373.85730281 | -2373.273927 | |
| INT1-t | -2373.11664811 | 0.578995 | -2373.87233058 | -2373.293336 | |
| INT2-s | -2373.32490070 | 0.584197 | -2373.86118869 | -2373.276992 | |
| INT2-t | -2373.29962685 | 0.577664 | -2373.85958603 | -2373.281922 | |
| INT3-s | -2449.67341898 | 0.607477 | -2450.32642895 | -2449.718952 | |
| INT3-t | -2449.72961386 | 0.599684 | -2450.31242409 | -2449.71274 | |
| INT4-s | -2449.69292886 | 0.608906 | -2450.34206472 | -2449.733159 | |
| INT4-t | -2449.74373186 | 0.606515 | -2450.33483553 | -2449.728321 | |
| INT5-s | -2526.15965283 | 0.631480 | -2526.79596887 | -2526.170988 | |
| INT5-t | -2526.17704806 | 0.619937 | -2526.78213501 | -2526.162198 | |
| INT6-s | -2526.17085167 | 0.624981 | -2526.79695638 | -2526.171975 | |
| INT6-t | -2526.19384897 | 0.624586 | -2526.81629869 | -2526.191713 | |
| INT7-s | -2449.73611953 | 0.606342 | -2450.30588859 | -2449.699547 | |
| INT7-t | -2449.38831139 | 0.600267 | -2450.31441837 | -2449.714151 | |
| INT8-s | -2449.72237863 | 0.600034 | -2450.29716147 | -2449.697127 | |
| INT8-t | -2449.75115234 | 0.606572 | -2450.31697728 | -2449.710405 | |
| INT8'-s | 2449.73028556 | 0.606451 | -2450.30810442 | -2449.701653 | |
| INT8'-t | -2449.73024715 | 0.598693 | -2450.30041074 | -2449.701718 | |
| INT9-s | -2449.48388624 | 0.605135 | -2450.29316839 | -2449.688033 | |
| INT9-t | -2449.49274597 | 0.603546 | -2450.30288942 | -2449.699343 | |
| INT10-s | -2449.72711750 | 0.604320 | -2450.33317309 | -2449.728853 | |
| INT10-t | -2449.71859420 | 0.599534 | -2450.33046714 | -2449.730933 | |
| INT11-s | -2526.18287279 | 0.629701 | -2526.79818133 | -2526.16848 | |
| INT11-t | -2526.15257878 | 0.623421 | -2526.7860440 | -2526.162623 | |
| TS1-s | -2373.31739944 | 0.580162 | -2373.84912451 | -2373.268963 | -170.23i |
| TS1-t | -2373.30795134 | 0.578466 | -2373.84235012 | -2373.263884 | -209.29i |
| TS2-s | -2449.73048845 | 0.603015 | -2450.29762372 | -2449.694609 | -1377.72i |
| TS2-t | -2449.69904273 | 0.599640 | -2450.28146333 | -2449.681823 | -1441.17i |
| TS2'-s | -2449.69903591 | 0.602659 | -2450.26694709 | -2449.664288 | -1308.69i |
| TS2'-t | -2449.73319493 | 0.600230 | -2450.29860175 | -2449.698372 | -837.12i |
| TS3-s | -2526.15402575 | 0.623875 | -2526.75296463 | -2526.12909 | -1178.22i |
| TS3-t | -2526.17390887 | 0.620032 | -2526.77429328 | -2526.154261 | -922.91i |
| TS4-s | -2449.69328689 | 0.591320 | -2450.27116359 | -2449.679844 | -1664.15i |
| TS4-t | -2449.65303549 | 0.587658 | -2450.26822122 | -2449.680563 | -1766.19i |
| TS5-s | -2449.72163244 | 0.604597 | -2450.29189242 | -2449.687295 | -240.03i |
| TS5-t | -2449.71337268 | 0.602139 | -2450.30030355 | -2449.698165 | -297.78i |

| | | | | | |
|---|---|---|---|---|---|
| TS6-s | -2449.69204117 | 0.600216 | -2450.26052778 | -2449.660312 | -1414.96i |
| TS6-t | -2449.68436017 | 0.593187 | -2450.25454487 | -2449.661358 | -1506.13i |
| TS7-s | -2449.69050965 | 0.603471 | -2450.28361895 | -2449.680148 | -184.39i |
| TS7-t | -2449.71127231 | 0.598567 | -2450.28151193 | -2449.682945 | -346.00i |
| TS8-s | -2449.48388624 | 0.605135 | -2450.29316839 | -2449.688033 | -1540.09i |
| TS8-t | -2449.49274597 | 0.603546 | -2450.30288942 | -2449.699343 | -1334.42i |
| MECP1 | -2373.10552386 | | | | |
| MECP2 | -2526.17964078 | | | | |
